# Supplementary material for: Interventions to improve primary healthcare in rural settings: A scoping review
Source: PLoS One. 2024 Jul 11;19(7):e0305516. doi: 10.1371/journal.pone.0305516 (PMC11239038; doi:10.1371/journal.pone.0305516)
Supplement: S1 Appendix — (PDF) [file pone.0305516.s002.pdf]

Appendix 1:

Sample search strategy for Medline

Search ((((("Rural Population"[Mesh] OR "Rural Health"[Mesh] OR "Rural Health Services"[Mesh] OR "rural health" OR "rural health care" OR "rural healthcare" OR "rural population"))) AND (("Program Evaluation"[Mesh] OR "Quality Assurance, Health Care"[Mesh:NoExp] OR "Quality Improvement"[Mesh] OR "Outcome and Process Assessment (Health Care)"[Mesh:NoExp] OR "Utilization Review"[Mesh:NoExp] OR "Organizational Case Studies"[Mesh] OR "Evaluation Studies as Topic"[Mesh:NoExp] OR "Peer Review, Health Care"[Mesh] OR "Comprehensive Health Care"[Mesh:NoExp] OR "Delivery of Health Care"[Mesh:NoExp] OR "Patient Care Team"[Mesh:NoExp] OR program OR quality OR utilization OR evaluation OR delivery OR team OR "patient care"))) AND (("Primary Health Care"[Mesh] OR "General Practice"[Mesh] OR "primary care" OR "primary health care" OR "general practice")))
