# Supplementary material for: Interventions to improve primary healthcare in rural settings: A scoping review
Source: PLoS One. 2024 Jul 11;19(7):e0305516. doi: 10.1371/journal.pone.0305516 (PMC11239038; doi:10.1371/journal.pone.0305516)
Supplement: S2 Appendix — (DOCX) [file pone.0305516.s003.docx]

**Table of article characteristics**

| Author, Year | Country | Study Design | Health Topic | Intervention Code | Access Outcomes | | | | Quality Outcomes | | | | | Efficiency Outcomes | | |
| --- | --- | --- | --- | --- | --- | --- | --- | --- | --- | --- | --- | --- | --- | --- | --- | --- |
|  |  |  |  |  | Recruitment | Retention | Alternative PCP | Services | EBM | Clinical | Safety | Experience | Equity | Wait Times | Health Care Use | Cost Savings |
| Ansari, 2022(22) | Pakistan | Prospective cohort | Diabetes | Patient education + reorganization of services |  |  |  |  |  | X |  |  |  |  |  |  |
| Burdick, 2022(23) | United States | UBA | N/A | Coordination/referral pathways + reorganization of services |  |  |  |  |  |  |  |  |  |  | X |  |
| Gur, 2022(24) | Israel | RCT | N/A | Well-being |  |  |  |  |  |  |  | X |  |  | X |  |
| Harding, 2022(25) | Australia | UBA | Weight management | Implementing a new service |  |  |  |  |  |  |  |  |  |  |  |  |
| Harrison, 2022(26) | United States | UBA | Mental health | Provider training |  |  |  |  | X |  |  |  |  |  |  |  |
| Harry, 2022(27) | United States | Cluster RCT | Vaccination | Decision support |  |  |  |  | X |  |  |  |  |  |  |  |
| Ndayizigiye, 2022(28) | Lesotho | CBA | Mental health | Patient education + implementing a new service |  |  |  |  |  | X |  |  |  |  |  |  |
| Okonofua, 2022(29) | Nigeria | UBA | Maternal & child health | Provider training + coordination/referral pathways + transportation + increasing resources |  |  |  | X |  |  |  |  |  |  |  |  |
| Osae, 2022(30) | United States | Retrospective cohort | Any condition | Expanded scope of practice |  |  |  |  |  | X |  |  |  |  |  |  |
| Renn, 2022(31) | United States | UBA | Mental health | Implementing a new service + coordination/referral pathways |  |  |  |  |  | X |  |  |  |  |  |  |
| Srinivasan, 2022(32) | India | Cluster RCT | Mental health | Provider training + reorganization of services + coordination/referral pathways |  |  |  |  |  | X |  |  |  |  |  |  |
| Befort, 2021(33) | United States | RCT | Weight management | Patient education |  |  |  |  |  | X |  |  |  |  |  |  |
| Camargo, 2021(34) | Brazil | Cohort | Diabetes | Implementing a new service |  |  |  |  |  | X |  |  |  |  |  |  |
| Chen, 2021(35) | China | RCT | Diabetes | Patient education + provider training |  |  |  |  | X | X |  |  |  |  |  |  |
| Chevillard, 2021(36) | France | Cohort | N/A | Reorganization of services + well-being | X |  |  |  |  |  |  |  |  |  |  |  |
| deBatlle, 2021(37) | Spain | CBA | Any condition (elderly) | Reorganization of services |  |  |  |  |  | X |  |  |  |  | X | X |
| Deng, 2021(38) | China | RCT | Limbs disability | Reorganization of services |  |  |  |  |  | X |  |  |  |  |  |  |
| Katzmarzyk, 2021(39) | United States | Cluster RCT | Obesity | Patient education |  |  |  |  |  | X |  |  |  |  |  |  |
| Kirkland, 2021(40) | United States | Cohort | Diabetes | Telehealth/virtual care |  |  |  |  |  | X |  |  |  |  |  |  |
| Muyambi, 2021(41) | Australia | CBA | Mental health | Provider training |  |  |  |  | X |  |  |  |  |  |  |  |
| OgobaraDougnon, 2021(42) | Niger | RCT | Malnutrition | Training of lay-persons |  |  |  |  |  |  |  |  |  |  | X |  |
| Pati (A), 2021(43) | India | Quasi-randomised cluster trial | NCD | Provider training + coordination/referral pathways |  |  |  |  |  |  |  |  |  |  | X |  |
| Pati (B), 2021(43) | India | Quasi-randomised cluster trial | NCD | Provider training + coordination/referral pathways + patient education |  |  |  |  |  |  |  |  |  |  | X |  |
| Poudiougou, 2021(44) | Mali | UBA | Mental health | Provider training |  |  |  |  | X | X |  |  |  |  |  |  |
| Schepens Niemiec, 2021(45) | United States | UBA | Lifestyle improvement | Implementing a new service + patient education |  |  |  |  |  | X |  |  |  |  |  |  |
| Stallings , 2021(46) | United States | UBA | Hypertension | Expanded scope of practice + patient education |  |  |  |  |  | X |  |  |  |  |  |  |
| Sylaja, 2021(47) | India | Cluster RCT | Stroke | Training of lay-persons |  |  |  |  |  | X |  | X |  |  |  |  |
| Wagner, 2021(48) | South Africa | Markov model | Epilepsy | Provider training + training lay-persons |  |  |  |  |  |  |  |  |  |  |  | X |
| Zittleman, 2021(49) | United States | UBA | Mental health | Provider training |  |  |  |  |  | X |  |  |  |  |  |  |
| Ameh, 2020(50) | South Africa | Cross-sectional | HIV and hypertension | Reorganization of services |  |  |  |  |  | X |  | X |  |  |  |  |
| Bellesheim, 2020(51) | United States | Cohort | Mental health | Provider education |  |  |  |  | X | X |  |  |  |  |  |  |
| Bowen, 2020(52) | United States | Cohort | Mental health | Reorganization of services |  |  |  |  | X | X |  |  | X |  |  |  |
| Brown, 2020(53) | United States | UBA | Weight management | Telehealth/virtual care + patient education |  |  |  |  |  | X |  | X |  |  |  |  |
| Chen, 2020(54) | China | RCT | Diabetes | Patient education |  |  |  |  |  |  |  |  |  |  |  |  |
| Chen, 2020 (55) | China | RCT | Diabetes | Provider training |  |  |  |  | X |  |  |  |  |  |  |  |
| Deng, 2020(56) | China | RCT | Stroke | Implementing a new service |  |  |  |  |  | X |  |  |  |  |  |  |
| Espinet, 2020(57) | Canada | RCT | Mental health | Provider training |  |  |  |  | X |  |  |  |  |  |  |  |
| Gordon, 2020(58) | United States | RCT | Diabetes | Patient education |  |  |  |  |  | X |  |  |  |  |  |  |
| Gu, 2020(59) | China | UBA | Any condition | Provider training |  |  |  |  | X |  |  |  |  |  |  |  |
| Hine, 2020(60) | United States | UBA | Mental health | Reorganization of services + provider training |  |  |  |  |  |  |  |  |  | X |  |  |
| Kitchener, 2020(61) | Australia | Cohort | N/A | Medical education - exposure |  | X |  |  |  |  |  |  |  |  |  |  |
| Larson, 2020(62) | Tanzania | Cluster RCT | Maternal & child health | Provider training + coordination/referral pathways + increasing resources |  |  |  |  | X | X |  |  |  |  |  |  |
| LeCloux, 2020(63) | United States | UBA | Mental health | Screening |  |  |  |  |  | X |  |  |  |  |  |  |
| Morris-Paxton, 2020(64) | South Africa | CBA | Any condition | Implementing a new service |  |  |  |  |  |  |  |  |  |  | X |  |
| Myers, 2020(65) | United States | Case series | Any condition | Expanded scope of practice + implementing a new service |  |  |  |  |  |  |  |  |  |  | X |  |
| Nagykaldi, 2020(66) | United States | UBA | N/A | Coordination/referral pathways + implementing a new service |  |  |  |  |  |  |  |  |  |  | X | X |
| Orchard, 2020(67) | Australia | Cross-sectional | Atrial fibrillation | Decision support + telehealth/virtual care |  |  |  |  | X | X |  |  |  |  |  | X |
| Peckens, 2020(68) | United States | Cohort | Diabetes | Screening + patient education + provider training |  |  |  | X |  | X |  |  |  |  |  |  |
| Powers, 2020(69) | United States | Cohort | Mental health | Reorganization of services |  |  |  |  |  | X |  |  |  |  |  |  |
| Prudencio, 2020(70) | United States | UBA | Diabetes | Implementing a new service + patient education |  |  |  |  | X | X |  |  |  |  |  |  |
| Pu, 2020(71) | China | UBA | N/A | Financial incentive |  |  |  |  |  |  |  |  |  |  | X |  |
| Smith, 2020(72) | Rwanda | UBA | Mental health | Implementing a new service |  |  |  |  |  | X |  |  |  |  |  |  |
| Stack, 2020(73) | United States | CBA | Pain | Implementing a new service + patient education |  |  |  |  |  | X |  |  |  |  |  |  |
| Worster, 2020(74) | Mexico | Prospective cohort | Diabetes & hypertension | Implementing a new service + training of lay-persons |  |  |  |  |  | X |  |  |  |  |  |  |
| Yapa, 2020(75) | South Africa | Cluster RCT | HIV | Provider training + training of lay-persons |  |  |  |  | X | X |  |  |  |  |  |  |
| AeyoungSo, 2019(76) | Korea | RCT | Urinary incontinence | Patient education |  |  |  |  |  | X |  |  |  |  |  |  |
| Anastasaki, 2019(77) | Greece | UBA | COPD | Patient education |  |  |  |  |  | X |  |  |  |  |  |  |
| Belcher, 2019(78) | Saudi Arabia | UBA | Diabetes | Patient education |  |  |  |  |  | X |  |  |  |  |  |  |
| Buist, 2019(79) | Scotland | UBA | Mental health | Expanded scope of practice |  |  | X |  |  | X |  |  |  |  |  |  |
| Campbell, 2019(80) | Australia | Cohort | N/A | Medical education (exposure) | X |  |  |  |  |  |  |  |  |  |  |  |
| Chen, 2019(81) | China | RCT | Diabetes | Patient education + provider training |  |  |  |  | X | X |  |  |  |  |  |  |
| Chevillard, 2019(82) | France | Cohort | N/A | Financial incentive | X | X |  |  |  |  |  |  |  |  |  |  |
| Chiswell, 2019(83) | United States | UBA | Respiratory tract infections | Patient education |  |  |  |  | X |  |  |  |  |  |  |  |
| Cummings, 2019(84) | United States | RCT | Depression & diabetes | Implementing a new service |  |  |  |  |  | X |  |  |  |  |  |  |
| Dahlberg, 2019(85) | United States | UBA | Dental care | Expanded scope of practice |  |  |  |  | X |  |  |  |  |  | X | X |
| Gatakaa, 2019(86) | Kenya | Cross-sectional | Pregnancy | Implementing a new service |  |  |  | X |  | X |  |  |  |  |  |  |
| Hanlon, 2019(87) | Ethiopia | Cohort | Mental health | Provider training |  |  |  |  |  | X |  |  | X |  |  |  |
| JabbariBeyrami, 2019(88) | Iran | ITS | Maternal & child health | Financial incentive + increasing resources + expanded scope of practice |  |  |  | X |  | X |  |  |  |  |  |  |
| Jarrett, 2019(89) | United States | UBA | Any condition | Coordination/referral pathways |  |  |  |  |  |  | X |  |  |  |  |  |
| King, 2019(90) | United States | UBA | Diabetes | Reorganization of services |  |  |  |  |  | X |  |  |  |  |  |  |
| Logan, 2019(91) | United States | UBA | Mental health | Reorganization of services |  |  |  |  |  | X |  |  |  |  |  |  |
| McLendon, 2019(92) | United States | UBA | Diabetes | Patient education + implementing a new service + telemedicine/virtual care |  |  |  |  |  | X |  | X |  |  |  |  |
| Moe, 2019(93) | Canada | Cross-sectional | Any condition | Increasing resources + reorganization of services |  |  |  |  |  |  |  |  |  |  | X |  |
| MurphyBuschkoetter, 2019(94) | United States | UBA | Diabetes | Decision support + provider training |  |  |  |  | X | X |  |  |  |  |  |  |
| Mutiso, 2019(95) | Kenya | UBA | Mental health | Provider training + training of lay-persons |  |  |  |  |  |  |  |  | X |  |  |  |
| Parchman, 2019(96) | United States | ITS | Mental health | Reorganization of services + provider training |  |  |  |  |  | X |  |  |  |  |  |  |
| Patel, 2019(97) | Indonesia | CBA | CVD | Decision support + screening + coordination/referral pathways + telehealth/virtual care |  |  |  |  | X | X |  |  |  |  |  |  |
| Reed, 2019(98) | United States | RCT | Weight management | Implementing a new service |  |  |  |  |  | X |  |  |  |  |  |  |
| Ward, 2019(99) | Australia | Cluster RCT | STI | Audit and feedback + financial incentive |  |  |  |  |  | X |  |  |  |  | X |  |
| Wei, 2019(100) | China | UBA | Diabetes | Provider training |  |  |  |  | X |  |  |  |  |  |  |  |
| Wei, 2019(101) | China | Cluster RCT | Respiratory tract infection | Provider training + patient education |  |  |  |  | X |  |  |  |  |  |  |  |
| Wong, 2019(102) | United States | Retrospective cohort | Mental health | Reorganization of services |  |  |  |  |  | X |  |  |  |  |  |  |
| Zheng, 2019(103) | China | UBA | Hypertension | Provider training |  |  |  |  | X | X |  |  |  |  |  |  |
| Amritanand, 2018(104) | India | UBA | Vision impairment | Training of lay-persons |  |  |  |  | X | X |  |  |  |  |  |  |
| Boltz, 2018(105) | United States | Cohort | Health system performance | Decision support |  |  |  |  |  | X |  |  |  |  |  |  |
| Bonnell, 2018(106) | Dominican Republic | UBA | Family planning | Training of lay-persons |  |  | X | X | X | X |  |  |  |  |  |  |
| Bonsignore, 2018(106) | United States | Cohort | Palliative care | Telehealth/virtual care |  |  |  |  |  | X |  | X |  |  |  |  |
| Boon, 2018(107) | South Africa | Prospective cohort | Mental health | Expanded scope of practice |  |  | X |  |  | X |  |  |  |  |  |  |
| Bouchonville, 2018(108) | United States | Cohort | Diabetes | Provider training |  |  |  |  | X |  |  |  |  |  |  |  |
| Causer, 2018(109) | Australia | RCT | STI | Implementing a new service |  |  |  |  |  | X |  |  |  |  |  |  |
| Collins, 2018(110) | Ireland | UBA | Mental health | Implementing a new service |  |  |  |  |  | X |  |  |  |  |  |  |
| Dragomani, 2018(111) | United States | Cross-sectional | Mental health | Implementing a new service |  |  |  |  |  | X |  |  |  |  |  |  |
| Falamic, 2018(112) | Croatia | RCT | Medication management | Expanded scope of practice |  |  | X |  |  | X |  |  |  |  |  |  |
| Gao, 2018(113) | China | Cross-sectional | Childhood diarrhea | Implementing a new service + screening |  |  |  |  |  | X |  |  |  |  |  |  |
| Giuliano, 2018(114) | Bolivia | UBA | Epilepsy | Provider training |  |  |  |  | X |  |  |  |  |  |  |  |
| Goldstein, 2018(115) | United States | UBA | Medication management | Patient education |  |  |  |  |  | X |  |  |  |  |  |  |
| Hocking, 2018(116) | Australia | Cluster RCT | STI | Provider training + Audit and feedback |  |  |  |  |  | X |  |  |  |  |  |  |
| Hotu, 2018(117) | Australia | UBA | Diabetes | Implementing a new service |  |  |  |  |  | X |  |  |  |  |  |  |
| Howe, 2018(118) | United States | UBA | Veteran's healthcare | Provider training |  |  |  |  | X |  |  | X |  |  |  |  |
| Khalil, 2018(119) | Australia | UBA | Medication management | Provider training |  |  |  |  | X |  |  |  |  |  |  |  |
| Kirby, 2018(120) | Australia | Retrospective cohort | Speech impairments | Reorganization of services |  |  |  | X | X | X |  |  |  |  |  |  |
| Kramer, 2018(121) | United States | Cohort | Any condition | Reorganization of services |  |  |  |  |  | X |  |  |  |  | X |  |
| Kranker, 2018(122) | United States | Cohort | N/A | Coordination/referral pathways |  |  |  |  |  |  |  |  |  |  | X | X |
| Lutes, 2018(123) | United States | RCT | Diabetes & depression | Reorganization of services |  |  |  |  |  | X |  |  |  |  |  |  |
| Mallow, 2018(124) | United States | UBA | Mental health | Telehealth/virtual care + patient education |  |  |  |  |  | X |  |  |  |  |  |  |
| Martinez, 2018(125) | Guatemala | RCT | Family planning | Decision support + provider training |  |  |  |  | X |  |  |  |  |  |  |  |
| Meade, 2018(126) | United States | UBA | Diabetes | Expanded scope of practice + Implementing a new service + Patient education |  |  |  |  |  | X |  |  |  |  |  |  |
| Naidoo, 2018(127) | South Africa | Cross-sectional | HIV | Training of lay-persons |  |  |  | X | X |  |  |  |  |  |  |  |
| Prabhakaran, 2018(128) | India | Cluster RCT | Chronic disease | Patient education |  |  |  |  |  | X |  |  |  |  |  |  |
| Rojas, 2018(129) | Chile | UBA | Mental health | Co-ordination/referral pathways |  |  |  |  |  | X |  | X |  |  |  |  |
| Saleh, 2018(130) | Lebanon | Cross-sectional | Diabetes & hypertension | Co-ordination/referral pathways |  |  |  |  |  | X |  |  |  |  |  |  |
| Saleh, 2018(131) | Lebanon | RCT | Non-communicable disease | Co-ordination/referral pathways |  |  |  | X |  |  |  |  |  |  |  |  |
| SchepensNiemiec, 2018(132) | United States | UBA | Preventative care | Patient education + telehealth/virtual care |  |  |  |  |  | X |  |  |  |  |  |  |
| Tiruneh, 2018(133) | Ethiopia | UBA | Family planning | Provider training + increasing resources |  |  |  |  |  | X |  | X |  |  |  |  |
| Vail, 2018(134) | India | UBA | Neonatal care | Provider training |  |  |  |  | X |  |  |  |  |  |  |  |
| Vandenberg, 2018(135) | United States | CBA | Medication management | Provider Education/Training + Audit and Feedback |  |  |  |  |  | X |  | X |  |  |  |  |
| Witt, 2018(136) | United States | UBA | Medication management | Co-ordination/referral pathways |  |  |  |  | X |  |  |  |  |  |  |  |
| Wu, 2018(137) | United States | Prospective cohort | Hypertension | Implementing a new service |  |  |  |  |  | X |  |  |  |  |  |  |
| Yugbaré Belemsaga, 2018(138) | Burkina Faso | UBA | Family planning | Expanded scope of practice |  |  |  | X | X |  |  |  |  |  |  |  |
| Zhang, 2018(139) | China | RCT | Respiratory tract infections | Provider training + patient education + audit and feedback |  |  |  |  | X |  |  |  |  |  |  | X |
| Zunza, 2018(140) | South Africa | Retrospective cohort | Health system performance | Reorganization of services |  |  |  |  | X | X |  |  |  |  |  |  |
| Ameh, 2017(141) | South Africa | ITS | Non-communicable disease | Reorganization of services |  |  |  |  |  | X |  |  |  |  |  |  |
| Bailie, 2017(142) | Australia | UBA | Chronic disease | Audit and feedback |  |  |  |  | X |  |  |  |  |  |  |  |
| Baldwin, 2017(143) | United States | Cross-sectional | Chronic disease | Audit and feedback |  |  |  |  |  |  |  | X |  |  |  |  |
| Barnett, 2017(144) | United States | RCT | Weight management | Patient education |  |  |  |  |  | X |  |  |  |  |  |  |
| Basu, 2017(145) | United States | Microsimulation Model | Mental health | Reorganization of services |  |  |  |  |  |  |  |  |  |  |  | X |
| Bocoum, 2017(146) | Burkina Faso | Cohort | STI | Provider training |  |  |  |  | X |  |  |  |  |  |  |  |
| Brown, 2017(147) | United States | Cohort | Asthma | Provider training |  |  |  |  | X | X |  |  |  |  |  |  |
| Carey, 2017(148) | Australia | CBA | Palliative care | Reorganization of services |  |  |  |  |  |  |  |  |  |  | X | X |
| Cene, 2017(149) | United States | Prospective cohort | Hypertension | Patient education + Reorganization of services |  |  |  |  |  | X |  |  |  |  |  |  |
| Doyle, 2017(150) | United States | Cross-sectional | Chronic lung disease | Expanded scope of practice |  |  |  |  | X | X |  |  |  |  |  |  |
| Emery, 2017(151) | Australia | RCT | Cancer | Provider training + coordination/referral pathways |  |  |  |  |  | X |  |  |  |  |  |  |
| Feltner, 2017(152) | United States | CBA | Diabetes | Expanded scope of practice |  |  |  | X |  | X |  |  |  |  |  |  |
| Fisher, 2017(153) | United States | Cohort | Mental health | Provider training |  |  |  |  |  |  |  | X |  |  | X | X |
| Halladay, 2017(154) | United States | Prospective cohort | Hypertension | Patient education + provider training |  |  |  |  |  | X |  |  |  |  |  |  |
| Hansel, 2017(155) | United States | UBA | Mental health | Patient education |  |  |  |  |  | X |  |  |  |  |  |  |
| Hountz, 2017(156) | United States | CBA | Cancer | Provider training + audit and feedback |  |  |  |  | X |  |  |  |  |  |  |  |
| Hunt, 2017(157) | United Kingdom | UBA | Diabetes | Provider training |  |  |  |  |  | X |  |  |  |  |  |  |
| Inch, 2017(158) | United Kingdom | Case series | Medication management | Decision support |  |  |  |  |  |  |  | X |  |  |  |  |
| Iyer, 2017(159) | Rwanda | ITS | Family planning | Expanded scope of practice |  |  |  | X |  |  |  |  |  |  |  |  |
| Jordans, 2017(160) | Nepal | Prospective cohort | Care-seeking | Co-ordination/referral pathways |  |  |  | X |  |  |  |  |  |  |  |  |
| Kiemtore, 2017(161) | Burkina Faso | UBA | Post-abortion care | Provider training |  |  |  |  | X | X |  |  |  |  |  |  |
| Klein, 2017(162) | United States | Cross-sectional | Veteran's healthcare | Patient education + telehealth/virtual care |  |  |  |  |  |  |  | X |  |  |  |  |
| Liddy, 2017(163) | Canada | Cross-sectional | N/A | Coordination/referral pathways |  |  |  |  |  |  |  |  |  |  | X | X |
| Lifson, 2017(164) | Ethiopia | UBA | HIV | Expanded scope of practice |  |  |  | X |  | X |  |  |  |  |  |  |
| Malekpour, 2017(165) | United States | Retrospective cohort | Care transfers | Provider training |  |  |  |  |  | X |  |  |  |  |  |  |
| Matousek, 2017(166) | Haiti | CBA | Surgery | Provider training + patient education |  |  |  |  | X |  |  |  |  |  |  |  |
| Maulik, 2017(167) | India | UBA | Mental health | Expanded scope of practice |  |  | X | X | X | X |  |  |  |  |  |  |
| Moeckli, 2017(168) | United States | Retrospective cohort | HIV | Telehealth/virtual care |  |  |  |  |  | X |  |  |  |  |  |  |
| Mueller, 2017(169) | Swaziland | Prospective cohort | HIV | Reorganization of services + increasing resources |  |  |  |  |  | X |  |  |  |  |  |  |
| Naderimagham, 2017(170) | Iran | Retrospective cohort | Pediatric outcomes | Increasing resources |  |  |  |  |  | X |  |  |  |  |  |  |
| Nagykaldi, 2017(171) | United States | UBA | Preventative care | Co-ordination/referral pathways |  |  |  | X | X |  |  |  |  |  |  | X |
| Omotayo, 2017(172) | Kenya | Cluster RCT | Medication management | Reorganization of services |  |  |  |  |  | X |  |  |  |  |  |  |
| Parsons, 2017(173) | United States | Cross-sectional | Sleep | Provider training |  |  |  |  | X |  |  |  |  |  |  |  |
| Paul, 2017(174) | Australia | Cluster RCT | Diabetes | Provider training |  |  |  |  | X |  |  |  |  |  |  |  |
| Paz-Pacheco, 2017(175) | Philippines | CBA | Diabetes | Training of lay-persons |  |  |  |  | X |  |  |  |  |  |  |  |
| Peterson, 2017(176) | United States | UBA | Integrated care | Expanded scope of practice |  |  |  |  | X | X |  |  |  |  |  |  |
| Rebello, 2017(177) | United States | CBA | Medication management | Reorganization of services + decision support |  |  |  | X |  | X |  |  |  |  | X | X |
| Sam-Agudu, 2017(178) | Nigeria | Prospective cohort | HIV | Training of lay-persons + implementing a new service |  |  |  |  |  | X |  |  |  |  |  |  |
| Sartor, 2017(179) | Argentina | UBA | Chagas disease | Provider training |  |  |  |  |  | X |  |  |  |  | X |  |
| Shetty, 2017(180) | India | Prospective cohort | Cardiovascular disease | Expanded scope of practice |  |  |  |  |  | X |  |  |  |  |  |  |
| Tewari, 2017(181) | India | UBA | Mental health | Decision support |  |  |  | X |  | X |  |  |  |  |  |  |
| Van de Mortel, 2017(182) | Australia | CBA | Palliative care | Telehealth/virtual care |  |  |  |  |  | X |  |  |  |  | X |  |
| Vellakkal, 2017(183) | India | CBA | Family planning | Reorganization of services |  |  |  |  | X |  |  |  |  |  |  |  |
| Wei, 2017(184) | China | Cluster RCT | Upper respiratory tract infection | Provider training |  |  |  |  |  |  |  |  | X |  |  |  |
| Wells, 2017(185) | New Zealand | Cluster RCT | Cardiovascular disease | Co-ordination/referral pathways |  |  |  |  | X |  |  |  |  |  |  |  |
| Whealin, 2017(186) | United States | Prospective cohort | Mental health | Patient education |  |  |  |  | X |  |  |  |  |  |  |  |
| Yan, 2017(187) | Zambia | RCT | Hypertension | Provider training + increasing resources |  |  |  |  |  | X |  | X |  |  |  |  |
| Zarrabian, 2017(188) | Canada | Prospective cohort | Surgery | Coordination/referral pathways |  |  |  | X |  |  |  |  |  | X | X |  |
| Zhan, 2017(189) | China | RCT | N/A | Provider training |  |  |  |  | X |  |  |  |  |  |  |  |
| Bottorff, 2016(190) | Canada | UBA | Smoking cessation | Provider training + patient education |  |  |  |  |  | X |  |  |  |  |  |  |
| Boyle, 2016(191) | Australia | Cross-sectional | Polycystic ovarian syndrome | Reorganization of services |  |  |  |  |  | X |  |  |  |  |  |  |
| Castellani, 2016(192) | Burkina Faso, Nigeria and Uganda | UBA | Febrile Illness | Training of lay-persons |  |  |  |  | X |  |  |  |  |  |  |  |
| Chen, 2016(193) | China | Cluster RCT | Vaccinations | Co-ordination/referral pathways |  |  |  | X |  |  |  |  |  |  |  | X |
| Crossland, 2016(194) | Australia | CBA | Diabetes | Expanded scope of practice |  |  |  |  |  | X |  |  |  |  |  |  |
| Das, 2016(195) | India | RCT | Any condition | Training of lay-persons |  |  |  |  | X |  |  |  |  |  |  |  |
| Dumphy, 2016(196) | United States | CBA | Breastfeeding | Provider training |  |  |  |  | X |  |  |  |  |  |  |  |
| Flum, 2016(197) | Germany | Cross-sectional | Physician access | Medical education- exposure |  |  |  |  |  | X |  |  |  |  |  |  |
| Frail, 2016(198) | United States | Retrospective cohort | Medication management | Expanded scope of practice | X |  |  |  |  |  |  |  |  |  |  |  |
| Hontelez, 2016(199) | South Africa | Retrospective cohort | HIV | Reorganization of services |  |  | X |  |  | X |  | X |  |  |  |  |
| Iwuji, 2016(200) | South Africa | Cluster RCT | HIV | Reorganization of services |  |  |  |  |  |  |  |  |  |  | X |  |
| Kompala, 2016(201) | South Africa | Retrospective cohort | HIV | Patient education |  |  |  |  | X |  |  |  |  |  |  |  |
| Lin, 2016(202) | Australia | UBA | Back pain | Provider training |  |  |  |  | X |  |  |  |  |  |  |  |
| Mark, 2016(203) | Canada | UBA | Diabetes | Patient education |  |  |  |  | X |  |  |  |  |  |  |  |
| Mehl-Madrona, 2016(204) | United States | CBA | Pain management | Patient education |  |  |  |  |  | X |  |  |  |  |  |  |
| Miao, 2016(205) | China | RCT | Hypertension | Patient education + reorganization of services |  |  |  |  |  | X |  |  |  |  |  |  |
| Mitchell, 2016(206) | Australia | UBA | Palliative care | Expanded scope of practice |  |  |  |  |  | X |  |  |  |  |  |  |
| Patel, 2016(207) | Ghana | UBA | Family planning | Transportation |  |  | X |  |  | X |  |  |  |  |  |  |
| Rodriguez Villa, 2016(208) | Spain | Retrospective cohort | Diabetes | Expanded scope of practice |  |  |  | X | X | X |  |  |  |  |  |  |
| Shakya, 2016(209) | Nepal | UBA | Cancer | Patient education |  |  |  | X | X | X |  |  |  |  |  |  |
| Shiferaw, 2016(210) | Ethiopia | Cross-sectional | Family planning | Co-ordination/referral pathways |  |  |  |  | X |  |  |  |  |  |  |  |
| Siribie, 2016(211) | Burkina Faso, Nigeria, and Uganda | Cross-sectional | Malaria | Training of lay-persons |  |  |  |  | X |  |  |  |  |  |  |  |
| Smyth, 2016(212) | Ireland | Prospective cohort | Atrial fibrillation | Screening |  |  |  | X | X |  |  |  |  |  |  |  |
| Taylor, 2016(213) | United States | Retrospective cohort | Physician access | Medical education- exposure |  |  |  |  | X | X |  |  |  |  |  |  |
| Tokuda, 2016(214) | United States | UBA | Diabetes | Patient education | X |  |  |  |  |  |  |  |  |  |  |  |
| Tomiak, 2016(215) | Poland | RCT | Cardiovascular disease | Patient education |  |  |  |  |  | X |  |  |  |  |  |  |
| Wendling, 2016(216) | United States | Retrospective cohort | Physician access | Medical education- recruitment |  |  |  |  |  | X |  |  |  |  |  |  |
| Wu, 2016(217) | China | CBA | Chronic disease | Patient education + increasing resources | X |  |  |  |  |  |  |  |  |  |  |  |
| Bodicoat, 2015(218) | Spain | UBA | Diabetes | Provider training + audit and feedback |  |  |  | X |  | X |  |  |  |  |  |  |
| Kirby, 2015(219) | Australia | UBA | Diabetes | Expanded scope of practice |  |  |  |  |  | X |  |  |  |  |  |  |
| Kozlowski, 2015(220) | United States | UBA | Mental health | Patient education |  |  |  |  |  | X |  | X |  |  |  |  |
| Lou, 2015(221) | China | RCT | COPD | Provider training |  |  |  |  |  | X |  | X |  |  |  |  |
| McClellan, 2015(222) | United States | Prospective cohort | Cancer | Screening |  |  |  |  | X | X |  |  |  |  |  |  |
| McDermott, 2015(223) | Australia | Cluster RCT | Diabetes | Provider training + coordination/referral pathways |  |  |  |  | X |  |  |  |  |  |  |  |
| Parra-Medina, 2015(224) | United States | RCT | Weight management | Patient education |  |  |  |  |  | X |  |  |  |  |  |  |
| Pyne (A), 2015(225) | United States | RCT | Mental health | Decision support + provider training |  |  |  |  |  | x |  |  |  |  |  | x |
| Pyne (B), 2015 (225) | United States | RCT | Mental health |  |  |  |  |  |  |  |  |  |  |  |  |  |
| Schnippel, 2015(226) | South Africa | Cross-sectional | Family planning | Reorganization of services |  |  |  |  |  | X |  |  |  |  |  |  |
| Tuntland, 2015(227) | Norway | RCT | Eldercare | Reorganization of services |  |  |  |  |  |  |  |  |  |  |  | X |
| Wibbenmeyer, 2015(228) | United States | Prospective cohort | Burn rehab | Decision support |  |  |  |  |  |  |  | X |  |  |  |  |
| Yun, 2015(229) | United States | UBA | Hypertension | Patient education |  |  |  |  |  | X |  |  |  |  |  |  |
| Baldwin, 2014(230) | United States | UBA | Health system performance | Reorganization of services |  |  |  |  |  |  |  |  |  |  | X | X |
| Barahimi, 2014(231) | Iran | Cohort | Kidney disease | Screening |  |  |  |  |  | X |  |  |  |  |  |  |
| Blattner, 2014(232) | New Zealand | UBA | Cardiovascular disease | Reorganization of services |  |  |  |  |  | X |  |  |  |  |  | X |
| Cicutto, 2014(233) | United States | UBA | Asthma | Provider training |  |  |  |  | X | X |  |  |  |  |  |  |
| Dignan, 2014(234) | United States | Cluster RCT | Cancer | Provider training |  |  |  |  | X | X |  |  |  |  |  |  |
| Josif, 2014(235) | Australia | UBA | Family planning | Reorganization of services |  |  |  |  |  |  |  | X |  |  |  |  |
| Köhler, 2014(236) | Germany | RCT | Dementia | Co-ordination/referral pathways |  |  |  |  |  | X |  |  |  |  |  |  |
| Nelson, 2014(237) | United States | Markov Modeling | Osteoporosis | Provider training |  |  |  |  |  | X |  |  |  |  |  | X |
| Nelson, 2014(238) | United States | Markov Modeling | Osteoarthritis | Provider training |  |  |  |  |  | X |  |  |  |  |  | X |
| Prasad, 2014(239) | Canada | UBA | Eldercare | Co-ordination/referral pathways |  |  |  | X | X |  |  |  |  |  |  |  |
| Wiechman, 2014(240) | United States | RCT | Burn rehab | Increasing resources + coordination/referral pathways |  |  |  |  |  | X |  | X |  |  |  |  |
| Yip, 2014(241) | China | Cluster RCT | Antibiotic prescribing | Reorganization of services |  |  |  |  | X |  |  | X |  |  |  |  |
| Zou, 2014(242) | China | UBA | Cardiovascular disease | Patient education |  |  |  |  | X | X |  |  |  |  |  |  |
| Branda, 2013(243) | United States | Cluster RCT | Diabetes | Decision support |  |  |  |  | X | X |  |  |  |  |  |  |
| Bray, 2013(244) | United States | CBA | Diabetes | Patient education + reorganization of services |  |  |  |  |  | X |  |  |  |  |  |  |
| Gardiner, 2013(245) | Australia | CBA | Physician access | Well-being |  | X |  |  |  |  |  |  |  |  |  |  |
| Greene, 2013(246) | Australia | UBA | Chronic disease | Financial incentive |  |  |  |  | X |  |  |  |  |  |  |  |
| Honeycutt, 2013(247) | United States | Cross-sectional | Cancer | Co-ordination/referral pathways |  |  |  |  | X | X |  |  |  |  |  |  |
| Jamieson, 2013(248) | Canada | Cohort | Physician access | Medical education- exposure | X | X |  |  |  |  |  |  |  |  |  |  |
| Krantz, 2013(249) | United States | UBA | Cardiovascular disease | Patient education + coordination/referral pathways |  |  |  |  |  | X |  |  |  |  |  |  |
| MacDowell, 2013(250) | United States | UBA | Physician access | Medical Education- recruitment | X | X |  |  |  |  |  |  |  |  |  |  |
| Petrany, 2013(251) | United States | CBA | Physician access | Medical education- exposure | X |  |  |  |  |  |  |  |  |  |  |  |
| Rabinowitz, 2013(252) | United States | Retrospective cohort | Physician access | Medical education- recruitment |  | X |  |  |  |  |  |  |  |  |  |  |
| Rashidian, 2013(253) | Iran | ITS | Health system performance | Reorganization of services |  |  |  |  |  |  |  |  |  |  | X |  |
| Walker, 2013(254) | Mexico | Cluster RCT | Obstetrics | Increasing resources |  |  |  |  | X |  |  |  |  |  |  |  |
| Chiu, 2012(255) | Taiwan | Cross-sectional | Minor illness | Expanded scope of practice |  |  | X |  |  |  |  | X |  |  |  |  |
| Clark, 2012(256) | United Kingdom | Prospective cohort | Hypertension | Reorganization of services |  |  |  |  |  | X |  |  |  |  |  |  |
| Hussain, 2012(257) | Australia | Cross-sectional | Health system performance | Reorganization of services |  |  |  |  |  |  |  |  |  |  | X |  |
| Kessler, 2012(258) | United States | Cross-sectional | Mental health | Co-ordination/referral pathways |  |  |  | X |  | X |  |  |  |  |  |  |
| Kluhsman, 2012(259) | United States | UBA | Cancer | Patient education |  |  |  |  | X |  |  |  |  |  |  |  |
| Lawson, 2012(260) | Canada | UBA | Chronic disease | Co-ordination/referral pathways |  |  |  |  |  | X |  |  |  |  |  |  |
| Mashari, 2012(261) | Canada | Cross-sectional | Pain management | Reorganization of services |  |  |  |  |  | X |  |  |  |  |  |  |
| Pathman, 2012(262) | United States | UBA | Physician access | Financial incentive | X |  |  |  |  |  |  |  |  |  |  |  |
| Robinson, 2012(263) | United Kingdom | Cross-sectional | Pain management | Expanded scope of practice |  |  |  |  |  | X |  |  |  |  |  |  |
| Albert, 2011(264) | United States | UBA | Medication management | Implementing a new service + training of lay-persons |  |  |  |  |  | X |  |  |  |  |  |  |
| Bender, 2011(265) | United States | UBA | Asthma | Provider training |  |  |  |  | X | X |  |  |  |  |  |  |
| Burgess, 2011(266) | Australia | ITS | Cardiovascular disease | Co-ordination/referral pathways |  |  |  |  | X | X |  |  |  |  |  |  |
| Farmer, 2011(267) | United Kingdom | Cohort | Health system performance | Expanded scope of practice |  |  | X | X |  | X |  | X |  |  |  |  |
| Nilsen, 2011(268) | Norway | CBA | Physician access | Medical education- exposure | X | X |  |  |  |  |  |  |  |  |  |  |
| Pape, 2011(269) | United States | Cluster RCT | Diabetes | Telehealth or virtual care |  |  |  |  |  | X |  | X |  |  |  |  |
| Quinn, 2011(270) | United States | Cohort | Physician access | Medical education- recruitment | X |  |  |  |  |  |  |  |  |  |  |  |
| Rabinowitz, 2011(271) | United States | Retrospective cohort | Physician access | Medical education- recruitment | X |  |  |  |  |  |  |  |  |  |  |  |
| Rabinowitz, 2011(272) | United States | Retrospective cohort | Physician access | Medical education- recruitment | X |  |  |  |  |  |  |  |  |  |  |  |
| Armstrong, 2010(273) | Australia | Cross-sectional | Mental health | Co-ordination/referral pathways |  |  |  |  | X | X |  |  |  |  |  |  |
| Burnham, 2010(274) | Canada | UBA | Pain management | Reorganization of services |  |  |  |  |  | X |  |  |  |  |  |  |
| Denewer, 2010(275) | Egypt | Cross-sectional | Cancer | Screening |  |  |  |  | X |  |  |  |  |  |  | X |
| Fiss, 2010(276) | Germany | Prospective cohort | Medication management | Expanded scope of practice |  |  |  |  |  | X |  |  |  |  |  |  |
| Gray, 2010(277) | Canada | RCT | Preventative care | Co-ordination/referral pathways |  |  |  |  | X | X |  |  |  |  |  | X |
| Larson, 2010(278) | Australia | UBA | Asthma | Patient education |  |  |  |  |  | X |  |  |  |  |  |  |
| Matsumoto, 2010(279) | Japan | Retrospective cohort | Physician access | Financial incentive |  | X |  |  |  |  |  |  |  |  |  |  |
| Noknoy, 2010(280) | Thailand | RCT | Alcohol use | Patient education |  |  |  |  |  | X |  |  |  |  |  |  |
| Orrantia, 2010(281) | Canada | Cross-sectional | Family planning | Reorganization of services |  |  |  |  |  |  |  | X |  |  |  |  |
| Perkins, 2010(282) | Australia | Prospective cohort | Mental health | Expanded scope of practice |  |  |  | X |  |  |  |  |  |  |  |  |
| Straume, 2010(283) | Norway | Retrospective cohort | Physician access | Medical education- exposure | X | X |  |  |  |  |  |  |  |  |  |  |
| Xin, 2010(284) | China | Cross-sectional | Diabetes | Implementing a new service |  |  |  |  |  | X |  |  |  |  |  |  |
| Andrews, 2009(285) | Australia | UBA | Skin infections | Training of lay-persons |  |  |  |  | X | X |  |  |  |  |  |  |
| Balcazar, 2009(286) | United States | UBA | Cardiovascular disease | Patient education |  |  |  |  |  | X |  |  |  |  |  |  |
| Deprez, 2009(287) | United States | UBA | COPD | Provider training |  |  |  |  | X | X |  |  |  |  |  |  |
| Ellerbeck, 2009(288) | United States | RCT | Smoking cessation | Reorganization of services |  |  |  |  |  | X |  |  |  |  |  |  |
| Hogg, 2009(289) | Canada | RCT | Chronic disease | Co-ordination/referral pathways |  |  |  |  | X | X |  |  |  |  |  |  |
| Kavanagh, 2009(290) | Australia | RCT | Alcohol use | Patient education |  |  |  |  |  | X |  |  |  |  |  |  |
| Kengne, 2009(291) | Cameroon | CBA | Diabetes | Expanded scope of practice |  |  |  |  | X | X |  |  |  |  |  |  |
| King, 2009(292) | United States | RCT | Diabetes | Provider training |  |  |  |  |  | X |  |  |  |  |  |  |
| Kirkbride, 2009(293) | United States | Cross-sectional | Diabetes | Reorganization of services |  |  |  |  | X | X |  |  |  |  |  |  |
| Murchie, 2009(294) | United Kingdom | Cross-sectional | Cancer | Co-ordination/referral pathways |  |  |  |  | X |  |  |  |  |  |  |  |
| Pastel, 2009(295) | United States | ITS | Diabetes | Expanded scope of practice |  |  |  | X |  | X |  |  |  |  |  |  |
| Probst, 2009(296) | United States | Cross-sectional | Health system performance | Financial incentive |  |  |  |  |  |  |  |  |  |  |  |  |
| Reynolds, 2009(297) | United States | CBA | Pain management | Patient education |  |  |  |  |  | X |  | X |  |  |  |  |
| Scrace, 2009(298) | Australia | UBA | Cancer | Transportation |  |  |  |  |  | X |  |  |  |  |  |  |
| Thomson, 2009(299) | New Zealand | UBA | Cancer | Patient education |  |  |  |  | X |  |  |  |  |  |  |  |
| Ding, 2008(300) | China | UBA | Epilepsy | Reorganization of services |  |  |  |  |  |  |  |  |  |  |  | X |
| Ely, 2008(301) | United States | RCT | Weight management | Patient education + decision support |  |  |  |  |  | X |  |  |  |  |  |  |
| Glasser, 2008(302) | United States | UBA | Physician access | Medical education- recruitment | X |  |  |  |  |  |  |  |  |  |  |  |
| Lane, 2008(303) | United States | Cluster RCT | Cancer | Provider training |  |  |  |  | X |  |  |  |  |  |  |  |
| Matsumoto, 2008(304) | Japan | Retrospective cohort | Physician access | Financial incentive |  | X |  |  |  |  |  |  |  |  |  |  |
| Pereira, 2008(305) | Canada | UBA | Palliative care | Provider training |  |  |  |  | X |  |  |  |  |  |  |  |
| Pomerantz, 2008(306) | United States | UBA | Mental health | Coordination/referral + reorganization of services |  |  |  | X |  |  |  | X |  | X |  |  |
| Saini, 2008(307) | Australia | CBA | Asthma | Increasing resources |  |  |  |  |  | X |  |  |  |  |  |  |
| Sears, 2008(308) | United States | UBA | Health system performance | Expanded scope of practice |  |  | X |  |  |  |  |  |  |  |  |  |
| Tyler, 2008(309) | United States | Retrospective cohort | Weight management | Patient education |  |  |  |  |  | X |  |  |  |  |  |  |
| Aghajanian, 2007(310) | Iran | UBA | Population health | Reorganization of services |  |  |  |  |  | X |  |  |  |  |  |  |
| Beckham, 2007(311) | United States | RCT | Alcohol use | Patient education |  |  |  |  |  | X |  |  |  |  |  |  |
| Cullum, 2007(312) | United Kingdom | RCT | Mental health | Reorganization of services |  |  |  |  |  | X |  | X |  |  |  |  |
| Florence, 2007(313) | United States | CBA | Physician access | Medical education- recruitment | X |  |  |  |  |  |  |  |  |  |  |  |
| Hodgins, 2007(314) | Australia | UBA | Mental health | Provider training |  |  |  |  | X |  |  |  |  |  |  |  |
| Laatikainen, 2007(315) | Australia | UBA | Diabetes | Patient education |  |  |  |  |  | X |  |  |  |  |  |  |
| Liaw, 2007(316) | Australia | Cluster RCT | Asthma | Provider training |  |  |  |  | X |  |  |  |  |  |  |  |
| McCrae, 2007(317) | United States | RCT | Sleep | Provider training + patient education |  |  |  |  |  | X |  |  |  |  |  |  |
| Mitton, 2007(318) | Canada | UBA | Chronic disease | Co-ordination/referral pathways |  |  |  |  |  |  |  |  |  |  | X | X |
| Riou, 2007(319) | France | CBA | Medication management | Provider training |  |  |  |  |  |  |  |  |  |  |  | X |
| Sherman, 2007(320) | United States | UBA | Physical activity | Patient education |  |  |  |  |  | X |  |  |  |  |  |  |
| Sutor, 2007(321) | United States | CBA | Mental health | Co-ordination/referral pathways |  |  |  |  |  |  |  |  |  |  | X |  |
| Watkins, 2007(322) | United States | CBA | Integrated care | Increasing resources |  |  |  |  |  |  |  |  |  |  | X |  |
| Ackermann, 2006(323) | Australia | UBA | Diabetes | Reorganization of services |  |  |  |  |  | X |  |  |  |  |  |  |
| Adams, 2006(324) | United States | Cohort | Mental health | Provider training |  |  |  |  |  | X |  |  |  |  |  |  |
| Ahles, 2006(325) | United States | RCT | Pain management | Patient education |  |  |  |  |  | X |  |  |  |  |  |  |
| Belkora, 2006(326) | United States | Cross-sectional | Cancer | Increasing resources |  |  |  |  |  |  |  | X |  |  |  |  |
| Dunbabin, 2006(327) | Australia | Cross-sectional | Physician access | Financial incentive | X |  |  |  |  |  |  |  |  |  |  |  |
| Gardiner, 2006(328) | Australia | CBA | Physician access | Well-being |  | X |  |  |  |  |  |  |  |  |  |  |
| Haase, 2006(329) | United States | UBA | Diabetes | Provider training + decision support + patient education |  |  |  |  |  | X |  |  |  |  |  |  |
| Kelaher, 2006(330) | Australia | Retrospective cohort | Medication management | Financial incentive |  |  |  | X |  |  |  |  |  |  |  |  |
| Kilkkinen, 2006(331) | Australia | UBA | Diabetes | Patient education |  |  |  |  | X | X |  |  |  |  |  |  |
| Meng, 2006(332) | China | UBA | HIV | Patient education + Reorganization of services |  |  |  |  |  | X |  |  |  |  |  |  |
| Oyama, 2006(333) | Japan | CBA | Mental health | Screening + patient education |  |  |  |  |  | X |  |  |  |  |  |  |
| Tobe, 2006(334) | Canada | RCT | Hypertension | Expanded scope of practice |  |  |  |  |  | X |  |  |  |  |  |  |
| Wang, 2006(335) | China | UBA | Epilepsy | Expanded scope of practice |  |  |  |  |  | X |  |  |  |  |  |  |
| Bergus, 2005(336) | United States | RCT | Mental health | Screening |  |  |  |  |  | X |  |  |  |  |  |  |
| Bray, 2005(337) | United States | UBA | Diabetes | Patient education + reorganization of Services |  |  |  |  |  | X |  |  |  |  |  | X |
| Bray, 2005(338) | United States | CBA | Diabetes | Expanded scope of practice |  |  | X |  |  | X |  |  |  |  |  |  |
| Campbell, 2005(339) | Australia | Prospective cohort | Mental health | Increasing resources |  |  |  |  |  | X |  |  |  |  |  |  |
| Dettori, 2005(340) | United States | UBA | Diabetes | Co-ordination/referral pathways |  |  |  |  | X |  |  |  |  |  |  |  |
| Farmer, 2005(341) | United States | UBA | Integrated care | Co-ordination/referral pathways |  |  |  | X |  | X |  | X |  |  |  |  |
| Hughes, 2005(342) | Australia | Cross-sectional | Cancer | Screening |  |  |  |  |  | X |  |  |  |  |  |  |
| Johnson, 2005(343) | United States | UBA | Diabetes | Co-ordination/referral pathways |  |  |  |  | X | X |  |  |  |  |  |  |
| Pacheco, 2005(344) | United States | Cross-sectional | Physician access | Medical education- exposure | X | X |  |  |  |  |  |  |  |  |  |  |
| Rabinowitz, 2005(345) | United States | Retrospective cohort | Physician access | Medical Education- recruitment |  | X |  |  |  |  |  |  |  |  |  |  |
| Reymond, 2005(346) | Australia | UBA | Palliative care | Provider training |  |  |  |  | X |  |  |  |  |  |  |  |
| Siminerio, 2005(347) | United States | UBA | Diabetes | Provider training + patient education |  |  |  |  | X | X |  |  |  |  |  |  |
| Vivilaki, 2005(348) | Greece | UBA | Cancer | Patient education |  |  |  |  | X |  |  |  |  |  |  |  |
| Askim, 2004(349) | Norway | RCT | Stroke rehab | Increasing resources |  |  |  |  |  | X |  |  |  |  |  |  |
| Bowden, 2004(350) | United States | UBA | Preventative care | Patient education |  |  |  |  |  | X |  |  |  |  |  |  |
| Cowan, 2004(351) | Canada | CBA | Asthma | Patient education |  |  |  |  | X |  |  |  |  |  | X |  |
| Edwards, 2004(352) | United Kingdom | Cluster RCT | Continuing education | Provider training |  |  |  |  | X |  |  |  |  |  |  |  |
| Graffen, 2004(353) | Australia | RCT | Medication management | Reorganization of services |  |  |  |  |  | X |  |  |  |  |  |  |
| Lee, 2004(354) | Korea | Cross-sectional | Health system performance | Reorganization of services |  |  |  |  |  |  |  |  |  |  |  | X |
| Lin, 2004(355) | Taiwan | CBA | Hypertension | Patient education + training of lay-persons |  |  |  |  |  | X |  |  |  |  |  |  |
| Maddigan, 2004(356) | Canada | Cluster RCT | Diabetes | Coordination/referral pathways + provider training |  |  |  |  |  | X |  |  |  |  |  |  |
| Santos, 2004(357) | United Kingdom | UBA | Cardiovascular disease | Provider training |  |  |  |  |  | X |  |  |  |  |  |  |
| Elley, 2003(358) | New Zealand | Cluster RCT | Physical activity | Provider training + patient education |  |  |  |  |  | X |  |  |  |  |  |  |
| Goldhaber-Fiebert, 2003(359) | Costa Rica | RCT | Diabetes | Patient education |  |  |  |  |  | X |  |  |  |  |  |  |
| Goldstein, 2003(360) | Canada | Cross-sectional | Family planning | Provider training |  |  |  | X |  |  |  |  |  |  |  |  |
| Jackson, 2003(361) | United States | CBA | Physician access | Financial incentive | X | X |  |  |  |  |  |  |  |  |  |  |
| Judd, 2003^129^ | Australia | Cross-sectional | Mental health | Provider training + coordination/referral |  |  |  |  | X |  |  |  |  |  |  |  |
| Kit, 2003(362) | Australia | UBA | Diabetes | Patient education |  |  |  |  |  | X |  |  |  |  |  |  |
| Majumdar, 2003(363) | Canada | Cluster RCT | Diabetes | Patient education |  |  |  |  | X | X |  |  |  |  |  |  |
| Naccarella, 2003(364) | Australia | Cross-sectional | Therapeutic relationship | Provider training |  |  |  |  | X |  |  | X |  |  |  |  |
| Simmons, 2003­(365) | Australia | UBA | Diabetes | Reorganization of services |  |  |  |  |  | X |  |  |  |  |  |  |
| Taylor, 2003(366) | United States | RCT | Medication management | Expanded scope of practice |  |  |  | X | X | X |  |  |  |  |  |  |
| Arthur, 2002(367) | United Kingdom | RCT | Vaccinations | Patient education + increasing resources |  |  |  |  |  | X |  |  |  |  |  |  |
| Elliott, 2002(368) | United States | Cluster RCT | Cancer | Provider training |  |  |  |  | X |  |  |  |  |  |  |  |
| James, 2002(369) | Australia | CBA | Preventative care | Patient education |  |  |  |  |  | X |  |  |  |  |  |  |
| Maislos, 2002(370) | Israel | UBA | Diabetes | Patient education |  |  |  |  |  | X |  |  |  |  |  |  |
| Malcolm, 2002(371) | Australia | UBA | Mental health | Expanded scope of practice |  |  | X |  |  | X |  |  |  |  |  |  |
| Walker, 2002(372) | Australia | ITS | Medication management | Audit and feedback |  |  |  |  | X |  |  |  |  |  |  |  |
| Ahles, 2001(373) | United States | RCT | Pain management | Patient education |  |  |  |  |  | X |  |  |  |  |  |  |
| Cave, 2001(374) | Canada | UBA | Asthma | Patient education |  |  |  |  | X | X |  |  |  |  |  |  |
| Kempe, 2001(375) | United States | Cross-sectional | Vaccinations | Reorganization of services |  |  |  |  |  | X |  |  |  |  |  |  |
| Mayer-Davis, 2001(376) | United States | RCT | Diabetes | Patient education + increasing resources |  |  |  |  |  | X |  |  |  |  |  |  |
| McDermott, 2001(377) | Australia | Cluster RCT | Diabetes | Reorganization of services |  |  |  |  |  | X |  |  |  |  |  |  |
| O'Grady, 2001(378) | New Zealand | UBA | Diabetes | Reorganization of services |  |  |  |  | X | X |  |  |  |  |  |  |
| Rabinowitz, 2001(379) | United States | Retrospective cohort | Physician access | Medical education- recruitment | X | X |  |  |  |  |  |  |  |  |  |  |
| van Amburgh, 2001(380) | United States | UBA | Vaccinations | Patient education |  |  |  |  |  | X |  |  |  |  |  |  |
| Weinehall, 2001(381) | Sweden | CBA | Cardiovascular disease | Patient education |  |  |  |  |  | X |  |  |  |  |  |  |
| Anderko, 2000(382) | United States | UBA | Health system performance | Expanded scope of practice |  |  | X |  |  |  |  | X |  |  |  |  |
| Hippisley-Cox, 2000(383) | United Kingdom | CBA | Cardiovascular disease | Coordination/referral pathways |  |  |  |  |  | X |  |  |  |  |  | X |
| Shum, 2000(384) | United Kingdom | RCT | Minor illness | Expanded scope of practice |  |  | X |  |  | X |  | X |  |  |  |  |
| Smith, 2000(385) | United States | RCT | Mental health | Provider training |  |  |  |  | X | X |  |  |  |  |  |  |
| Thomas, 2000(386) | United States | UBA | STI | Training of lay-persons |  |  |  |  | X |  |  |  |  |  |  |  |
| Johansson, 1999(387) | Sweden | RCT | Cancer | Expanded scope of practice |  |  |  | X |  | X |  |  |  |  |  |  |
| Kinsinger, 1998(388) | United States | Cluster RCT | Cancer | Audit and feedback |  |  |  |  | X | X |  |  |  |  |  |  |
| Carter, 1997(389) | United States | CBA | Hypertension | Expanded scope of practice + provider training |  |  |  |  | X | X |  | X |  |  |  |  |
| Keyserling, 1997(390) | United States | Cluster RCT | Hypertension | Patient education + coordination/referral |  |  |  |  |  | X |  |  |  |  |  |  |

*UBA: uncontrolled before and after; CBA: controlled before and after; RCT: randomized controlled trial; ITS: interrupted time series.
