# Supplementary material for: Interventions to improve primary healthcare in rural settings: A scoping review
Source: PLoS One. 2024 Jul 11;19(7):e0305516. doi: 10.1371/journal.pone.0305516 (PMC11239038; doi:10.1371/journal.pone.0305516)
Supplement: S3 Appendix — (DOCX) [file pone.0305516.s004.docx]

**Quality: Evidence-based practice**

| **Author, Year, Country** | **Design** | **Aim** | **Brief Intervention description** | **Outcome measurement** |
| --- | --- | --- | --- | --- |
| Antibiotic Prescribing | | | | |
| Yip, 2014, China | Cluster RCT | To evaluate the effects of capitation with pay-for-performance on primary care providers’ antibiotic prescribing practices, health spending, outpatient visit volume, and patient satisfaction. | Code: Reorganization of Services  It involved a policy intervention that changed from a fee-for-service to a capitated budget with pay-for-performance to cover outpatient services. | The primary outcome measures were the proportion of patient visits that included one or more prescriptions for antibiotics, total health care expenditure per visit, drug expenditure per visit, the number of patient consultations per day in a facility, and patient satisfaction. |
| Any Condition | | | | |
| Gu, 2020, China | Uncontrolled before/after | To evaluate the effectiveness of a continuing medical education program to improve the knowledge and skills of rural doctors in China. | Code: Healthcare Provider Training  The Spring Seedling Project-Zhaotong program was a continuing medical education program for rural physicians. It consisted of three stages: remote education, workshops conducted in Zhaotong and field training in Shanghai. | The program's effectiveness was assessed based on differences between pre- and post-training knowledge and skill test scores. |
| Chinhoyi, 2018, South Africa | Cluster RCT | To assess the impact of family physician (FP) supply on indicators of district health system performance, clinical processes and clinical outcomes in the Western Cape Province. | Code: Reorganization of Services  The intervention involved an analysis of data from the Western Cape Department of Health’s routinely collected database. Data extracted included: Persal (human resources database), Sinjani (database of aggregated performance data of hospitals), ETR.net (electronic tuberculosis [TB] registry) and the National Population Census 2011. Data were linked using district and subdistrict names. | Data from the Western Cape Department of Health’s routinely collected database was used to measure outcomes. Primary health care utilisation was used to assess health system performance. Cervical smears, TB treatment, couple-year protection rate, early antenatal booking and under 1-year immunisation coverage were used as clinical processes indicators. Maternal, perinatal, and under-5 mortality were used to assess clinical outcomes. Dependent variables were calculated using the Western Cape Department of Health’s Annual Performance Plan 2016–2017 definitions and formulas. |
| Das, 2016, India | RCT | To evaluate the impact of a multitopic training program on diagnostic and treatment quality for informal providers. | Code: Training of Lay Community Members  Providers across the district were invited to participate. The training consisted of 72 sessions and 150 teaching hours over nine months. It included various topics, emphasising basic medical conditions, triage, and avoiding harmful practices. | The primary outcomes were condition-specific metrics obtained from the standardized patient interactions: potential improvements in necessary care through condition-specific checklists of recommended care and rates of correct case management. |
| Edwards, 2004, United Kingdom | Cluster RCT | To evaluate the effects of training general practitioners (GPs) in shared-decision making (SDM) and using simple risk communication aids in general practice on patient-based outcomes. | Code: Healthcare Provider Training  The intervention involved training general practitioners in shared decision-making and using simple risk communication aids. The training workshops with simulated patients for doctors focused on acquiring skills in shared decision-making or using risk communication materials. Participants were randomized by cluster (i.e., by doctor). Within each cluster, patients were also allocated randomly to consult with the doctor at one of the three time points in the study (baseline, first intervention phase or combined intervention phase). Additionally, randomization allocated patients to attend either a ‘research clinic’ or a usual surgery time. | The patient-based outcomes measured cognitive, affective and health outcomes Measures included: (i) COMRADE instrument (the principal measure) risk communication subscale patient’s confidence in the decision subscale (ii) Anxiety (short form of Spielberger) (iii) Enablement (iv) Health status (SF-12) mental subscale physical subscale (v) Satisfaction with the decision made (single item) (vi) Intention to adhere to chosen treatment (single item) (vii) Patient’s perceived support in decision (single item). All outcomes were assessed after the study consultation and one month later. |
| Any Condition (Elderly) | | | | |
| Prasad, 2014, Canada | Uncontrolled before/after | To describe the implementation of the Care for Seniors model of care and to provide evidence of the effective use of specialist resources and acute care services. | Code: Coordination/Referral Pathways  The Care for Seniors model improves care coordination and integration for older adults with complex medical conditions. The coordinator of care in this model is the NP-Geri. | This study measured the number of new geriatrician referrals and follow-up visits before and after the launch of the Care for Seniors program, the number of Nurse Practitioner visits in a primary care setting, in-home, retirement home and hospital, and the number of hospital discharges and hospital stay. |
| Any Condition (Veterans) | | | | |
| Howe, 2018, United States | Uncontrolled before/after | To strengthen team-based solutions in recognizing issues faced by older veterans. | Code: Healthcare Provider Training  The Rural Interdisciplinary Team Training Program (RITT) is a workforce training program to enhance the geriatrics knowledge and skills of VA primary care clinicians and staff caring for older veterans in rural communities. It involves completing a 6.5-hour workshop. | They evaluated team development with a web-based survey administered before, three, and six months after the workshop. Additionally, evaluated change in geriatric knowledge from immediately before to immediately after the workshop with a geriatrics knowledge test. Content analysis was used to evaluate the quality of action plans. In addition, participant satisfaction with the program was assessed. |
| Any Condition (Pediatrics) | | | | |
| Naccarella, 2003, Australia | Cross-sectional | The Adolescent Health Project (AHP) was a rural pilot project to strengthen the relationship between general practitioners (GPs) and adolescents. | Code: Healthcare Provider Training  The intervention included: a health professional education seminar program providing education to GPs, GP clinic staff, school counsellors and other service providers. Other aspects included: students visiting GP clinics, a school program where GPs visited schools and a parent education program. The intervention also provided resource development (ex., posters, manuals, and protocols). | Feedback forms were developed to assess GPs’, school counsellors’ and students’ beliefs, perceptions, and experiences of the AHP strategies. Two semi-structured interviews were conducted To assess the implementation of the AHP strategies, with the GP Project Manager, Inter-Divisional Coordinator and the three Divisional Project Officers; a sample of six GPs (i.e. six out of 16) and three GP clinic staff (i.e. three out of six) who had participated in the project; and all three School Counsellors who had participated in the project. Questions were posed about involvement in the AHP, perceptions about strategies and the AHP management and support, the transferability of the AHP model, outcomes experienced, future commitments and possible project developments. |
| Asthma | | | | |
| Brown, 2017, United States | Cohort | To assess the feasibility of incorporating telepharmacy services within a community pharmacy to deliver asthma education services in a rural community. | Code: Healthcare Provider Training  They developed an asthma education program based on the National Asthma Education and Prevention Program guidelines and Thomas Plaut’s book One Minute Asthma. All patients received the One Minute Asthma book and three educational sessions. Patient education was delivered through real-time audio/video counselling using telepharmacy equipment. The program consisted of three monthly education visits, with follow-up visits every three months over one year. The first educational visit reviewed various asthma management topics and developed an asthma action plan. | Asthma Control Test (ACT) and FEV1% were measured at each time frame (visits at months 1, 6, 9, and 12). One-sample t-tests assessed the mean difference in patient outcomes at a given visit against established clinical benchmarks. |
| Cicutto, 2014, United States | Uncontrolled before/after | The evaluation of a primary care, asthma-focused, evidence-based program provided to a 6-county, rural-frontier region in Colorado. | Code: Healthcare Provider Training  The program included an asthma champion workshop, asthma workshops, in-clinic coaching visits, clinician support tools, patient asthma education materials and teaching aids, a resource website and practice report cards. | Adherence to guidelines measured by six practice indicators: (1) use of spirometry to confirm asthma diagnosis and to assess asthma severity, control, and responsiveness; (2) assessment of asthma control; (3) recommended pharmacotherapy; (4) review and coaching for inhaler technique; (5) provision of written asthma action plans; and (6) advanced scheduling of a follow-up asthma appointment. |
| Bender, 2011, United States | Uncontrolled before/after | To determine whether the Colorado Asthma Toolkit increases adherence to asthma management guidelines. | Code: Healthcare Provider Training  The Colorado Asthma Toolkit Program is designed to: (1) increase the capacity of primary care practices to assess and manage asthma and (2) help clinicians to educate and support patients to increase medication adherence and effective self-management. Staff received training in asthma management consistent with evidence-based guidelines and in the use and interpretation of spirometry. Clinicians had 3 x 4-hour coaching visits and had access to a patient toolkit for self-management. | Adherence to asthma management guidelines was measured by assessing: (1) inhaled corticosteroid (ICS) prescriptions, (2) asthma action plans, and (3) spirometry between 1-3 months after completing all coaching visits. |
| Liaw, 2007, Australia | Cluster RCT | To determine the effectiveness of practice guidelines and education about paediatric asthma management delivered to general practitioners (GPs) in small group interactive workshops. | Code: Healthcare Provider Training  The intervention consisted of a GP paediatric asthma education program delivered over 2 x 3-hour workshops. GPs were also provided laminated paediatric guidelines adapted to their local context by an interdivisional group of GPs and investigators. | The primary outcome was GPs' self-reported use of Written Asthma Action Plans (WAAP). Secondary outcomes included (1) GPs knowledge of paediatric asthma management and (2) confidence to manage paediatric asthma. |
| Cowan, 2004, Canada | Controlled before/after | To determine the impact of a regional asthma education centre on reducing asthma-related morbidity and improving the quality of prescribing. | Code: Patient Education/Navigation  An accredited asthma teaching centre was implemented in a community health centre to educate patients with severe, moderate and mild asthma. The centre was staffed by two nurses and supervised by a physician. The first educational session usually lasted from 60 min to 90 min. Repeat sessions last 30 to 45 min and are scheduled based on perceived needs by the patient and nurse educator. Topics covered include mechanisms of asthma, triggers and inducers, goals of asthma treatment, medication use, side effects, and inhaler technique. | The number of emergency department (ED) visits for respiratory-related illness and prescribing anti-asthmatic medications were monitored during consecutive 18-month pre- and postintervention periods in two communities with similar health care resources. The quality of prescribing was assessed by calculating the ratio of inhaled corticosteroids to inhaled, short-acting beta2-agonists. |
| Cave, 2001, Canada | Uncontrolled before/after | To assess the effect of nurse-run clinics on patients' symptoms, pulmonary function, and healthcare utilisation over four months. | Code: Patient Education/Navigation  A nurse-run asthma clinic that provided advice on medication use, education on inhaler technique and basic asthma information according to the patient needs and the National Asthma and Respiratory Training Centre guidelines. Follow-up was arranged for reinforcement of teaching and question answering at one and four weeks. Reassessment took place at 16 weeks. | Measured the patient’s symptoms (e.g., number of days off work, night awakenings, pulmonary function (e.g., spirometry test, bronchodilator) via assessments by the nurse and self-report. |
| Atrial Fibrillation | | | | |
| Orchard, 2020, Australia | Cross-sectional | To improve the proportion of patients screened and treated for atrial fibrillation (AF) using the refined eHealth tools and to inform strategies on AF screening implementation in the rural setting. In addition, this study provides the first cost-effectiveness analysis in Australian general practice. | Code: Decision Support + Telehealth or Virtual Care  General practitioners/nurses at practices in rural Australia screened eligible patients (≥65 years of age without AF) using a smartphone ECG during practice visits. eHealth tools included electronic prompts, guideline-based electronic decision support, and regular data reports. | Primary outcomes were the proportion of screened patients with confirmed new AF, the proportion of AF and screened patients where the electronic decision support was accessed, the proportion of patients with AF who were prescribed an OAC according to guidelines, baseline AF prevalence in patients ≥65 years of age compared with metropolitan and nonrandomized control groups, new screen-detected AF incidence at the end of the study period in patients ≥65 years of age compared with the metropolitan and nonrandomized control groups, rates of OAC and antiplatelet treatment at baseline and completion for patients in the OAC recommended category compared with the metropolitan and nonrandomized control groups. The economic model developed in the  SEARCH-AF (Screening Education and Recognition in Community Pharmacies of Atrial Fibrillation) pharmacy screening study was adapted to evaluate the cost-effectiveness of the iECG screening in general practice. |
| Smyth, 2016, Ireland | Prospective Cohort | To test the feasibility of opportunistic screening in rural Ireland for AF. | Code: Screening  Opportunistic screening for AF. Local training was provided in each area, and each participating practice had a resource pack including information sheets, educational material and a treatment algorithm for AF. | Over six months, practices were requested to screen consecutive patients aged 65 and older, using digital palpation of the radial artery. For those diagnosed with new AF, choice of antithrombotic therapy and reasons for not choosing to anticoagulated were collected. |
| Back Pain | | | | |
| Lin, 2016, Australia | Uncontrolled before/after | To evaluate low back pain care changes following a systematic, theory-informed intervention in a rural Australian Aboriginal Health Service. | Code: Healthcare Provider Training  Involved an intervention to improve LBP management based on the TDF, including: (i) 2 x 3-hour interactive educational workshops on the management of LBP accredited by the Royal Australian College of General Practitioners, (ii) A retrospective audit and feedback of their LBP practice, (iii) provision of two clinical tools: a LBP clinical decision support tool and the Start Back tool. | Clinical audit of three target behaviours: (i) imaging referral behaviour, (ii) psychosocial assessment, and (iii) encouragement of self-management. |
| Cancer | | | | |
| Hountz, 2017, United States | Controlled before/after | To use a quality improvement approach to increase CRC screening rates in a rural FQHC nurse-managed health clinic. | Code: Healthcare Provider Training + Audit and Feedback  Quality improvement initiatives to increase CRC screening included educational staff meetings, ongoing communication with staff, weekly chart audits, and individual feedback through individua l flags in the HER. | Biweekly chart audits, retrospective chart reviews and comparison and analysis of pre and post-data assessed CRC screening rates. Assessments of the success of mailed reminders to patients were also analyzed. |
| McClellan, 2015, United States | Prospective Cohort | Colorectal cancer screening program to increase access to affordable colonoscopies for underinsured or uninsured residents of target counties while providing colonoscopy training to family medicine resident physicians.  Report quality indicators from colonoscopy procedures performed by family medicine physicians as part of a colorectal cancer prevention program targeting uninsured, low-income individuals. | Code: Screening  Report quality indicators from colonoscopy procedures performed by family medicine physicians as part of a colorectal cancer prevention program targeting uninsured, low-income individuals. | The number of colonoscopies performed during a 3-year period. Quality indicator results in comparison to ASGE recommendations. |
| Dignan, 2014, United States | Cluster RCT | To determine whether academic detailing to reach rural primary care providers with a CRC screening intervention was associated with increased CRC screening. | Code: Healthcare Provider Training  Academic detailing covering four modules: CRC screening efficacy, clinical performance measures, patient counselling, and creating a screening-friendly practice environment (including tools to identify patients who need screening). Individuals who knew the local community well and were familiar with primary care practices were selected to deliver the intervention. | Medical record review of physician recommendations for patients to obtain screening and documentation of fecal occult blood test results, flexible sigmoidoscopy, double contrast barium enema, and colonoscopy. |
| Honeycutt, 2013, United States | Cross-sectional | To evaluate the effectiveness of a patient navigation program among normal-risk patients for CRC. | Code: Coordination/Referral Pathways  Implement the CRC screening component of the Community Cancer Screening Program (CCSP), which involves health navigators who: 1) conduct chart audits to identify patients due for screening, 2) manage provider reminder systems, 3) coordinate screening and follow-up services, 4) provide one-on-one patient education and appointment reminders, 5) assist patients in overcoming barriers to screening (e.g., costs, transportation, literacy), 6) ensure that the colonoscopy recall schedule, based on gastroenterologist specialist recommendation, is entered into patient charts, and 6) coordinate provider feedback on screening referral patterns. | Outcomes were colonoscopy referral, examination, and CRC screening guideline compliance during the study period. |
| Kluhsman, 2012, United States | Uncontrolled before/after | Assess the acceptability of a take-home fecal immunochemical test and the effect of follow-up telephone counselling for increasing CRC screening in rural Appalachia. | Code: Patient Education/Navigation  A take-home fecal immunochemical test (FIT) with the American Cancer Society brochure ‘They know how to prevent colon cancer — and you can, too’ and a follow-up client-centred telephone counselling session. | Primary outcomes were i) acceptance of a provider-recommended, take-home FIT, as measured by enrollment and initial FIT adherence rates, and ii) FIT adherence rates after telephone counselling. |
| Denewer, 2010, Egypt | Cross-sectional | To evaluate the disease pattern and the screen-detected breast cancer rate and determine the effectiveness of clinical breast assessment-based screening (clinical examination and history taking with selective diagnostic breast imaging). | Code: Screening  Clinical breast assessment-based screening. Stage 1: a detailed questionnaire to identify at-risk women. Stage 2: Depending on the woman's age, CBE and mammography with/without ultrasonography. | Cost-effectiveness of screening program and detection rate using history taking and selective imaging. |
| Murchie, 2009, United Kingdom | Cross-sectional | To explore how GP-led melanoma follow-up had worked from the GPs' perspective. | Code: Coordination/Referral Pathways  GPs provided regular, scheduled and protocol-based follow-up examinations in GP surgeries, underpinned by a rapid-referral pathway to secondary care. | Qualitative interviews were conducted with GPs delivering GP-led melanoma follow-up. |
| Thomson, 2009, New Zealand | Uncontrolled before/after | To describe strategies to increase breast-screening participation in a rural general practice with a Maori population. | Code: Patient Education/Navigation  Multifaceted intervention targeting breast cancer screening that included local community involvement (sharing of past stories, promotion of screening, provision of screening information at various events), breast cancer screening education, and new enrolment opportunities such as group-based enrolment. | The computerised practice management system obtained screening coverage and outcomes data. |
| Lane, 2008, United States | Cluster RCT | To determine if provider interventions within health centre practices could improve the delivery and utilization of CRC screening. | Code: Healthcare Provider Training  A 1-hour accredited education session for clinicians on health behaviours and barriers to colorectal cancer (CRC) screening, how to facilitate risk communication, and shared decision-making. Individual health centre action plans to improve CRC screening rates were developed from a 1-hour SWAT analysis. | The primary outcome measure (coded yes or no) was deﬁned as referral/dispensing and completion of any CRC screening examination postintervention in a patient due for screening. Determined from an audit of patient records one year prior and post-intervention. |
| Vivilaki, 2005, Greece | Uncontrolled before/after | To determine whether a patient health education meeting can improve cervical screening rates. | Code: Patient Education/Navigation  Theoretically informed health education with women to promote cervical screening. Two midwives led the discussion; women were given a brochure and invited to organise a group visit for screening within the next 15 days to be screened together as a team. | Rates of cervical screening were used to assess the intervention’s effectiveness. |
| Elliott, 2002, United States | Cluster RCT | To evaluate educational and systems strategies to improve rural primary-care physicians’ cancer practice behaviours. | Code: Healthcare Provider Training  An 8-component educational intervention for physicians, nurses, and pharmacists practising in rural communities: 1) clinical opinion leaders; 2) annual mini fellowships for the clinical opinion leaders; 3) bimonthly cancer conferences in each experimental community; 4) quarterly project newsletters; 5) a rapid-cycle quality improvement system based on the feedback of practice data; 6) clinical practice guidelines (CPGs); 7) a telecommunication system linking experimental communities with the regional cancer centre; and 8) annual rural advisory committee meetings. | Thirty-seven clinical endpoints were constructed to measure and evaluate the adherence of primary-care physicians to clinical practice guidelines for each cancer type. |
| Kinsinger, 1998, United States | Cluster RCT | To evaluate whether a practice facilitation intervention with GPs will result in better breast screening rates. | Code: Audit and Feedback  A practice facilitation intervention that included (i) identifying a breast cancer screening performance gap through audit and feedback, (ii) revision of practice activities to improve screening, (iii) provision of materials for tracking/prompting, tailored for each practice and (iv) educational program on breast cancer screening. | Breast cancer screening performance (primarily mammography and CBE) was collected through cross-sectional reviews of randomly chosen medical records. |
| Chronic Disease | | | | |
| Bailie, 2017, Australia | Uncontrolled before/after | To examine the impact of state/territory policy support on the uptake of evidence-based continuous quality improvement activities and quality of care for Indigenous Australians. | Code: Audit and Feedback  The intervention involved implementing the Audit and Best Practice in Chronic Disease (ABCD) programme. The programme is designed to enable services to assess their practice against evidence-based guidelines, and an audit protocol accompanies each tool in the programme to assess performance. | They used the Quality of Care Index to measure adherence to evidence-based clinical best practice guidelines. |
| Greene, 2013, Australia | Uncontrolled before/after | This study examines the impact of Australia’s pay-for-performance (P4P) program for general practitioners (GPs). | Code: Financial Incentive  The Australian government initiated a ﬁnancial incentive program for “improved management of diseases such as asthma and diabetes and increased screening for cervical cancer” for GPs, who are paid for each patient visit on a fee-for-service basis. | Tracked trends in the provision of four incentivized services before P4P program implementation (1995–2000) and afterwards (2001–2010). They used descriptive statistics to examine whether there was an increase in the annual number of incentivized diabetes-related tests and cervical cancer screens after program implementation. Additionally, they measured GP participation in the program. They tracked the extent to which changes in a GP’s P4P program participation were related to increases in diabetes testing (HbA1c and microalbumin) or cervical cancer screening (diagnostic and treatment). |
| Hogg, 2009, Canada | RCT | To examine whether the quality of care (QOC) improves when nurse practitioners and pharmacists work with family physicians in community practice and focus on patients 50 and older who are considered at risk of experiencing adverse health outcomes. | Code: Co-ordination/referral pathways  Patients were randomly assigned to receive usual care from their family physicians or Anticipatory and Preventive Team Care (APTCare). Teams comprised the patient’s physicians, 1 of 3 nurse practitioners, and a pharmacist. Care included comprehensive chart reviews, home visits, medication management reviews, and individualized care plans that identified active health issues and management goals for the patient. | The primary outcomes of interest were quality of care for chronic disease management (CDM; adherence to guidelines) for diabetes, coronary artery disease, congestive heart failure, and chronic obstructive pulmonary disease via chart review and end-of-study QOC measures. Questionnaires assessed QOL, daily living evaluations, emergency department visits and hospitalization. Additionally, clinical outcomes of hemoglobin A1C and blood pressure were measured at the study's start and completion. |
| Chronic Lung Disease | | | | |
| Doyle, 2017, United States | Cross-sectional | To report on the implementation and clinical outcomes of a community-based pulmonary rehabilitation program in rural Appalachia. | Code: Extending Scope of Practice - Non-FP  Three rural health centres and a large referral hospital worked together to establish pulmonary rehabilitation services based on AACVPR guidelines. The guideline's recommendations included staffing by a registered respiratory therapist, 12 weeks and 24 sessions of education and exercise, coverage of 8 core educational topics, assessment and attention to psychosocial and nutritional issues, encouragement of continued exercise (maintenance) after program completion, and ongoing program evaluation. | To measure clinical outcomes, a retrospective medical record study compared pre- and post-program values for the modified Medical Research Council dyspnea level, 6-minute walk test (6MWT), opposing inspiratory force (NIF), respiratory disease knowledge, St George Respiratory Questionnaire (SGRQ), BODE index (body mass index, airflow obstruction, dyspnea and exercise capacity), and smoking status—the percentages of persons completing the program and participating in maintenance exercises after the program was recorded. |
| COPD | | | | |
| Lou, 2015, China | RCT | To evaluate an established a 4-year community-based health management intervention on improving the health status of patients with COPD. | Code: Healthcare Provider Training  The intervention involved two days of training for general practitioners in health management. The training included: general information about COPD, pathogenesis, risk factors, clinical manifestations, assessment of subjects’ conditions, display of exacerbation, and the stable stage of treatment and rehabilitation of COPD. In addition, they learned the follow-up process, how to help subjects quit smoking, and how to help subjects improve self-management skills. General practitioners in this study were trained according to GOLD guidelines and health management content. | The primary outcomes included BODE (body mass index) [B], degree of air-flow obstruction [O], level of functional dyspnea [D], and exercise capacity [E]). Secondary outcome measures were changes in COPD knowledge, awareness, and risk factors, measured with a survey on epidemiology, prevention, treatment, and rehabilitation. Cumulative COPD deaths for all causes within 96 months were entered through our link to the China Information System for Disease Control and Prevention. Changes in anxiety and depression symptoms were measured with the Hospital Anxiety and Depression Scale (HADS). Changes in hospital admissions, emergency department visits, and changes in medication regimens were obtained by subject interview, healthcare databases, or prospectively from diary cards, and frequency was recorded. |
| Deprez, 2009, United States | Uncontrolled before/after | To describe implementing evidence-based changes for COPD care in primary care practices. | Code: Healthcare Provider Training  A COPD project was initiated at 18 primary care clinical practices in rural areas of Maine to improve the diagnosis and treatment of patients with COPD. Clinical guidelines based on the Global Initiative for Chronic Obstructive Lung Disease (GOLD) were implemented by the practices using the Institute for Healthcare Improvement Breakthrough Series learning session mode. During these sessions, participants were taught by faculty, experts familiar with the standards of care for COPD, and other community resources. | Baseline data to assess current COPD practice were collected for each practice before the initial learning session. Focus groups were used to identify and address practice and patient barriers at the learning sessions and through direct contact with the practices. Clinical practice and patient care changes were measured pre and post-initiative to evaluate the improvements. |
| CVD | | | | |
| Patel, 2019, Indonesia | Controlled before/after | To evaluate whether a mobile technology–supported primary health care intervention would improve the use of preventive drug treatment among people in rural Indonesia with a high risk of CVD. | Code: Decision support + Screening + Coordination/Referral Pathways + Telehealth or Virtual Care  A multifaceted mobile technology–supported intervention facilitating community-based, guideline-based CVD risk screening with referral, tailored clinical decision support for drug prescription, and patient follow-up. | The primary outcome was the proportion of individuals taking appropriate preventive CVD medications, defined as at least 1 BP-lowering drug and a statin for all high-risk individuals, and an antiplatelet drug for those with prior diagnosed CVD. Secondary outcomes included mean change in BP from baseline. |
| Wells, 2017, New Zealand | Cluster RCT | To assess the effect of the Point of Care device on the frequency of completed CVD risk assessments and whether this technology would impact ethnic disparities in screening. Additionally, to assess the effect of the point of care (POC) device for testing lipids and HbA1c in addition to testing by community laboratory facilities (usual practice) on the completion of cardiovascular disease (CVD) risk assessments in general practice. | Code: Coordination/Referral Pathways  The intervention aggregated data on CVD risk assessments using a web-based decision support programme common to each practice. | The primary outcome was the proportion of completed CVD risk assessments. Secondary outcome measures included: incomplete CVD risk assessment and time (in days) to complete CVD risk assessment. Additionally, differences in completion rate by practice location (urban/rural), practice size (large/small), age group, gender, Māori/nonMāori, and deprivation (NZDep) quintile were measured. The NZDep Index Score is a measure assigned to a patient's area of residence based on nine variables from the Census reflecting eight dimensions of relative deprivation of census tracts aggregated into quintiles (1 to 5, from least to most deprived). |
| Zou, 2014, China | Cluster RCT | To evaluate the preliminary effects of a systematic CVD risk reduction package on improving blood pressure, uptake of CVD-preventive medicine and lifestyle modifications in one township hospital in Zhejiang, China. | Code: Patient Education/Navigation  The intervention included implementing a systematic CVD risk reduction package in one township hospital. This package was a systematic guideline that included recommendations for CVD-preventive drugs, lifestyle modifications and adherence support for individuals with a high risk of CVD. | Primary outcome measures are blood pressure (measured through the mean change of blood pressure), uptake of CVD-preventive medicine (measured by calculating the proportion of individuals using CVD-preventive medicine) and lifestyle modifications (self-reported). |
| Burgess, 2011, Australia | Interrupted Time Series | To determine if holistic CVD risk assessment results in better identification of elevated CVD risk, improved delivery of preventive care for CVD and improvements in the CVD risk profile for Aboriginal adults in a remote community. | Code: Coordination/Referral Pathways  The intervention consisted of a team of healthcare professionals conducting “adult health checks” at homelands, township residences, workplaces and public spaces. The “adult health checks included (i) identification of patients with elevated CVD risk, (ii) chronic disease care and (iii) follow-up of patients for chronic disease monitoring and further care planning. | Outcomes were assessed via self-reported health indicators (ex., Weight or smoking habits) and auditing medical records (delivery of preventative care, medication, CVD events, and iatrogenic events) in six-month intervals for three years. They assessed improvements in delivering preventive care services, prescription of CVD-related medications, and estimated relative reduction in CVD risk. |
| Dental Care | | | | |
| Dahlberg, 2019, United States | Uncontrolled before/after | To determine if implementing fluoride varnish (FV) application to young children by providers in a rural primary care office was feasible. | Code: Expanding Scope of Practice – FP  Primary care providers at the clinical site participated in an educational session that included information on fluoride varnish application. Clinics were provided with a resource notebook containing national guidelines on FV application, recent journal publications regarding FV in primary care, and third-party reimbursement information. The nursing staff was provided with FV patient education handouts and stickers to give to pediatric patients who received FV. | The number of FV applications, time spent on procedure, perceived barriers, and overall cost. |
| Diabetes | | | | |
| Chen, 2021, China | RCT | To assess the long-term impact of an educational intervention on patients’ diabetes knowledge and fasting blood glucose (FBG) level and whether these outcomes differed between two rural counties. | Code: Patient Education/Navigation + Healthcare Provider Training  The intervention had three goals: 1) to strengthen collaboration between hospital care and PHC, 2) to improve patients’ diabetes knowledge and improved fasting blood glucose (FBG) level, and 3) to improve the knowledge and management of diabetes among healthcare professionals in primary care. The intervention for patients included education lectures, follow-up services, and special medical services, while healthcare professionals in PHC received professional skills training, team discussions, and regular meetings. | The primary outcomes were fasting blood glucose levels and diabetes knowledge scores measured via medical records and questionnaires. Measurements were taken at baseline in 2015 and two follow-ups in 2016 and 2017, respectively. |
| Chen, 2020, China | RCT | To assess the impact of an educational intervention on the knowledge, attitudes, and practice regarding Type 2 Diabetes Mellitus of primary health care professionals and the types of services, they could provide. | Code: Healthcare Provider Training  The intervention for health care professionals consisted of team communication improvement, regular meetings, and professional skills training sessions. Patients received health education lectures, periodical follow-up interviews with an annual physical examination, and special medical services (including helping patients with medical treatment, transfer treatment, return visits, and clinical care). | The primary outcomes were diabetes knowledge scores, practice scores, and attitudes scores measured by a questionnaire at the one-year follow-up. |
| Prudencio, 2020, United States | Uncontrolled before/after | To assess the impact that a clinical pharmacist-led comprehensive medication management (CMM) service has on outcomes for patients with diabetes | Code: Implementing a New Service + Patient Education/Navigation  Patients were referred to pharmacists for the medication management service. During the appointments, a comprehensive medication reconciliation was completed at the start of each visit to clarify any medication discrepancies or non-adherence. The remainder of the appointment provides motivational interviewing, medication and lifestyle counselling, and clarifying any patient questions. After making the necessary adjustments and providing education to the patient, the patient is then scheduled for a future follow-up appointment with the pharmacist or the PCP based on the discretion of the pharmacist. | The primary outcome of this study is the change in the goal attainment rates of the three clinical goals of hemoglobin A1c, blood pressure, and appropriate statin therapy after pharmacist intervention. |
| Chen, 2019, China | RCT | To assess the impact of an education-based intervention to improve  vertical integration and management of type 2 diabetes mellitus in primary care in rural China. | Code: Patient Education + Provider Education/Training  The intervention had three goals: 1) to strengthen collaboration between hospital care and PHC, 2) to improve patients’ diabetes knowledge and improved fasting blood glucose (FBG) level, and 3) to improve the knowledge and management of diabetes among healthcare professionals in primary care. The intervention for patients  included education lectures, follow-up services, and special medical services, while healthcare professionals in PHC received professional skills training, team discussions, and regular meetings. | The primary outcomes were changes in fasting blood glucose (FBG) level and health-related quality of life (measured by EQ-5D-3L questionnaire) post-intervention (one-year follow-up). |
| Murphy Buschkoetter, 2019, United States | Uncontrolled before/after | To increase the number of comprehensive foot examinations for adults with type-2 diabetes mellitus in rural primary care. | Code: Decision Support + Healthcare Provider Training  All primary care providers and clinic nurses attended educational sessions on  screening guidelines, and a template for this protocol was created in their EMR. | The primary outcome was the percentage of comprehensive foot examinations completed pre and post-intervention. |
| Wei, 2019, China | Uncontrolled before/after | To evaluate the effect of web-based knowledge training programs on diabetes management among primary health care providers in rural China and to further compare the effects of the training effect between primary health care providers with different backgrounds. | Code: Healthcare Provider Training  Primary care providers received web-based training on diabetes management that included information on clinical features and complications, how to screen for complications among diabetes patients, the treatment and management of patients with diabetes, and health education for patients with diabetes. | The primary outcome was knowledge achievement of participants measured by a multiple choice test at baseline, at the end of two weeks of training and three months after training. |
| Bouchonville, 2018, United States | Cohort | To determine whether participation in a multidisciplinary telementorship healthcare delivery model improves primary care provider (PCP) and community health worker (CHW) confidence in managing patients with complex diabetes in medically underserved regions. | Code: Healthcare Provider Training  A multidisciplinary team of specialists was recruited from the University of New Mexico Health Sciences Center to participate in Endo ECHO weekly teleECHO sessions. Additionally, PCPs and community health workers (CHW) were recruited at ten federally qualified health centres (FQHCs) around New Mexico, a largely rural state with many underserved communities, to remotely engage with Endo ECHO specialists via the weekly teleECHO sessions. PCPs and CHWs initially attended Project ECHO for a 2-day training in information technology use, team-building, and clinical skills around diabetes technology, including insulin pump therapy. All subsequent mentorship was conducted via the weekly teleECHO sessions. | Surveys were administered two years after the launch of Endo ECHO and completed anonymously by participants. Surveys were only administered to the PCPs and CHWs CHW-specific surveys included self-efficacy in 11 measures of health coaching, patient education, and identification of psychosocial treatment barriers. PCP-specific surveys included self-efficacy in 14 measures of complex diabetes management, recognition of diabetic complications, identification and appropriate treatment of depression, and serving as local diabetes experts for other community PCPs. |
| Paul, 2017, Australia | Cluster RCT | Researchers aimed to use objective administrative data to examine the effectiveness of online diabetes continuing medical education (CME) and additional intervention strategies at the population level in the rural setting. | Code: Healthcare Provider Training  The intervention provided GPs with prerequisite knowledge for optimal primary care management of diabetes and opportunities to practice. The Online Active Learning Module (ALM) included various features and presentation types, including evidence-based Australian clinical guidelines, video demonstrations, case studies, knowledge-based quizzes, clinical audits, self-reflection activities and a moderated peer discussion forum. | Recruitment to the intervention online ALM was monitored during the intervention period. Module completion was also obtained electronically via website-generated notification of when a participant had completed the final survey/self-assessment items, which were part of the ALM. Online access to specialist advice was also assessed using a log of contacts received. |
| Paz-Pacheco, 2017, Philippines | Controlled before/after | This study aimed to assess the effectiveness of diabetes self-management education (DSME) in a rural agricultural town. | Code: Training of Lay Community Members  Peer educators (I.e., village leaders, a retired school principal, a village health worker, a village nutrition scholar, and housewives) attended a two-day workshop conducted by endocrinologists. After the peer educators were trained and asked to do a return demonstration, this strategy focused on training lay health advisors to function as ‘community catalysts’ to promote a healthy lifestyle among people with diabetes in their community. Participants in both groups were given oral advice on diet, exercise, foot care, and medication compliance on each follow-up visit. The participants in the intervention group additionally received DSME. Modules developed by the International Diabetes Federation Consultative Section on Diabetes Education were translated and modified according to the participant’s level of knowledge. There were eight modules in the DSME program: (1) overview of diabetes mellitus, (2) diabetes and exercise, (3) diabetes and diet, (4) pharmacologic treatment of diabetes, (5) insulin use, (6) acute complications of diabetes, (7) microvascular and macrovascular complications of diabetes, and (8) foot care. The teaching sessions were held in the village health centres. | Data taken at baseline were again determined after three and six months. The outcome measures in both groups were analyzed in terms of differences in (1) mean or median values, (2) change from baseline, and (3) the proportion of participants achieving the recommended anthropometric and biochemical measures for persons with diabetes. |
| Crossland, 2016, Australia | Controlled before/after | The study aimed to trial the impact of general practice-based DR screening across a range of geographic contexts, integrated into the practices’ Diabetes Annual Cycle of Care and compared this with conventional methods of Diabetic Retinopathy (DR) screening. The secondary aim was to investigate the efficacy of routine monitoring by GPs of mild to moderate DR levels with distant ophthalmic support, compared with usual methods of DR management. | Code: Extending Scope of Practice FP  The participating GPs in the intervention practices completed a four-hour online DR upskilling program through the University of Queensland Master of Medicine (General Practice) Program, followed by an accreditation assessment through RANZCO Queensland Faculty. Each intervention practice was partnered with a distant ophthalmologist for the duration of the study. Patients without DR were rescreened later, according to NHMRC Guidelines. Patients with mild-moderate DR, diagnosed by the screening GP, were reviewed with the practice partner ophthalmologist through regular teleconferences and e-contact. Ongoing management regarding a referral or later reassessment was agreed upon and recorded. | The primary outcome measures reported were: the percentage of patients with type 2 diabetes in the intervention practices who received timely and appropriate DR screening, compared to rates in the control practices, and the proportion of patients with identified mild-moderate level DR who attended for review appointments in the intervention practices, compared to rates in the control practices. |
| RodriguezVilla, 2016, Spain | Retrospective Cohort | The objective of this study is to analyze the results of said teleophthalmology program, assess the epidemiological characteristics of the included population, the diagnostic capability of primary care physicians to identify diabetic retinopathy (DR) and adequate coordination with ophthalmologists, as well as assessing the savings said program produces. | Code: Extending Scope of Practice - Non-FP  Primary care physicians were trained to interpret retinographs (DR classification as per the international DR severity scale) with four-hour training workshops. In addition, two nurses were trained to carry out non-mydriatic retinographs (NMR), one posterior pole and four peripheral, and assess intraocular pressure (IOP) with applanation tonometry and detect alterations in the Amsler grid. | Outcomes included clinical measures of age, type of DM, years of evolution, treatment received (only diet, oral antidiabetics [OAD], insulin or combination of both), glycosylated hemoglobin percentage (HbA1c) taken three months before or after the retinograph date, presence of other cardiovascular risk factors (arterial hypertension, dyslipidemia, diabetic nephropathy defined as microalbuminuria >30mg/ml/24h or microalbumin/creatinine ratio of >20mg/g), cardiovascular pathologies of the ischemic cardiopathy type, ischemic encephalopathy and peripheral ischemia such as diabetic foot or amputations due to distal necrosis. Additionally, all retinographs of the sample were assessed by two ophthalmologists for accuracy. |
| Branda, 2013, United States | Cluster RCT | To determine the effectiveness of decision aids to support antihyperglycemic medication and statins for diabetes management in nonacademic and rural primary care clinics. | Code: Decision Support  The intervention involved primary care practices and patients with type 2 diabetes and comparing decision aids to support antihyperglycemic medication and statins to “usual care” for diabetes management. | Decisional outcomes were assessed using a survey to evaluate their knowledge of the medications and their risk of a heart attack without the statin medication. Additionally, they measured decisional comfort, efficacy, and satisfaction with the Decisional Conflict Scale. A fidelity checklist was also used to review the video encounters of physicians to see whether they were to use the decisional aids as intended. Clinical outcomes included reviews of medical records for medication use and change in crucial laboratory parameters (lipid profile, Hb1A1c, LDL-cholesterol). Pharmacy records were used to estimate medication adherence. |
| Kengne, 2009, Cameroon | Controlled before/after | To evaluate the effect of a primary nurse-led care program for type 2 diabetes involving guideline-driven glucose and blood pressure control. | Scope: Extending Scope of Practice - Non-FP  State-registered nurses with no experience in managing chronic diseases were trained to be in charge of running the clinics. They received a 1-week intensive training with the demonstration at the beginning of the program and a refresher course a year later. Three nurses were directly in charge of patient care in each clinic. Medical doctors monitored their activities monthly. Physicians covered aspects of diabetes care from diagnosis to management and referral-provided training. The first clinic visit (30min or more) offered the opportunity for baseline assessment and educating a patient on risk factors. Blood pressure levels and weight were measured during subsequent visits, and fasting capillary glucose was assessed. | The primary outcome measures were trajectories of fasting capillary glucose and blood pressure indices and differences in the mean levels between baseline and final visits. |
| Kirkbride, 2009, United States | Cross-sectional | To examine the relationship between the presence of a rural health clinic in a rural primary care service area (PCSA) and the likelihood of receiving recommended diabetes-related services associated with high-quality primary care for adults categorically eligible (eligible through Temporary Assistance to Needy Families [TANF] or disabled status) Medicaid beneﬁciaries with diabetes. | Code: Reorganization of Services  Retrospectively assessed differences in the rates of recommended diabetes-related primary care services within a sample of adult categorically eligible Medicaid beneﬁciaries identiﬁed as residing in PCSAs designated as urban or rural with and without at least 1 RHC, after accounting for individual subject characteristics that may inﬂuence receipt of the measured services. The study was based upon all administrative claims, encounters, and enrollment data from Oregon’s Medicaid program, the Oregon Health Plan (OHP), for the study period of calendar years 2002-2003. | The relative quality of diabetes-related primary care was assessed based on the proportion of subjects receiving the following diabetic care services at least once during a study year: hemoglobin A1c (HbA1c), lipid proﬁle, and an eye exam. |
| Kilkkinens, 2006, Australia | Uncontrolled before/after | To evaluate the efficacy and feasibility of a primary care-based diabetes prevention model with modest resource requirements involving a combined dietary and physical activity intervention in rural Australia. | Code: Patient Education/Navigation  The intervention model consisted of six group counselling sessions (12 months) facilitated by trained study nurses, dieticians and physiotherapists. The group counselling sessions provided individuals with dietary and physical activity guidance based on Dietary Guidelines for Australian Adults and National Physical Activity Guidelines. The Health Action Process Approach (HAPA) model and self-regulation theory are used to set individual goals and motivate individuals to progress from intention to actual behaviour change. | To evaluate the program, clinical measurements including height, weight, waist, hip and blood pressure measurements and fasting blood samples were taken before the intervention started, at three months and one year. |
| Dettori, 2005, United States | Uncontrolled before/after | To describe effective changes in the delivery of preventive care, improved clinical outcomes, and reduced barriers to patient self-management for diabetes. | Code: Coordination/Referral Pathways  The Park County Diabetes Project and the Montana Diabetes Control Program included health systems interventions and coordinated diabetes education to improve the quality of diabetes care. Some of these changes included: establishing and maintaining the patient registries, nurses conducting mail and telephone outreach to patients, mailing personalized patient education materials regarding the ABCs of diabetes, and providing ongoing continuing education workshops for the Park County Diabetes healthcare team. Community-based activities included organizing six 1-day foot-care clinics for patients with diabetes, establishing a lending library for the community with patient education materials, and developing and disseminating a patient newsletter on diabetes management. | Clinical data from the diabetes registries in 2 primary care practices and baseline and follow-up telephone surveys were used to evaluate improvements in care, outcomes, education, and barriers to self-management. |
| Johnson, 2005, United States | Uncontrolled before/after | To describe a field-based diabetes care program for rural primary care patients and provide some evaluative data. | Code: Coordination/Referral Pathways  The intervention consisted of implementing a simple computerized registry called the Diabetes Quality Care Monitoring System (DQCMS) to support primary care clinicians in tracking key elements of care for their patient populations with diabetes. | Outcomes were assessed via medical records. Key indicators of diabetes care were recorded (hemoglobin A1c tests, blood pressure, low-density lipoprotein cholesterol, urinalyses, foot and dilated retinal exams, and pneumococcal vaccinations). |
| Siminerio, 2005, United States | Uncontrolled before/after | To determine the impact of implementing chronic care model elements on providers’ diabetes care practices and patient outcomes in a rural practice setting. | Code: Healthcare Provider Training + Patient Education/Navigation  The intervention was modelled on a chronic care model of diabetes self-management education (DSME). The provider intervention consisted of education and training on standards of care and guidelines and practice using problem-based learning case studies for adherence to guidelines and DSME. The patient intervention consisted of participation in the DSME program. If the patient agreed to participate, they would participate in a series of biweekly five two-hour group sessions that consisted of goal setting and behavioural change strategies for managing their diabetes. | The provider intervention was assessed via a qualitative survey that insight into what they perceived as patient barriers to care and a chart review to measure adherence to guideline practices. Patients were assessed on empowerment and knowledge via questionnaires and biological health indicators (Hemoglobin A1C, serum cholesterol, blood pressure). |
| Majumdar, 2003, Canada | Cluster RCT | T to assess the effectiveness of a multidisciplinary diabetes outreach service (intervention) for improving the quality of care for rural patients with type 2 diabetes. | Code: Patient Education/Navigation  Local providers delivered usual care in the control region by adding three bimonthly visits by the CDA Traveling Diabetes Resource Program (CDA-TDRP). The CDA-TDRP travels to communities in rural Alberta, raising diabetes awareness and emphasizing patient self-management. In addition to bimonthly CDA-TDRP visits, the intervention region was exposed to the diabetes outreach service. The service consisted of a team of specialist physicians, nurse educators, dieticians, and pharmacists. The service travelled to the largest communities in the region monthly for six months, delivering targeted educational messages. | The primary outcome measurement was an improvement in the care of patients with diabetes. Researchers deﬁned this as a 10% improvement over baseline in any of the following after six months: blood pressure, total cholesterol, or HbA1c. As additional quality indicators, researchers examined changes in target medications, i.e., medications for lowering blood pressure, cholesterol, and glucose levels. Trained study coordinators collected baseline data through interviews, physical assessments, laboratory testing, and self-report questionnaires. Subjects were followed-up six months after study entry when all the measurements above and questionnaires were repeated. |
| O'Grady, 2001, New Zealand | Uncontrolled before/after | To evaluate the Diabetes Project implemented to  self-care and improve diabetes management in a rural Northland, New Zealand community. | Code: Reorganization of Services  The Diabetes Project aimed to evolve a new method of care based on group meetings with family and community involvement. The intervention consisted of weekly meetings, outside the health centre, in homely and non-institutional surroundings. Consultations with a doctor, dietitian, nurse, podiatrist or retinal photographer were voluntary and optional, with staff operating in a supportive partnership. Consultations were conducted as part of the group (with permission) and privately. | An annual audit process of the South Auckland Diabetes Project was conducted. Outcomes that were assessed included regular assessment of weight, blood pressure and blood glucose, and foot care with periodic checking of lipids, renal function, eyes and a measure of long-term glucose control (initially fructosamine and then HbA1c as it became available). |
| Epilepsy | | | | |
| Giuliano, 2018, Bolivia | Uncontrolled before/after | To assess the baseline level of knowledge, attitudes, and practices (KAP) towards epilepsy among general practitioners (GPs) of the rural communities of the Chaco region and the change in the level of knowledge, attitudes, and practices post-training. | Code: Healthcare Provider Training  The intervention consisted of two training modules six months apart, each with a two-day duration on epilepsy care. | The primary outcome was knowledge, attitudes, and practices measured by a pre-and post-training questionnaire. |
| Health System Performance | | | | |
| Zhan, 2017, China | RCT | To evaluate the effects of a blended-learning approach for rural primary healthcare workers in improving their knowledge about basic public health services (BPHS) and training satisfaction compared with a pure e-learning approach. | Code: Healthcare Provider Training  Three-course modules were developed based on the BPHS contents: 1) health management of patients with hypertension, course module, 2) health records management for residents, and 3) vaccination. Each module had two components: theoretical learning and case studies. The intervention group received the modules online on a learning platform, and the case studies were delivered face-to-face. | Primary outcomes were score changes on the knowledge test and participant satisfaction via a questionnaire. |
| HIV | | | | |
| Yapa, 2020, South Africa | Cluster RCT | To establish the effects of continuous quality improvement (CQI) on the quality of antenatal HIV care in primary care clinics in rural South Africa. | Code: Healthcare Provider Training + Training of Lay Community Members  The intervention focused on developing the capacity of local antenatal care (ANC) health workers in study clinics and aimed to improve the implementation of the national eMTCT guidelines. The intervention was delivered by trained CQI mentors and included standard CQI tools(process maps, fishbone diagrams, run charts, Plan-Do-Study-Act [PDSA] cycles, and action learning sessions). CQI mentors worked with health workers, including nurses and HIV lay counsellors. The mentors flexibly used the standard CQI tools tailored to local clinic needs. Health workers were direct intervention recipients, whereas pregnant women attending ANC were the ultimate beneficiaries. | Quality of care in HIV-related ANC: (i) viral load monitoring among pregnant women living with HIV and (ii) repeat HIV testing among pregnant women not living with HIV. |
| Naidoo, 2018, South Africa | Cross-sectional | To measure fidelity to HIV programme implementation as provided by community health workers from the community’s perspective. | Code: Training of Lay Community Members  Community health workers worked under the supervision of a nurse to provide services of health education to prevent HIV infection, identifying individuals that need to test for HIV, referring HIV-infected individuals not yet in care to start antiretroviral therapy (ART), providing adherence support to those on ART, tracing and referral of HIV-infected individuals that have been lost to the ART programme, and identifying individuals who have clinically failed on ART and require further assessment. | Implementation fidelity was assessed by measuring the content, coverage, frequency, and duration of the provincial guidelines that are currently in place. Coverage, ‘reach’, as measured by the proportion of households that reported CHW visits ever. Frequency was assessed by determining the proportion of households that received CHW visits according to the required schedule (at least once a month in the case of vulnerable households). Duration of implementation refers to the need for ongoing service delivery, i.e., no major programme interruptions; defined duration as high in case of CHW visit < 6 months ago or low for CHW visit > 6 months ago. The content was measured by the proportion of individuals reporting HIV services delivered by CHWs that the researchers deemed aligned with the guidelines, including HIV health education and referrals. |
| Kompala, 2016, South Africa | Retrospective Cohort | This study aimed to determine the impact of community-based CD4 testing services within a community-based intensive case finding with voluntary counselling and testing (CBVCT) program that refers identified HIV-positive individuals to treatment programs in rural South Africa. | Code: Patient Education/Navigation  The intervention involved providing patients with HIV health education and rapid HIV testing with on-site follow-up. If a nurse was present, CD4 count phlebotomy was performed on-site to determine ART eligibility. Patients were referred to their local primary care clinic for CD4 testing if a nurse was absent. Staff notified community members via phone or physical notification and were referred to local primary health clinics to initiate ART according to South African National Guidelines. | Outcome measures included standardized screening questionnaires and CD4 test completion data collected from clinics and laboratories. Result notification was a successful telephone or in-person contact to relay the results. Information on ART initiation was collected as part of the program monitoring and evaluation. Linkage to care and data on ART initiation was gathered from patient self-report or clinics and depended on successful contact. |
| Hypertension | | | | |
| Zheng, 2019, China | Uncontrolled before/after | To evaluate the effectiveness of hypertension management intervention and analyse the factors associated with blood pressure reduction within China’s primary healthcare system. | Code: Healthcare Provider Training  All the participating GPs, nurses and public health doctors received training before the intervention. The training included hypertension screening, treatment and management, follow-up visits and health education. All patients in the intervention received one physical examination and at least four face-to-face follow-ups with GPs in the year. Further interventions were offered depending on the outcome of the follow-ups. | Blood pressure was the primary outcome variable measured by a mercury sphygmomanometer. |
| Carter, 1997, United States | Controlled before/after | To build on previous research evaluating pharmacy care for hypertension patients, evaluate the program in a rural clinic pharmacy. The impact on blood pressure control, quality of life, patient satisfaction, quality of care, and cost of care was evaluated. | Code: Extending Scope of Practice - Non-FP + Healthcare Provider Training  Involved in a program to train community pharmacists to provide hypertension monitoring and direct consultation to physicians and nurses delivered in a Veterans Affairs pharmacist-managed hypertension clinic in Chicago. They were given extensive reading materials concerning hypertension, including national guidelines, workshops and practice experience. | Outcome measures include surveys and blood pressure measurements using the American Heart Association Standards and Guidelines. Both study groups completed the Short Form 36 at baseline and six months. Patients also received a questionnaire at the end of the study to assess their overall satisfaction with care delivery and pharmacy services. |
| Malaria | | | | |
| Siribie, 2016, Burkina Faso, Nigeria, and Uganda | Cross-sectional | The present article reports on the training and performance of community health workers in applying HIV care recommendations. | Code: Training of Lay Community Members  The intervention involved choosing community members to act as community healthcare workers to aid in treating malaria. Community healthcare workers underwent extensive training that covered diagnosing malaria, treating malaria (including using indicators or tools like respiratory rates or thermometers), counselling caregivers posttreatment, documentation, follow-up visits, and referral advice for immediate transit. The training comprised practical sessions with theory, including demonstrations, role play and discussion sessions. | The training was evaluated via pre and post-tests using a standardized questionnaire and through observation of practice on real patients by facilitators of training sessions. |
| Maternal & Child Health | | | | |
| Larson, 2020, Tanzania | Cluster RCT | To examine the success of a maternal healthcare quality improvement intervention in improving quality. | Code: Healthcare Provider Training + Coordination/Referral Pathways + Increasing Staff Resources  The quality improvement intervention involved In-service training on basic emergency obstetric and newborn care, access to referral facilities, mentorship and supportive supervision by an obstetrician and infrastructure support in the form of necessary equipment, supplies and medication. | Knowledge of obstetric and newborn care (measured by a test and clinical vignettes), obstetric services provided (recorded by the registers at the facilities), quality of care (measured via self-report by patients and providers), satisfaction (self-report from patients), and maternal health consisting of lack of anemia (hemoglobin level is 12.0 g/dl or above for nonpregnant women and 11.0 g/dl or above for pregnant women), lack of hypertension(average systolic reading less than 140 mm Hg and average diastolic reading less than 90 mm Hg), distribution of EQ-5D (EuroQol Group, Rotterdam, Netherlands) and distribution of mid-upper arm circumference (all biomarkers were collected via a household survey). |
| Medication-Related | | | | |
| Khalil, 2018, Australia | Uncontrolled before/after | To describe the steps involved in successfully implementing a medication safety program in primary care in rural Australia, report on its evaluation, and provide recommendations for future initiatives. | Code: Healthcare Provider Training  The medication safety training consisted of lectures, case studies, and small group discussions over one day. Overall, the training addressed problem-solving for possible errors and how to prevent them as a strategy for medication errors, medication administration, high-alert medications, and medication risk reductions. | The evaluation consisted of a questionnaire that targeted the following general domains: 1. knowledge about medication safety principles and sources of medication errors within the workplace 2. Confidence about medication safety practices and what to do to avoid errors in the workplace. 3. Behaviour about the ability of the participants to implement the newly developed medication safety principles and guidelines in the workplace. 4. Satisfaction of the participants with the training and mentoring received during the training. The participants were invited to complete the questionnaire before and immediately after the training. |
| Witt, 2018, United States | Uncontrolled before/after | To implement process changes to improve adherence to chronic opioid therapy prescribing and monitoring guidelines within a primary care health system serving three small rural communities. | Code: Coordination/Referral Pathways  A quality improvement project on opioid prescribing for chronic non-cancer pain was implemented. This included: prescribing registries, a nurse coordinator, an Opioid Use Review Panel and following opioid prescribing guidelines. | The primary outcome of the study was the implementation of opioid prescribing guidelines. Additional outcomes included decreased prescribing of chronic opioid therapy for chronic non-cancer pain. Outcomes were measured using the prescribing registry. The average daily dose of chronic opioid therapy prescribed for all patients was measured during the 6-month pre-implementation period and again during the six months after implementation. The number of patients using high-dose chronic opioid therapy and intermediate and low doses was also measured. |
| Taylor, 2003, United States | RCT | To determine the effect of pharmacist-provided pharmaceutical care on adverse drug-related events. | Code: Extending Scope of Practice - Non-FP  Pharmacists provided pharmaceutical care to patients 2-3 times per week in addition to usual care. Pharmaceutical care included individualized patient education and review of medical records to determine drug compliance and complications. | Number of ED visits and hospitalizations obtained from review of medical records and patient interviews. Medication compliance was self-reported by patients, and the 36-Item Short-Form Health Survey was used to assess quality of life and mental health. Prescribing appropriateness was determined with the Medication Appropriateness Index. Clinical endpoints such as hypertension, diabetes, anticoagulation, and dyslipidemia were also reviewed. |
| Walker, 2002, Australia | Interrupted Time Series | To determine the impact of a prescribing initiative on the prescribing patterns of a group of general practices. | Code: Audit and Feedback  The initiative comprised input from a health authority pharmaceutical adviser, practice comparison feedback, peer review meetings, and prescribing recommendations. The pharmaceutical adviser worked for the group approximately one day a week for one year, obtaining electronic prescribing analyses and cost (ePACT) data for six therapeutic areas in partnership with the HA medical advisor (a GP member of the intervention group). The six areas were: gastrointestinal, cardiovascular, respiratory, central nervous system, infections, and musculoskeletal and analgesics. | Practice data were collated for overall prescribing and therapeutic categories between 1997/1998 and 1998/1999 and analysed statistically. Prescribing expenditure trends were also collated. Outcome measures were: deviation from practices’ prescribing budgets; changes in practices’ ePACT data (net ingredient cost/patient unit [nic/PU]; number of items/PU; percentage of generic items) between the two years April 1997–March 1998 and April 1998–March 1999, for overall prescribing and BNF chapters 1–6, 10 and ‘all others’; and changes in practice monthly prescribing trends over the two years. Figures were also obtained for the health authority average to ascertain to what extent the practices were representative. Two quality markers were used: BNF chapter 6, the endocrine system, with rising prescribing indicating good practice, and the overall number of items/PU. |
| Mental Health | | | | |
| Harrison, 2022, United States | Uncontrolled before/after | To evaluate the effectiveness of an early childhood tele-education program in preparing primary care pediatric clinicians to manage developmental and mental health disorders in young children. | Code: Healthcare Provider Training  Pediatric primary care clinicians participated in weekly tele-education videoconferences. The sessions include learning modules on neurodevelopmental, behavioural disorders, and adverse childhood experiences. | The primary outcomes were knowledge and confidence gain measured by the change in scores on knowledge tests and surveys pre and post-intervention. |
| Muyambi, 2021, Australia | Controlled before/after | To measure the effect of depression awareness and management training on the attitudes of rural primary health care workers. | Code: Healthcare Provider Training  A six-session training workshop was informed by the National Institute for Health and Care Excellence guidelines for treating and caring for people with depression. | The 22-item Revised Depression Attitude Questionnaire comprised the primary outcome measure. Participants were assessed 12 weeks before the training, again on the day of the start of the training and after the training. |
| Poudiougou, 2021, Mali | Uncontrolled before/after | To provide a mental health training intervention to rural general practitioners (GPs), to organize community awareness activities, and to evaluate the impact on mental health knowledge and through the number of new patients diagnosed with mental disorders and managed by these general practitioners. | Code: Healthcare Provider Training  Two face-to-face group training workshops followed by individual follow-up supervisions were conducted to train GPs on diagnosing and managing mental health conditions. Some GPs were also trained to train other healthcare workers. | Change in knowledge was measured through pre-and post-training questionnaires. Other outcomes, such as the number of patients diagnosed with mental disorders and managed by the newly trained GPs, were reported by each GP every month. |
| Bellesheim, 2020, United States | Cohort | To evaluate the utility of Maintenance of Certification Quality Improvement training designed to improve developmental (e.g. autism spectrum disorder) screening rates in underserved, rural primary care practices. | Code: Reorganization of Services  The intervention included training primary care providers using Extension for Community Healthcare Outcomes (ECHO) Autism, a virtual learning network. The network provided them access to a pediatrician specializing in development and behaviour, a child and adolescent psychiatrist, a clinical psychologist, a social worker, a dietician, and a parent of a child with an autism spectrum disorder. | The primary outcomes were screening rates. |
| Bowen, 2020, United States | Cohort | To compare the effects of implementing a collaborative care program on depression outcomes in rural Native American and Alaska Native (AI/AN) patients with White patients and patients of other ethnic backgrounds at three clinics. | Code: Healthcare Provider Training  The intervention was designed to integrate treatment for common mental health disorders (e.g. depression, anxiety) into primary care settings using principles of chronic disease management using an interdisciplinary team (primary care provider, a psychiatric consultant, and a behavioural health care manager). The intervention provided clinic staff with training on universal screening for depression, evidence-based treatment to target, and the use of behavioural health care.  " | Primary outcomes were depression response (i.e., reduction in symptom severity as measured by PHQ-9) and depression remission (measured by HEDIS) measured over two years. |
| Espinet, 2020, Canada | RCT | To test the effectiveness of the Practitioner Training in Child and Adolescent Psychiatry (PTCAP) program. | The intervention involved 8 hr of training for primary care providers in practice guidelines and brief therapeutic skills for depression, anxiety, attention deficit hyperactivity disorder, and behavioural disorders with case discussion and video examples. | Primary outcomes were the provider's confidence (via the Provider Confidence Scale, the Physician Confidence Scale, the Ease of Consultation Scale, & the Referral Comfort Scale), attitudes (Physician Belief Scale) and knowledge (the Practitioner Mental Health Knowledge) at one-week follow-up. |
| Maconick, 2018, South Africa | Prospective Cohort | To develop and evaluate a locally delivered, long-term, in-service training programme to facilitate mental health care in primary care. | Code: Extending Scope of Practice - Non-FP  The in-service training programme was delivered weekly 1-h sessions by local psychiatry staff to 20 primary care nurses at the clinic over five months. The training was based on the “Practical Approach to Care Kit” guidelines that teach primary care workers first-line treatments for depression, substance misuse, psychosis and dementia. | Data were collected before the training began and again at four months. A questionnaire was administered to all participants examining competence at diagnosing and treating common mental disorders and some core skills for mental health, such as mental state examination. Additionally, the number of referrals was collected for one month before training and at the 4-month evaluation mark after training was complete. Interviews were conducted to assess potential barriers to using the training at work daily. |
| Maulik, 2017, India | Uncontrolled before/after | To evaluate the feasibility and acceptability of an intervention for identifying and treating common mental health disorders. | Code: Extending Scope of Practice - Non-FP  A mental health services delivery model that leveraged technology and task sharing to facilitate identification and treatment (including following treatment guidelines) of common mental disorders (CMDs) such as stress, depression, anxiety and suicide risk in rural areas of Andhra Pradesh, India. The intervention was delivered by lay village health workers (Accredited Social Health Activists – ASHAs) and primary care doctors. An anti-stigma campaign using multi-media approaches was conducted across the villages at the project's outset. | The primary outcome was an evaluation of pre- and post-intervention mental health service utilization. Additionally, depression and anxiety scores of those who tested positive for a CMD at baseline were measured and compared post-intervention. Trained interviewers conducted a baseline survey. The survey enquired about sociodemographic details, stressors, social networks, CMD, history of mental disorders and their treatment, family history of mental disorders, and perceptions about stigma related to mental health. Process evaluation of the project was done using focus group discussions and in-depth interviews with key stakeholders. |
| Tewari, 2017, India | Uncontrolled before/after | The project evaluated an intervention to provide a mental health service intervention with preliminary evidence of effectiveness, feasibility, acceptability, and potential for scale-up. | Code: Decision Support  A mental health services delivery model that leveraged technology and task sharing to facilitate the identification and treatment of common mental disorders (CMDs) such as stress, depression, anxiety and suicide risk in rural areas of Andhra Pradesh, India. The intervention was delivered by lay village health workers (Accredited Social Health Activists – ASHAs) and primary care doctors. | Quantitative data to understand frequency and type of services used - type and appropriateness of care provided by doctors, frequency of follow-up of screen positive cases by ASHAs, and numbers and success rate of interactive voice response system calls. |
| Armstrong, 2010, Australia | Cross-sectional | To explore the views of women screened for postnatal depression at maternal and child health checks for many years in one rural shire. | Code: Coordination/Referral Pathways  The intervention included maternal and child health nurses screening all women for postnatal depression using the Edinburgh Postnatal Depression Scale. | They used a postal survey to ask if they received screening for postnatal depression, their experience, and if they had any referrals due to being screened. Additionally, women were invited to be interviewed about their experiences, including physical and emotional health during their first postnatal year, who conducted the screening, results of screening, whether they considered themselves to be depressed, any formal diagnoses, sources of help they used, and any suggestions to improve the program. |
| Hodgins, 2007, Australia | Uncontrolled before/after | To examine whether local ‘context-driven’ training would increase knowledge and reported change in practice by GPs with mental health patients. | Code: Healthcare Provider Training  Several workshops (6 hours total) were provided to GPs to improve their access to mental health education and training. They covered discussions on the use of screening and symptom severity measures, the use of patient self-monitoring forms, and patient educational material. | Questionnaires assessing attitudes, satisfaction, and practice were given before and after the workshops. To assess the impact of the workshops, comparisons to pre- and post-test responses to the following questions were made: (1) how often they saw patients with depression and anxiety; (2) treatment plans; (3) referrals to other mental healthcare professionals (4) the ease of getting advice on mental disorders; (5) their attitude regarding mental disorders about their competence, effectiveness, comfort, satisfaction and stress. |
| Judd, 2003, Australia | Cross-sectional | To evaluate the first 12 months' activities of a key component of a General Practice Psychiatry program – the GP practice visit. | Code: Healthcare Provider Education + Coordination/Referral Pathways  The Rural Depression Anxiety Research and Treatment (DARTR) GP program was part of a broader program designed to increase access to treatment for people with anxiety and depressive disorders who reside in rural areas. The program aimed to: 1. Enhance the capacity of GPs to identify anxiety and depressive disorders. 2. Enhance the capacity of GPs to use evidence-based treatments for depression and anxiety disorders. 3. Provide a referral source for GPs to assess or treat people with complex or severe anxiety or depressive disorders. Clients referred to the DART-R clinic by a GP also received assessments from a psychiatrist or a clinical psychologist. | Questionnaires were used to evaluate the effects on participating general practitioners’ practice. The questionnaire assessed: 1. Which practice activities the GP participated in? 2. The educational content of the visit. 3. How useful had the information gained during the visit been in managing 4. Whether the GP had made changes to their practice as a result of the practice visit.5. Whether the GP’s interaction with the Area Mental Health Service (AMHS) had changed due to the visit. 6. Whether the GP had participated in the associated DART-R GP program activities. 7. Which other DART-R GP activities the GP was interested in? Outcomes of the clinic that were assessed included: diagnostic clarification, management advice (formulation of a management plan, medication review, review of suitability for CBT or interpersonal therapy) or development of shared care with the DART-R clinician and the GP. |
| Smith, 2000, United States | RCT | To assess a guideline-based intervention's impact on depression care provided in rural vs. urban primary care settings. | Code: Healthcare Provider Training  The QUEST (Quality Enhancement by Strategic Teaming) intervention was to increase the proportion of depressed primary care patients who completed a guideline-concordant course of antidepressant pharmacotherapy or psychotherapy in the six months following the index visit at which they were identified as depressed. All participating physicians and nurses in enhanced care practices participated in a series of four academic, detailed telephone conference calls over two months to become systematically engaged with the AHCPR depression treatment guidelines. The training also included interactive roleplaying exercises and written tests to retain information. Additionally, the research team’s clinical social worker ensured nurse fidelity to the intervention by reviewing weekly patient treatment logs completed by the nurses and by completing a series of telephone calls with each nurse to provide feedback and support as they adapted to their new roles. | Data were collected via structured telephone interviews during the week following the index visit (baseline) and again six months following the index visit. Pharmacotherapy was measured via self-report from the patient on taking antidepressant medication at minimum therapeutic guideline-concordant daily doses for at least three months between baseline and six-month follow-up. |
| Neonatal Care | | | | |
| Vail, 2018, India | Uncontrolled before/after | Evaluates the impact of PRONTO International simulation training on the quality of neonatal resuscitation skills in simulated resuscitations and live deliveries in rural PHCs throughout Bihar, India. | Code: Healthcare Provider Training  PRONTO International training consists of in-situ simulations of various neonatal and obstetric emergencies, which include teamwork and communication activities, skills stations, and case-based learning supplements. Using a  train-the-trainer model, PRONTO provided six days of training for mentors on simulation facilitation, team building, communication skills, and debriefing skills before mentoring began and four-day refresher training three months into the mentoring period. | Video recordings were evaluated for the use of proper techniques. The following was evaluated: proper neck extension, positive pressure ventilation (PPV) with chest rise, and assessment of heart rate, stimulation, suction, proper PPV rate, or time to completion of key steps. |
| Obstetrics | | | | |
| Walker, 2013, Mexico | Cluster RCT | This study evaluates the relative strengths of adding an obstetric nurse or professional midwife to the physician-based team in rural clinics. | Code: Increasing Staff Resources  Undertook a cluster-randomised trial in 27 clinics in 2 states with high maternal mortality. Twelve non-physician providers (obstetric nurses (4) and professional midwives (8)) were randomly assigned to clinics; 15 clinics served as control sites. | Over 18 months in 2009–2010, we evaluated the quality of care through chart review and monthly interviews with providers about the last three deliveries performed. We analysed practices by creating indices using WHO care guidelines for normal labour and childbirth. The volume of care was assessed using administrative reporting forms. |
| Palliative Care | | | | |
| Pereira, 2008, Canada | Uncontrolled before/after | This study assessed the impact of the course on palliative care-related competencies for two classes: 2004 and 2005. | Code: Healthcare Provider Training  The University of Calgary offers a palliative care course involving classroom and web-based learning for rural-based family medicine residents. | Outcomes included pre- versus post-course changes in knowledge (15-item quiz), attitudes (12-item survey), self-perceived comfort levels (19-item survey) and skills (3 long Objective Structured Clinical Examination stations (OSCEs), with accompanying standardised score sheets). |
| Reymond, 2005, Australia | Uncontrolled before/after | To pilot and evaluate an intervention to increase the palliative care capacity of primary health care providers in rural and remote communities. | Code: Healthcare Provider Training  The intervention consisted of workshops tailored to local palliative case loads facilitated by a specialist palliative care team from the Mt Olivet Hospice Service. Workshop content consisted of introductory didactic teaching based on participant-nominated topics, small group case management discussions and a session devoted to psychosocial and counselling inputs. | The primary outcomes included cost per participant, GP reach, evaluation of educational (confidence, learning objectives) and clinical objectives (knowledge, skills and management of palliative symptoms confidence). |
| PCOS | | | | |
| Boyle, 2016, Australia | Cross-sectional | To conduct a process evaluation of a pilot clinic which aimed to provide a comprehensive, evidence-based service for women with PCOS. | Code: Reorganization of Services  A PCOS clinic based on evidence-based guidelines for PCOS management was developed to provide evidence-based health care and improve health care outcomes and service delivery. | The outcome variables of interest were the consistency in which the guidelines were applied, barriers and enablers to using the service and the clinic’s ability to meet health needs. These outcomes were assessed through medical records, semi-structured interviews with key informants and focus group discussions with women who attended the clinic. |
| Post Abortion Care | | | | |
| Kiemtore, 2017, Burkina Faso | Uncontrolled before/after | To evaluate the results of an intervention to improve post-abortion care (PAC) in rural areas of Burkina Faso. The project’s objectives were to improve the skills of service providers in family planning and PAC, equip health facilities with manual vacuum aspiration (MVA) devices and provide them with misoprostol, improve the use of misoprostol in the management of incomplete abortion, and improve the uptake of post-abortion contraception. | Code: Healthcare Provider Training  Provided training in health care and equipment for PAC to 56 health facilities. Three 12-day training sessions were organized at a teaching hospital. The training covered theoretical and practical training in modern methods of contraception (intrauterine devices, implant, injectable contraceptives, pills, and condoms) and practical training on PAC (MVA and use of misoprostol). | Outcome measures included comparing clinical and patient statistics from the 45 rural health facilities before and after the intervention. The variables evaluated included the presence of functional MVA devices, percentage of incomplete abortions managed by MVA, percentage of incomplete abortions managed by misoprostol, percentage of incomplete abortions treated by inappropriate methods, post-treatment complication rate, and percentage of women receiving a modern method of post-abortion family planning. |
| Pregnancy-Related | | | | |
| Bonnell, 2018, Dominican Republic | Uncontrolled before/after | To evaluate the feasibility and acceptability of using mobile health technology by community health workers (CHWs) to improve the identification of pregnancy complications and access to care for pregnant women. | Code: Training of Lay Community Members  CHWs in three communities were taught to provide third-trimester antenatal assessment, upload the data on a mobile phone application, send the data to the local physician who monitored data for “red flags,” and call directly if a mother had an urgent problem. | Data were measured at baseline and after baby delivery (or follow-up visit). Variables measured included gravida; parity; abortions (loss of pregnancy before 20 weeks gestation); natimuertos (loss of pregnancy after 20 weeks gestation); the number of cesarean deliveries; birth date of most recent child; method of delivery; child’s general health at birth; ages of children; last menstrual cycle; due date; the number of prenatal visits with current pregnancy; a medical history of thalassemia; whether mother has received tetanus vaccination, and if so how many; medications being taken, including folic acid, iron, and calcium; maternal height and weight (body mass index); and hemoglobin. Women were also assessed for alcohol, tobacco, substance use, and safety in the home. A senior supervising CHW and/or the physician obtained a hemoglobin level. Additionally, the number of participants lost to follow-up was also measured. |
| Martinez, 2018, Guatemala | RCT | The goals of the trial were to characterize, for the first time, baseline rates of complication detection and facility-level referral by traditional birth attendants (TBAs) in rural Guatemala and to evaluate the impact of the mHealth system on these rates. | Code: Decision Support + Healthcare Provider Education  A perinatal monitoring mHealth platform was introduced in the daily practice of participating TBAs. The platform was a decision support tool that could be used on an Android phone. The platform provided integrated use of peripheral sensor devices, including a pulse oximeter, a hand-held 1-dimensional Doppler ultrasound device, and—via a customized camera application—a self-inflating oscillometric blood pressure cuff. Additionally, the application could collect simple demographics, maternal and perinatal symptoms and clinical signs, maternal vital signs (pulse, oxygen saturation, systolic and diastolic blood pressure), and fetal heart rate. Any abnormal findings found by the TBAs trigger automatic communications with the on-call clinical team by voice call or text message. Training for the application was led by study nurses, where they first refreshed key medical concepts related to perinatal complications, including risk factors for maternal and neonatal morbidity and mortality, indications for referral to higher levels of medical care, the importance of timely assessment, and use of the smartphone decision support platform. | The primary outcome was the number of monthly referrals to facility-level care from TBAs for maternal and perinatal complications, adjusted by the monthly birth volume. A secondary outcome was the proportion of completed referrals, defined as successfully receiving facility-based care after a TBA-initiated referral. |
| Yugbaré Belemsaga, 2018, Burkina Faso | Uncontrolled before/after | To reduce maternal and newborn mortality and morbidity within the year after childbirth in four sub-Saharan African countries. | Code: Extending Scope of Practice - Non-FP  MOMI interventions, including integrating maternal and infant services in the postpartum (PP) period at day 6–10, weeks 6–8, and month nine, were implemented from September 2013 to December 2015. | Outcome indicators were: attendance of PPC on days 6–10 and weeks 6–8, provision of PP family planning counselling, and management of PP morbidity in mother and infant. |
| Shiferaw, 2016, Ethiopia | Cross-sectional | The impact of mHealth interventions on key maternal health outcomes in low-income settings. | Code: Coordination/Referral Pathways  Health workers in the intervention group received an Android phone loaded with an application that sends reminders for scheduled visits during antenatal care (ANC), delivery and postnatal care (PNC), and educational messages on dangerous signs and common complaints during pregnancy. | Primary outcomes were the percentage of women who had at least 4 ANC visits, institutional delivery and PNC visits at the health centre after 12 months of intervention implementation. |
| Preventative Care | | | | |
| Nagykaldi, 2017, United States | Uncontrolled before/after | To implement and evaluate a sustainable, rural community–based patient outreach model for preventive care provided through primary care practices (PCPs) in a rural county in Oklahoma. | Code: Coordination/Referral Pathways  A Wellness Coordinator (WC) was used to help county residents receive evidence-based preventive services. The WC used a registry connected to electronic medical records via HIE and called patients at the county level based on PCP-prioritized and tailored protocols. The registry identified patient-level preventive care gaps, tracked outreach efforts, and documented the delivery of preventive services throughout the community. | Return on investment (ROI) for prioritized preventive services was estimated in participating organizations. Rates of preventive service delivery for selected services were calculated for a pre-implementation baseline year and the intervention year for each participating practice and the hospital. |
| Gray, 2010, Canada | RCT | To evaluate the cost-effectiveness of Anticipatory and Preventive Team Care (APTCare). | Code: Coordination/Referral Pathways  At-risk patients were randomly assigned to receive usual care from their family physicians or APTCare from a collaborative team. APTCare consisted of being assigned to the care of 1 of 3 NPs, the pharmacist, and their usual family physicians. Care provided by the NPs and pharmacists was delivered almost exclusively in the patients’ homes, while patients continued to see their family physicians in the office. The main objective of the intervention was to ensure evidence-based disease management and solid social support for patients. Additionally, some patients received a telehealth system in the home for remote monitoring of clinical parameters (e.g., blood pressure, weight, glucose levels, and blood oxygen levels) by the NPs. | Cost-effectiveness and the net benefit to society of the APTCare intervention. Costs measured included clinical-related costs (such as medication, lab tests, physician salary etc.), APTCare-specific costs (such as staff training, medical supplies etc.), overhead, and human resource costs. Each participant's quality of care score was also calculated, and patient characteristics were recorded. |
| Skin Infections | | | | |
| Andrews, 2009, Australia | Uncontrolled before/after | To report the results of a community-based collaboration within the East Arnhem Region, which aimed to reduce the prevalence of pyoderma and scabies (skin infections) in Aboriginal children. | Code: Training of Lay Community Members  Using a standard data collection form, trained local community workers (local Aboriginal people) and a project team member were screened for skin infections. Skin infections were diagnosed clinically with a pictorial flip chart developed. Those who screened positive for infection were given a topical ointment or referred to a local clinic according to current guidelines. | Outcomes were recorded over three years, from September 2004–August 2007. Children were screened for skin infections using a standard data collection form. Additional data was collected for a subset of children seen during school screening, which included: appearance of pyoderma (crusted, purulent or flat/dry), site of pyoderma (upper body or lower body), the number of sores (<5, 5–20 or >20) and, for those with scabies, whether or not the infestation had become infected (scabies with a superficial bacterial skin infection = pyoderma [1]). |
| Sexually Transmitted Infection | | | | |
| Bocoum, 2017, Burkina Faso | Cohort | To develop and implement an intervention that integrates a rapid screening test for syphilis in ANC services in rural primary health care facilities in Burkina Faso. | Code: Healthcare Provider Training  The intervention involved the development and implementation of a decentralized model of syphilis screening among pregnant women. The components of this intervention included: (1) providing onsite training of health workers involved in ANC, (2) providing supplies and drugs to health facilities for diagnosis and treatment, (3) implementing a quality control system, (4) and supervision and monitoring. | Data were collected through qualitative and quantitative methods. Outcomes recorded included: socio-demographic characteristics of women, test results and medication. Interviews were conducted with healthcare workers and district managers to explore opinions on organizational and managerial issues and their experience with point-of-care tests for syphilis. Non-participant observations were conducted during ANC visits. |
| Thomas, 2000, United States | Uncontrolled before/after | To determine if the LHA programme could be efficacious in decreasing rates of sexually transmitted diseases. | Code: Training of Lay Community Members  They implemented a lay health advisor (LHA) intervention to decrease rates of sexually transmitted disease (STD) among rural low-income African Americans. Participants were identified by their peers as natural helpers who were trained as LHAs to disseminate information, change attitudes, and improve skills among their social networks in the neighbourhoods with the highest STD rates. The behaviours they targeted involved care-seeking and condom use. | The outcome evaluation included cross-sectional household surveys conducted before the intervention and after 18 months of activity. Outcomes measured included: the proportion seeking prompt care for an STD, those seeking screening for infection after suspecting exposure and consistent condom use with main partners. |
| Sleep | | | | |
| Parsons, 2017, United States | Cross-sectional | To describe the feasibility of an ECHO (extension for community healthcare outcomes) program for sleep medicine. | Code: Healthcare Provider Training  ECHO creates a virtual learning community through video-teleconferencing, combining didactics with individualized clinical case reviews. Multidisciplinary providers were invited to attend up to 10 stand-alone, 1-hour sessions. | They examined participant characteristics and self-reported changes in practice and comfort with managing sleep complaints. They surveyed participation barriers among invitees with low/no attendance. |
| Speech Impairments | | | | |
| Kirby, 2018, Australia | Retrospective Cohort | To test the feasibility of providing a nurse-led annual cycle of diabetes care in remote locations and to explore patient-reported factors important in diabetes self-management. | Code: Reorganization of Services  Supervised speech pathology students on rural clinical placement and provided speech, language and communication screening, assessment and therapy to children starting kindergarten in Broken Hill, New South Wales, Australia. The students collected service outcome data for children in the program. | Quarterly clinical outcomes and lifestyle changes were collected from the patient records of all patients. Categorical variables were created for patient demographics (age, sex, living alone/with others, medications) and outcomes (HbA1C level, glomerular filtration rate, weight) for analysis. Interviews were also conducted; interview questions covered perceptions of diabetes care before and during the nurse-led care and lifestyle adjustments necessitated by diabetes. |
| Surgery | | | | |
| Matousek, 2017, Haiti | Controlled before/after | To compare the population rates of elective operations between the intervention group and the two control groups before and after the patient navigation intervention. | Code: Healthcare Provider Training + Patient Education/Navigation  Trained community health workers to be patient navigators. Their role was to guide enrolled subjects through the medical process for elective surgery, including: obtaining a hospital chart, financial support, surgical evaluation, any required testing, admission and an operation should it be required to follow each subject for the duration of admission and assisted with obtaining medications from the pharmacy upon discharge and postoperative visits. One group of patient navigators worked in the community to identify patients needing surgery, enrol them, coordinate with the program administrator when each patient would be assigned to the hospital, and complete some post-operative home visits. The other navigators received the patients at the hospital and guided them through all the necessary steps. | The rate of elective operations was the primary outcome and was calculated as the ratio of elective operations to the total number of people in the group. |
| Upper Respiratory Tract Infection | | | | |
| Chiswell, 2019, United States | Uncontrolled before/after | To assess the effects of a combination patient and provider education program on antibiotic prescribing in RTIs in a rural primary care clinic. | Code: Patient Education/Navigation  The program required providers to educate patients on RTIs and antibiotics, reinforced by a summary handout they provided. Some patients were given delayed prescriptions for antibiotics as well. | The primary outcome was the rates of antibiotic prescriptions. |
| Wei, 2019, China | Cluster RCT | To assess the effectiveness of an antimicrobial stewardship programme to reduce inappropriate antibiotic prescribing in paediatric outpatients by targeting providers and caregivers in primary care hospitals in rural China. | Code: Healthcare Provider Training + Patient Education/Navigation  The intervention included clinician training on guidelines for appropriate prescribing, monthly prescribing, peer-review meetings, and brief caregiver education. | The antibiotic prescription rate in children attending the hospitals, measured by the cluster-level proportion of prescriptions for upper respiratory tract infections in 2–14-year-old outpatients, was issued during the final 3 months of the 6-month intervention period and included one or more antibiotics based on prescription records. |
| Zhang, 2018, China | RCT | To assess the cost-effectiveness of our intervention in reducing antibiotic prescribing in rural primary care facilities as measured by the intervention’s effect on the antibiotic prescription rates for childhood URTIs. | Code: Healthcare Provider Training + Patient Education/Navigation + Audit and Feedback  The intervention included concise, evidence-based clinical guidelines on URTI management. It facilitated training on using/applying the guidelines during consultations and monthly peer review meetings assessing providers’ antibiotic prescription rates (APR). Patients and caregivers received information on appropriate antibiotic use verbally and via an educational leaflet. A video with key messages on the appropriate use of antibiotics was played daily in the township hospitals' waiting rooms and public areas. | The primary outcome was the cost per percentage point decrease in the antibiotic prescribing for childhood URTIs in the intervention arm compared to the control arm. |
| Wei, 2017, China | Cluster RCT | To assess the effectiveness of an antimicrobial stewardship programme to reduce inappropriate antibiotic prescribing in paediatric outpatients by targeting providers and caregivers in primary care hospitals in rural China. | Code: Healthcare Provider Training  Clusters were primary care township hospitals in two counties of Guangxi province in China, randomly allocated to the intervention or control groups. The intervention included clinician guidelines and training on appropriate prescribing, monthly prescribing peer-review meetings, and brief caregiver education. In hospitals allocated to the control group, usual care was provided, with antibiotics prescribed at the individual clinician’s discretion. | The primary outcome was the antibiotic prescription rate in children attending the hospitals, defined as the cluster-level proportion of prescriptions for upper respiratory tract infections in 2–14-year-old outpatients, issued during the final three months of the 6-month intervention period that included one or more antibiotics. The outcome was based on prescription records and analysed by modified intention-to-treat. |
| Vaccination | | | | |
| Harry, 2022, United States | Cluster RCT | To test the effect of HPV clinical decision support (CDS) with or without HPV shared decision-making tools (SDMT) on the percentage of young adult primary care patients aged 18–26 who completed the HPV vaccination series within 12 months after  a study index visits in the CDS intervention arm clinics (one arm with and one arm without SDMT) compared to UC clinics. | Code: Decision Support  The clinical decision support provided clinicians with primary (HPV vaccination, tobacco cessation, weight management) and secondary (breast, cervical, colorectal, and lung) cancer prevention recommendations for patients at average risk meeting eligibility criteria (e.g., not up to date on HPV vaccination or breast, cervical, colorectal, or lung cancer screening; tobacco use; obesity). The system included handouts for patients prompting them to ask physicians about HPV vaccines and education on HPV vaccines to promote shared decision-making. Clinic staff were provided with the training. | The primary outcome was the difference in HPV vaccination status 12 months after index visits by the study arm. |
| Vision Impairment | | | | |
| Amritanand, 2018, India | Uncontrolled before/after | To determine the numbers identified with visual problems, establish follow‑up rates, proportions correctly referred by community health workers, and the effect of this intervention on uptake of existing services. | Code: Training of Lay Community Members  Community Health Workers underwent a training module to screen and refer individuals with visual impairments for additional services. | Used surveys to assess perceived visual impairment; any affirmative answers were given referral slips for further evaluation. Proportions of those who followed up after referral and those who were correctly referred were calculated. |
