# Supplementary material for: Interventions to improve primary healthcare in rural settings: A scoping review
Source: PLoS One. 2024 Jul 11;19(7):e0305516. doi: 10.1371/journal.pone.0305516 (PMC11239038; doi:10.1371/journal.pone.0305516)
Supplement: S4 Appendix — (DOCX) [file pone.0305516.s005.docx]

**Quality: Clinical outcomes**

| **Author, Year, Country** | **Design** | **Aim** | **Brief Intervention description** | **Outcome measurement** |
| --- | --- | --- | --- | --- |
| Alcohol Use | | | | |
| Noknoy, 2010, Thailand | RCT | To determine if motivational enhancement therapy can reduce alcohol consumption in hazardous drinkers. | Code: Patient Education/Navigation  The intervention involved three 15-minute sessions of motivational enhancement therapy over six weeks. Different counselling strategies were provided depending on the participant’s stage of change (pre-contemplation, contemplation, determination, and maintenance). | Self-reported drinks per day and week, hazardous drinking per day and week, frequency of binge drinking sessions per week, and frequency of being drunk in the past month were measured. Assessments were completed at six weeks and 3, and 6 months. Serum gamma-glutamyltransferase (GGT) was also measured. |
| Beckham, 2007, United States | RCT | To determine if a motivational interviewing session can reduce alcohol consumption in hazardous drinkers. | Code: Patient Education/Navigation  One motivational interviewing session lasting 45-60 minutes following the FRAME (feedback, responsibility, advice, menu, empathy, and self-efficacy) guidelines. | Drinks per day and serum gamma-glutamyltransferase (GGT) were measured before and after treatment. |
| Kavanagh, 2003, Australia | RCT | To determine if providing newsletters to patients in addition to GP care was more effective than GP care alone in reducing alcohol consumption in hazardous drinkers. | Code: Patient Education/Navigation  Participants were given eight newsletters focusing on cognitive content (e.g., cognitive therapy, thought challenging, problem-solving, motivation enhancement, creating goals) over 12 weeks in addition to GP care. | Alcohol consumption was self-monitored daily and reported every three months from a mailed questionnaire or telephone interview. Other outcomes included psychological distress measured with the Goldberg Anxiety and Depression Scale and Kessler-10. |
| Any Condition | | | | |
| Osae, 2022, United States | Retrospective cohort | To evaluate the attainment of clinical quality measures for pharmacist-delivered annual wellness visits (AWVs) compared with physician-delivered AWVs. | Code: Expanded Scope of Practice – Non- FP  Clinical pharmacy specialist (CPS) provided annual wellness visits (AWV) 1.5 days a week for 60 min appointments. On completion, CPS provided patients with a personalized prevention plan (preventive screening and services plan  for the next 5-10 years based on a patient’s current health and risk factors), requested prescriptions, and assisted with scheduling appointments for physician follow-up if necessary. | Data from the intervention group was compared to the usual care group. Data on quality measure eligibility, attainment, and drug therapy problems were collected through electronic chart review. AWV-related quality measures obtained from the electronic health record were organized into three categories: (1) referrals (abdominal aortic aneurysm [AAA] screenings, breast cancer screenings, colorectal cancer screenings, lung cancer screenings, osteoporosis screenings, and advanced care planning [ACP] education), (2) vaccines (herpes zoster, influenza, Pneumovax, Prevnar, and tetanus), and (3) laboratory screenings (diabetes and cardiovascular diseases). |
| Kramer, 2018, United States | Cohort | To assess the change in access to Veterans Affairs long-term care benefits and utilization for rural populations after the expansion of the home-based primary care program to rural communities. | Code: Reorganization of Services  Home-based primary care is a VA benefit that provides patients with interdisciplinary, noninstitutional long-term care for veterans with complex chronic, disabling conditions. | The primary outcomes were activities of daily living (ADL), previous VA enrollment, hospital admissions and emergency department (ED) visits as a function of time, accounting for IHS beneficiary and functional statuses. |
| Any Condition (Elderly) | | | | |
| deBattle, 2018, United States | Controlled before/after | To assess the effectiveness and cost-effectiveness of implementing a mobile health (mHealth)-enabled integrated care model for elderly, complex chronic patients. | Code: Reorganization of Services  The CONNECARE organizational integrated care model provided patients with coordinated interdisciplinary care with an eHealth platform supporting it, consisting of a patient self-management app, a set of integrated sensors, and a web-based platform connecting professionals from different settings. | The primary outcomes were changes in health status (Short-Form Survey), unplanned visits and admissions during a 6-month follow-up, and the incremental cost-effectiveness ratio (ICER). |
| Asthma | | | | |
| Brown, 2017, United States | Cohort | To assess the feasibility of incorporating telepharmacy services within a community pharmacy to deliver asthma education services in a rural community. | Code: Healthcare Provider Training  They developed an asthma education program based on the National Asthma Education and Prevention Program guidelines and Thomas Plaut’s book One Minute Asthma. All patients received the One Minute Asthma book, and the graphics and concepts were emphasized from the book during the three educational sessions. Patient education was delivered through real-time audio/video counselling using the telepharmacy equipment. The program consisted of three monthly education visits, with follow-up visits every three months over one year. The first educational visit reviewed basic asthma facts, signs of asthma exacerbation, medication(s) roles as reliever and controller, medication technique assessment and with patient involvement, and a symptom-based asthma action plan was developed. | Asthma Control Test (ACT) and FEV1% were measured at each time frame (visits at months 1, 6, 9, and 12). One-sample t-tests assessed the mean difference in patient outcomes at a given visit against established clinical benchmarks. |
| Cicutto, 2014, United States | Uncontrolled before/after | The evaluation of a primary care, asthma-focused, performance improvement program provided to a 6-county, rural-frontier region in Colorado to determine whether asthma care practices could be enhanced to become concordant with evidence-based asthma care. | Code: Healthcare Provider Training  The program included an asthma champion workshop, asthma workshops, in-clinic coaching visits, clinician support tools, patient asthma education materials and teaching aids, a resource website and practice report cards. | Adherence to guidelines measured by six practice indicators: (1) use of spirometry to confirm asthma diagnosis and to assess asthma severity, control, and responsiveness; (2) assessment of asthma control; (3) recommended pharmacotherapy according to the NAEPP step-based approach; (4) review and coaching for accurate inhaler technique; (5) provision of written asthma action plans; and (6) advanced scheduling of a follow-up asthma appointment. |
| Bender, 2011, United States | Uncontrolled before/after | To determine whether the Colorado Asthma Toolkit increases adherence to asthma management guidelines. | Code: Healthcare Provider Training  The Colorado Asthma Toolkit Program is designed to: (i) increase the capacity of primary care practices to assess and manage asthma and (ii) help clinicians to educate and support patients to increase medication adherence and effective self-management. Practice clinicians and staff received training in asthma management consistent with evidence-based guidelines and in the use and interpretation of spirometry. Practices were provided with a spirometer. Clinicians had 3 x 4-hour coaching visits and had access to a patient toolkit for self-management. | Adherence to asthma management guidelines by assessing three behaviours: (1) inhaled corticosteroid (ICS) prescriptions, (2) asthma action plans, and (3) spirometry between 1-3 months after completing all coaching visits. |
| Larson, 2010, Australia | Uncontrolled before/after | To assess the outcome for asthma patients who underwent a nurse-led patient education session with a general practice review of an Asthma Action Plan. | Code: Patient Education/Navigation  A brief patient education session was delivered by certified respiratory nurses with information on the development of asthma, signs and symptoms, triggers, treatment for asthma and living with asthma, and a general practice review of an Asthma Action plan. | Used the Asthma Control Questionnaire and the Adult Asthma Quality of Life Questionnaire to measure symptom severity and quality of life and used medical records to access previous Asthma Action plans and asthma exacerbations. |
| Saini, 2008, Australia | Controlled before/after | To compare the effect of a pharmacist-delivered rural asthma management service (RAMS) with ‘standard care’ delivered by community pharmacies on asthma symptoms. | Code: Increasing Staff Resources  A pharmacist-delivered RAMS that included standardised protocols and resources based on national asthma management guidelines. Pharmacists provided the RAMS service to recruited patients at baseline, 1-month, 3-months and 6-months after baseline. | The primary outcome was asthma ‘severity’ score - a composite score based on recency, frequency and severity of asthma symptoms, and history - calculated using the National Asthma Council’s asthma severity classification. |
| Cave, 2001, Canada | Uncontrolled before/after | To assess the effect of nurse-run clinics on patients' symptoms, pulmonary function, and healthcare utilisation over four months. | Code: Patient Education/Navigation  A nurse-run asthma clinic that provided advice on medication use, education on inhaler technique and basic asthma information according to the patient needs and the National Asthma and Respiratory Training Centre guidelines. Follow-up was arranged for reinforcement of teaching and question answering at one and four weeks. Reassessment took place at 16 weeks. | They measured the patient’s symptoms (e.g., number of days off work, night awakenings, and pulmonary function (e.g., spirometry test, bronchodilator) via assessments by the nurse and self-report. |
| Atrial Fibrillation | | | | |
| Orchard, 2020, Australia | Cross-sectional | To improve the proportion of patients screened and treated for atrial fibrillation (AF) using the refined eHealth tools and to inform strategies on AF screening implementation in the rural setting. | Code: Decision Support + Telehealth or Virtual Care  General practitioners/nurses at practices in rural Australia screened eligible patients (≥65 years of age without AF) using a smartphone ECG during practice visits. eHealth tools included electronic prompts, guideline-based electronic decision support, and regular data reports. | Primary outcomes were the proportion of screened patients with confirmed new AF, the proportion of AF and screened patients where the electronic decision support was accessed, the proportion of patients with AF who were prescribed an OAC according to guidelines, baseline AF prevalence in patients ≥65 years of age, new screen-detected AF incidence at the end of the study period in patients ≥65 years of age, rates of OAC and antiplatelet treatment at baseline and completion for patients in the OAC recommended category compared to the control.  The economic model developed in the  SEARCH-AF (Screening Education and Recognition in Community Pharmacies of Atrial Fibrillation) pharmacy screening study was adapted to evaluate the cost-effectiveness of the iECG screening in general practice. |
| Smyth, 2016, Ireland | Prospective Cohort | To test the feasibility of opportunistic screening in rural Ireland for AF. | Code: Screening  Opportunistic screening for AF. Local training was provided in each area, and each participating practice had a resource pack including information sheets, educational material and a treatment algorithm for AF. | Over six months, practices were requested to screen consecutive patients aged 65 and older, using digital palpation of the radial artery. For those diagnosed with new AF, choice of antithrombotic therapy and reasons for not choosing to anticoagulated were collected. |
| Breast Feeding | | | | |
| Dumphy, 2016, United States | Controlled before/after | To examine the effect of a primary care intervention on breastfeeding rates from the newborn visit through the 4-month visit. | Code: Healthcare Provider Training  All staff and providers received lactation-specific education through the American Academy of Pediatrics (AAP) Education Physician in Their Communities (EPIC) breastfeeding program, conducted by a contracted pediatrician. In addition, they provided general breastfeeding skills training, triage, and follow-up telephone call protocols and specifically addressed breastfeeding complications and interventions with the providers. | Two independent groups of mother-infant couplets, a pre-implementation and a post-implementation, were longitudinally evaluated on breastfeeding rates at the newborn, 1-month, 2-month, and 4-month well-child visits for exclusive, partial, and any breastfeeding rates. |
| Burn Rehabilitation | | | | |
| Wibbenmeyer, 2015, United States | Prospective Cohort | To evaluate the addition of video telemedicine to a current telephone burn transfer program. | Code: Decision Support  A telemedicine program was implemented to increase access to medical professionals with expertise in burn care. This study compared telephone-only care vs video-enhanced care on burn care and management. | A 10-question Google survey using a 4-point Likert scale was sent to the referring staff participating in the video-enhanced transfer via email the following day. The survey assessed years of medical experience, experience with burns, and evaluation of the telemedicine experience (feasibility and education). Additionally, outcomes of demographics and clinical characteristics (type of injury, fluids at admission, comorbidities, intubation, transfer), burn estimation and triage modifications after telemedical advice were compared between groups. |
| Wiechman, 2014, United States | RCT | To overcome the barriers to effective burn rehabilitation by utilizing an expanded care coordinator (ECC) to supplement the existing outpatient services. | Code: Increasing Staff Resources + Coordination/Referral Pathways  In this between-group, single-blind, randomized, controlled trial, the control group received standard outpatient care, and the experimental group received additional services provided by the ECC, including telephone calls at set intervals (24 hours postdischarge, 2, 4, 8, 12 weeks postdischarge and 5, 7, 9 months postdischarge). The ECC was trained in motivational interviewing, crisis intervention, and solution-focused counselling. They assisted patients before and after each clinic visit, coordinated outpatient services in their geographic area (physical and occupational therapy, counselling, primary care provider referrals, etc.), and helped develop problem-solving approaches to accomplish individualized goals. | Outcome measures included patient-identified goals utilizing the goal attainment scale, the burn-specific health scale-brief, the Short Form 12, a patient satisfaction survey, and a return to work survey. |
| Cancer | | | | |
| Emery, 2017, Australia | RCT | To measure the effect of community-based symptom awareness and general practice-based educational interventions on time to diagnosis in rural patients presenting with breast, prostate, colorectal or lung cancer in Western Australia. | Code: Healthcare Provider Training + Coordination/Referral Pathways  The intervention involved two components: a community component and a GP component. The community component involved a cancer symptom awareness campaign tailored for rural Australians. The GP component involved giving GPs resource cards, symptom risk assessment charts, and local cancer referral pathways implemented through multiple academic detailing visits. | The primary outcome was the total diagnostic interval (TDI), defined as the time from the first symptom to cancer diagnosis. |
| Shakya, 2016, Nepal | Uncontrolled before/after | The primary purpose of this study was to assess the knowledge of cervical cancer among women in rural Nepal and explore the feasibility and impact of a community-based awareness program on cervical cancer. | Code: Patient Education/Navigation  Community-based educational meetings on cervical cancer and its prevention were conducted among women’s groups in rural Nepal. | Questionnaires with open-ended and closed-ended questions were administered through a face-to-face interview. All interviews were performed by the primary investigator and a doctor trained in interviewing. The questionnaire consisted of sociodemographic information (age, education level, and income source), personal information (age at marriage, number of live births, number of marriages), and questions on knowledge and attitudes related to cervical cancer to determine the outcome measures of interest. The messages that were emphasized during the education session were: (1) cervical cancer is preventable, (2) risk factors, signs, symptoms, and asymptomatic nature of early cervical cancer, and (3) the importance of undergoing gynaecological examination and cervical screening. The participants were asked whether they would participate in an upcoming cost-free cervical cancer screening program two weeks after the educational meeting. |
| Dignan, 2014, United States | Cluster RCT | To determine whether academic detailing to reach rural primary care providers with colorectal cancer (CRC) screening intervention was associated with increased CRC screening. | Code: Healthcare Provider Training  Academic detailing covering four modules: CRC screening efficacy, clinical performance measures, patient counselling, and creating a screening-friendly practice environment (including tools to identify patients who need screening). Individuals who knew the local community well and were familiar with primary care practices were selected to deliver the intervention. | Outcomes were measured through a medical record review of physician recommendations for patients to obtain screening and documentation of results for fecal occult blood tests, flexible sigmoidoscopy, double contrast barium enema, and colonoscopy. |
| Honeycutt, 2013, United States | Cross-sectional | To evaluate the effectiveness of a patient navigation program among normal-risk patients for CRC. | Code: Coordination/Referral Pathways  Implement the CRC screening component of the Community Cancer Screening Program (CCSP), which involves health navigators: 1) conducting chart audits to identify patients due for screening, 2) managing provider reminder systems to prompt health care providers to refer patients for screening, 3) coordinating screening and follow-up services, 4) providing one-on-one patient education and appointment reminders, 5) assisting patients in overcoming barriers to screening (e.g., costs, transportation, literacy), 6) ensuring that the colonoscopy recall schedule, based on gastroenterologist specialist recommendation, is entered into patient charts, and 6) coordinating provider feedback on screening referral patterns. | Outcomes were colonoscopy referral, examination, and CRC screening guideline compliance during the study period. |
| Scrace, 2009, Australia | Uncontrolled before/after | To ascertain whether the Royal Flying Doctor Service (RFDS) skin cancer clinic could improve skin cancer health outcomes for the target population while providing care at a level consistent with that documented for metropolitan skin cancer clinics. | Code: Transportation  A retrospective longitudinal report compared historical controls with a dedicated fly-in/fly-out primary care skin cancer outreach clinic provided by the RFDS. The clinic was run concurrently with the regular primary care medical service; the entire focus of this additional service was on skin cancer diagnosis and management. This model was used to minimise the additional costs of providing the service. | Outcomes included: the consultation to biopsy ratio (CBR; equals the total number of consultations divided by the total number of biopsies), the biopsy to treatment ratio (BTR; equals the total number of biopsies divided by total number of non-melanoma skin cancers - treated either surgically or non-surgically), number needed to treat (NNT; equals the number of benign lesions -pigmented or non-pigmented) and excised per melanoma (defined as number of benign lesions excised plus number of melanomas excised divided by number of melanomas excised). |
| Hughes, 2005, Australia | Cross-sectional | To describe patient participation and clinical performance in a colorectal cancer (CRC) screening program utilising faecal occult blood test (FOBT). | Code: Screening  One of two fecal occult blood test kits – guaiac (Hemoccult-II) or immunochemical (!nform) – was assigned by general practice and mailed to participants (3,358 patients aged 50-74 years listed with the local practices). | The primary outcome was participant participation in the screening program. Additionally, positivity rates were recorded for each type of kit and colonoscopy outcomes were reviewed by the project’s consultant gastroenterologist (BL). The most severe diagnosis was identified for each patient. Participants were interviewed to assess patients’ self-reported complications from the procedure. |
| Johansson, 1999, Sweden | RCT | To evaluate the effects of intensified primary care on cancer patients' home care nurse contacts and to study if patients' use of home care services six months after diagnosis can be predicted. | Code: Extending Scope of Practice - Non-FP  Intensified primary care involves routines to improve general practitioners' and home care nurses' possibilities to support and monitor patients, i.e., increased information from specialist care, education and supervision in cancer care. | Demographic data and data on treatments were collected during the first six months after diagnosis. Anxiety and depression were also assessed using a standardized measure. An 18-item questionnaire was developed to assess patients' contacts with the home care nurses and the perceived benefits of these contacts six months after diagnosis. |
| Kinsinger, 1998, United States | Cluster RCT | To evaluate whether a practice facilitation intervention with GPs will result in better breast screening rates. | Code: Audit and Feedback  A practice facilitation intervention that included (i) identifying a breast cancer screening performance gap through audit and feedback, (ii) working with staff to document how they would revise practice activities to improve screening, (iii) provision of materials for tracking/prompting, tailored for each practice and (iv) an in-service educational program on breast cancer screening. | Breast cancer screening performance (primarily mammography and CBE) was collected through cross-sectional reviews of randomly chosen medical records. |
| Cardiovascular disease | | | | |
| Shetty, 2017, India | Prospective Cohort | To demonstrate the feasibility of a telecardiology system to link rural clinics to a teaching hospital. | Code: Extending Scope of Practice - Non-FP  Five rural clinics were linked to a teaching hospital, using an inexpensive system of cardiographs and tablet PCs to transmit ECGs to the hospital and have them interpreted by cardiologists. Training was provided to clinical staff. | Outcomes included metrics of data quality, system performance, and operational variables related to the recording of quality, noise-free ECGs, the transmission of ECGs from the clinics to the hospital, ECG interpretation, and response to PCP. In addition, data on the prevalence of ECG abnormalities in enrolled patients and cardiologists’ advice to patients on further follow‑up were also analyzed. |
| Tomiak, 2016, Poland | RCT | To determine the effect of consultations aimed at preventing cardiovascular disease (CVD) on CVD risk factors and total CVD risk, as well as the prevalence of CVD risk factors in the population. | Code: Patient Education/Navigation  A team of two physicians and two nurses consulted with participants and used computer visualizations and action plans to motivate change in lifestyle behaviours. | CVD risk was calculated using the SCORE scale. Other outcomes included fasting blood glucose levels, lipid profile, BMI, waist circumference, blood pressure, and heart rate. |
| Blattner, 2014, New Zealand | Uncontrolled before/after | To evaluate a cardiac exercise tolerance test (ETT) service project designed to improve access to ETT cost-effectively and not compromise standards of care. | Code: Reorganization of Services  For 12 months, from Sept 2011, a generalist-led ETT service was provided in two rural hospitals in New Zealand: Dunstan in Central Otago and Rawene in Northland. The rural hospitals drew up clinical protocols with input from the participating specialists, according to established guidelines. General Practitioners were advised of the referral process by letter. A standardised clinical record form was completed by the generalist for each ETT. | Patient outcomes included: ongoing GP management, referral to a cardiologist, percutaneous intervention (PCI), and Coronary Artery Bypass Grafting (CABG). Additionally, they audited the reporting of ETT by generalist doctors. They determined the tangible costs and attempted to determine whether or not there was any rural-urban difference in the utilisation of ETT. |
| Zou, 2014, China | Cluster RCT | To evaluate the preliminary effects of a systematic CVD risk reduction package on improving blood pressure, uptake of CVD-preventive medicine and lifestyle modifications in one township hospital in Zhejiang, China. | Code: Patient Education/Navigation  The intervention included implementing a systematic CVD risk reduction package in one township hospital. This package was a simplified, user-friendly systematic guideline that included recommendations for CVD-preventive drugs, lifestyle modifications and adherence support for individuals with a high risk of CVD. | Primary outcome measures are blood pressure (measured through the mean change of blood pressure), uptake of CVD-preventive medicine (measured by calculating the proportion of individuals using CVD-preventive medicine) and lifestyle modifications (self-reported). |
| Zou, 2014, China | Uncontrolled before/after | To determine the effect of a cardiovascular disease (CVD) risk reduction package on blood pressure, preventive CVD medicine use, and lifestyle modifications in patients at risk for CVD. | Code: Patient Education/Navigation  A CVD risk reduction package was developed. It included a systematic guideline for patients at high risk of CVD. The guideline recommended lifestyle modifications and preventive medicine for CVD. | Family doctors recorded the following outcomes at each follow-up visit: blood pressure measurements, prescriptions, medication use, booked appointments, drug adherence and lifestyle changes. |
| Krantz, 2013, United States | Uncontrolled before/after | To evaluate the impact of coronary heart disease (CHD) prevention programs delivered by community health workers (CHW) on CHD risk. | Code: Patient Education/ Navigation + Coordination/Referral Pathways  CHWs carried out motivational interviewing techniques. They provided counselling and helped the participant develop a plan of action to increase healthy behaviours. Individuals at risk of CHD received extra information and medical referrals. Follow-up calls were performed two weeks after the initial session. | 10-year CHD risk was calculated using the Framingham Risk Score (FRS) from age, gender, total cholesterol and HDL cholesterol, systolic BP, hypertension, diabetes, and smoking. BMI and medication use were also recorded. |
| Burgess, 2011, Australia | Interrupted Time Series | To determine if holistic CVD risk assessment results in better identification of elevated CVD risk, improved delivery of preventive care for CVD and improvements in the CVD risk profile for Aboriginal adults in a remote community. | Code: Coordination/Referral Pathways  The intervention consisted of a team of healthcare professionals conducting “adult health checks” at homelands, township residences, workplaces and public spaces. The “adult health checks included (i) identification of patients with elevated CVD risk, (ii) chronic disease care and (iii) follow-up of patients for chronic disease monitoring and further care planning. | Outcomes were assessed via self-reported health indicators (ex., weight or smoking habits) and auditing medical records (delivery of preventative care, medication, CVD events, and iatrogenic events) in six-month intervals for three years. They assessed improvements in delivering preventive care services, prescription of CVD-related medications, and estimated relative reduction in CVD risk. |
| Balcazar, 2009, United States | Uncontrolled before/after | To evaluate the impact of the Health for Your Heart program, a community-based program directed at Hispanic communities with a high risk of CVD. | Code: Patient Education/Navigation  The intervention was Health for Your Heat, a program delivered by community health workers. It was a series of 8 lessons that involved education and providing relevant materials for people at high risk for CVD. | Outcomes included weight, BMI, low-density lipoprotein (LDL) cho¬lesterol level, high-density lipoprotein (HDL) cholesterol level, triglyceride level, glycated hemoglobin (HbA1c), systolic and diastolic blood pressure, and physical activity. The My Family Habits Scale was used to collect behavioural data. |
| Santos, 2004, United Kingdom | Uncontrolled before/after | To assess the impact of the Hearts in the Borders program on patients with coronary heart disease (CHD | Code: Healthcare Provider Training  The Hearts in the Borders program had three stages: (1) creating a guideline for the secondary prevention of CHD, (2) setting up disease registers to facilitate management, and (3) implementing a training program with a resource pack including information about motivational interviewing and risk factors. | Audits were performed to obtain measures of blood cholesterol levels, blood pressure, smoking habits, dietary and exercise advice given to patients, and prescribing of medications such as aspirin, statins, ACE inhibitors and beta-blockers. The number of hospital admissions was also recorded. |
| Weinehall, 2001, Sweden | Controlled before/after | To determine the impact of a community intervention program on preventing cardiovascular disease (CVD). | Code: Patient Education  There were population-based and individually oriented components of this intervention. The population-based component was carried out by local associations, the media, sports clubs, etc., who carried messages about healthy lifestyle factors (e.g., nutrition, physical activity, etc.). People were invited to an annual health provider survey for the individually oriented component. Once complete, health providers discussed with the participant their test results and provided medical counselling. Those at higher risk of CVD received extra medical and lifestyle information. | Blood pressure, total cholesterol levels, and whether the participant was a smoker were recorded. Measures of predicted mortality were also taken by using the North Karelia risk equation. |
| Hippisley-Cox, 2000, United Kingdom | Controlled before/after | To evaluate the feasibility and cost-effectiveness of screening and treating hyperlipidaemia in patients with ischaemic heart disease in primary care. | Code: Coordination/Referral Pathways  The intervention involved developing management plans for hyperlipidaemia with a local consultant chemical pathologist and cardiologist. Additionally, a nurse gave patients with hyperlipidaemia dietary advice and a diet sheet. All patients with persistent hyperlipidaemia after a three-month trial of diet were asked to see their usual GP to discuss lipid-lowering drugs. Patients who did not attend for follow-up were sent a reminder. | The primary outcomes measured were costs, benefits, mood and quality of life. Costs were measured by administration costs of identifying and inviting patients for screening; data entry time; number of GP consultations; number of nurse consultations; costs of all the blood tests as specified in protocol; and costs of lipid-lowering drugs and the cost of an average 10 min consultation with a GP. Benefits were estimated using the number of patients who needed treatment to prevent cardiac events and deaths. Mood and quality of life were measured via questionnaires before and six months after treatment. |
| Care Transfers | | | | |
| Malekpour, 2017, United States | Retrospective Cohort | To determine if the Rural Trauma Team Development Course (RTTDC) training was associated with the early evaluation and transfer of trauma patients in the trained rural facilities of Pennsylvania. | Code: Healthcare Provider Training  Researchers conducted a retrospective cohort study of 3 non-trauma referring facilities that had participated in the RTTDC training and were referring trauma patients to a level I trauma centre. Data were collected from the Geisinger electronic health record, trauma registry, and Emergency Medical Services (EMS) trip sheets. The study spanned from 2 years before the RTTDC training to 2 years after the course. | Demographic information, data about the mechanism of injury, Injury Severity Score (ISS), imaging before transfer, mode of transport, time to physician evaluation, time to transfer acceptance, transfer time, length of stay, complication, and mortality were collected. Researchers compared the collected data with a specific focus on time to acceptance of transfer (transfer acceptance time) and arrival at definitive care (transfer time) before and after the RTTDC training. The imaging rate before the transfer was also compared before and after the RTTDC training. |
| Chagas Disease | | | | |
| Sartor, 2017, Argentina | Uncontrolled before/after | To implement and test a strategy to increase access to diagnosis and treatment of human T. cruzi infection in sparsely populated rural sections of Pampa del Indio municipality, including 13 villages. | Code: Healthcare Provider Training  The treatment program included participatory workshops, local health personnel capacity strengthening, serodiagnosis, qualitative and quantitative PCRs, a 60-day treatment course with benznidazole, and follow-up. Parents and healthcare agents were instructed on drug administration and early detection and notification of adverse drug-related reactions (ADR). Healthcare agents monitored medication adherence and ADRs at the village level. | Outcomes measured included seroprevalence of T. cruzi infection, treatment coverage, treatment completion, medication adherence, and adverse drug reactions. |
| Cardiovascular Disease | | | | |
| Patel, 2019, Indonesia | Controlled before/after | To evaluate whether a mobile technology–supported primary health care intervention would improve the use of preventive drug treatment among people in rural Indonesia with a high risk of CVD. | Code: Decision Support + Screening + Coordination/Referral Pathways + Telehealth or Virtual Care  A multifaceted mobile technology–supported intervention facilitating community-based, guideline-based CVD risk screening with referral, tailored clinical decision support for drug prescription, and patient follow-up | The primary outcome was the proportion of individuals taking appropriate preventive CVD medications, defined as at least 1 BP-lowering drug and a statin for all high-risk individuals, and an antiplatelet drug for those with prior diagnosed CVD. Secondary outcomes included mean change in BP from baseline. |
| Childhood Diarrhea | | | | |
| Gao, 2018, China | Cross-sectional | To determine the effect of the “Child Care” intervention (CCI) in rural Primary Health Care Programs on the prevalence of childhood diarrhea in rural western China. | Code: Implementing a New Service + Screening  The "Child Care" intervention was part of the Rural Primary Health Care (RPHC) program for rural western China. This intervention involved various health services such as 1) growth monitoring, screening low birth weight and developing reasonable feeding guidance purposefully for children less than 36 months of age; 2) community nutrition interventions ;3) General administration of vitamin A twice a year for children; 4) Vaccinations for hepatitis B virus. | The outcome variable of interest in the study was two-week prevalence of childhood diarrhea (measured via a survey). |
| Chronic Disease | | | | |
| Jha, 2018, India | Cluster RCT | To evaluate the effectiveness of a nurse-facilitated, mHealth-based EDS for the integrated management of 5 chronic conditions in primary care settings of India through the mWellcare trial. | Code: Patient Education/Navigation  The mWellcare system was an Android application designed to generate EDS recommendations for managing hypertension and diabetes mellitus, comorbid depression, and alcohol and tobacco use, tailored to the participant’s profile and risk level. It allowed for long-term monitoring and message service reminders. Training was provided to staff on the use of the application. | The primary study outcomes were the between-group differences in mean change (from baseline to 1 year) in SBP and glycated hemoglobin (HbA1c) among participants with hypertension and diabetes mellitus, respectively. The secondary outcomes included the between-group difference in mean change (from baseline to 1 year) in fasting plasma glucose, total cholesterol, predicted 10-year risk of CVD with the recalibrated Framingham risk score, tobacco use, body mass index, and alcohol use. The depression score was measured only at the end-of-study evaluation. |
| Wu, 2016, China | Controlled before/after | To evaluate the effectiveness of an intervention to increase access to Basic Public health Services (BPHS) in the Henan province of China. | Code: Patient Education/Navigation + Increasing Staff Resources  The Basic Public Health Services (BPHS) intervention included: the creation of health records for rural families, health education, and health care for children under three years old, maternal health care, health care for older persons, immunization, reporting of infectious disease, management of hypertension and diabetes and treatment of severe mental illness. The county developed detailed management guidelines for each item in the package according to the National BPHS Guideline Book. It established a team to monitor each township quarterly to assess their adherence to the management guidelines. Monitoring reports captured the performance at township and village levels and determined the performance-related element of their pay. | Evaluated effects of the intervention from five aspects, i) distribution of funds; ii) workload of health services providers; iii) payment to health workers; iv) number of patient referrals between different levels of health institutes) outputs of chronic disease management (hypertension and diabetes). |
| Lawson, 2012, Canada | Uncontrolled before/after | To evaluate an enhanced collaborative care model that includes team building and the addition of a nurse practitioner to the team. | Code: Coordination/Referral Pathways  An observational, retrospective chart audit, pre-and post-implementation of the enhanced collaborative care model of patients seen by healthcare providers at a rural, community PHC practice | A broad range of national and provincially identified clinical quality of care indicators (QIs) targeting preventive and chronic disease management care were used. Thirty-four QIs targeting seven patient subgroups (sex by age) and four chronic conditions were assessed. |
| Hogg, 2009, Canada | RCT | To examine whether the quality of care (QOC) improves when nurse practitioners and pharmacists work with family physicians in community practice and focus their work on patients who are 50 years of age and older and considered to be at risk of experiencing adverse health outcomes. | Code: Coordination/Referral Pathways  At-risk patients were randomly assigned to receive usual care from their family physicians or Anticipatory and Preventive Team Care (APTCare) from a collaborative team composed of physicians, 1 of 3 nurse practitioners, and a pharmacist. Care included comprehensive chart reviews, home visits, medication management reviews, and individualized care plans that identified active health issues and management goals for the patient. | The primary outcomes of interest were quality of care for chronic disease management (CDM; adherence to guidelines) for diabetes, coronary artery disease, congestive heart failure, and chronic obstructive pulmonary disease via chart review and end-of-study QOC measures. Questionnaires assessed QOL, daily living evaluations, emergency department visits and hospitalization. Additionally, clinical outcomes of hemoglobin A1C and blood pressure were measured at the study's start and completion. |
| Chronic Kidney Disease | | | | |
| Barahimi, 2014, Iran | Cohort | This study reports the results of a chronic kidney disease (CKD) program designed and implemented in Shahreza, Iran. | Code: Screening  The program involved identifying the risk factors in patients through the national screening program; patients with hypertension and diabetes were referred to the family doctor as those at risk of CKD. Individuals at risk were referred to the laboratory to be screened for CKD. Patients identified with diabetes and hypertension for the first time had complications, were not controlled with medications, and had a progressive course were referred to the second level of CKD management. Patients were treated in specialized clinics by internists or nephrologists, nurses, and dietitians, and the type of diet, physical activity, and medication regimens were planned. If the risk of chronic complications were present, the internists would examine the patient and determine the treatment protocol. | Outcomes included rates of hypertension and diabetes, abnormal CKD tests, CKD rates, CKD stage, repeated measures of Albumin-Creatinine ratio and Glomerular Filtration rate across three time points. |
| Chronic Lung Disease | | | | |
| Doyle, 2017, United States | Cross-sectional | To report on the implementation and clinical outcomes of a community-based pulmonary rehabilitation program in rural Appalachia. | Code: Extending Scope of Practice - Non-FP  Three rural health centres and a large referral hospital worked together to establish pulmonary rehabilitation services based on AACVPR guidelines. The guideline's recommendations included staffing by a registered respiratory therapist, 12 weeks and 24 sessions of education and exercise, coverage of 8 core educational topics, assessment and attention to psychosocial and nutritional issues, encouragement of continued exercise (maintenance) after program completion, and ongoing program evaluation. | To measure clinical outcomes, a retrospective medical record study compared pre- and post-program values for the modified Medical Research Council dyspnea level, 6-minute walk test (6MWT), negative inspiratory force (NIF), respiratory disease knowledge, St George Respiratory Questionnaire (SGRQ), BODE index (body mass index, airflow obstruction, dyspnea and exercise capacity), and smoking status—the percentages of persons completing the program and participating in maintenance exercise after the program was recorded. |
| Chronic Obstructive Pulmonary Disease | | | | |
| Anatasaki, 2019, Greece | Uncontrolled before/after | To assess the feasibility, acceptability and impact of a pulmonary rehabilitation programme on respiratory patients' health and quality of life in a rural primary care setting in Crete, Greece. | Code: Patient Education/Navigation  The intervention comprised six weeks of exercise and education sessions supervised by physiotherapists, nurses and general practitioners. | Patient outcomes (Clinical COPD Questionnaire (CCQ), COPD Assessment Test (CAT), St. George’s Respiratory  Questionnaire (SGRQ), Patient Health Questionnaire-9 (PHQ-9), Incremental Shuttle Walking Test (ISWT))  were analysed descriptively. Qualitative outcomes (feasibility, acceptability) were analysed using thematic content analysis. |
| Lou, 2015, China | RCT | To evaluate a 4-year community-based health management intervention on improving the health status of patients with COPD. | Code: Healthcare Provider Training  The intervention involved two days of training for general practitioners in health management. The training included: general information about COPD, pathogenesis, risk factors, clinical manifestations, assessment of subjects’ conditions, display of exacerbation, and the stable stage of treatment and rehabilitation of COPD. In addition, they learned the follow-up process, how to help subjects quit smoking, and how to help subjects improve self-management skills. General practitioners in this study were trained according to GOLD guidelines and health management content. | The primary outcomes included BODE (body mass index [B], degree of air-flow obstruction [O], level of functional dyspnea [D], and exercise capacity [E]). Secondary outcome measures were changes in COPD knowledge, awareness, and risk factors, measured with a survey on epidemiology, prevention, treatment, and rehabilitation. Cumulative COPD deaths for all causes within 96 months were entered through our link to the China Information System for Disease Control and Prevention. Changes in anxiety and depression symptoms were measured with the Hospital Anxiety and Depression Scale (HADS). Changes in hospital admissions, emergency department visits, and changes in medication regimens were obtained by subject interview, healthcare databases, or prospectively from diary cards, and frequency was recorded. |
| Deprez, 2009, United States | Uncontrolled before/after | To describe the implementation of evidence-based changes for COPD care in primary care practices, the practice improvements achieved using this model, the challenges faced, and the lessons learned. | Code: Healthcare Provider Training  A chronic obstructive pulmonary disease (COPD) project was initiated at 18 primary care clinical practices in rural areas of northeastern Maine to improve the diagnosis and treatment of patients with COPD by adopting evidence-based and best-practice clinical guidelines for care management. Clinical guidelines based on the Global Initiative for Chronic Obstructive Lung Disease (GOLD) were implemented by the practices using the Institute for Healthcare Improvement Breakthrough Series learning session mode. During these sessions, participants were taught by faculty, experts familiar with the standards of care for COPD, and other community resources. | Baseline data to assess current COPD practice were collected for each practice before the initial learning session. Practice and patient barriers were identified through focus groups and addressed at the learning sessions and through direct contact with the practices. Changes in clinical practice and patient care were measured pre and post-initiative to evaluate the improvements resulting from the project. |
| Dementia | | | | |
| Kohler, 2014, Germany | RCT | To evaluate an existing regional dementia network on the care of patients with dementia. | Code: Coordination/Referral Pathways  Intervention patients received diagnostic evaluation and subsequent treatment according to network guidelines. The network consists of GPs and medical specialists committed to providing care and treatment for people with dementia according to structured treatment paths. Network targets included confirming an early diagnosis and differential diagnostics and delivering a person-centred and comprehensive therapy throughout the disease. Patients should benefit from the network by their ability to live independently and to remain socially included. Patients were randomized by general partitioner to the intervention or usual care groups. | The primary outcome measures were early contact with a neurologic or psychiatric specialist, dementia-specific medication as well as the quality of life of the patients. Secondary outcomes were caregiver burden and caregiver health-related quality of life. Assessments were conducted at baseline and 6-12 months later. These included demographics, QOL (measured through scales), caregiver burden (measured through scale), and cognitive impairment (measured using a Mini-Mental State Examination). Additionally, data on the utilization of medical treatment and pharmacotherapy was obtained from caregivers. |
| Diabetes | | | | |
| Ansari, 2022, Pakistan | Prospective cohort | To implement and assess the Chronic Care Model (CCM) for the self-management of type 2 diabetes in primary health care settings of rural areas of Pakistan. | Code: Patient Education/Navigation + Reorganization of Services  The intervention consisted of two main components: patient Self-Management Support (SMS) and Delivery System Design (DSD). The self-management support component involved nurse-delivered education classes for six months, three days a week, and one hour with each group of 15 patients with diabetes. The Delivery System Design component involved proactive scheduled visits of general practitioners incorporating the patients’ goals, assisting individuals in maintaining optimal health, and enabling the health system to manage its resources more effectively. | HbA1c was measured at the clinic at baseline after 3-month and 6-month follow-ups between male and female patients with diabetes. |
| Camargo, 2021, Brazil | Cohort | To evaluate a point-of-care technology for glycated hemoglobin (HbA1c) estimation and identification of new diagnoses of diabetes in primary care among rural communities in Brazil. | Code: Implementing a New Service  Previously identified individuals (via health fairs and home visits by the healthcare teams) who were diagnosed with diabetes (but without HBA1c results) or suspected of having diabetes were invited to specific clinics set up in different rural areas to have their HBA1c tested using the point of care technology. | The primary outcomes were fasting blood glucose levels and diabetes knowledge scores measured via medical records and questionnaires. Measurements were taken at baseline in 2015 and two follow-ups in 2016 and 2017, respectively. |
| Chen, 2021, China | RCT | To assess the long-term impact of an educational intervention on patients’ diabetes knowledge and fasting blood glucose (FBG) level and whether these outcomes differed between two rural counties. | Code: Patient Education/Navigation + Healthcare Provider Training  The intervention had three goals: 1) to strengthen collaboration between hospital care and PHC, 2) to improve patients’ diabetes knowledge and improved fasting blood glucose (FBG) level, and 3) to improve the knowledge and management of diabetes among healthcare professionals in primary care. The intervention for patients included education lectures, follow-up services, and special medical services, while healthcare professionals in PHC received professional skills training, team discussions, and regular meetings. | The primary outcomes were fasting blood glucose levels and diabetes knowledge scores measured via medical records and questionnaires. Measurements were taken at baseline in 2015 and two follow-ups in 2016 and 2017, respectively. |
| Kirkland, 2021, United States | Cohort | To determine whether underserved patients enrolled in a statewide remote patient monitoring (RPM) program for diabetes achieve sustained improvements in hemoglobin A1c at 6 and 12 months and whether those improvements are affected by demographic and clinical variables. | Code: Telehealth or Virtual Care  The intervention was a remote patient monitoring program that involved providing enrolled patients with a 2-in-1 blood pressure and glucose-monitoring device (FORA D40g, ForaCare®, Moorpark, CA) and glucose monitoring supplies free of charge that uploads their glucose scores for their medical team to review. The RPM program had a central coordinating hub that performed administrative functions, coordinated program operations, trained local clinical staff and routed abnormal clinical data for patients’ medical teams to review. | HbA1c values were measured at baseline, six months and 12 months. |
| Gordon, 2020, United States | RCT | To test an intervention to improve patient engagement in telehealth visits by encouraging patients to use active communication behaviours. | Code: Patient Education/Navigation  Participants in the intervention arm watched the “Speak Up!” video and received a pamphlet encouraging them to use active participatory communication behaviours in telehealth visits. | Data were collected before and after the intervention from the medical record and at telephone interviews (questionnaires). The primary outcome was HbA1c. |
| Peckens, 2020, United States | Cohort | To describe the strategies a family medicine clinic in Appalachia utilized to increase nephropathy screening rates and explore the factors predictive of nephropathy screening in patients with diabetes. | Code: Screening + Patient Education/Navigation + Healthcare Provider Training  Involved bulk orders for albumin-to-creatinine (ACR) testing and urine collection during clinic visits, enhanced patient communication through bulk communication reminders and individual patient outreach, and education of clinic providers. | To compare patients screened within the previous 12 months to those overdue for screening, 2-sample t-tests were used to examine differences in patients’ age, HbA1c, ACR, creatinine level and the distance (in miles) between the patient’s home and the clinic. |
| Prudencio, 2020, United States | Uncontrolled before/after | To assess the impact a clinical pharmacist-led comprehensive medication management (CMM) service has on outcomes for patients with diabetes. | Code: Implementing a New Service + Patient Education/Navigation  Patients were referred to pharmacists for the medication management service. During the appointments, a comprehensive medication reconciliation was completed at the start of each visit to clarify any medication discrepancies or non-adherence. The remainder of the appointment provides motivational interviewing, medication and lifestyle counselling, and clarifying any patient questions. After making the necessary adjustments and providing education to the patient, the patient is then scheduled for a future follow-up appointment with the pharmacist or the PCP based on the discretion of the pharmacist. | The primary outcome of this study is the change in the goal attainment rates of the three clinical goals of hemoglobin A1c, blood pressure, and appropriate statin therapy after pharmacist intervention. |
| Belcher, 2019, Saudi Arabia | Uncontrolled before/after | To explore the impact of patient portal use on HbA1c and fasting blood sugars in individuals with diabetes in a rural primary care clinic. | Code: Patient Education/Navigation  They were involved in sending messages twice weekly for 12 weeks via an online patient portal system. Messages contained information on general diabetes, medication, healthy living, and dietary information. | The primary outcomes were mean HbA1c & fasting blood sugars. |
| Chen, 2019, China | RCT | To assess the impact of an education-based intervention to improve  vertical integration and management of type 2 diabetes mellitus in primary care in rural China. | Code: Patient Education/Navigation + Healthcare Provider Training  The intervention had three goals: 1) to strengthen collaboration between hospital care and PHC, 2) to improve patients’ diabetes knowledge and improved fasting blood glucose (FBG) level, and 3) to improve the knowledge and management of diabetes among healthcare professionals in primary care. The intervention for patients  included education lectures, follow-up services, and special medical services, while healthcare professionals in PHC received professional skills training,  team discussions, and regular meetings. | The primary outcomes were changes in fasting blood glucose (FBG) level and health-related quality of life (measured by EQ-5D-3L questionnaire) post-intervention (one-year follow-up). |
| King, 2019, United States | Uncontrolled before/after | To evaluate the effectiveness of an interdisciplinary diabetes team model of care in assisting patients to achieve improved glucose control in a primary care rural setting. | Code: Reorganization of Services  The intervention involved developing an interdisciplinary diabetes team clinic composed of a certified diabetes educator/dietitian, a case manager, a pharmacist, nursing staff, a family medicine resident, a psychologist, and a board-certified family medicine attending physician. Patients were referred if their hemoglobin A1c (HbA1c) was ≥9% (75 mmol/mol); patients were seen for an initial consultation and for additional visits as needed. | The primary outcomes were: HbA1c percentage, systolic and diastolic blood pressure, immunization rates, and blood lipids recorded at the first clinic visit and within 1 to 6 months post initial visit, and at the most recent visit to the end of the study. |
| McLendon, 2019, United States | Uncontrolled before/after | To evaluate a grant‐funded pilot diabetes care program for rural adults. | Code: Patient Education/Navigation + Implementing a New Service  Interventions included nurse care management, telemedicine endocrinology consults, as well as diabetes self‐management education (DSME) to enhance disease management and prevention of complications. | A1C, hospital utilization via hospital claims, and patient and provider satisfaction were measured pre and post-intervention. |
| Murphy Buschkoetter, 2019, United States | Uncontrolled before/after | To evaluate a grant‐funded pilot diabetes care program for rural adults. | Code: Patient Education/Navigation + Implementing a New Service + Telehealth or Virtual Care  Interventions included nurse care management, telemedicine endocrinology consults, as well as diabetes self‐management education to enhance disease management and prevention of complications. | A1C, hospital utilization via hospital claims, and patient and provider satisfaction were measured pre and post-intervention. |
| Hotu, 2018, Australia | Uncontrolled before/after | To determine the impact of an integrated diabetes service on risk factors for micro- and macrovascular diabetes complications in three remote Indigenous Australian communities over 12 months. | Code: Implementing a New Service  A specialist diabetes outreach service was developed to support remote primary care services in delivering diabetes care. The team consisted of diabetes nurse educators (DNE) and endocrinologists. The team reviewed patients referred by the primary care teams, provided them with clinical advice and education, and helped collaboratively develop care plans for the patients. | The primary outcomes included: Glycosylated haemoglobin, lipid profile, estimated glomerular filtration rate, urinary albumin: creatinine ratio and blood pressure recorded in patient records at the initial visit and 12 months after the initial visit. |
| Meade, 2018 | Uncontrolled before/after | To evaluate the effect of pharmacist interventions and education on A1C in a rural family medicine clinic | Code: Expanded Scope of Practice – Non-FP + Implementing a New Service + Patient Education/Navigation  A pharmacist-led intervention was implemented to help patients stabilize their blood sugar levels before being referred to their GPs. Pharmacists managed their care while providing patients with diabetes education and resources (e.g., glucometers). | The primary outcome measure was a change in A1C from baseline. A1C levels were collected at baseline and 3, 6, 9, and 12 months after the initial visit. |
| Hunt, 2017, United Kingdom | Uncontrolled before/after | To examine the impact of upskilling primary care nurses on diabetes care on improving patient outcomes and reducing the burden of care on secondary care teams. | Code: Healthcare Provider Training  The intervention included a diabetes specialist nurse leading 4-hour educational sessions for primary care nurses on diabetes care and management. | The primary outcome was a reduction in HbA1c. |
| Feltner, 2017, United States | Controlled before/after | To evaluate the effectiveness of community health workers' (CHWs) health coaching and support in improving diabetes health outcomes. | Code: Extending Scope of Practice - Non-FP  Seventeen CHWs were trained to administer the study measures, provide coordination for the nurse educator, answer questions and provide supporting educational materials after clients received the intervention. All participants completed each nurse education screening visit. They all received the CHW health coaching home visits to repeat the nurse education materials and to preview the following nurse education materials on diabetes self-management education. | All participants completed all pre- and post-test surveys and clinical outcome measures (height, weight, to calculate their BMI, blood pressure, random glucose level, foot check, medication review, and HbA1c). The CHWs collected demographic and background data, including age, gender, marital status, level of education, income, federal poverty level, health insurance status, visits to diabetes educators, and New Vital Sign (NVS) test of health literacy levels. The CHWs also administered the Diabetes Knowledge Test (DKT), Diabetes Empowerment Scale-Short Form (DES-SF), and the Summary of Diabetes Self-Care Activities (SDSCA) measures pre- and post-Diabetes Self-Management Education (DSME) intervention. |
| Paz-Pacheco, 2017, Philippines | Controlled before/after | To assess the effectiveness of diabetes self-management education (DSME) in a rural agricultural town. | Code: Training of Lay Community Members  Peer educators (I.e., village leaders, a retired school principal, a village health worker, a village nutrition scholar, and homemakers) attended a two-day workshop conducted by endocrinologists. After the peer educators were trained and asked to do a return demonstration. This strategy focused on training lay health advisors to function as ‘community catalysts’ to promote a healthy lifestyle among people with diabetes in their community. Participants in both groups were given oral advice on diet, exercise, foot care, and medication compliance on each follow-up visit. The participants in the intervention group additionally received DSME. Modules developed by the International Diabetes Federation Consultative Section on Diabetes Education were translated and modified according to the participant’s level of knowledge. There were eight modules in the DSME program: (1) overview of diabetes mellitus, (2) diabetes and exercise, (3) diabetes and diet, (4) pharmacologic treatment of diabetes, (5) insulin use, (6) acute complications of diabetes, (7) microvascular and macrovascular complications of diabetes, and (8) foot care. The teaching sessions were held in the village health centres. | Data taken at baseline were again determined after three and six months. The outcome measures in both groups were analyzed in terms of differences in (1) mean or median values, (2) change from baseline, and (3) the proportion of participants achieving the recommended anthropometric and biochemical measures for persons with diabetes. |
| Mark, 2016, Canada | Uncontrolled before/after | To evaluate a replicated lifestyle intervention model in two physician-led interventions. | Code: Patient Education/Navigation  The intervention included clinically facilitated meetings featuring a presentation on healthier lifestyles, such as sugar addiction, medication management, maintaining adherence while on vacation, etc. Participants’ questions were addressed, and individuals requiring additional support were encouraged to form peer-led support groups. The weight-loss diet restricted calories to approximately 1 100 and 1 500 kcal/day for women and men, respectively; participants were instructed to avoid foods containing sugar and other refined carbohydrates and restrict dietary fat consumption. Participants were instructed not to undertake moderate or vigorous physical activity until they hit their weight loss target to assist in appetite control. | The following outcome measures included: Height, waist circumference. Weight was measured at every group visit, with participants wearing light indoor clothing. Furthermore, participants completed the PHQ-9 questionnaires to assess their mood. |
| RodriguezVilla, 2016, Spain | Retrospective Cohort | The objective of this study is to analyze the results of said teleophthalmology program, assess the epidemiological characteristics of the included population, the diagnostic capability of primary care physicians to identify diabetic retinopathy (DR) and adequate coordination with ophthalmologists, as well as assessing the savings said program produces. | Code: Extending Scope of Practice - Non-FP  Primary care physicians were trained to interpret retinographs (DR classification as per the international DR severity scale) with four-hour training workshops. In addition, two nurses were trained to carry out non-mydriatic retinographs (NMR), one posterior pole and four peripheral, assessing intraocular pressure (IOP) with applanation tonometry detecting alterations in the Amsler grid. | Outcomes included clinical measures of age, type of DM, years of evolution, treatment received (only diet, oral antidiabetics [OAD], insulin or combination of both), glycosylated hemoglobin percentage (HbA1c) taken three months before or after the retinograph date, presence of other cardiovascular risk factors (arterial hypertension, dyslipidemia, diabetic nephropathy defined as microalbuminuria >30mg/ml/24h or microalbumin/creatinine ratio of >20mg/g), cardiovascular pathologies of the ischemic cardiopathy type, ischemic encephalopathy and peripheral ischemia such as diabetic foot or amputations due to distal necrosis. Additionally, all retinographs of the sample were assessed by two ophthalmologists for accuracy. |
| Tokuda, 2016, United States | Uncontrolled before/after | To explore whether Video-Shared Medical Appointments (video-SMA), where group education and medication titration were provided remotely through video-conferencing technology, would improve diabetes outcomes in remote rural settings. | Code: Patient Education/Navigation  Participants received four weekly video-SMA group sessions (3–5 patients per group) followed by two bi-monthly booster video-SMA visits for a total of 5months (a total of 6 sessions). Each session was 120 min. Family members, friends or social support were also encouraged to participate in the sessions. A nurse practitioner and clinical pharmacist at the Honolulu VAMC facilitated sessions through video teleconferencing with participants. Both facilitators were diabetes core content experts and certified diabetes educators. The sessions included education on behavioural and pharmacological interventions for diabetes, hypertension, and hyperlipidemia. Each session focused on one or two core diabetes content areas, such as healthy eating or physical activity. During the video-SMA, participants were given an individualized cardiovascular risk report card that contained their current vitals and laboratory values. These report cards were updated with lab results, such as HbA1c and lipid panel, at the 1-month, 3-month, and 5-month visits. Medications for diabetes, blood pressure, and lipids were initiated or titrated according to their report cards based on the national VHA diabetes guidelines and the VHA national formulary. Individualized plans regarding diet, exercise, medication, and self-monitoring of blood pressure or blood glucose levels were given to patients at each visit. Telephone follow-up visits were provided on an “as-needed basis” to participants regarding self-monitoring, management skills, and laboratory values and occurred on average once twice monthly. | The primary outcome of this study was to assess changes from baseline in HbA1c measured at five months. The secondary outcomes included changes in blood pressure and fasting lipid values (LDL-c, triglycerides) in the same time frame. Blood pressure was measured using the standard methodology by clinical staff, with the participant seated using an automatic electronic cuff. HbA1c and lipid tests were performed in a Clinical Laboratory. The third outcome of interest was ED visits and hospitalizations recorded through chart review. |
| Bodicoat, 2015, Spain | Uncontrolled before/after | To assess whether a continuous quality improvement programme in primary care for people with type 2 diabetes led to better care and outcomes in hard-to-reach groups. | Code: Healthcare Provider Training + Audit and Feedback  Group of Study of Diabetes in Primary Care (GEDAPS) was based on continuous quality improvement methodology. It primarily consisted of regular publication of guidelines, workshops and seminars, and data audits and feedback for process outcomes. Workshops to disseminate the GEDAPS guidelines and recommendations and to propose local corrective interventions were held in Primary Health Care centres approximately every year and were delivered by region. One general practitioner and one nurse attended from each centre, and they were provided with teaching slides to pass on their gained knowledge to other members of their centre. Regardless of whether they attended the workshops, guidelines and proposals were available to healthcare professionals. | Process, intermediate and ﬁnal outcomes were used as outcome variables. Process indicators pertain to the organisation, such as 2–4 GP/nurse visits, which were perceived to indicate sufﬁcient care without overburdening patients with appointments. Other process indicators were the occurrence of at least one measurement of blood pressure, HbA1c, total cholesterol, and weight, of screening for fundoscopy and microalbuminuria, and of a foot examination. The intermediate patient outcomes included reaching target levels for HbA1c, HDL cholesterol, total cholesterol, body mass index, blood pressure, and smoking status. Final patient outcomes were the presence of foot ulcers, nephropathy, retinopathy, coronary artery disease, stroke or transient ischaemic attack, and hospital admission for amputation, hypoglycaemia. All outcome variables were binary. |
| Kirby, 2015, Australia | Uncontrolled before/after | To test the feasibility of providing a nurse-led annual cycle of diabetes care in remote locations and to explore patient-reported factors important in diabetes self-management. | Code: Extending Scope of Practice - Non-FP  The same nurse visited each patient over a year, from February 2013 to February 2014. Patients’ weight and girth were measured at the beginning, and the end of the pilot, and advice was given about medications, diet, weight loss and exercise. In addition, lifestyle changes were reviewed at quarterly intervals by the CDN under GP supervision, thereby conforming with the diabetes management guidelines of the Royal Australian College of General Practitioners. | Quarterly clinical outcomes and lifestyle changes were collected from the patient records of all patients involved in the pilot. Categorical variables were created for patient demographics (age, sex, living alone/with others, medications) and outcomes (HbA1C level, glomerular filtration rate, weight) for analysis. Interviews were also conducted. Interview questions covered perceptions of diabetes care before and during the nurse-led care and lifestyle adjustments necessitated by diabetes. |
| McDermott, 2015, Australia | Cluster RCT | Researchers aimed to evaluate the effectiveness of a community-based health-worker-led case management approach to the care of Indigenous adults with poorly controlled type 2 diabetes in primary care services in remote northern Australia. | Code: Healthcare Provider Training + Coordination/Referral Pathways  Each site allocated to the intervention arm recruited an Indigenous health worker resident in the community (selected by the health service) to work as part of the primary care team and allocated a caseload of 9-26 clients. All health workers at the commencement of the study received intensive 3-week training in clinical aspects of diabetes and other chronic condition care, including how to support patients in self-management skills, advice on medications, routine foot care, nutrition, smoking cessation, follow-up referrals to other providers, and scheduled tests. The roles of the health workers included helping patients make and keep appointments, understand their medications and nutrition and the effects of smoking and, where appropriate, work with the family to help support the patient in self-management. The curriculum included specific training and practice in Rationale for the chronic care model and evidence-based management and treatment goals in diabetes, hypertension, chronic obstructive pulmonary disease, renal disease and coronary heart disease. “Hands-on” case management: regular client home visits, including primary diabetes care (scheduled clinical checks and blood tests, counselling and referral as per the clinical guidelines supported by the clinical team). During the 18-month intervention period, the health workers attended two workshops where they underwent refresher training, including in Good Clinical Practice and reflective practice. During these sessions, they reported on their patients’ progress and shared approaches to problem-solving with the clinical support team and peers. | The primary outcome measure for this trial, glycemic control (HbA1c), was measured using standard high-pressure liquid chromatography methods. Test of Functional Health Literacy for Adults (TOFHLA) was administered at study enrolment to all participants to gauge the patients’ general understanding of health messages and procedures. Quality of Life was estimated using the Assessment of Quality of Life (AQoL) multi-attribute utility instrument. Socio-demographic data was self-reported, including years of formal education, household income, employment, food insecurity, current smoking and medication adherence. |
| Branda, 2013, United States | Cluster RCT | To determine the effectiveness of decision aids to support antihyperglycemic medication and statins for diabetes management in nonacademic and rural primary care clinics. | Code: Decision Support  The intervention involved primary care practices comparing decision aids to support antihyperglycemic medication and statins to “usual care” for diabetes management. | Decisional outcomes were assessed using a survey to ascertain their knowledge of the medications and their risk of a heart attack without the statin medication. Additionally, they measured decisional comfort, efficacy, and satisfaction with the Decisional Conflict Scale. A fidelity checklist was also used to review the video encounters of physicians to see whether they were to use the decisional aids as intended. Clinical outcomes included reviews of medical records for medication use and change in key laboratory parameters (lipid profile, Hb1A1c, LDL-cholesterol). Pharmacy records were used to estimate medication adherence. |
| Bray, 2013, United States | Controlled before/after | To determine the effectiveness of a redesigned primary care model on patients’ glycemic, blood pressure, and lipid level control. | Code: Patient Education/Navigation + Reorganization of Services  African American patients with type 2 diabetes received point-of-care education, coaching, and medication intensification from a diabetes care management team of a nurse, pharmacist, and dietitian. African American patients received usual care in 5 randomly selected control practices matched for practice and patient characteristics. Six key elements of the intervention design were: education with behavioural coaching, treatment intensification, point-of-care management, expanded clinic staff roles to facilitate management, a team-care approach, and physician leadership. Full details regarding the program’s design, including a detailed description of clinic staff and leadership training, patient flow protocols, implementation details, start-up budgets, and staff roles. A nurse, pharmacist, and dietitian care manager travelled to the three intervention practice sites on different days and provided proactive, individualized, office-based care management and follow-up as part of the usual office visit for adult patients with type 2 diabetes mellitus. Patients with new-onset type 2 diabetes mellitus or a hemoglobin A1c level greater than 7.5% were scheduled to be seen by one of the care managers. | The following patient data were then collected by unblinded staff at baseline, 18 months (intermediate), and 36 months (long term): demographic characteristics (age, sex, insurance, duration of diabetes); height, weight, and blood pressure using standard procedures; and blood specimens obtained by venipuncture and measured in the hospital laboratory using standard laboratory procedures for total, LDL, and high-density lipoprotein (HDL) cholesterol, and glycemic control (hemoglobin A1c). |
| Pape, 2011, United States | Cluster RCT | This study evaluated the impact of remote physician-pharmacist team-based care on cholesterol levels in patients with diabetes mellitus (DM). | Code: Telehealth or Virtual Care  The intervention included remote physician-pharmacist team-based care focused on managing cholesterol in DM using a health information technology tool CareManager. It provided automated DM-related point-of-care prompts, a Web-based registry, and performance feedback with benchmarking. According to protocol, the pharmacy practitioner reviewed the patients with elevated LDL-C levels in medical charts. The pharmacist develops individualised, evidence-based treatment recommendations based on the patient's medical conditions and medication history, including medication therapy and follow-up laboratory monitoring. The proposed treatment plan was electronically sent to the physician for review. The physician had the option to ignore the recommendation, act on the recommendation, or approve the intervention by the pharmacist. | Study outcomes included the difference in low-density lipoprotein cholesterol (LDL-C) goal attainment, mean LDL-C, prescribed lipid-lowering therapy, and patient satisfaction between the intervention and control arms. |
| Xin, 2010, China | Cross-sectional | To develop and evaluate a simple tool, using data collected in a rural Chinese general practice, to identify those at high risk of Type 2 diabetes (T2DM) and prediabetes (PDM). | Code: Implementing a New Service  All subjects were invited to attend a baseline health examination. Two different cohorts were used in this study. Cohorts 1 and 2 consisted of randomly selected halves of the participants. The first group was used to devise the tool, and the second cohort was used to validate the tool. Researchers also built two models using a classification tree method. The target variable depended on whether PDM plus T2DM or pure T2DM was present or absent. The risk factors included in the classification tree model were age, BMI, weight, WHR, waist circumference, duration of hypertension, family history of diabetes, and hypertension. Cohort 2 was also used to validate the model. | The baseline health examination included anthropometric and blood pressure measurements in addition to completing a general health questionnaire, with questions on personal and family history of disease and lifestyle factors, including smoking and drinking habits. The clinical parameters included in the model were age, gender, waist circumference, waist/hip ratio, weight, body mass index, systolic pressure, diastolic pressure, family history of diabetes, history of hypertension, smoking, drinking, and duration of hypertension. |
| Kengne, 2009, Cameroon | Controlled before/after | To evaluate the effect of a primary nurse-led care program for type 2 diabetes involving guideline-driven glucose and blood pressure control. | Code: Extending Scope of Practice - Non-FP  State-registered nurses with no experience managing chronic diseases were empowered to be directly in charge of running the clinics. They received a 1-week intensive training with a demonstration at the beginning of the program and a refresher course a year later. Three nurses were directly in charge of patient care in each clinic. Medical doctors monitored their activities monthly. During monitoring visits, areas of improvement in their knowledge and practice were identified, and relevant actions were taken. Physicians covered aspects of diabetes care from diagnosis to management and referral provided the training. The first clinic visit (30min or more) offered the opportunity for baseline assessment and educating a patient on risk factors. Blood pressure levels and weight were measured during subsequent visits, and fasting capillary glucose was assessed. Actions were taken following the treatment algorithm. | The primary outcome measures were trajectories of fasting capillary glucose and blood pressure indices and differences in the mean levels between baseline and final visits. |
| King, 2009, United States | RCT | To investigate the benefits of a novel program for disseminating guidance in the treatment of diabetes from a central specialist clinic to primary care centres with access to midlevel provider services (E.g., nurse practitioner [NP], physician assistants [PA], certified diabetes educators [CDE]). | Code: Healthcare Provider Training  In the intervention group, the clinic NPs received a 6-hour instruction in using the treatment algorithms based on ADA guidelines of care, the accompanying algorithm guidebook for reference, and ﬂow sheets for the chart record in the clinic. Diabetes specialists and NPs held telephone conference calls every 2–4 weeks and had bimonthly visits to monitor and review individual and group progress. The NPs faxed ﬂow sheets to diabetes specialists, who responded within 24h to critique the proposed treatment plan. In the primary care sites, several hours of clinic schedule per week were designated speciﬁcally for patients with diabetes to treat their diabetes and related cardiovascular risk factors. In the control site, no contact was made with the individual patients after the chart review and during the 12-month study. | The primary endpoint of this study was the change in glycosylated hemoglobin from baseline at the end of the study. Secondary variables included low-density lipoprotein cholesterol, non-high-density lipoprotein cholesterol, systolic blood pressure, and diastolic blood pressure. |
| Kirkbride, 2009, United States | Cross-sectional | To examine the relationship between the presence of a rural health clinic in a rural primary care service area (PCSA) and the likelihood of receiving recommended diabetes-related services associated with high-quality primary care for adults categorically eligible (eligible through Temporary Assistance to Needy Families [TANF] or disabled status) Medicaid beneﬁciaries with diabetes. | Code: Reorganization of Services  Retrospectively assessed differences in the rates of recommended diabetes-related primary care services within a sample of adult categorically eligible Medicaid beneﬁciaries identiﬁed as residing in PCSAs designated as urban or rural with and without at least 1 RHC, after accounting for individual subject characteristics that may inﬂuence receipt of the measured services. The study was based upon all administrative claims, encounters, and enrollment data from Oregon’s Medicaid program, the Oregon Health Plan (OHP), for the study period of 2002-2003. | The relative quality of diabetes-related primary care was assessed based on the proportion of subjects receiving the following diabetic care services at least once during a study year: hemoglobin A1c (HbA1c), lipid proﬁle, and an eye exam. |
| Pastel, 2009, United States | Interrupted Time Series | To evaluate the change caused by process redesign to identify all patients with diabetes every time they visit the clinic and improve performance and documentation of recommended process measures. | Code: Extending Scope of Practice - Non-FP  The team of providers and licensed nursing assistants (LNAs) assessed the gaps in care by walking through the process of an office visit. Key leverage points were identified, such as notifying providers of care needed that day, engaging frontline workers in completing the care, and creating a system to ensure documentation. The LNAs generated a list of patients with diabetes who had scheduled appointments every clinic day using the hospital database. A flow sheet was designed that listed the last dates of all required routine care for a patient with diabetes (I.e., the last date of A1C testing and the last date of foot exam). Flow sheets were completed by an LNA each day for scheduled patients with diabetes. The flow sheets prompted LNAs and providers to address needed care. Documentation of the day’s care was then entered into the electronic medical record by an LNA. Results and ongoing plans were displayed on a dashboard in the staff lounge and discussed during staff meetings. | To track improvement, the clinic focused on both process and outcome measures. Data were obtained monthly from the Hitchcock Data Reporting System (HDRS), a database of patient measures based on coding and documentation in the computerized information system flow sheets. The team followed rates of pneumococcal and influenza vaccinations, foot exams, eye exams, A1C testing, and LDL and blood pressure control. Data were obtained monthly from HDRS reports from February 2007 until April 2008. |
| Laatikainen, 2007, Australia | Uncontrolled before/after | To determine the feasibility of a type 2 diabetes mellitic lifestyle-based prevention program (six 90 min group sessions over eight months) in primary care settings. | Code: Patient Education/Navigation  The intervention consisted of six structured 90-minute group sessions using the Health Action Process Approach over eight months. The first five sessions occurred within the first three months, with two-week intervals between sessions. The last session took place at eight months. The sessions were facilitated by specially trained study nurses, dietitians and physiotherapists. A goal-setting approach was used to motivate individuals to progress from intention to actual behaviour change. Session content for diet and physical activity was based on Dietary Guidelines for Australian Adults and National Physical Activity Guidelines for Adults. Intervention targets included reducing weight, total and saturated fat intake and increasing fibre intake and physical activity. | Clinical measurements included height, weight, waist, hip and blood pressure measurements, all taken by study nurses before the intervention, at three months and one year. Blood pressure was measured using a mercury sphygmomanometer. At baseline and 12 months, participants were assessed using the Kessler 10 Psychological Distress Scale (K-10) and Hospital Anxiety and Depression Scale (HADS). General health was assessed using Short Form 36 (SF-36v2). |
| Ackerman, 2006, Australia | Uncontrolled before/after | To assess the impact of structured diabetes care in a rural general practice. | Code: Reorganization of Services  A pre-formatted care plan was generated using an electronic patient management system. All the GPs continued to manage patients with diabetes according to their therapeutic frameworks, including optional referral to the dietitian and diabetic educator. Patients were recalled every three months for assessment and, if necessary, management change. Two practice nurses attended to all patients and were responsible for managing the recall system, ensuring pathology tests were ordered (serum lipid levels, glycated haemoglobin [HbA1c] concentration, and annual urinary microalbumin estimation), measuring and recording the patient’s weight, waist circumference, blood pressure, and visual acuity, and for performing foot screening before every general practice visit. | The primary outcome measures included change in cardiovascular disease risk factors (waist circumference, body mass index, serum lipid levels, blood pressure); change in indicators of risks associated with poorly controlled diabetes (HbA1c, foot lesions, clinically significant hypoglycaemia); change in 5-year cardiovascular disease risk. |
| Haase, 2006, United States | Uncontrolled before/after | To evaluate a diabetes practice-level self-management and education intervention. | Code: Healthcare Provider Training + Decision Support + Patient Education/Navigation  Academic detailing in practice guidelines with algorithms for care and a diabetes self-management education program were the first critical activities in the improvement initiative. A variety of performance improvement activities were implemented. A diabetes care flow sheet was used to monitor and report process and outcome measurements. A diabetes registry to enter process and outcome data was introduced; data can be displayed by specific providers, by the organization as a whole, by patients whose A1C is > 7, and by patients who have not had an A1C in the last six months. The diabetes care team includes the patient, the primary care provider who provides medical management, and the diabetes educators (pharmacist and dietitian). | Outcome reports distributed to the providers included the two key quality measures— (1) patients with A1C > 7 and (2) patients not having a documented A1C in the registry within the last six months. Patients who have received DSME (diabetes self-management education) and MNT (medical nutrition therapy) referrals. |
| Kilkkinens, 2006, Australia | Uncontrolled before/after | To evaluate the efficacy and feasibility of a primary care-based diabetes prevention model with modest resource requirements involving a combined dietary and physical activity intervention in rural Australia. | Code: Patient Education/Navigation  The intervention model consisted of six group counselling sessions (12 months) facilitated by trained study nurses, dieticians and physiotherapists. The group counselling sessions provided individuals with dietary and physical activity guidance based on Dietary Guidelines for Australian Adults and National Physical Activity Guidelines, which aim to improve health outcomes. The Health Action Process Approach (HAPA) model and self-regulation theory are used to set individual goals and motivate individuals to progress from intention to actual behaviour change. The goals for lifestyle changes are as follows: (1) no more than 30% of energy from fat; (2) no more than 10% of energy from saturated fats; (3) at least 15 g/1000 kcal fibre; (4) at least 30 min/day moderate physical activity; (5) at least 5% weight reduction. | To evaluate the program, clinical measurements including height, weight, waist, hip and blood pressure measurements and fasting blood samples were taken before the intervention started, at three months and one year. |
| Bray, 2005, United States | Uncontrolled before/after | This study assessed the feasibility and potential for the cost-effectiveness of restructuring care in rural fee-for-service practices for predominantly minority patients with diabetes mellitus. | Code: Patient Education/Navigation + Reorganization of Services  An advanced practice nurse visited each practice weekly for 12 months, provided intensive diabetes case management, and facilitated a 4-session group visit educational program. The nurse case manager reviewed the patient’s care plan and facilitated or provided, under protocol and supervision, the contemporary diabetes care that each patient needed, including facilitating laboratory testing and referrals to other providers. The case manager also facilitated a return visit schedule and worked with office staff to create a reminder and callback system for patients who missed appointments. To track patient progress, the leadership team implemented a registry system, allowing the case manager to remind providers of care standards and diabetes disease management goals. | The program's sustainability was measured regarding improved clinic productivity and billable encounters. The proportion of patients achieving diabetes management goals, with a documented self-management goal, a documented lipid panel, current aspirin use and current foot examination. |
| Bray, 2005, United States | Controlled before/after | To explore the efficacy of combining care management and interdisciplinary group visits for rural African American patients with diabetes mellitus. | Code: Extending Scope of Practice - Non-FP  In the intervention practice, an advanced practice nurse visited the practice weekly for 12 months and facilitated diabetes education, patient flow and management. Patients participated in a 4-session group visit education/support program led by a nurse, a physician, a pharmacist, and a nutritionist. The control practice patients in a separate practice received the usual care. | Outcomes included weight, blood pressure, and HbA1c values from before enrollment in the system redesigns and from approximately 12 months after enrollment were abstracted from patient records in the CVDEMS system at the intervention practice. Data on demographics and glycemic control only were collected in the control practice. |
| Johnson, 2005, United States | Uncontrolled before/after | To describe a field-based diabetes care program for rural primary care patients and provide some evaluative data. | Code: Coordination/Referral Pathways  The intervention consisted of implementing a simple computerized registry called the Diabetes Quality Care Monitoring System (DQCMS) to support primary care clinicians in tracking key elements of care for their patient populations with diabetes. | Outcomes were assessed via medical records. Key indicators of diabetes care were recorded (hemoglobin A1c tests, blood pressure, low-density lipoprotein cholesterol, urinalyses, foot and dilated retinal exams, and pneumococcal vaccinations). |
| Siminerio, 2005, United States | Uncontrolled before/after | To determine the impact of implementing a chronic care model on providers’ diabetes care practices and patient outcomes in a rural practice setting. | Code: Healthcare Provider Training + Patient Education/Navigation  The intervention was modelled on a chronic care model of diabetes self-management education (DSME). The provider intervention consisted of education and training on standards of care and guidelines and practice using problem-based learning case studies for adherence to guidelines and DSME. The patient intervention consisted of participation in the DSME program. If the patient agreed to participate, they would participate in a biweekly five two-hour group sessions that consisted of goal setting and behavioural change strategies for managing their diabetes. | The provider intervention was assessed via a qualitative survey that insight into what they perceived as patient barriers to care and a chart review to measure adherence to guideline practices. Patients were assessed on empowerment and knowledge via questionnaires and biological health indicators (Hemoglobin A1C, serum cholesterol, blood pressure). |
| Maddigan, 2004, Canada | Cluster RCT | To examine patient-reported outcomes in a controlled trial of a multifaceted provider-level intervention to improve the quality of care for rural patients with type 2 diabetes. | Code: Coordination/Referral Pathways + Healthcare Provider Training  The intervention consisted of six monthly visits by a multidisciplinary healthcare team and was primarily directed at primary care providers. Overall, the aim was to promote the concept of vascular health by emphasizing the interaction of blood pressure, cholesterol, and glucose on macrovascular complications of diabetes. | Clinical and patient-reported outcomes were assessed after six months. Patient-reported outcomes included changes in health-related quality of life (Health Utilities Index Mark 3 [HUI3]), satisfaction with care, lifestyle (Diabetes Lifestyle Form), and adherence to self-care activities. |
| Goldhaber-Fiebert, 2003, Costa Rica | RCT | To determine whether a community-based, group-centred public health intervention addressing nutrition and exercise can ameliorate glycemic control and associated cardiovascular risk factors in type 2 diabetic patients in rural Costa Rica. | Code: Patient Education/Navigation  Before random assignment to the treatment groups, all volunteers received standard diabetes education through a lecture that reviewed type 2 diabetes and its symptoms, treatment, and associated complications. After randomization, the subjects in the intervention group began the 12-week lifestyle intervention, and the subjects in the control group were informed that they would be offered a similar program at the end of the study. The lifestyle intervention included 11 weekly nutrition classes (90 min each session) that three nutritionists taught. The course focused on portion control for weight reduction and healthier food substitutes. To harness the benefits of peer support, subjects set weekly goals for specific changes in their eating behaviours to decrease portion sizes and make healthier food choices. These goals were shared with the group at the end of class, and progress was reported at the beginning of the next class. Emphasis was placed on the importance of nutritional health for all family members, regardless of diabetes status. Each participant was given written handouts and a notebook to take notes and record food diaries. The subjects in the intervention group were invited to participate in 60-min walking group sessions three times per week for 12 weeks. | Study subjects underwent measurement of weight, height, blood pressure, glycosylated hemoglobin, fasting plasma glucose, serum total cholesterol, HDL cholesterol, and triglyceride levels at baseline and the end of the study. LDL cholesterol was calculated using the Friedewald equation. |
| Kit, 2003, Australia | Uncontrolled before/after | To develop and trial new program tools and processes for goal setting, behaviour change and self-management for Aboriginal people with diabetes | Code: Patient Education/Navigation  A one-year demonstration pilot of a diabetes self-management program, administered to 60 Aboriginal people with type-2 diabetes from two remote regional centres and run by four Aboriginal Health Workers who received training in goal setting and self-management strategies. | Health outcome data and longitudinal patient records were kept during the project. Local data kept by project coordinators to assist the recruitment and management of patients in the pilot program enabled the aggregation of basic health and wellbeing information, demographic data, and service utilisation information. The main focus was to test several tools during the program to determine the best methods for collecting overall health and well-being status information and to determine patient willingness to participate in behaviour change initiatives. Tools tested: PIH or “Partners in Health”, modified SF-12, modified Work and Social Adjustment Scale (WASAS), a diabetes assessment form, and problems and goals assessment. |
| Majumdar, 2003, Canada | Cluster RCT | To assess the effectiveness of a multidisciplinary diabetes outreach service (intervention) for improving the quality of care for rural patients with type 2 diabetes. | Code: Patient Education/Navigation  In the control region, local providers delivered usual care with the addition of three bimonthly visits by the CDA Traveling Diabetes Resource Program (CDA-TDRP). The CDA-TDRP travels to communities in rural Alberta, raising diabetes awareness and emphasizing patient self-management. In addition to bimonthly CDA-TDRP visits, the intervention region was exposed to the diabetes outreach service. The service consisted of a team of specialist physicians, nurse educators, dieticians, and pharmacists. The service travelled to the largest communities in the region monthly for six months, delivering targeted educational messages. The overall aim was to promote vascular health by emphasizing the interaction of blood pressure, cholesterol, and glucose on macrovascular complications of diabetes. These risk factors were promoted because of the burden of macrovascular disease in people with diabetes, the availability of effective therapies, and their emphasis on the CDA clinical practice guidelines. For the most part, educational messages were delivered by specialist physicians to small groups of primary care physicians (i.e., two to six), using techniques of group academic detailing. The study pharmacist also met with all local primary care physicians for one-on-one academic detailing. | The primary outcome measurement was the improvement in the care of patients with diabetes. Researchers deﬁned this as a 10% improvement over baseline in any of the following after six months: blood pressure, total cholesterol, or HbA1c. As additional quality indicators, researchers examined changes in target medications, i.e., medications for lowering blood pressure, cholesterol, and glucose levels. Trained study coordinators collected baseline data through interviews, physical assessments, laboratory testing, and self-report questionnaires. Subjects were followed-up six months after study entry when all the measurements above and questionnaires were repeated. |
| Simmons, 2003, Australia | Uncontrolled before/after | To describe the effectiveness of an integrated primary–secondary care diabetes clinic on metabolic control among indigenous patients in a rural community. | Code: Reorganization of Services  A weekly diabetes specialist clinic was established adjacent to the rooms used by GPs, nurses and a podiatrist. Each clinic lasted 2–2.5 h, including appointments with a maximum of two new patients and between one and five follow-up patients. Each patient had a provisional care plan developed by the specialist with the patient (and, when possible, other care providers and family members) and then modified on immediate discussion with the GP, nurse and AHW. The final plan was then discussed with the patient. GP and specialist follow-ups were tailored to need, and the nurse reviewed blood glucose results weekly and, where necessary, discussed with the GP, nurse or Aboriginal health worker (AHW). | The AHW or community nurse measured weight, height (on the first visit), blood pressure, finger prick glucose testing and HbA1c using the DCA2000. |
| Maislos, 2002, Israel | Uncontrolled before/after | To evaluate a mobile diabetes clinic aimed to provide comprehensive, interdisciplinary care to patients with diabetes residents in a semi-rural area. | Code: Patient Education/Navigation  Researchers created a mobile diabetes clinic aimed to provide comprehensive, interdisciplinary care to patients with diabetes that live in a semi-rural area. The mobile tertiary care diabetes clinic comprised a diabetologist, a diabetes nurse-educator and a dietitian. The clinic regularly visited primary-care facilities in three towns in a semi-rural area of southern Israel and applied a standardised computer-based clinical protocol. Patients received dietary counselling from a dietitian, advice on physical activity, assessment by a physician specializing in diabetes care and an educational session with a diabetes nurse-educator. The patient’s specific educational requirements were individualized at the first encounter with the therapeutic team, and a structured educational program was implemented accordingly. | Main outcome measures included clinical practice parameters such as nutritional advice by a dietitian, interaction with a diabetes nurse-educator, performance of periodic ophthalmologic examinations, and measurement of microalbumin excretion. Additional outcome measures included changes in clinical variables, such as body mass index, systolic and diastolic blood pressures, fasting plasma glucose, hemoglobin A1c, and low-density lipoprotein and high-density lipoprotein cholesterol. |
| Mayer-Davis, 2001, United States | RCT | The pilot study had two aims. The first was to confirm the feasibility and determine the optimal study design for evaluating nonpharmacologic strategies for weight management in a rural primary-care setting. The second was to determine if there was a difference between a formal, systematic program evaluation vs an informal evaluation of a program for continuous quality improvement. | Code: Patient Education/Navigation + Increasing Staff Resources  The intervention consisted of combined research-based weight loss interventions with continuous quality programming. Participants were randomized to one of two intervention groups. Each group had a different interventionist; however, both groups received the same 8-week weight management intervention. Participants met with the interventionist once per week (two individual and six group sessions). The intervention emphasized frequent and sustained contact between the interventionist and participants, a low-calorie/ low-fat diet, moderate physical activity, and self-monitoring tools for eating and physical activity. The weight management materials contained a “toolbox” to provide guidelines for monitoring and supporting the participant’s adherence to the program. It also provided problem-solving ideas for dealing with issues such as lack of weight loss, weight regain, resistance to self-monitoring diet and physical activity and assistance in meeting fat gram, calorie and physical activity goals made each week. Weekly group activity sessions were offered for participants who wanted to incorporate them into their activity plan. | Information regarding participants’ age, insulin use, gender, ethnicity, education, income, current employment, and marital status was collected by standardized interview. Weight and height were measured using a Detecto balance beam scale with a stadiometer at randomization, week four, and at the end of the study. Fasting blood glucose measurements were ascertained by fingerstick, and random blood glucose measurements (nonfasting) were taken at subsequent screening visits and periodically during the intervention. |
| McDermott, 2001, Australia | Cluster RCT | To evaluate a system for improving diabetes care in remote Indigenous communities. | Code: Reorganization of Services  A diabetes outreach service (comprising a diabetologist, nutritionist, podiatrist and diabetes healthcare worker) saw individual patients on a referral basis, providing clinical, nutritional and general health advice. A diabetes recall system was established at eight of the 21 sites, staff training in basic diabetes care, regular phone calls from the project officer, a two-monthly newsletter and a mid-project workshop. The researchers visited each of the eight intervention sites to negotiate with the local healthcare staff as to how the recall system would be managed, to set up the recall system, and to conduct clinical training in the basics of diabetes care (checking weight, blood pressure, visual acuity, feet, blood for glycosylated haemoglobin (HbA1c) and lipid levels. The healthcare workers designed and used a simple recall card system in the eight intervention sites. This visit was followed by regular (initially, weekly) phone calls and a newsletter every eight weeks. | The primary outcome measures included regular checks of weight, blood pressure, eye and foot care, serum lipid levels and glucose monitoring and control, urinary albumin to creatinine ratio and serum creatinine levels, and administration of recommended vaccines; hospitalisation in the previous 12 months. |
| O'Grady, 2001, New Zealand | Uncontrolled before/after | A diabetes club was established to support self-care and improve diabetes management in a rural Northland, New Zealand community. A structured approach to care and an associated audit were also introduced. | Code: Reorganization of Services  Researchers set out to evolve a new method of care based on group meetings with family and community involvement. There were weekly meetings, outside the health centre, in homely and non-institutional surroundings. Consultations with a doctor, dietitian, nurse, podiatrist or retinal photographer were voluntary and optional, with staff operating in a supportive partnership. Consultations were conducted as part of the group (with permission) and privately. Clinical strategies included the provision of medical care by the general practitioner with specialist back-up and the active introduction of insulin, either alone or at night in combination with tablets by day, among those receiving maximal oral therapy. | The evaluation was undertaken using the annual audit process of the South Auckland Diabetes Project. Measures included regular weight, blood pressure and blood glucose assessment, and foot care with periodic checking of lipids, renal function, eyes and a measure of long-term glucose control (initially fructosamine and then HbA1c as it became available). |
| Diabetes & Depression | | | | |
| Cummings, 2019, United States | RCT | To evaluate the effect of cognitive behavioural therapy (CBT) plus lifestyle counselling in primary care on hemoglobin A1c (HbA1c) in rural adult patients with type 2 diabetes (T2D) and comorbid depressive or regimen-related distress (RRD) symptoms. | Code: Implementing a New Service  Compared with usual care, the intervention was a 16-session severity-tailored CBT plus lifestyle counselling intervention. The behavioural intervention was delivered by a team of trained behavioural providers who provided lifestyle coaching, CBT sessions, navigation, and social support. Primary care providers were offered consultation with a diabetologist to optimize medical management. | Primary outcomes included HBA1c, regimen-related distress, depressive symptoms, self-care behaviours, and medication adherence across 12 months. |
| Lutes, 2018, United States | RCT | To determine the feasibility of delivering and evaluating coordinated and integrated behavioral and medical care for adult patients with uncontrolled T2D with comorbid diabetes-related distress and depressive symptoms. | Code: Reorganization of Services  The intervention involved 16 sessions of cognitive and behavioural intervention tailored to symptom severity across 12 months, along with routine medical care delivered by a team of behavioural providers working together, including a nurse care manager and a doctoral student in clinical psychology or a psychologist. | Outcomes included change from baseline to 12 months in HbA1c, diabetes-related distress (measured by Diabetes Distress Scale (DDS-17)), depressive symptoms (measured by Patient Health Questionnaire (PHQ-9)), and diabetes self-care activities (measured by Diabetes Self-Care Activities Measure (SDSCA)). |
| Diabetes & Hypertension | | | | |
| Worster, 2020, Mexico | Prospective Cohort | To determine whether a Community Health Worker-led intervention targeting diabetes and hypertension could improve markers of clinical disease control in rural Mexico. | Code: Implementing a New Service + Training of Lay Community Members  The community health worker (CHW)-led intervention follows a community-based accompaniment approach centred on regular home visits. CHWs were trained to deliver the intervention and participated in monthly refresher training sessions, covering themes such as motivational interviewing, recognition of emergencies and complications and navigation of interactions with challenging patients. CHWs conducted home visits with patients to provide disease counselling with motivational interviewing, assessment of medication adherence and supply and disease monitoring, including blood pressure and capillary glucose measurement. CHWs also accompanied patients to clinic visits and regularly met with physicians to discuss patient management. | Primary outcomes were glycated hemoglobin (HbA1c) and systolic blood pressure (SBP), which the CHWs measured at the home visits. |
| Saleh, 2018, Lebanon | Cross-sectional | To assess the association of a community-based eHealth intervention employed in rural and refugee settings in Lebanon with the detection and referral rates related to diabetes and hypertension. | Code: Coordination/Referral Pathways  The intervention involved community healthcare workers conducting outreach screenings for diabetes and hypertension. During the visits, referrals were made remotely via an online appointment system linked to the corresponding primary healthcare centre. | The primary outcome variable was compliance with appointments. Compliance was determined by a short phone survey with referred individuals (i.e., show-ups/no-shows and reasons for no-shows). |
| Epilepsy | | | | |
| Wang, 2006, China | Uncontrolled before/after | To test a model for treating people with convulsive epilepsy at the primary healthcare level in rural China. | Code: Extending Scope of Practice FP  Patients with convulsive epilepsy were identified at the primary care level and provided phenobarbital monotherapy. Local physicians, who were provided with special training, carried out screening, treatment, and follow-up. A local neurologist confirmed the diagnosis. | The primary outcome was the number of convulsive seizures experienced by each patient in the 12 months before the study was used as the baseline seizure frequency. Efficacy was assessed from the percentage reduction in convulsive seizure frequency from baseline. The long-term continuation rate (or retention rate) was estimated by Kaplan-Meier analysis. |
| Family Planning | | | | |
| Bonnell, 2018, Dominican Republic | Uncontrolled before/after | To evaluate the feasibility and acceptability of using mobile health technology by community health workers (CHWs) to improve the identification of pregnancy complications and access to care for pregnant women. | Code: Training of Lay Community Members  CHWs in three communities were taught to provide third-trimester antenatal assessment, upload the data on a mobile phone application, send the data to the local physician who monitored data for “red flags,” and call directly if a mother had an urgent problem. | Data were measured at baseline and after baby delivery (or follow-up visit). Variables measured included gravida; parity; abortions (loss of pregnancy before 20 weeks gestation); natimuertos (loss of pregnancy after 20 weeks gestation); the number of cesarean deliveries; birth date of most recent child; method of delivery; child’s general health at birth; ages of children; last menstrual cycle; due date; the number of prenatal visits with current pregnancy; a medical history of thalassemia; whether mother has received tetanus vaccination, and if so how many; medications being taken, including folic acid, iron, and calcium; maternal height and weight (body mass index); and hemoglobin. Women were also assessed for alcohol, tobacco, substance use, and safety in the home. A senior supervising CHW and/or the physician obtained a haemoglobin level. Additionally, the number of participants lost to follow-up was also measured. |
| Tiruneh, 2018, Ethiopia | Uncontrolled before/after | To assess the effectiveness of the BEmONC initiative, this study measures its implementation strength and examines the effect of its variability across intervention health centres on the rate of facility deliveries and the met need for BEmONC. | Code: Healthcare Provider Training + Increasing Staff Resources  Basic emergency obstetric and newborn care (BEmONC) is a primary health care level initiative promoted in low- and middle-income countries to reduce maternal and newborn mortality. Tailored support, including BEmONC training to providers, mentoring and monitoring through supportive supervision, provision of equipment and supplies, strengthening referral linkages, and improving infection-prevention practice, was provided in a package of interventions to 134 health centres covering 91 rural districts of Ethiopia to ensure timely BEmONC. | Before and after data from 134 intervention health centres were collected in April 2013 and July 2015, a BEmONC implementation strength index was constructed from seven input and five process indicators measured through observation, record review, and provider interview; while facility delivery rate and the met need for expected obstetric complications were measured from service statistics and patient records. |
| Health System Performance | | | | |
| Boltz, 2018, United States | Cohort | To evaluate a cross-organizational pilot program by comparing a nurse practitioner (NP)/telemedicine physician hospitalist programme with a traditional physician hospitalist model to assess effects on length of patient stay, mortality rates, readmission rate, Hospital Consumer Assessment of Healthcare Providers and Systems (HCAHPS) ratings of provider communication, and total hospital costs. | Code: Decision Support  The intervention for this quality improvement programme was using NP hospitalists with physician telemedicine support instead of locum physician hospitalists. Each NP underwent extensive training at the Kalispell Regional Medical Center (KRMC) hub facility, including establishing collegial relationships with physician providers at KRMC. Each NP became familiarised with local and regional resources and practised using video and telephone communications for collaboration on complex patient care management. NPs attended the American Academy of Physician Assistants ‘Hospitalist Bootcamp’, providing evidence-based continuing education on commonly encountered topics in hospital-based medicine. Next, the NP providers transitioned to providing direct patient care at CPMC with continued support from physician hospitalist providers. Every morning the NP provider reported each patient admitted to the CPMC hospitalist service to a designated KRMC physician hospitalist. Electronic medical records from both facilities were directly accessible across facilities, allowing the remote physician hospitalist to review patient data and NP documentation regarding each case. The physician was available by text, telephone or video throughout the seven-day hospitalist shift and provided consultation on all admissions, discharges and transfers and any patient care concerns that might develop throughout the day. | The Standard for Quality Improvement Reporting Excellence (SQUIRE) guidelines were followed. Using a one-year retrospective chart review, the average length of stay, mortality rates, 30-day readmission rates and provider communication ratings were compared between hospitalists that were nurse practitioners working with physicians through telemedicine support and physicians alone. |
| Chinhoyi, 2018, South Africa | Retrospective Cohort | To assess the impact of family physician (FP) supply on indicators of district health system performance, clinical processes and clinical outcomes in the Western Cape Province. The objectives were to determine the impact of FPs, nurses, medical officers (MOs) and other specialists. | Code: Reorganization of Services  The intervention involved an analysis of data from the Western Cape Department of Health’s routinely collected database. Data extracted included: Persal (human resources database), Sinjani (database of aggregated performance data of hospitals), ETR.net (electronic tuberculosis [TB] registry) and the National Population Census 2011. Data were linked using district and subdistrict names. | Data from the Western Cape Department of Health’s routinely collected database was used to measure outcomes. Primary health care utilisation was used to assess health system performance. Cervical smears, TB treatment, couple-year protection rate, early antenatal booking and under 1-year immunisation coverage were used as clinical processes indicators. Maternal, perinatal, and under-5 mortality were used to assess clinical outcomes. Dependent variables were calculated using the Western Cape Department of Health’s Annual Performance Plan 2016–2017 definitions and formulas. |
| Farmer, 2011, United Kingdom | Cohort | To evaluate the impact and contribution made by PAs to delivering effective health care in National Health Service (NHS) Scotland. | Code: Extending Scope of Practice - Non-FP  The medical home team consisted of a primary care physician and office staff, the child and the family, a nurse practitioner (NP), and a parent consultant (a paid family member of a child with special health care needs). The intervention focused on providing care coordination, information about resources and services, emotional support and encouragement, and empowerment for families to advocate for their children. To accomplish these goals, every participant received from the NP a set of basic services that included a home visit to conduct a comprehensive assessment of medical and nonmedical needs of the child and family members, a personalized letter that described health, educational, and community resources for meeting these needs; an individualized written health plan for the child; assistance in developing short-term family goals; and at least one follow-up to discuss progress toward goals and to problem solve about any barriers to needed care. In addition, the parent consultant assisted with family-to-family support as needed. The intervention lasted six months from the first home visit, with periodic follow-ups in the second 6 months. | Outcomes were assessed at the first visit and after completion of the program. Outcomes included family demographics, child health services (including current health service needs, health service utilization and parental satisfaction with services), family functioning, child functioning, and program acceptability. |
| Human Immunodeficiency Virus | | | | |
| Yapa, 2020, South Africa | Cluster RCT | To establish the effects of continuous quality improvement (CQI) on the quality of antenatal HIV care in primary care clinics in rural South Africa. | Code: Healthcare Provider Training + Training of Lay Community Members  The intervention focused on developing the capacity of local antenatal care (ANC) health workers in study clinics and aimed to improve the implementation of the national eMTCT guidelines. The intervention was delivered by trained CQI mentors and included standard CQI tools(process maps, fishbone diagrams, run charts, Plan-Do-Study-Act [PDSA] cycles, and action learning sessions). CQI mentors worked with health workers, including nurses and HIV lay counsellors. The mentors flexibly used the standard CQI tools tailored to local clinic needs. Health workers were direct intervention recipients, whereas pregnant women attending ANC were the ultimate beneficiaries. | Quality of care in HIV-related ANC: (i) viral load monitoring among pregnant women living with HIV and (ii) repeat HIV testing among pregnant women not living with HIV. |
| Lifson, 2017, Ethiopia | Uncontrolled before/after | To evaluate implementing a community support worker program to reduce loss of follow-up for HIV-infected patients in rural Ethiopia. | Code: Extending Scope of Practice - Non-FP  Patients were assigned 1 of 13 trained community health support workers (CHSWs) who were HIV positive and from the same neighbourhood/village. The CHSWs provided HIV and health education, counselling/social support, and facilitated communication with the HIV clinics. | The primary outcomes were changes in client knowledge, perceived social support, self-reported quality of life (QOL), and feelings of internalized stigma over one year. Additionally, clinical and patient care outcomes were abstracted from clinic records at baseline, six months and 12 months. Variables measured included dates of clinic visits, transfers of care, cause of death (if applicable), and CD4 counts. |
| Moeckli, 2017, United States | Retrospective Cohort | To evaluate 3 HIV Extension for Community Health Outcomes (ECHO) programs in the Veterans Health Administration, focusing on uptake by primary care clinics and veterans. | Code: Telehealth or Virtual Appointments  Community Health Outcomes (ECHO) model uses telemedicine to create regional communities of practice that link speciality clinics with primary care providers (PCPs) in outlying areas. ECHO is a provider-level telemedicine model; patients receive all care from nearby PCPs, who interact with distant specialist teams using videoconferencing and shared medical records. | Administrative data were used to assess program uptake, including adoption (i.e., the proportion of primary care clinics participating) and reach (i.e., the proportion of eligible veterans participating). Interviews were conducted with HIV specialists, primary care providers (PCPs), and administrators engaged in HIV ECHO, and interview transcripts were analyzed to identify factors that influenced program adoption and reach. |
| Mueller, 2017, Swaziland | Prospective Cohort | The objective of this study was to assess the feasibility of tuberculin skin test-based 36 months isoniazid preventive therapy (IPT) strategy in HIV-infected patients in programmatic conditions in 2 rural clinics of the Shiselweni region in Swaziland to inform policymakers in the country and similar settings. | Code: Reorganization of Services + Increasing Staff Resources  People living with HIV (PLHIV) eligible for IPT followed in 2 rural nurse-led primary care clinics. Nurses provided HIV-TB care, and expert clients (PLHIV trained to provide patient support education and counselling) assisted the nurses in managing patient files, clinic registers, and adherence support. Medical doctors visited the clinic 1/week or biweekly as needed to attend to complicated cases and offer technical support. | Data was collected by the clinic staff on regular patient files during the routine follow-up visits before extraction on study-specific case report forms by a study nurse. The occurrence of adverse drug reactions was recorded in the patient file by the clinic nurse. |
| Sam-Agudu, 2017, Nigeria | Prospective Cohort | To evaluate the impact of structured vs unstructured peer support on postpartum retention and viral load suppression among rural Nigerian women. | Code: Training of Lay Community Members + Implementing a New Service  HIV-positive pregnant women were enrolled at primary health care centres with structured mentor mother (MM) support. Mentor mothers received training on peer counselling, had structured schedules for client visits, supervision of their work, standardized documentation, performance evaluations, and retraining as appropriate. | The primary outcomes were retention of patients and viral suppression. Retention was defined by clinic attendance during the first six months postpartum. Participants with $3 of 6 expected monthly visits were considered retained. Women with a 6-month postpartum plasma viral load of 20 copies/ mL were suppressed. |
| Iwuji, 2016, South Africa | Cluster RCT | To investigate whether antiretroviral therapy (ART) can reduce new HIV infections in the general population, piloted the trial to check whether starting HIV-positive individuals on antiretroviral therapy directly after diagnosis is feasible and acceptable. | Code: Reorganization of Services  In the intervention clusters, HIV-positive individuals were informed during home-based HIV testing that they would be provided ART irrespective of CD4 count and clinical stage. In the control clusters, HIV-positive individuals were informed that ART would be offered according to the criteria of the national South African guidelines or coinfection with multidrug-resistant or extensively drug-resistant tuberculosis. | Outcome measures included a study questionnaire, a blood sample from a finger prick and a rapid HIV test using a serial HIV testing algorithm. |
| Meng, 2006, China | Uncontrolled before/after | This pilot study, conducted in rural Jilin Province in northeastern China, was to develop and test an infrastructure for delivering highly active antiretroviral therapy (HAART) that might apply to other such areas across the country. | Code: Patient Education + Reorganization of Services  The intervention consisted of a five-tier administrative structure involving the provincial, city, district, town, and village levels and an embedded three-tier team delivery structure involving the district, town, and village levels. Village doctors provided ongoing education on medicine taking and compliance issues, surveillance for drug side effects, transmission and prevention knowledge, non-discrimination, and the daily needs of a person with AIDS. | Participants’ CD4 cell count was taken at the beginning of the therapy and 3-month intervals by the Jilin Province Center for Disease Control. The ability to return to work was used as a quality of life measurement to gauge therapy success. |
| HIV & Hypertension | | | | |
| Ameh, 2020, South Africa | Cross-sectional | To determine the quality of care provided in the integrated chronic disease model, describe patients’ and operational managers’ perceptions of the model, and assess the effectiveness of the integrated model in controlling CD4 counts and blood pressure of patients from 2011 to 2013. | Code: Reorganization of Services  The government implemented an integrated chronic disease management (ICDM) model in health facilities as a pilot programme for adult populations. | The primary outcomes included patients’ and operational managers’ satisfaction using Avedis Donabedian’s quality of medical care framework. Additionally, CD4 counts & blood pressure was also measured. |
| Hypertension | | | | |
| Stallings, 2021, United States | Uncontrolled before/after | To describe clinical pharmacy services provided in a rural North Carolina primary care clinic and assess the impact of these services on systolic and diastolic blood pressures in patients with uncontrolled hypertension. | Code: Extending Scope of Practice + Patient Education/Navigation  The pharmacist conducts independent patient and blood pressure assessments during hypertension visits and provides extensive patient education. The pharmacist may also prescribe and adjust antihypertensive medications, order routine laboratory tests (e.g., basic metabolic panel following initiation of a thiazide diuretic), directly consult with primary care providers, order additional pertinent tests such as an electrocardiogram (EKG) or chest X-ray, and refer patients to cardiologists when deemed necessary. | Change in systolic and diastolic blood pressures from baseline, percentage of patients with blood pressure reductions, percentage at The Eight Joint National Committee goal blood pressure, percentage at care gap closure defined as obtaining a blood pressure <140/90 mm Hg, and time to reach care gap closure. |
| Zheng, 2018, United States | Uncontrolled before/after | To evaluate the effectiveness of hypertension management intervention and analyse the factors associated with blood pressure reduction within China’s primary healthcare system. | Code: Healthcare Provider Training  All the participating GPs, nurses and public health doctors received training before the intervention. The training included hypertension screening, treatment and management, follow-up visits and health education. All patients in the intervention received one physical examination and at least four face-to-face follow-ups with GPs in the year. Further interventions were offered depending on the outcome of the follow-ups. | Blood pressure was the primary outcome variable measured by a mercury sphygmomanometer. |
| Wu, 2018, United States | Prospective Cohort | To examine the effects of a health-coaching intervention on medication adherence and blood pressure (BP) and to explore whether changes in medication adherence over time were associated with changes in BP in patients with hypertension. | Code: Implementing a New Service  It involved a practice‐based phone health‐coaching component embedded in a larger quality improvement intervention involving strategies to systematically improve care processes for hypertension management in rural primary care. The intervention was led by two local health coaches who received training in using the scripted health‐coaching materials. Each participant received: (1) a home BP monitor and instructions on accurate home BP measurement technique and (2) 12 monthly coaching calls that included a review of home BP measurements, medications, challenges to medication adherence, and self‐determined goal setting. | Medication adherence (measured by patient self-report) and BP were collected at clinic visits at baseline, 6, 12, 18, and 24 months. |
| Cene, 2017, United States | Prospective Cohort | To evaluate the effect of a multicomponent practice-based quality improvement (QI) intervention on lowering systolic BP (SBP) in a cohort of patients selected on having uncontrolled HTN as measured at their practice. We also aimed to determine whether the intervention would have a differential effect by race. | Code: Patient Education/Navigation + Reorganization of Services  The Promoting Action on Research Implementation in Health Services (PARIHS) model was adapted to conceptualize practice change for practice-level intervention. The practices and the community-based health coach were taught communication and behaviour change strategies based on social cognitive theory, the transtheoretical model of behaviour change, and motivational interviewing. The telephone coaching part of the intervention was informed by components of Bosworth’s Take Care of Your Blood Pressure (TCBY) study, which included telephone case management. The coaches delivered the intervention via 12 monthly phone calls. The coaches helped participants set care goals, reviewed appropriate BP measurement techniques and discussed BP target values on each phone call. Participants were also mailed educational materials to correspond to the topics of each upcoming phone coaching session. | Data was collected at baseline and 6-, 12-, 18-, and 24 months post-enrollment. All outcomes measure assessment was done by trained research staff using measurements obtained at research study visits, not part of routine clinical care. |
| Halladay, 2017, United States | Prospective Cohort | Multilevel quality improvement intervention could differentially improve Systolic Blood Pressure (SBP) more so in patients with low vs. higher health literacy. | Code: Patient Education/Navigation + Healthcare Provider Training  The intervention had components at the practice level and patient level. The practice-level intervention involved activities to impact all patients with hypertension, like educational meetings on health literacy and hypertension treatment, instruction on goal setting with patients, and motivational interviewing techniques. The patient level involved activities for patients with uncontrolled hypertension, including phone coaching, materials mailed to patients' homes, and using a home BP monitor and BP log book at home. | The primary outcome was BP. Health literacy was measured using the Short-Test of Functional Health Literacy in Adults. |
| Yan, 2017, Zambia | RCT | To present data on novel retrospectively generated process and outcome indicators for hypertension management, informed by those from Western countries but adapted to the Zambian primary care clinics enrolled in the Better Health Outcomes through Mentoring and Assessment (BHOMA) study. | Code: Healthcare Provider Training + Increasing Staff Resources  BHOMA is a 5-year, randomized stepped-wedge trial of improved clinical service delivery underway in 46 rural government clinics. BHOMA aims to improve primary healthcare through standardized protocols for common visits, onsite electronic medical records (EMR), and ongoing mentoring to improve key indicators. District based Quality Improvement teams of 5–8 specially trained nurses and clinical officers make regular visits to participating clinics for mentoring meetings, record review and general support. Community volunteers were also trained to perform non-specialist tasks as clinic support workers, including patient file management and vital signs. We used an explanatory sequential design by conducting a quantitative analysis, which was then explained through a qualitative follow-up component. | Routine clinical data on patient demographics, BP measurements, diagnosis, physical exam, lab tests, medication prescription and visit dates were extracted from the EMR. For the qualitative component, facility audits focusing on available equipment, medication stocks and staffing were conducted as part of routine visits by trained members of the BHOMA Quality Improvement teams at half of the clinics, once during March-April. Semi-structured in-depth interviews with health care providers and a representative from the central medication distribution agency in Zambia, Medical Stores Limited, were also completed during these routine visits. |
| Miao, 2016, China | RCT | To estimate whether or not the integrative strategy of health services delivery can improve Health-Related Quality of Life (HRQoL) among rural hypertensive patients. | Code: Patient Education/Navigation + Reorganization of Services  Physicians from village clinics, town hospitals and county hospitals worked collaboratively to deliver multidisciplinary health services for the intervention group, while physicians in the control group provided services without cooperation. The intervention package mainly included integrating preventive-curative services delivery and cooperation among village-town-county physicians. Village clinic physicians provided goal-oriented health education mainly focused on smoking cessation, moderate drinking, a light and healthy diet, regular exercise and mastering skills to alleviate negative emotions according to individualized lifestyle and health education schemes made by township hospital physicians. Township hospital physicians acted as integrators for preventive-curative services and further patient movement within the county-township health system. Township hospital physicians made individualized lifestyle and health education schemes according to the patient reports from the village clinic physicians and advised village clinic physicians on adjusting the health education carried out in previous stages per patients’ health condition and disease progress. County hospital physicians played a leadership role in the delivery of multi-disciplinary care. They guided the adjustment of the proactive treatment scheme made by township hospital physicians according to their clinical reports. | The Medical Outcome Study Short-Form 36-Item Health Survey (SF-36 Scale) was used to assess HRQoL, and the differences in scores between the intervention group and the control group represented the effect of the intervention. Town hospitals reported blood pressure. |
| Yun, 2015, United States | Uncontrolled before/after | The study aimed to evaluate the effects of a pilot project to establish linkages among community-based organizations, healthcare providers, and public healthcare systems. | Code: Patient Education/Navigation  The county health department partnered with community-based organizations to help the members control blood pressure. The health educator or registered nurse from the county health department went to the community-based organizations to provide group education for the organization’s members about the cause, classification, prevention and management of high blood pressure. | Data were collected using several instruments, including the attendance sheet, presentation planning form, pre- and post-questionnaires, and the follow-up documentation form. Information on the program operation was collected through monthly reports, quarterly conference calls, and periodic site visits. |
| Clark, 2012, United Kingdom | Prospective Cohort | To determine whether a difference in systolic blood pressure readings between arms can predict a reduced event-free survival after ten years. | Code: Reorganization of Services  The current study evaluated participants receiving treatment for hypertension in primary care. The intervention consisted of bilateral blood pressure measurements recorded at three successive surgery attendances. | Pairs of blood pressure readings were measured using a standard mercury sphygmomanometer. The primary outcome measure consisted of cardiovascular events (e.g., myocardial infarction or a new diagnosis of angina) and deaths from all causes during a median follow-up of 9.8 years. |
| Tobe, 2006, Canada | RCT | The study aimed to assess whether a community-based treatment strategy implemented by home care nurses would effectively control hypertension in First Nations people with existing hypertension and type 2 diabetes. | Code: Extending Scope of Practice - Non-FP  Researchers compared two community-based strategies for controlling hypertension in First Nations people with existing hypertension and diabetes. In the intervention group, a home care nurse followed a predefined pharmacologic antihypertensive therapy treatment algorithm. In the control group, treatment decisions were made by each subject’s primary care physician. | The primary outcome measure was the difference between the two groups in the change in systolic blood pressure after 12 months. Secondary outcome measures were the change in diastolic blood pressure over time, the change in urine albumin status and the incidence of adverse events. |
| Lin, 2004, Taiwan | Controlled before/after | The study aimed to evaluate a 3.5-year hypertension control programme of combined high-risk and mass strategies in a rural community with limited medical resources and its impact on hypertension control and stroke mortality. | Code: Patient Education/Navigation + Training of Lay Community Members  The high-risk strategy intervention consisted of trained volunteers responsible for follow-up and education. During bimonthly home visits, the volunteers measured blood pressure and body weight. They conveyed information about hypertension, regular blood pressure checks, weight reduction, reduction of alcohol intake, cessation of smoking, and moderate isotonic physical exercise. The mass strategy intervention included trained volunteers with one year of practice and on-job training and acquired more experience, and displayed better skills in working with hypertensives. Hypertension education classes and lectures were delivered by invited outside experts for the target population at every village four times a year. Most programme components were designed to provide cardiovascular disease risk reduction information. | Outcome measures included the same structured questionnaire used at the baseline visit administered during repeat visits to record changes in related knowledge and behaviour. A repeat blood screening for the subjects with hypertension was conducted during the 1-year intervention. A comparison of the stroke mortality between the intervention community and a neighbourhood reference community before and after the intervention programme was performed. |
| Carter, 1997, United States | Controlled before/after | To evaluate pharmacy care for hypertension patients and the impact on blood pressure control, quality of life, patient satisfaction, quality of care, and cost of care were evaluated. | Code: Extending Scope of Practice - Non-FP + Healthcare Provider Training  Researchers designed a program to train community pharmacists to provide hypertension monitoring and direct consultation to physicians and nurses. The pharmacists were provided extensive skill development, including problem-solving workshops and practice experience with the principal investigator in a Veterans Affairs pharmacist-managed hypertension clinic in Chicago. They were given extensive reading materials concerning hypertension, including national guidelines. | Outcome measures include surveys and blood pressure measurements using the American Heart Association Standards and Guidelines. Both study groups completed the Short Form 36 at baseline and six months. Patients also received a questionnaire at the end of the study to assess their overall satisfaction with care delivery and pharmacy services. |
| Keyserling, 1997, United States | Cluster RCT | To assess the effectiveness of a cholesterol-lowering intervention designed to facilitate the management of hypercholesterolemia by primary care clinicians | Code: Patient Education/Navigation + Coordination/Referral Pathways  Trained clinicians administered a structured assessment and treatment program (Food for Heart Program) consisting of a brief dietary assessment and three 5- to 10-minute dietary counselling sessions given by the primary care clinician, referrals to a local dietitian if the low-density lipoprotein cholesterol (LDL-C) remains elevated at the 4-month follow-up, and prompt the clinician to consider lipid-lowering medication based on the LDL-C at the 7-month follow-up. | Phlebotomy procedures and blood collection were completed to measure cholesterol and triglyceride levels. The primary outcome measure consisted of changes in total and LDL-C at 4-month follow-up and averaged over a 1-year follow-up period (4-, 7-, and 12-month follow-up). |
| Integrated Care | | | | |
| Peterson, 2017, United States | Uncontrolled before/after | To demonstrate the value of an integrated behavioural health program within primary care practices and to evaluate the financial viability of an integrated care model in a rural setting. | Code: Extending Scope of Practice - Non-FP  Three Behavioral Health Providers (BHPs), the clinic physicians, and the administration received training in an integrated care model. The BHPs were expected to work in a brief solution-focused model, using warm hand-offs with their schedules built for 20-minute appointments. It was expected that patients would average three sessions per episode of care. All behavioural health providers completed a 40-hr “boot camp” training to ensure sufficient provider training and understanding of the model. The training included a comprehensive overview of the behavioural health model outlined by Robinson and Reiter (2007), program Startup, interdisciplinary communication and working effectively as part of a multidisciplinary team, and evidence-based interventions for common behavioural health and medical presentations within a primary care setting. Following the training, providers received in-clinic support during their initial two weeks of start-up and continued to attend monthly consultation meetings emphasizing model adherence and best practices. | A reduction in medical claims measured effectiveness. Healthcare utilization included primary care visits, inpatient speciality care, outpatient speciality care, emergency care, ambulance use, and lab and facility charges. This data was pulled before the intervention and for the six months following the patients’ respective episodes of care with the BHP. This data was also used to assess the reach of the intervention. |
| Farmer, 2005, United States | Uncontrolled before/after | To evaluate the feasibility and impact of a medical home demonstration project in a rural population. | Code: Coordination/Referral Pathways  The medical home team consisted of a primary care physician and office staff, the child and the family, a nurse practitioner (NP), and a parent consultant (a paid family member of a child with special health care needs). The intervention focused on providing care coordination, information about resources and services, emotional support and encouragement, and empowerment for families to advocate for their children. To accomplish these goals, every participant received from the NP a set of basic services that included a home visit to conduct a comprehensive assessment of medical and nonmedical needs of the child and family members, a personalized letter that described health, educational, and community resources for meeting these needs; an individualized written health plan for the child; assistance in developing short-term family goals; and at least one follow-up to discuss progress toward goals and to problem solve about any barriers to needed care. In addition, the parent consultant assisted with family-to-family support as needed. The intervention lasted six months from the first home visit, with periodic follow-ups in the second 6 months. | Outcomes were assessed at the first visit and after completion of the program. Outcomes included family demographics, child health services (including current health service needs, health service utilization and parental satisfaction with services), family functioning, child functioning, and program acceptability. |
| Lifestyle Improvement | | | | |
| Schepens Niemiec, 2021, United States | Uncontrolled before/after | To evaluate patients’ long-term health-related outcomes after lifestyle intervention. | Code: Implementing a New Service  A culturally tailored, 4-month lifestyle intervention co-led by occupational therapy practitioners and Latino community health workers that features telehealth and in-home sessions covering topics such as healthy eating and navigating health care. | Self-reported and physiological outcomes: symptom–well-being (primary), stress, sleep disturbance, social satisfaction, physical activity, patient activation, blood pressure, and weight. |
| Limbs Disability | | | | |
| Deng, 2021, China | RCT | To evaluate the effect of a transitional care program on health outcomes in individuals with limbs disability. | Code: Reorganization of Services  Intervention participants received ongoing rehabilitation at the specialized care centre and home through a multidisciplinary team. | Primary outcomes were quality of life (short form health survey), functional performance (modified Barthel index), and burden of illness (caregiver strain index). |
| Maternal & Child Health | | | | |
| Larson, 2020, Tanzania | Cluster RCT | To examine the success of a maternal healthcare quality improvement intervention in improving quality. | Code: Healthcare Provider Training + Coordination/Referral Pathways + Increasing Staff Resources  The quality improvement intervention involved In-service training on basic emergency obstetric and newborn care, access to referral facilities, mentorship and supportive supervision by an obstetrician and infrastructure support in the form of necessary equipment, supplies and medication. | Knowledge of obstetric and newborn care (measured by a test and clinical vignettes), obstetric services provided (recorded by the registers at the facilities), quality of care (measured via self-report by patients and providers), satisfaction (self-report from patients), and maternal health consisting of lack of anemia (hemoglobin level is 12.0 g/dl or above for nonpregnant women and 11.0 g/dl or above for pregnant women), lack of hypertension(average systolic reading less than 140 mm Hg and average diastolic reading less than 90 mm Hg), distribution of EQ-5D (EuroQol Group, Rotterdam, Netherlands) and distribution of mid-upper arm circumference (all biomarkers were collected via a household survey). |
| Jabbari Beyrami, 2019, Iran | Interrupted Time Series | To assess the impact of the Family Physician programme on maternal and child health (MCH) indicators in 20 years in Iran. | Code: Financial Incentive + Increasing Staff Resources + Expanded Scope of Practice FP  This intervention comprised a medical, maternal and childcare services package and aimed to make healthcare services affordable through a new insurance plan. The team structure of health centres was expanded to include additional staff, such as a general physician, midwives and a pharmaceutical technician. One family physician was settled in each village. A new insurance plan (Rural Health Insurance) was also scaled up to cover the whole Iranian rural population. GP services were increased to round-the-clock service, with increased GPs available in each area. GP salaries and services were increased to include special antenatal and postnatal care, laboratory tests (including measurement of haemoglobin (Hb) levels to monitor iron supplementation plan) and routine ultrasounds to detect fetus abnormality. The FP teams also started providing maternity care for all women in rural areas. | The indicators were grouped into three categories: structure (mother’s age, education, occupation and gravidity), process (number of antenatal care visits (ACVs), laboratory tests, ultrasounds and natural vaginal deliveries (NVDs)) and outcomes (maternal mortality ratio (MMR), neonatal mortality rate (NMR), birth weight (BW), history of abortion and stillbirth, and haemoglobin level (Hb)). |
| Medication (Prescribing or medication safety) | | | | |
| Falamic, 2018, Croatia | RCT | To determine the effect of a pharmacist-delivered education and medication review on time in therapeutic range (TTR) in elderly patients on warfarin. | Code: Extending Scope of Practice - Non-FP  45-minute education session on warfarin treatment. Participants were then given a dosing scheme for their warfarin therapy and followed up monthly for six months. | TTR was measured using the Rosendaal method. |
| Goldstein, 2018, United States | Uncontrolled before/after | To determine the effect of an educational tool on the frequency of contraceptive counselling by resident physicians. | Code: Patient Education/Navigation  Resident physicians were instructed to complete a checklist of female patients presenting to the clinic to determine their eligibility for counselling. If they were eligible, contraceptive counselling was provided by the resident using an educational table outlining the available contraception forms. | The rate of contraceptive counselling was reported with an ICD-10 code. |
| Vandenberg, 2018, United States | Controlled before/after | To describe the methods used to implement the expanded IMPROVE model and report its effectiveness results, including dissemination feasibility to rural settings. | Code: Healthcare Provider Training + Audit and Feedback  Adapted a successful medication management model, Integrated Management and Polypharmacy Review of Vulnerable Elders (IMPROVE), from an urban geriatric speciality clinic to rural community-based clinics that deliver primary care. The goals were to promote prescribing quality and safety for older adults, including reducing prescribing of potentially inappropriate medications (PIMs). They augmented the original model, which involved a pharmacist-led, one-on-one medication review with high-risk older veterans, to provide rural primary care providers (PCPs) and pharmacists with educational outreach through academic detailing and tools to support safe geriatric prescribing practices, as well as individual audit and feedback on prescribing practice and confidential peer benchmarking. | They analyzed prescribing data in aggregate over three time periods: baseline, intervention, and after implementation. The following quality improvement measures were calculated: PIM incidence—number of new PIM prescriptions divided by all encounters (opportunities) that a provider had with veterans aged 65 and older; PIM prevalence—number of encounters with veterans currently taking at least 1 PIM divided by all encounters; Multiple PIM prevalence—number of encounters with veterans taking two or more PIMs divided by all encounters. They also tracked using the IMPROVE templated note in the electronic medical record to quantify individual medication management visits with a clinical pharmacist in each CBOC. They collected demographic information on veterans seen and medications stopped or started at the visit with the clinical pharmacist. They contacted each IMPROVE participant (PCP, pharmacist, individuals seen by pharmacist) for a telephone interview to assess their satisfaction with the IMPROVE intervention. |
| Omotayo, 2017, Kenya | Cluster RCT | To determine the effect of a simpler medication regimen with lower daily dosages on medication adherence and mean calcium intake compared to the standard higher dose regimen. | Code: Reorganization of Services  The medication regimen included 500mg Ca/d twice daily with 200 IU cholecalciferol, a calcium pill, and an iron-folic acid pill taken separately. | Medication adherence and mean calcium daily intake were recorded at 4- and 8-week follow-ups. |
| Rebello, 2017, United States | Controlled before/after | To evaluate the efficacy of the Rural Pharmacological Intervention in Late Life (PILL) program. | Code: Reorganization of Services + Decision Support  A quality improvement initiative in which a Boston-based pharmacist provided post-discharge telepharmacy care to veterans. Using an automated screening tool, they identified 100 veterans aged 65 and older who had an acute care admission to VA medical centres and were at risk of problems with medication management. The PILL pharmacist called patients the week after hospital discharge to reconcile medications, assess adherence, and identify potentially inappropriate drugs. The pharmacist worked with each veteran’s family and providers to resolve problems and increase support. | To determine whether the intervention decreased acute care admissions, rehospitalizations, or deaths, we matched one unique control to each PILL patient by age, hospital location, length of stay, admitting service, and reason for admission. |
| Frail, 2016, United States | Retrospective Cohort | To describe an innovative community pharmacy-based pilot program using technology to support care transitions for patients living in rural areas. | Code: Extending Scope of Practice - Non-FP  Community pharmacists worked with patients immediately following discharge to reconcile their medications and make recommendations to optimize therapy. The pharmacy packaged their new medication regimen in precise, individual dose adherence packaging. Medications were delivered by a staff driver to the patient's home within 72 hours of discharge. Patients consulted with the pharmacist by videoconference using a computer tablet device. Patients received telephone follow-ups shortly before their medication supply was to run out and additionally as needed individually. | Self-reported hospital readmissions were collected at 30 and 180 days after enrollment. Patient satisfaction data were also collected at 30 and 180 days using a tool modified from the 5-item Transition Measure (15-item Care Transitions Measure). |
| Albert, 2011, United States | Uncontrolled before/after | To determine the effect of a community-based overdose prevention program on overdose death rates in a county with high overdose rates. | Code: Implementing a New Service + Training of Lay Community Members  A community-based overdose prevention program with five components: community activation and coalition building; monitoring and surveillance data; prevention of overdoses; use of rescue medication for reversing overdoses by community members; and evaluation of project components. | Overdose death rate and number of people who received a prescription for the substance implicated in their overdose. |
| Fiss, 2010, Germany | Prospective Cohort | To determine the feasibility of community-based home medication reviews (HMR) and establishing a health professional network to identify drug-related problems (DRP) in the domicile of elderly patients. | Code: Extending Scope of Practice - Non-FP  The AGnES-concept (general practitioner-relieving, community-based, e-health assisted, systemic intervention) is based on delegating original physician activities to qualified AGnES-practice assistants. The AGnES intervention was subdivided into distinct modules. Each assistant was prepared for the extended responsibilities in an individually tailored curriculum. Training modules included, e.g. documentation, communication, age-related diseases, pharmacotherapy and drug-related problems, and geriatric assessment. An important task of the AGnES-practice assistants was to conduct home medication reviews (HMR). The questionnaire focussed on drug storage conditions, expiration, adherence, adverse drug reactions (ADR), and potential drug-drug interactions. | Outcomes included: the number of drugs taken, adverse drug reactions, drug-drug interactions (DDI) and factors related to drug safety and administrative aspects of the intervention (age, gender, non-adherence, use of medication plan etc.) |
| Graffen, 2004, Australia | RCT | To determine the effect of a medication review on quality of life and medication-related hospital admissions in community-dwelling elderly patients managed by their GP. | Code: Reorganization of Services  A pharmacist performed medication reviews and provided recommendations to the GP. If the GP agreed with the recommendations, the medication changes were implemented, and a follow-up was performed six months later. | The 36-Item Short-Form Health Survey was used to measure quality of life. The number of medication-related hospitalizations was self-reported by patients at six months. |
| Taylor, 2003 United States | RCT | To determine the effect of pharmacist-provided pharmaceutical care on adverse drug-related events. | Code: Extending Scope of Practice - Non-FP  Pharmacists provided pharmaceutical care to patients 2-3 times per week in addition to usual care. Pharmaceutical care included individualized patient education and review of medical records to determine drug compliance and complications. | Number of ED visits and hospitalizations obtained from review of medical records and patient interviews. Medication compliance was self-reported by patients, and the 36-Item Short-Form Health Survey was used to assess quality of life and mental health. Prescribing appropriateness was determined with the Medication Appropriateness Index. Clinical endpoints such as hypertension, diabetes, anticoagulation, and dyslipidemia were also reviewed. |
| Mental Health | | | | |
| Ndayizigiye, 2022, Lesotho | Controlled before/after | To evaluate a novel early childhood development (ECD) programme integrated into the primary healthcare system. | Code: Patient Education/Navigation + Implementing a New Service  The hybrid care delivery model focuses on parent coaching for knowledge about child development, practising contingent interaction with the child, parent social support and encouragement. | Developmental outcomes were evaluated using the Extended Ages and Stages Questionnaire (EASQ) and caregiving practices using two measure sets (i.e., UNICEF Multiple Indicator Cluster Survey (MICS), Parent Ladder) and compared to the control group. |
| Renn, 2022, United States | Uncontrolled before/after | To examine the differences in depression outcomes between younger and older adults in a large-scale implementation demonstration of the collaborative care model (CoCM). | Code: Implementing a New Service + Coordination/Referral Pathways  All participants received depression treatment via collaborative care management, which enhances usual primary care and efficiently uses specialists using a behavioural healthcare manager and a psychiatric consultant to support primary care providers. Clinics were followed for up to 27 months. | The Patient Health Questionnaire (PHQ-9) assessed depressive symptoms at baseline (enrollment) and most follow-up contacts. The primary treatment outcome was a change between a  patient's first and last recorded PHQ-9 scores. |
| Srinivasan, 2022, India | Cluster RCT | To test whether depression outcomes differ among recipients of a collaborative care model compared to enhanced standard treatment in patients with co-morbid chronic medical conditions. | Code: Provider Education/Training + Reorganization of Service + Coordination/Referral Pathways  The collaborative care model consisted of a clinic-based and a community-based component. The clinic-based component involved training the primary health care team in treating mental health and chronic disease according to the collaborative stepped care model. Responsibilities included a nurse acting as the case manager, pharmacists trained in identifying common adverse side effects of antidepressants and educating participants about them, weekly phone calls between PHC physician and study psychiatrist, referrals to district psychiatrist for suicidal patients, and referrals for abnormal lab values. The community component involved healthy living groups to develop and maintain skills for improved mental and physical health that can be incorporated into their lifestyle: 12 weekly sessions, followed by nine monthly sessions. | The primary outcome was the individual-level Patient Health Questionnaire Depression Scale score over time. |
| Poudiougou, 2021, Mali | Uncontrolled before/after | To provide a mental health training intervention to rural general practitioners (GPs), to organize community awareness activities, and to evaluate the impact on mental health knowledge and through the number of new patients diagnosed with mental disorders and managed by these general practitioners. | Code: Healthcare Provider Training  Two face-to-face group training workshops followed by individual follow-up supervisions were conducted to train GPs on diagnosing and managing mental health conditions. Some GPs were also trained to train other healthcare workers. | Change in knowledge was measured through pre-and post-training questionnaires. Other outcomes, such as the number of patients diagnosed with mental disorders and managed by the newly trained GPs, were reported by each GP every month. |
| Zittleman, 2021, Mali | Uncontrolled before/after | To evaluate the impact of Opioid Use Disorder (OUD) training on implementing and delivering OUD treatment with buprenorphine in primary care practices of rural Colorado. | Code: Healthcare Provider Training  A practice-focused intervention that provides training to improve awareness, adoption, and use of buprenorphine treatment for OUD. | Practice-reported and population-level data from the Prescription Drug Monitoring Program were obtained to describe changes in treatment delivery after training. |
| Bellesheim, 2020, United States | Cohort | To evaluate the utility of Maintenance of Certification Quality Improvement training designed to improve developmental (e.g. autism spectrum disorder) screening rates in underserved, rural primary care practices. | Code: Healthcare Provider Training  The intervention included training primary care providers using Extension for Community Healthcare Outcomes (ECHO) Autism, a virtual learning network. The network provided them access to a pediatrician specializing in development and behaviour, a child and adolescent psychiatrist, a clinical psychologist, a social worker, a dietician, and a parent of a child with an autism spectrum disorder. | The primary outcomes were screening rates. |
| Bowen, 2020, United States | Cohort | To compare the effects of implementing a collaborative care program on depression outcomes in rural Native American and Alaska Native (AI/AN) patients with White patients and patients of other ethnic backgrounds at three clinics. | Code: Reorganization of Service  The intervention was designed to integrate treatment for common mental health disorders (e.g. depression, anxiety) into primary care settings using principles of chronic disease management using an interdisciplinary team (primary care provider, a psychiatric consultant, and a behavioural health care manager). The intervention provided clinic staff with training on universal screening for depression, evidence-based treatment to target, and the use of behavioural health care. | Primary outcomes were depression response (i.e., reduction in symptom severity as measured by PHQ-9) and depression remission (measured by HEDIS) measured over two years. |
| LeCloux, 2020, United States | Uncontrolled before/after | To evaluate the feasibility and impact of a suicide risk screening program in a rural West Virginia primary care practice. | Code: Screening  Patients were asked to participate in electronic suicide risk screening using the Ask Suicide-Screening Questions tool. Patients who screened positive were assessed with the Ask Suicide-Screening Questions Brief Suicide Safety Assessment. | Screening impact was evaluated quasi-experimentally by comparing electronic medical record documentation of suicide risk screening, assessment, and risk determination in practice patients before and after implementing the screening program. |
| Powers, 2020, United States | Cohort | To evaluate depression treatment outcomes, clinical processes of care, and primary care provider experiences for collaborative care implementation in 8 rural clinics treating low-income patients. | Code: Reorganization of Services  Rural clinics were funded and trained to adapt a collaborative care model to their local contexts. Key components, such as ensuring key components necessary for clinical effectiveness, such as first follow-up contact within four weeks of treatment initiation and psychiatric consultation, had to be maintained. They were encouraged to use a registry to track treatment outcomes and cue proactive treatment changes. | Outcomes were change in depression severity from baseline to last measurement among patients treated, variation in clinical outcomes (depression response and remission) among patients receiving care by modelling the effects of patient demographic characteristics, treatment exposure, and treatment intensity while adjusting for clinic variation on change in depression symptoms, and PCP satisfaction. |
| Smith, 2020, Rwanda | Uncontrolled before/after | To assess the implementation reach, fidelity, and clinical outcomes at health centres supported by The Mentoring and Enhanced Supervision at Health  Centres (MESH MH) program. | Code: Implementing a New Service  Nurses and community health care workers were trained to deliver the following services: complete mental health assessment, including medical and psychosocial assessment; psychoeducation to service users and families; psychosocial interventions; medication management regular monitoring and follow up (weekly to monthly); referral to community-based support for adherence promotion and follow-up management as needed; and triage and referral to specialist mental health care for acute or complex needs as needed. | Primary outcome assessments at baseline, 2 and 6 months included symptoms and functioning, measured by the General Health Questionnaire (GHQ-12) and the World Health Organization Disability Assessment Scale (WHO-DAS Brief), respectively. |
| Buist, 2019, Scotland | Uncontrolled before/after | To evaluate a pilot mental health intervention using pharmacists to provide psychopharmacological care for depression and anxiety from the patient and provider perspective. | Code: Extended Scope of Practice – Non-FP  A 12-month pilot was implemented in two general practices in remote and rural Scotland. General practitioners referred patients to specialist mental health pharmacists as independent prescribers for evidence-based psychopharmacological interventions. As part of the service, all patients completed Patient Health Questionnaire (PHQ-9) and Generalised Anxiety Disorder (GAD-7) rating scales at their first and last appointments. | The primary outcome was the reduction in anxiety and depression symptoms as measured by Patient Health Questionnaire (PHQ-9) and Generalised Anxiety Disorder (GAD-7) rating scales. |
| Hanlon, 2019, Ethiopia | Cohort | To evaluate the safety and impact of a district-level plan for task-shared mental health care on six and 12-month clinical and social outcomes of people with severe mental illness in rural southern Ethiopia. | Code: Provider Education/Training  An interdisciplinary intervention was implemented at the health system level, primary healthcare facility and community. The intervention focused on training primary healthcare (PHC) workers to assess community referrals, diagnose severe mental illness and initiate treatment with independent research diagnostic assessments by psychiatric nurses. | Primary outcomes included clinical symptom severity (measured by the Brief Psychiatric Rating Scale), disability (measured by the World Health Organisation Disability Assessment Schedule), experience of discrimination (measured using the ‘unfair treatment’ subscale of the discrimination and stigma scale), restraint (measured by self-report of whether the person had been ‘restrained, chained or confined’ in the months preceding), alcohol use disorder (measured by Alcohol Use Disorder Identification Test, depression (measured by Patient Health Questionnaire), suicide attempts in the past three months (measured via Mini International Neuropsychiatric Interview). |
| Logan, 2019, United States | Uncontrolled before/after | To describe the role of behavioural health in an addiction medicine program integrated in a primary care clinic and to evaluate retention, substance use, and mental health symptoms for patients in a rural underserved community. | Code: Reorganization of Services  The addiction medicine team included: three waivered family practice physician prescribers, three licensed psychologists, a licensed clinical social worker [LCSW], a behavioural health case manager [BHCM], and a clinical pharmacist, all with extensive training in motivational interviewing (MI) and SUD treatment. The psychologists and LCSW (behavioural health providers [BHPs]) conducted psychological and substance use assessments, developed treatment plans, and provided counselling services for substance-related disorders and other mental conditions. Ancillary support services were available through various West Hawaii Community Health Center departments, including psychiatry, insurance eligibility and prior authorizations, pharmacy services, internal and external referrals, nursing, overall care coordination, and scheduling/reception services. | Patient Health Questionnaire-9 and Generalized Anxiety Disorder Scale-7 measured depression and anxiety. Urine samples were collected to measure substance misuse. All data was collected at each visit. |
| Parchman, 2019, United States | Interrupted Time Series | To examine the effect of implementing these six opioid management strategies (six building blocks) on opioid-prescribing practices. | Code: Reorganization of Services  The Six Building Blocks refers to 6 elements for a team-based redesign of opioid medication management within smaller primary care practice settings as follows: (1) provide leadership support, (2) revise and align clinic policies, use patient agreements, and define workflows, (3) track the patient population, (4) implement planned, patient-centred visits, (5) identify resources for complex patients, and (6) measure success. Each clinic completed a self-assessment and education on the six building blocks for opioid management redesign. Each clinic designated an opioid improvement team and had access to resources and education to assist with management redesign. Clinics were offered an online registry to track their patients on long-term opioid therapy. They were reimbursed to help pay for a medical assistant to track and enter patient data. | Outcomes were a monthly trend in the proportion of patients undergoing long-term opioid therapy prescribed a ≥100 morphine equivalent dose (MED) of opioids daily and the total number of patients receiving an opioid prescription. |
| Wong, 2019, United States | Retrospective Cohort | To compare depression outcomes in rural vs urban settings while accounting for the known differences between Collaborative Care Management (CCM) and usual care within an extensive Southeast Minnesota primary care practice. | Code: Reorganization of Services  Each clinic provided Collaborative Care Management (CCM) for depression within a patient-centred medical home practice. CCM supports the primary care clinician in caring for depressed patients with an electronic registry, specially trained registered nurse care managers, the use of established clinical guidelines, and an integrated behavioural health team consisting of psychologists, social workers, clinical nurse specialists, and a consulting psychiatrist. The care managers, social workers, and psychologists were in the clinic to maintain face-to-face, telephone, or electronic contact with CCM patients. The team followed practice guidelines in consultation with a psychiatrist to assist the physician in caring for depressed patients by providing counselling, medication advice, and frequent follow-up. | Two outcome variables were constructed; Remission, defined by a six-month Patient Health Questionnaire (PHQ-9) < 5 and Persistent Depressive Symptoms (PDS), defined by a six-month PHQ-9 ≥ 10. |
| Collins, 2018,  Ireland | Controlled before/after | To evaluate a computerized cognitive behavioural therapy (cCBT) programme, MindWise (2.0), for adults  attending Irish primary care psychology services. | Code: Implementing a New Service  MindWise is a computerized cognitive behavioural therapy (cCBT) programme that assists adults with mild depression or  anxiety to manage their mood by introducing them to CBT theory and techniques. | The primary outcome measures were the total scores on the Patient Health Questionnaire9 (PHQ-9) and the Generalised Anxiety Disorder-7 (GAD-7). The Work and Social  Adjustment Scale (WSAS) was utilized as a secondary outcome measure. |
| Dragomani, 2018, United States | Cross-sectional | To examine the impact of routine pharmacogenomic testing on the incidence of ADHD  medication side effects and changes in behavioural symptoms. | Code: Implementing a New Service  Children attending a clinic for ADHD appointments were asked to fill out an assessment form each time they visited to assist nurses in recording the child's symptoms and medication side effects into EMR to improve documentation. Pharmacogenomic testing was offered to all children before being prescribed medication to assist in choosing the correct medication and dose for the child. | ADHD symptoms were measured through the Vanderbilt Attention-deficit/Hyperactivity Assessment Rating Scale. Quality of life was measured using cross-sectional data from 40 KINDL Quality-of-Life Questionnaires (parent-respondent). Quality of care was assessed through parent interviews. |
| Maconick, 2018, South Africa | Prospective Cohort | To develop and evaluate a locally delivered, long-term, in-service training programme to facilitate mental health care in primary care. | Code: Extending Scope of Practice - Non-FP  The in-service training programme was delivered weekly 1-h sessions by local psychiatry staff to 20 primary care nurses at the clinic over five months. The training was based on the “Practical Approach to Care Kit” guidelines that teach primary care workers first-line treatments for depression, substance misuse, psychosis and dementia. | Data were collected before the training began and again at four months. A questionnaire was administered to all participants examining competence at diagnosing and treating common mental disorders and some core skills for mental health, such as mental state examination. Additionally, the number of referrals was collected for one month before training and at the 4-month evaluation mark after training was complete. Interviews were conducted to assess potential barriers to using the training at work daily. |
| Mallow, 2018, United States | Uncontrolled before/after | To present the initial effectiveness of a web-based system of sensors and mobile devices designed to overcome the known health determinant of access to care for rural, chronically ill patients using technology. | Code: Telehealth or Virtual Care + Patient Education/Navigation  The mI SMART tool is a web-based application usable with any mobile device and operating system. The patient side of mI SMART combines synchronous and asynchronous patient education, reminders to perform self-management, a record of self-monitoring readings with automated and personal responses from clinicians, notifications of medications due, a secure asynchronous messaging portal, video conferencing for routine appointments, access to laboratory results, and research survey links. Each participant was given Bluetooth-enabled self-monitoring devices such as a scale, glucometer, blood pressure cuff, a tablet, and three months of internet data service. They used the application to access all their healthcare needs instead of attending the primary care clinic for 12 weeks. A nurse practitioner-led the program. | The intervention lasted for 12 weeks. Blood glucose, blood pressure, and weight were collected using the provided Bluetooth devices and means were evaluated before and after the intervention. |
| Rojas, 2018, Chile | Uncontrolled before/after | This study reports the feasibility, acceptability, and effectiveness of a remote collaborative care program for patients with depression living in rural areas of Chile. | Code: Coordination/Referral Pathways  The intervention involves following specific algorithms for treating depression. This involved any primary care clinician referring suspected cases of depression to an on-site physician who can diagnose and initiate treatment. Severe cases were referred to specialized mental health services, and mild to moderate cases may receive a combination of antidepressants, psychosocial interventions, and monitoring visits in primary care, according to severity. | Baseline and follow-up assessments 3 and 6 months after baseline evaluation were carried out via telephone. Treatment adherence to antidepressants during the previous three months was assessed using a questionnaire, and user satisfaction was measured through a depression treatment satisfaction scale. Depressive symptom scores were assessed using the Beck Depression Inventory (BDI-I), and health-related quality of life was recorded by the 36-item Short Form Survey (SF-36). |
| Hansel, 2017, United States | Uncontrolled before/after | To describe the impact of newly implemented pediatric mental and behavioural health models in health clinics and demonstrate the sustainability and effectiveness of services provided. | Code: Patient Education/Navigation  Brief behavioural health services included parental education, medication management, stress management, empowerment, and psychodynamic interventions. | The primary outcome measures were the Pediatric Symptom Checklist (measures behavioural and emotional problems) and the Parenting Stress Index. |
| Maulik, 2017, India | Uncontrolled before/after | To evaluate the feasibility and acceptability of an intervention for identifying and treating common mental health disorders. | Code: Extending Scope of Practice - Non-FP  A mental health services delivery model that leveraged technology and task sharing to facilitate identification and treatment (including following treatment guidelines) of common mental disorders (CMDs) such as stress, depression, anxiety and suicide risk in rural areas of Andhra Pradesh, India. The intervention was delivered by lay village health workers (Accredited Social Health Activists – ASHAs) and primary care doctors. An anti-stigma campaign using multi-media approaches was conducted across the villages at the project's outset. | The primary outcome was an evaluation of pre- and post-intervention mental health service utilization. Additionally, depression and anxiety scores of those who tested positive for a CMD at baseline were measured and compared post-intervention. Trained interviewers conducted a baseline survey. The survey enquired about sociodemographic details, stressors, social networks, CMD, history of mental disorders and their treatment, family history of mental disorders, and perceptions about stigma related to mental health. Process evaluation of the project was done using focus group discussions and in-depth interviews with key stakeholders. |
| Whealin, 2017, United States | Prospective Cohort | The aims were to determine the feasibility of a cultural adaptation of a cognitive-behavioural clinical intervention for use by rural Pacific Island veterans. | Code: Patient Education/Navigation  The " Koa " intervention is a multisession family psychoeducational program that integrates selected Pacific Islander values, beliefs, and healing traditions with an empirically based mainstream U.S. intervention. | Outcomes included relationship quality, relationship satisfaction, caregiver burnout, and patient satisfaction/acceptability. All outcomes were measured via standardized scales or interviews. The fidelity of the intervention's content and process was also monitored to ensure the outcomes could be replicated. |
| Kozlowski, 2015, United States | Uncontrolled before/after | To assess the feasibility and effects of a brief seven-session cognitive behavioural skills-building intervention, Creating Opportunities for Personal Empowerment (COPE), in children with anxiety. | Code: Patient Education/Navigation  COPE was administered by a trained pediatric nurse practitioner (PNP) using two manuals – a user manual for the PNP and a manual for the child/teen. The individual COPE sessions lasted 30 minutes and followed a specific topic built from session to session. The core concept, a subset of CBT techniques, was presented to the child and then reinforced through games, interactive activities, and real-life application of these concepts. Homework/skills-building activities were assigned to reinforce the topic further. | Postintervention outcomes included assessment of anxiety symptom reduction, cognitive skills knowledge, level of functioning, and post-program satisfaction. Cognitive-behavioural skills learned through the COPE program were assessed through a 15-question content quiz given before and after all seven sessions were complete. Both the children and parents responded to open-ended questions about the helpfulness of the program, precisely what they learned, the length of the program, the therapy location, and if they would recommend this program to another child. |
| Pyne A, 2015, United States | RCT | To examine the cost-effectiveness of on-site practice-based collaborative care (PBCC) versus off-site telemedicine-based collaborative care (TBCC) for depression in federally qualified health centres (FQHCs). | Code: Decision Support + Healthcare Provider Training + New Staff Resources  Practice-based collaborative care involved on-site PC providers and on-site nurse depression care managers (DCMs). Each clinic location employed a half-time DCM funded by the study. All DCMs received one day of training in depression care management, a care manager training manual, and access to a Web-based decision support system. Depending on patient preference encounters with a DCM were conducted face-to-face or by telephone. The initial encounter with the DCM included PHQ-9 symptom monitoring, education and self-management behavioural activation, barrier assessment and resolution, and establishing self-management goals, such as planning physical, rewarding, and social activities. Follow-up encounters included monitoring symptoms with the PHQ-9, medication adherence, side effects, and engagement in planned self-management activities. | Primary effectiveness outcomes for cost-effectiveness analysis were depression-free days and quality-adjusted life years (QALYs). Depression outcomes of Depression treatment response, remission, and severity were compared between groups using the Symptom Checklist. |
| Pyne B, 2015, United States | RCT | To examine the cost-effectiveness of on-site practice-based collaborative care (PBCC) versus off-site telemedicine-based collaborative care (TBCC) for depression in federally qualified health centres (FQHCs). | Code: Decision Support + Healthcare Provider Training + Increasing Staff Resources + Telemedicine or Virtual Care + Coordination/Referral Pathways  TBCC involved five types of providers: on-site PC providers and off-site DCM (a registered nurse), clinical pharmacist (Pharm.D.), psychologist (PhD), and psychiatrist (M.D.). All encounters between DCMs and patients were conducted by telephone and followed the protocol described above. The DCM met weekly with the psychiatrist to discuss clinical cases and treatment recommendations. These notes were faxed to the FQHC for implementation by the PC providers. If the patient did not respond to the initial antidepressant, the off-site pharmacist conducted a medication history and provided medication management recommendations as needed. A psychiatry consultation via interactive video was scheduled if the patient did not respond to two trials. Patients had access to cognitive-behavioral therapy delivered via interactive video at any time. | Primary effectiveness outcomes for cost-effectiveness analysis were depression-free days and quality-adjusted life years (QALYs). Depression outcomes of Depression treatment response, remission, and severity were compared between groups using the Symptom Checklist. |
| Kessler, 2012, United States | Cross-sectional | This study aims to examine treatment startup rates in 2 collaborative care settings: a rural family medicine ofﬁce and a suburban internal medicine ofﬁce. | Code: Coordination/Referral Pathways  A collaborative care pilot tracked mental health referrals and treatment initiation as part of quality improvement efforts. After the initial pilot in a family medicine practice, a second intervention was started in an internal medicine practice, providing the opportunity to report rates of attendance at initial mental health visits at a second site. In both practices, referrals for mental health services are made within the practice. The collaborative care model focuses on the clinical, operational, and ﬁnancial elements of care and planning, and ongoing quality improvement is built into implementation and operation. Model elements are drawn from best practices identiﬁed in the ﬁeld. | Outcomes included the number of patients referred by the provider, the number who scheduled an appointment and the number who attended the ﬁrst appointment. |
| Armstrong, 2010, Australia | Cross-sectional | To explore the views of women screened for postnatal depression at maternal and child health checks for many years in one rural shire. | Code: Coordination/Referral Pathways  The intervention included maternal and child health nurses screening all women for postnatal depression using the Edinburgh Postnatal Depression Scale. | They used a postal survey to ask if they received screening for postnatal depression, their experience, and if they had any referrals due to being screened. Additionally, women were invited to be interviewed about their experiences, including physical and emotional health during their first postnatal year, who conducted the screening, results of screening, whether they considered themselves to be depressed, any formal diagnoses, sources of help they used, and any suggestions to improve the program. |
| Cullum, 2007, United Kingdom | RCT | To compare liaison psychiatric nursing with usual medical care in managing older medical inpatients who screened positive for depression. | Code: Reorganization of Services  A liaison psychiatric nurse (LPN) implemented the intervention, assessed participants, formulated a care plan for their depression, ensured its implementation through liaison with appropriate agencies, and monitored participants’ mood and response to treatment for up to 12 weeks. Participants in the control group received the usual treatment from the hospital and primary care staff. | The primary outcomes at follow-up were the presence of ICD-10-defined depressive disorder and a change in Geriatric Depression Scale-15 score from baseline. Secondary outcomes were differences in quality-adjusted life weeks (QALWs) and patient satisfaction ratings. Outcomes were compared between the intervention group and the control (usual care) group. |
| Adams, 2006, United States | Cohort | To explore whether a depression disease management program has a comparable impact on clinical outcomes over two years in patients treated in rural and urban primary care practices and whether the impact is mediated by receiving evidence-based care (antidepressant medication and speciality care counselling). | Code: Healthcare Provider Training  The research team provided primary care professionals with brief training in depression disease management to improve the detection and management of major depression. Primary care nurses provided systematically scheduled care management over two years to depressed patients in the registry. Care management consisted of defined patient education, systematic treatment adequacy/compliance monitoring, and clinical improvement. | The effect of care management on mental health status was measured using the mental component summary (MCS), a combination of 4 subscales from the SF-36 18,19, totalling 14 items. |
| Oyama, 2006, Japan | Controlled before/after | To evaluate outcomes of a community-based program to prevent suicide among the elderly. | Code: Screening + Patient Education/Navigation  A community-based program including depression screening with follow-up using its community resources of primary care and public health nursing. They included health education components such as mental health workshops that older people attended. | The primary outcome was changes in the incidence rates (IR) of suicide among the elderly aged 65 and over, with stratification for age and gender, before and after the program implementation. |
| Bergus, 2005, United States | RCT | This study investigated whether screening for depressive symptoms improves outcomes for depressed patients seen in rural fee-for-service primary care offices. | Code: Screening  Depression screening was based on the 9-item PHQ-9. Patients who indicated either low mood or anhedonia for more than half the days of the last two weeks (on the questionnaire) were invited to enrol and randomized to the control or intervention group. Medical providers of patients in the control group were not informed of the PHQ-9 results. Providers of patients in the intervention group were asked to review the completed PHQ-9 and therefore were aware of the patient’s self-reported severity of depression symptoms. All providers were educated about the PHQ-9 but were not otherwise influenced to change their practices. | The PHQ-9 scores were assessed by telephone at 4, 10, and 24 weeks after the index visit. |
| Campbell, 2005, Australia | Prospective Cohort | The primary purpose was to evaluate whether participants seen within the local service delivery model would have different clinical outcomes from those seen within the ‘traditional’ mental health service delivery model. | Code: Increasing Staff Resources  Involved employing a mental health worker to provide counselling, lead regular tutorials and case conferences with the GPs, liaise with other local services and address community groups. | Changes in symptomatology were assessed using the SCL-90R summary scales, and changes in quality of life were assessed using the EuroQOL. |
| Malcolm, 2002, Australia | Uncontrolled before/after | To evaluate the effects of employing a mental health worker to provide additional mental health services on access to treatment and mental health outcomes (i.e., symptoms and functioning). | Code: Extending Scope of Practice - Non-FP  Involved employing a mental health worker to provide counselling, educate patients and the public about mental illness, improve the skills of local health workers, liaise with other counselling agencies, and undertake research into mental health in the area. | Questionnaires were given to patients seen and returned anonymously. Questions covered demographics, referral source, reason for presentation, the effect of stigma on help-seeking behaviour and changes in symptoms. Other questionnaires were used to help assess the project's impact on patients presenting with non-mental health problems, relatives of those with mental illness, GPs and the local community. Figures were obtained from the State Coroner’s Office for suicides in the project area. The number of known suicides in the area in the three years before the commencement of the project and the three years during which the project was noted. Additionally, numbers of new patients, age-range and geographical distribution, and diagnoses made by the mental health worker were recorded. |
| Smith, 2000, United States | RCT | To assess a guideline-based intervention's impact on depression care provided in rural vs. urban primary care settings. | Code: Healthcare Provider Training  The QUEST (Quality Enhancement by Strategic Teaming) intervention was to increase the proportion of depressed primary care patients who completed a guideline-concordant course of antidepressant pharmacotherapy and psychotherapy in the six months following the index visit at which they were identified as depressed. All participating physicians and nurses in enhanced care practices participated in a series of four academic, detailed telephone conference calls over two months to become systematically engaged with the AHCPR depression treatment guidelines. The training also included interactive roleplaying exercises and written tests to retain information. Additionally, the research team’s clinical social worker ensured nurse fidelity to the intervention by reviewing weekly patient treatment logs completed by the nurses and by completing a series of telephone calls with each nurse to provide feedback and support as they adapted to their new roles. | Data were collected via structured telephone interviews during the week following the index visit (baseline) and again six months following the index visit. Pharmacotherapy was measured via self-report from the patient on taking antidepressant medication at minimum therapeutic guideline-concordant daily doses for at least three months between baseline and six-month follow-up. |
| Minor Illness | | | | |
| Shum, 2000, United Kingdom | RCT | To assess the acceptability and safety of a minor illness service led by practice nurses in general practice. | Code: Extending Scope of Practice - Non-FP  Patients were assigned to treatment by either a specially trained nurse or a general practitioner. Patients seen by a nurse were referred to a general practitioner when appropriate. | The consultation satisfaction questionnaire measured the general satisfaction of the patients. Other outcome measures included the length of the consultation, number of prescriptions written, rates of referral to general practitioners, patient’s reported health status, patient’s anticipated behaviour in seeking health care in future, and number of patients who returned to the surgery, visits to accident and emergency, and out of hours calls to doctors. |
| Non-Communicable Disease | | | | |
| Ameh, 2017, South Africa | Interrupted Time Series | To assess the effectiveness of the integrated chronic disease management (ICDM) model in controlling patients’ CD4 counts and blood pressure in primary health care (PHC) facilities in the Bushbuckridge municipality, South Africa. | Code: Reorganization of Services  A controlled interrupted time-series study was conducted using the data from patient’s clinical records collected multiple times before and after the ICDM model was initiated in PHC facilities in Bushbuckridge. Patients >/= 18 years were recruited by proportionate sampling from the pilot (n = 435) and comparing (n = 443) PHC facilities from 2011 to 2013. Health outcomes for patients were retrieved from facility records for 30 months. | Key outcome variables included viral load, CD4 count and BP values during the 30 months of data collection. |
| Osteoarthritis | | | | |
| Nelson, 2014, United States | Markov Modeling | To evaluate the cost-effectiveness of training rural primary care providers to perform knee injections in community-based outpatient clinics (CBOCs). | Code: Healthcare Provider Training  Developed a decision-analysis model to compare costs and outcomes of hypothetical patients seen by rural providers who are trained to perform knee injections versus those patients seen by providers who are not trained. | The primary outcomes were costs and effectiveness. The costs from the perspective of the VA included the direct medical costs to perform the knee injection, the mini-residency training program, and travel reimbursement given to qualified patients. All costs were converted to 2011 US dollars. The effectiveness outcome was quality-adjusted life years. |
| Osteoporosis | | | | |
| Nelson, 2014, United States | Markov Modeling | To determine the cost-effectiveness analysis of training rural providers to identify and treat osteoporosis. | Code: Healthcare Provider Training  We constructed a Markov microsimulation model to compare the costs and outcomes of a hypothetical cohort of veterans seen by rural providers. Parameter estimates were derived from previously published studies, and we conducted one-way and probabilistic sensitivity analyses on the parameter inputs. | Outcomes included the impact of training on patient life years, quality-adjusted life years (QALYs), treatment rates, fracture incidence, and costs from the Department of Veterans Affairs perspective. |
| Pain Management | | | | |
| Stack, 2020, United States | Controlled before/after | To evaluate the effects of implementing a comprehensive opioid reduction protocol on overall opioid prescribing among patients with chronic non-cancer pain in rural family medicine clinics. | Code: Implementing a New Service  Opioid reduction protocol consisted of several interventions primarily guided by the CDC guidelines: risk assessment and mitigation; patient education via an 8-week psychoeducational group; checking urine drug screens and statewide prescription drug monitoring program; treating psychiatric comorbidities; maximizing nonpharmacological and nonopioid pharmacological treatments; slow taper of opioids at 10% of original dose per month, aiming to get all patients under 90 MME and most under 50 MME; and finally assessment for opioid use disorder with referral for medication-assisted therapy if indicated. | Compared mean daily milligrams morphine equivalent prescribed to patients before and after protocol implementation. |
| Mehl-Madrona, 2016, United States | Controlled before/after | To compare evidence-based complementary and alternative medicine to conventional care for chronic pain. | Code: Patient Education/Navigation  Patients attended group medical visits (GMVs), provided education about non-pharmacological methods for pain management and taught mindfulness techniques, movement, guided imagery, relaxation training, yoga, qigong, and t’ai chi. | Patients attending GMV were compared to patients receiving conventional care. Outcome measures consisted of (1) change in opiate dosage in morphine equivalents, (2) change in scores on the My Medical Outcome Profile, 2nd version (MYMOP2), and (3) change in visual analogue pain ratings. The MYMOP2 is a patient-centred, problem-specific outcome measure, and evidence suggests that it is a valuable and sensitive measure of change in perceived symptoms and quality of life. |
| Mashari, 2012, Canada | Cross-sectional | To examine the effectiveness of epidural steroid injections (ESIs) on chronic low back pain in those with neurologic symptoms. | Code: Reorganization of Services  Reviewed the use of ESI for the two most common types of chronic low back pain in those with neurologic symptoms: lumbar disc herniation (LDH) and lumbar spinal stenosis (LSS). Conducted a retrospective chart review of all patients who underwent ESI between Jan. 1, 2005, and Feb. 25, 2010, at a rural hospital in northwestern Ontario. | The primary outcomes included the total number of ESIs administered and the reported improvement, worsening or lack of change after the patient’s first injection. |
| Robinson, 2012, United Kingdom | Cross-sectional | To determine if GP-led acupuncture improves health outcomes and decreases medication use in patients of rural general practice. | Code: Extending Scope of Practice FP  10- to 20-minute acupuncture treatment sessions. | A short questionnaire was provided to patients that assessed their subjective outcome of the presenting condition after treatment, the degree of improvement of the condition, the number of analgesic/anti-inflammatory medications taken following treatment, and whether the patient believed referral to physiotherapy or a hospital specialist was avoided due to the treatment. |
| Burnham, 2010, Canada | Uncontrolled before/after | To determine the effect of a multidisciplinary primary care program on pain and disability in chronic pain patients. | Code: Reorganization of Services  Based on the assessment, patients were assessed and received one of the following four methods of care: consultation only, interventional spinal care, supervised medication management, or full multidisciplinary management. | Pain intensity was measured with a numerical rating scale, and disability was measured with the Pain Interference Questionnaire. |
| Reynolds, 2009, United States | Controlled before/after | To implement and evaluate in a rural setting a predischarge patient education intervention focused on self-pain management of uncomplicated postsurgical patients after being discharged home. | Code: Patient Education/Navigation  The intervention involved patients randomly assigned to the education intervention or control group. The intervention group involved a 10 min session with a research assistant to review a two-page patient education brochure on pain management. | A knowledge and experience questionnaire was completed before and after the study. Additionally, other outcomes included the Brief Pain Inventory, demographics, and a patient pain log used to evaluate the effectiveness of the intervention. |
| Ahles, 2006, United States | RCT | To determine the effect of tailored information, and tailored information in addition to telephone consultation for patients with persistent pain with or without psychosocial problems. | Code: Patient Education/Navigation  Patients with persistent pain received tailored information based on their computer-based survey responses. Patients with persistent pain and psychosocial problems received calls from a nurse who assessed the patient’s pain and psychosocial problems and provided self-management strategies and written materials in addition to the tailored information. | Disability was measured with the Functional Interference Estimate, and other health outcomes were measured with the 36-Item Short Form Survey. Healthcare utilization was measured from self-reported physician visits, days at the hospital, and days at home. |
| Ahles, 2001, United States | RCT | To determine the effect of mailed education materials, education materials, and telephone consultations for patients with persistent pain with or without psychosocial problems. | Code: Patient Education/Navigation  Patients with persistent pain received education materials via mail. Patients with persistent pain and psychosocial problems received calls from a nurse who provided problem-solving and self-management strategies in addition to the education materials. | Disability was measured with the Functional Interference Scale, and other health outcomes were measured with the 36-Item Short Form Survey. Knowledge and satisfaction were measured with questionnaires developed by the research team. |
| Palliative Care | | | | |
| Bonsignore, 2018, United States | Cohort | To evaluate a telehealth system's feasibility, usability, and acceptability in palliative care. | Code: Telehealth or Virtual Care  Involved using the TapCloud remote patient monitoring application and videoconferencing system. The system allowed patients to access an interdisciplinary team. It addressed the needs of patients with life-limiting illnesses through symptom management, prognostication, psychosocial care, advance care planning (ACP), spiritual care, caregiver support, patient/ family education, and coordination with community-based resources. Physicians and community health workers were trained in using the program to monitor patients' health status and connected with interdisciplinary teams to assist them. | The primary outcomes included patient symptom burden and improvement, hospice transitions, and advanced directives. Qualitative data on satisfaction with the program was collected from a subpopulation of telehealth patients, caregivers, and providers using interviews. The self-efficacy of community healthcare workers and primary care providers was also measured using surveys. All survey measures were reported on a scale of 1 to 7, where “1” indicated “none or no skill” and “7” indicated “expert, teach others. |
| vandeMortel, 2019, Australia | Controlled before/after | To examine the feasibility of using a GP registrar (GPR) to facilitate communication among palliative care specialists, consumers and GPs, and provide risk assessment, care planning and continuity of care. | Code: Implementing a New Service  Intervention group participants received the GPR service, which involved liaison among the patient, family, General Practitioner, specialist palliative care team and community nurses. Specified risk assessment, care planning and continuity of care were provided. The GPRs conducted the initial patient assessment and case conference with the medical and nursing teams and the family to develop the care plan. They also conducted a 3-month follow-up (for stable patients) or re-assessed and updated the plan if the patient deteriorated. | Hospital admissions per 100  patient-days, bed-days per 100 patient-days and proportion of deaths at home. |
| Mitchell, 2016, Australia | Uncontrolled before/after | To evaluate a pilot on whether nurse practitioner (NP)-led care, including clinical care plans negotiated with involved health professionals, including the general practitioner (GP), ± patient and carer, through a single multidisciplinary case conference (SMCC), could influence patient and health system outcomes. | Code: Extending Scope of Practice - Non-FP  The intervention was NP-led and involved the NP performing an assessment followed by an SMCC as soon as possible after referral. Then a clinical care plan was developed to create management plans for current and anticipated problems and to dictate who was responsible for each action. | Eligible patients had baseline, one and 3-month patient-reported assessment of function, quality of life, depression and carer stress, and a clinical record audit. Interviews with key service providers assessed the utility and feasibility of the service. |
| Pediatric Outcomes | | | | |
| Naderimagham, 2017, Iran | Retrospective Cohort | The present study aimed to examine the impacts of the implementation of the family physician program and rural insurance program on neonatal (NMR), infant (IMR), and under-5-year (U5MR) mortality rates in rural areas of Iran. | Code: Increasing Staff Resources  Three segmented regression models were built to evaluate the program's effects on NMR, IMR, and U5MR. Several independent variables were entered into the models, including the annual incremental effect of the program (variable of interest), time effect, behaves density, the effect of the family physician and rural insurance programs, as well as socioeconomic variables, including years of schooling, wealth index, sex ratio, and logarithmic scales of rural population size in each area. Data were gathered from secondary sources and other studies. | Three response variables were recognized in the segmented regression model of this study, the neonatal mortality rate (NMR), the infant mortality rate (IMR), and the under-5 mortality rate (U5MR). Several independent variables were entered into the model, including the annual incremental effect of the rural family physician program and social protection scheme (the primary variable of interest), time effect, behaviour density, and effect of the program. |
| Physical Activity | | | | |
| Sherman, 2007, United States | Uncontrolled before/after | This study assessed the effect of a brief primary care-based walking intervention in rural women. | Code: Patient Education/Navigation  The enrolled subjects were given a pedometer and exercise videotape and provided exercise counselling at intake and four-time points over six months. | The week one pedometer step counts were compared with step counts at 6-month follow-up. |
| Elley, 2003, New Zealand | Cluster RCT | To assess the long-term effectiveness of the "green prescription" programme, a clinician-based initiative in general practice provides counselling on physical activity. | Code: Healthcare Provider Training + Patient Education/Navigation  All patients aged 40-79 who attended the participating practices for five days received a screening form based on currently recommended physical activity levels to establish eligibility. The "Green Prescription" intervention included primary care clinicians offering four hours of training in using motivational interviewing techniques to give advice on physical activity and the green prescription. Patients identified as "less active" through screening at the reception desk who agree to participate receive a prompt card, stating their stage of change, from the researcher to give to the general practitioner during consultation. In the consultation, the primary care professional discusses increasing physical activity and decides on appropriate goals with the patient. Specific advice about exercise or community groups is provided, and if appropriate Quarterly newsletters from the sports foundations about physical activity initiatives in the community and motivational material are sent to participants. Other mailed materials, such as specific exercise programmes, are sent to interested participants. | Primary outcome measures were evaluated at baseline and 12-month follow-up and included change in total expenditure of energy and leisure time expenditure of energy, cardiovascular risk (as assessed by systolic and diastolic blood pressure and coronary heart disease risk), and quality of life. Measures of potential harm included change in injuries and falls in the previous month and admission to hospital in the previous year. |
| Population Health | | | | |
| Aghajanian, 2007, Iran | Uncontrolled before/after | To examine the impact of the rural health development programme on the health of a rural population. | Code: Reorganization of Services  Established a solid network of rural health centres (RHCs) and smaller centres called “health houses” to deliver low-technology primary health care through indigenous health care providers at the village level. Additionally, they utilized mobile teams of a physician, a health technician, and a community health worker to fill any gaps in care. Services provided included: census, maintaining health records, public health education, family health care, pregnancy-related care, family planning services, immunizations, disease control services and environmental health activities. | Pre-intervention outcomes and post-intervention outcomes categorized outcomes recorded. Pre-intervention outcomes included the status of health care in 1979 (indicators included crude death rate (per 1000 people), infant mortality rate (per 1000 live births), child mortality rate (1-4 years per 1000 live births), life expectancy at birth (years), domestic facilities (%). Post-intervention outcomes included the impact of the programme (indicators included neonatal mortality (per 1000 live births), infant mortality rate, child mortality rate, children with one illness during the last two weeks, mothers treating their children's sickness correctly, children under five years have diarrhea, children with diarrhea who used oral rehydration therapy, children with respiratory infection correctly treated, women w0ith no prenatal care, pregnant women who received the vaccination, received postnatal care at least once, received postnatal care at least twice, ever-married women who had knowledge about and ever-married women who used oral contraceptives, condoms, IUDs, injectable contraceptives, vasectomy, tubectomy, safe period, withdrawal). |
| Post Abortion Care | | | | |
| Kiemtore, 2017, Burkina Faso | Uncontrolled before/after | To evaluate the results of an intervention by the Societé de Gynécologues et Obstétriciens du Burkina (SOGOB) to improve post-abortion care (PAC) in rural areas of Burkina Faso. The project’s objectives were to improve the skills of service providers in family planning and PAC, equip health facilities with manual vacuum aspiration (MVA) devices and provide them with misoprostol, improve the use of misoprostol in the management of incomplete abortion, and improve the uptake of post-abortion contraception. | Code: Healthcare Provider Training  The present comparative study examined practices in PAC in the year before and the year after the application of a health intervention by SOGOB in 45 rural health facilities at the primary level of the Burkina Faso health system. SOGOB provided training in health care and equipment for PAC to 56 health facilities, which were then followed up for at least one year. The Burkina Faso health system is pyramidal. The primary level of the pyramid consists of two sublevels: health and social promotion centres (HSPCs) and medical ¬centers with a surgical unit (MCSUs). The second level of the pyramid is represented by regional hospital centres (RHCs). At the top of the pyramid, the tertiary level comprises university hospital centres (UHCs). Members of SOGOB who were highly skilled in abortion care provided training to 58 midwives, 27 nurses, and five general practitioners. Three 12-day training sessions were organized at a teaching hospital. Thirty healthcare providers were trained per session; the first six days covered theoretical and practical training in modern methods of contraception (intrauterine devices, implants, injectable contraceptives, pills, and condoms). Four days were reserved for theoretical and practical training on PAC (MVA and use of misoprostol). The final two days were used for clarification of abortion values and transformation training of the attitudes of healthcare providers to improve access to PAC. | Outcome measures included comparing clinical and patient statistics from the 45 rural health facilities before and after the intervention. The variables evaluated included the presence of functional MVA devices, percentage of incomplete abortions managed by MVA, percentage of incomplete abortions managed by misoprostol, percentage of incomplete abortions treated by inappropriate methods, post-treatment complication rate, and percentage of women receiving a modern method of post-abortion family planning. |
| Pregnancy-Related | | | | |
| Gatakaa, 2019, Kenya | Cross-sectional | To investigate the effect of autonomous community medical centres on maternal and newborn care access indicators over two years of initial implementation. | Code: Implementing a New Service  A network of 16 autonomous community medical centres/kiosks were implemented to help facilitate access to maternal and newborn care services. | The primary outcomes on the setting of the last delivery, the number of visits to a health facility, and examination of the newborn post-delivery in the previous pregnancy were assessed via a questionnaire. |
| Preventative Care | | | | |
| SchepensNiemiec, 2018  United States | Uncontrolled before/after | To determine the feasibility and efficacy of a culturally tailored lifestyle intervention, ¡Vivir Mi Vida! (Live My Life!). | Code: Patient Education/Navigation + Telehealth or Virtual Care  This intervention was designed to improve the health and well-being of high-risk late middle-aged Latino adults and to be implemented in a rural primary care system. The first intervention session included a home visit with community health workers (CHW) and a telemedicine occupational therapy (OT) consultation. During this session, the participant created a personalized health action plan (HAP), which was revisited at subsequent sessions. CHWs led weekly visits (including seven one-on-one home sessions, two group sessions held at local community facilities, and two telephone check-ins) over 16 weeks. In addition, the OT provided two 20-min telephone consultations to discuss individually experienced wellness facilitators and troubleshoot barriers to health-related goals. CHWs received a structured manual, a picture-based flip-over booklet, and demonstration tools. Participants were given health-related materials and tools throughout the program, such as a Garmin Vivo Fit activity monitor and a measuring cup. Overarching modular topics included healthy eating and physical activity, healthcare navigation, chronic disease management, and mental well-being. | The primary patient-centred outcomes consisted of sub-scores on the Measure Yourself Medical Outcome Profile 2 (MYMOP2; Paterson, 1996). Secondary outcomes ranged from lifestyle factors (e.g., physical activity engagement) to cardiometabolic measures (e.g., cholesterol level). Comorbidity information was collected via self-report and from AVCC electronic medical records. Feasibility was assessed using a mixed-methods process evaluation that considered the viewpoints of participants, stakeholders, and the study team. |
| Gray, 2010, Canada | RCT | To evaluate the cost-effectiveness of Anticipatory and Preventive Team Care (APTCare). | Code: Coordination/Referral Pathways  At-risk patients were randomly assigned to receive usual care from their family physicians or APTCare from a collaborative team. APTCare consisted of being assigned to the care of 1 of 3 NPs, the pharmacist, and their usual family physicians. Care provided by the NPs and pharmacists was delivered almost exclusively in the patients’ homes, while patients continued to see their family physicians in the office. The main objective of the intervention was to ensure evidence-based disease management and solid social support for patients. Additionally, some patients received a telehealth system in the home for remote monitoring of clinical parameters (e.g., blood pressure, weight, glucose levels, and blood oxygen levels) by the NPs. | Cost-effectiveness and the net benefit to society of the APTCare intervention. Costs measured included clinical-related costs (such as medication, lab tests, physician salary etc.), APTCare-specific costs (such as staff training, medical supplies, etc.), overhead, and human resource costs. Each participant's quality of care score was also calculated, and patient characteristics were recorded. |
| Bowden, 2004, United States | Uncontrolled before/after | To describe the results of an intervention program to help adults change health risk behaviours and to describe barriers to behaviour change. | Code: Patient Education/Navigation  A counselling intervention was conducted by nurse practitioners (NPs) at a rural clinic in Oregon. The intervention was designed to help individuals change health risk behaviours. Three focus groups were conducted to aid in understanding the barriers to changing health behaviours and to identify resources that rural individuals needed to succeed in behaviour change. | Data was collected using behaviour-specific appraisal forms and written summaries of focus groups and comments from participants. Participants completed at least one behaviour-specific appraisal form at baseline, six months and 12 months. Themes were analysed from focus group data. |
| James, 2002, Australia | Controlled before/after | To evaluate a dissemination and support intervention for unceasing preventive care among rural patients. | Code: Patient Education/Navigation  Researchers designed a nonrandomised trial to evaluate an active dissemination and support intervention. Six rural practices were recruited and paired by practice size and characteristics; three were nonrandomly allocated to ‘active support’ while the others acted as controls. A division prevention facilitator provided active support for six months, working with staff in each practice weekly and visiting at least fortnightly. Support took the form of teaching sessions about health promotion initiatives, assistance with displays, and provision of resources and verbal prompts; but did not include supporting computer-based management practices. Control practices provided data only, which was then fed back to them. | Face-to-face interviews were conducted with consenting staff and GPs from each practice about preventive activities in the practice before and after the intervention. Practice staff completed a Practice Prevention Inventory. Researchers directly observed patient waiting areas and the literature provided there. A patient survey was conducted in each practice of 100 consenting patients randomly selected from patient records. The Patient Practice Prevention Questionnaire was mailed to each patient at the beginning of the study and six months. The questionnaire asked about risk-taking behaviours and current health status. |
| Sexually Transmitted Infection | | | | |
| Ward, 2019, Australia | Cluster RCT | To assess whether continuous improvement strategies related to sexual health could reduce infection rates in Remote Australian Aboriginal communities in Australia. | Code: Audit and Feedback + Financial Incentive  A multifaceted intervention comprising annual assessments of sexual health service delivery, implementation of a sexual health action plan (to improve sexual health service delivery), six-monthly clinical service activity data reports, regular feedback meetings with a regional coordinator, training and financial incentive payments (Services receive payments of $100 (AUD) for each episode of treating patients with STI symptoms at consultation; for treating a person diagnosed with an STI within seven days of the laboratory result being received at the clinic; and for re-testing each individual diagnosed with an STI at three months.). | Primary endpoints were community prevalence and testing coverage in residents aged 16–34 years for Chlamydia trachomatis, Neisseria gonorrhoeae, and Trichomonas vaginalis. |
| Causer, 2018, Australia | RCT | To examine a new diagnostic test for Chlamydia trachomatis (CT) and Neisseria gonorrhoeae (NG) (GeneXpert CT/NG) on its diagnostic performance when used by clinicians in remote community health services in Australia with high prevalences of CT and NG infection. | Code: Implementing a New Service  Primary care services were trained in using the GeneXpert CT/NG for STI testing. Specimens were also sent in parallel laboratory analysis to determine the concordance of results. | The GeneXpert CT/NG test results were compared with corresponding initial laboratory results reported to the health service. The standard method determined positive, negative and overall, per cent concordance separately for CT and NG, along with 95% CI. |
| Hocking, 2018, Australia | Cluster RCT | The Australian Chlamydia Control Effectiveness Pilot (ACCEPt) investigated the effects of opportunistic chlamydia testing in primary care on chlamydia prevalence, pelvic inflammatory disease (PID) and epididymitis in the population. | Code: Healthcare Provider Training + Audit and Feedback  Researchers developed a multifaceted intervention package to encourage staff to offer annual chlamydia testing to all eligible patients based on evidence from systematic reviews and considerations of long-term feasibility in Australian primary care. The intervention package included: an education package for GPs and nurses about strategies for offering testing for chlamydia, management of infection and partner notification; clinical criteria for PID and epididymitis diagnosis; payments for GPs; payments for nurses; quarterly written feedback to GPs on their testing rates discussed in a face-to-face meeting between a research staff member and GPs; a computer alert prompting testing with eligible patients; support to develop a reminder system to recall patients after 12 months if chlamydia negative or after three months if treated for chlamydia; and partner notification information and resources. The intervention included computerised reminders, an education package, payments for chlamydia testing and feedback on testing rates. | The primary outcome was chlamydia prevalence, estimated before randomisation and at the trial end in patients attending clinics. Secondary outcomes included chlamydia testing and the incidence of PID (diagnosed in clinics and hospitals) and epididymitis (in clinics). |
| Skin Infections | | | | |
| Andrews, 2009, Australia | Uncontrolled before/after | To report the results of a community-based collaboration within the East Arnhem Region, which aimed to reduce the prevalence of pyoderma and scabies (skin infections) in Aboriginal children. | Code: Training of Lay Community Members  Involved in conducting an ecological study that included active surveillance for skin infections amongst children in five remote East Arnhem communities over three years. The screening was undertaken by trained local community workers (local Aboriginal people), usually accompanied by another project team member, using a standard data collection form. Skin infections were diagnosed clinically with a pictorial flip chart developed. Topical 5% permethrin was provided for age-eligible children and all household contacts whenever scabies was diagnosed, whilst those with pyoderma were referred to the clinic for treatment following current guidelines. In addition, annual mass scabies treatment (5% permethrin cream) was offered to all community residents following current guidelines but was not directly observed. | Outcomes were recorded over three years, from September 2004–August 2007. Children were screened for skin infections using a standard data collection form. Additional data was collected for a subset of children seen during school screening, which included: the appearance of pyoderma (crusted, purulent or flat/dry), site of pyoderma (upper body or lower body), the number of sores (5, 5–20 or .20) and, for those with scabies, whether or not the infestation had become infected (scabies with a superficial bacterial skin infection = pyoderma). |
| Sleep | | | | |
| McCrae, 2007, United States | RCT | To test the effectiveness of brief behavioural intervention for insomnia in rural elderly. | Code: Healthcare Provider Training + Patient Education/Navigation  Existing care providers (mental health counsellors, a provisionally licensed counsellor, and a social worker) were trained to provide the treatments during a two-day workshop on Multicomponent Behavioral Treatment for insomnia. The training involved teaching different stimulus control recommendations, sleep restriction, and sleep hygiene education. Each patient in the intervention received a workbook containing detailed treatment instructions, sleep diaries, and compliance logs. Treatment was individually administered during two in-person sessions (~50 minutes each) and two telephone follow-ups (~30 minutes each) conducted ~1 week apart. | At the clinical interview, the Geriatric Depression Scale (GDS), Mini-Mental Status Exam (MMSE), Instrumental Activities of Daily Living (IADL), and a two-page demographics/ health questionnaire, which included screening questions for apnea/other sleep disorders, were administered Individuals who were still eligible following the interview completed two weeks of sleep diaries, which had to demonstrate sleep latency or awake time during the night of 31 minutes for six nights Sleep diaries provided the following variables: 1) sleep latency (SL): time from initial lights out until sleep onset; 2) number of awakenings during the night (NWAK); 3) wake after sleep onset (WASO): time awake after initial sleep onset until last awakening; 4) total sleep time (TST): computed by subtracting total wake time from time in bed; and 5) sleep efficiency (SE): ratio of TST to total time in bed. |
| Smoking Cessation | | | | |
| Bottorff, 2016, Canada | Uncontrolled before/after | To examine the impact of a Stop Smoking Before Surgery (SSBS) program in a health authority where health professionals share responsibility for surgical services in regional centres and outlying communities. | Code: Healthcare Provider Training + Patient Education/Navigation  The Stop Smoking Before Surgery (SSBS) program was a collaborative initiative that aimed to (a) increase patient awareness of the benefits of quitting surgery, (b) increase the number of healthcare professionals providing brief interventions to support cessation among surgical patients, and (c) increase the number of patients who quit smoking for surgery. Information and advice regarding stopping smoking for surgery resources were developed and distributed in physician and surgeon offices. All healthcare professionals were encouraged to provide brief interventions following the 5 As (Ask, Advise, Assess, Assist, Arrange), and online training was offered. Stress balls and a discharge brochure to encourage patients to stay smoke-free for recovery were provided for distribution to surgical patients. | Outcomes included assessing whether participants were advised to quit smoking if they quit or reduced smoking before surgery and whether they quit or reduced within a 2-month before surgery. Additionally, they assessed awareness of the risks concerning smoking and surgery and SSBS materials. Demographic questions, type of elective surgery, and questions to describe smoking history (age of initiation, years of smoking, Fagerström Nicotine Dependence Scale) were asked of all participants to describe the sample. |
| Ellerbeck, 2009, United States | RCT | To compare cessation rates among smokers receiving pharmacotherapy alone or combined with either moderate- or high-intensity disease management that includes counselling and provider feedback. | Code: Reorganization of Services  Patients smoking > 10 cigarettes/day were recruited from rural primary care clinics across the state of Kansas and randomly assigned to receive pharmacotherapy management alone, pharmacotherapy management supplemented by 1–2 counselling calls every six months (moderate-intensity disease management), or pharmacotherapy management supplemented by up to 6 counselling calls every six months (high-intensity disease management). For moderate-intensity and high-intensity disease management recipients, periodic progress reports were faxed to the patient’s physician. All participants were offered free pharmacotherapy (bupropion or transdermal nicotine patch) every six months. Participants were enrolled between June 2004 – October 2005 and followed for 24 months, with follow-up completed in December 2007. | The primary outcome measure was self-reported 7-day abstinence at 24 months, defined as not having smoked cigarettes during the prior seven days. Secondary outcomes included self-reported 7-day abstinence at 6, 12, and 18 months and utilization of pharmacotherapy based on whether or not the participant requested bupropion or the transdermal nicotine patch during any 6-month treatment cycle. At baseline, assessed age, gender, education level, and major comorbid conditions. Smoking history included the number of cigarettes smoked per day, previous bupropion use, previous nicotine replacement use, and stage of readiness to stop smoking. Nicotine dependence was assessed using the Fagerström Test for Nicotine Dependence. Importance and confidence in quitting were assessed separately using an 11-point Likert scale ranging from no importance or confidence (0) to extreme importance or confidence (10). |
| Speech Impairments | | | | |
| Kirby, 2018, Australia | Retrospective Cohort | To test the feasibility of providing a nurse-led annual cycle of diabetes care in remote locations and to explore patient-reported factors important in diabetes self-management. | Code: Reorganization of Services  Supervised speech pathology students on rural clinical placement and provided speech, language and communication screening, assessment and therapy to children starting kindergarten in Broken Hill, New South Wales, Australia. The students collected service outcome data for children in the program. | Quarterly clinical outcomes and lifestyle changes were collected from the patient records of all patients involved in the pilot. Categorical variables were created for patient demographics (age, sex, living alone/with others, medications) and outcomes (HbA1C level, glomerular filtration rate, weight) for analysis. Interviews were also conducted; interview questions covered perceptions of diabetes care before and during the nurse-led care and lifestyle adjustments necessitated by diabetes. |
| Stroke Rehabilitation | | | | |
| Sylaja, 2021, India | Cluster RCT | To evaluate whether a Community Health Worker (CHW) based educational intervention will improve risk factor control among stroke survivors and enhance behaviour change communication. | Code: Training of Lay Community Members  A formal training program on symptoms of acute stroke and its management, nursing care of stroke survivors, control of vascular risk factors, caregiver-based rehabilitation, benefits of making regular visits to the home of stroke survivors and education of the patient and the family regarding risk factor control, lifestyle modifications, medication adherence, and blood pressure monitoring was conducted for CHW. | Control of selected risk factors for secondary prevention of stroke (blood pressure, blood sugar levels, cholesterol levels, tobacco and alcohol consumption) among stroke survivors. The quality of behaviour change communication provided by CHWs is assessed through feedback from patients/ caregivers. All the study participants were asked to provide feedback on a structured interview schedule regarding the number of health visits being undertaken by CHWs, and health education provided by CHWs on medication adherence, lifestyle changes, rehabilitation, and dietary modifications. |
| Deng, 2020, China | RCT | To evaluate the effect of an integrated transitional care program on health outcomes in  stroke survivors. | Code: Implementing a New Service  Participants in the intervention received ongoing rehabilitation at home through a multidisciplinary team (specialists & primary care providers). | Primary outcomes were quality of life (short form health survey), functional performance (modified Barthel index), and burden of illness (caregiver strain index). |
| Askim, 2004, Norway | RCT | To evaluate the effect of an extended stroke unit service (extended service), with early supported discharge and coordination of further rehabilitation in co-operation with the primary health care system in three rural municipalities. | Code: Increasing Staff Resources  The intervention involved comparing extended service with ordinary stroke unit service. Extended service involves a stroke unit treatment combined with a home-based programme of follow-up care coordinated by a mobile stroke team that offers early supported discharge and works in close cooperation with the primary health care system during the first four weeks after discharge. | The primary outcome was the proportion of independent patients according to the Modified Rankin Scale 52 weeks after stroke onset. Other outcomes included the Barthel Index to measure disability, Nottingham Health Profile (assesses energy, pain, emotion, sleep, social isolation and physical mobility), Caregiver Strain Index, Mortality and length of stay. |
| Urinary Incontinence | | | | |
| AeyoungSo, 2019, Korea | RCT | To examine the long-term effects of urinary incontinence self-management program for older women on severity, knowledge, and attitudes. | Code: Patient Education/Navigation  The intervention consisted of a 5-sessions urinary incontinence self-management educational program held once weekly. Its contents included a lecture, instructions on pelvic muscle exercises, and assignments related to action plans (exercises and a daily bladder diary). | The primary outcomes were the International Consultation on Incontinence Questionnaire Short Form (ICIQ-SF) score, along with knowledge and attitudes toward UI measured by the UI knowledge scale (UIKS) and UI attitude scale (UIAS). |
| Vaccinations | | | | |
| Chen, 2016, China | Cluster RCT | To assess the effectiveness of an EPI smartphone application (EPI app) in improving vaccination coverage. | Code: Coordination/Referral Pathways  The intervention consisted of giving rural doctors a mobile phone app to manage child vaccination and using text messages to alert caregivers about upcoming vaccinations. The EPI app had four modules with distinct functions: 1) making appointments; 2) recording vaccination status; 3) tracking overdue children; and 4) providing education. | Two cross-sectional household surveys were conducted at baseline (December 2013) and end-line (January 2015) to assess the vaccination coverage of children in rural Sichuan, China. The primary outcome was full vaccination coverage, defined as the percentage of surveyed children aged 12-23 months immunized with three doses of Hepatitis B, one dose of BCG, three doses of OPV, three doses of DPT and one dose of measles vaccine before their first birthday. The secondary outcomes were vaccination coverage indicators of one dose of BCG, three doses of Hepatitis B, three doses of OPV, three doses of DPT, and one dose of MV. Two modules (household information module and child immunization module) were selected from the Maternal, Newborn and Child Health Household Survey (MNCH HHS) Tool. Trained interviewers administered structured questionnaires to collect children's general socioeconomic and vaccination information. |
| Arthur, 2002, United Kingdom | RCT | To compare different approaches in improving influenza vaccination uptake among patients aged 75 years and over in primary care. | Code: Patient Education/Navigation + Increasing Staff Resources  The first approach was a personal letter of invitation to attend any of the influenza vaccination clinics held at the surgery. The letter stressed the importance of preventing influenza and reassured patients that the vaccine was associated with few side effects. The second approach was to combine an over-75 health check carried out by a practice nurse in the patient’s home with an offer of influenza vaccination. Patients were contacted by letter offering a health check that could include an influenza vaccination if the patient wished. | The primary outcome measure was the uptake of influenza vaccination. The primary outcome measure was the proportion of patients receiving the influenza vaccine by 31 December 2000. |
| Kempe, 2001, United States | Cross-sectional | To determine increases in immunization up-to-date (UTD) rates at a rural pediatric practice with the sequential addition of records from other sites (regional registry) | Code: Reorganization of Services  As part of developing a regional registry for the CRISP project, site-speciﬁc computerized immunization databases were established at all major childhood immunization providers in the two target counties in the San Luis Valley. The immunization tracking system consisted of proprietary software utilizing site-speciﬁc Access databases. To populate the tracking system, each private and community health centre site generated an enumeration of all children aged 0– 36 months who had been seen at least once for health maintenance or acute care at that site. Trained CRISP personnel used the enumeration data to directly abstract immunization information from the medical charts into the CRISP tracking system software. For the nursing service sites, data from existing immunization tracking software were migrated into the CRISP tracking system software. A data quality review performed six months after the private and nursing service sites began using the CRISP tracking system compared data in the tracking system to medical record immunization data for children seen after the time the sites began using the tracking system. | The outcomes of interest were the UTD rates at 3, 5, 7, 12, 19, and 24 months of age. The UTD criteria for each age group are summarized in the Table and reﬂect the recommendations of the joint Advisory Committee on Immunization Practices/American Academy of Pediatrics/ American Academy of Family Physicians (ACIP/AAP/ AAFP)–harmonized schedule.19 |
| van Amburgh, 2001, United States | Uncontrolled before/after | To increase the influenza vaccination rate in high-risk patients through a pharmacist-managed immunization campaign. | Code: Patient Education/Navigation  High-risk patients identified by chart review were mailed an education packet on influenza immunization. Vaccinations were given in specially designated clinics and during routine clinic visits. | Follow-up surveys determined campaign success and reasons why patients remained unvaccinated. Outcomes measured included demographics, vaccination rates, and reasons for vaccinations. |
| Vision Impairment | | | | |
| Amritanand, 2018, India | Uncontrolled before/after | To determine the numbers identified with visual problems, establish follow‑up rates, proportions correctly referred by community health workers, and the effect of this intervention on uptake of existing services. | Code: Training of Lay Community Members  Community Health Workers underwent a training module to screen and refer individuals with visual impairments for additional services. | Used surveys to assess perceived visual impairment; any affirmative answers were given referral slips for further evaluation. Proportions of those who followed up after referral and those who were correctly referred were calculated. |
| Weight Management | | | | |
| Befort, 2021, United States | RCT | To compare the Medicare Intensive Behavioral Therapy for Obesity fee-for-service model with two alternatives: in-clinic group visits based on a patient-centred medical home model and telephone-based group visits based on a disease management model. | Code: Patient Education/Navigation  Involved in a lifestyle intervention focused on diet, physical activity, and behaviour change strategies. The fee-for-service intervention involved 15-minute in-clinic individual visits with a physician at a frequency similar to that reimbursed by Medicare (weekly for one month, biweekly for five months, and monthly after that). In the in-clinic group intervention, clinicians delivered group visits that were weekly for three months, biweekly for three months, and monthly after that. In the telephone group intervention, patients received the same intervention as the in-clinic group intervention, but sessions were delivered remotely via conference calls by centralized staff. | The primary outcome was weight change at 24 months. |
| Katzmarzyk, 2021, United States | Cluster RCT | To report the results of five weight-loss interventions in primary care settings in underserved patients and compare the pragmatism level across the interventions using the Pragmatic Explanatory Continuum Indicator Summary (PRECIS-2) tool. | Code: Patient Education/Navigation  The PROPEL-clinic/phone group received counselling sessions for 18 months, starting weekly for six months and then switching to monthly. In-person individual sessions were 30 minutes, in-person group sessions were 1 hour, and phone sessions were 15 to 20 minutes long. All sessions were delivered by study-employed health coaches in the clinics that had received training. Counselling consisted of education on setting goals and developing individualized action plans for diet and physical activity plans. Patients were given an electronic scale (BodyTrace) and told to weigh themselves daily. The primary care physicians in the PROPEL-clinic/phone group had access to an online obesity science education program on obesity management, management of coexisting conditions, minimization of bias and stigma related to obesity, and health literacy principles.  Patients in the REPOWER-clinic-individual group received 15-minute face-to-face individual counselling visits from practice-employed clinicians that occurred weekly for one month, every other week for months 2 to 6, and monthly after that. Counsellors were trained using a 3-hour training session focused on dietary and physical activity recommendations, behavioural strategies, and motivational interviewing.  Patients in the REPOWER-clinic group arm participated in group counselling visits that practice-employed clinicians led. Visits were 60 minutes and occurred weekly for the first three months, every other week for months 4 to 6, and monthly after that. Counsellors in the REPOWER-clinic group arm participated in the same 3-hour training as the REPOWER-clinic-individual arm and received a group treatment manual with accompanying patient manuals, a 1-day  in-person workshop focused on group facilitation, and optional biweekly to monthly telementoring sessions.  Patients in the REPOWERphone-group arm received the same group-based intervention as the REPOWER-clinic group arm. Still, sessions were delivered via telephone conference calls by centralized study staff employed by the research team with graduate degrees in relevant fields (e.g., nutrition, exercise science, psychology). The treatment manual, session frequency, session length, and group size were the same as for the clinic-group arm. Training included shadowing an experienced counsellor, weekly to monthly staff meetings, and fidelity monitoring through review of recorded sessions. | The primary outcome in the PROPEL trial was the weight loss percentage from baseline to 24 months. The primary outcome in the REPOWER trial was absolute weight loss (kilograms) from baseline to 24 months. |
| Brown, 2020, United States | Uncontrolled before/after | To describe and evaluate a telemedicine weight management programme, Wellness Connect, on weight outcomes from seven patient cohorts. | Code: Telehealth or Virtual + Patient Education/Navigation  Eight bi-weekly sessions were provided via telemedicine videoconferencing for groups of patients at these rural primary care clinics led by registered dietitians, exercise physiologists and clinical psychologists. The sessions covered various weight management topics related to the dietary, exercise and behavioural management of obesity. | The primary outcome was a change in weight from baseline to post as measured by Bluetooth-enabled peripherals, which synced data in real-time to a secure iPad app. Participant and provider satisfaction was also examined via a survey. |
| Reed, 2019, United States | RCT | To examine the feasibility of the effectiveness of using the 5A’s model for physical activity counselling on rural adults’ physical activity behaviours. | Code: Implementing a New Service  All subjects wore a Fitbit to track steps and active minutes. The intervention group completed action plans with a nurse practitioner to improve self-regulatory physical activity strategies and received weekly motivational text messages to improve physical activity behaviours. | Physical activity data were collected through Fitbits. Active minutes were summed over 7-day intervals to compute the total active minutes per week for each week of the 12-week study. All other measures were collected at week one and week 12 (completion of the study). The Godin Leisure-Time Exercise Questionnaire (GLTEQ) was used to measure the frequency and duration of physical activity. The theory of planned behaviour (TPB) tool for exercise assessed attitude, subjective norm, perceived behavioural control, and intention toward exercising over the next week. A health beliefs survey for self-regulatory skills assessed the degree to which participants set goals, plan, and track PA. The PROMIS Short Form v1.1—Global Health Scale was used to measure each subject’s perception of their general health status. |
| Barnett, 2017, United States | RCT | To evaluate the results of a quality improvement program to reduce adolescent obesity as measured by BMI 5% over 18 months in a rural health primary care clinic. | Code: Patient Education/Navigation  The intervention provided information on structured physical activity regimens and dietary education classes. | The primary outcomes were BMI and weight. |
| Parra-Medina, 2015, United States | RCT | To pilot a behavioural intervention for overweight or obese Hispanic children in a rural pediatric clinic. | Code: Patient Education/Navigation  Children and their parents were recruited from a rural pediatric clinic and randomized to standard care (SC) or behavioural intervention (INT). In both INT and SC, participants received a Healthy Lifestyle Prescription (HLP) from their clinical provider. The HLP lists 11 healthy lifestyle strategies (i.e., eat breakfast every day, play outside 1 hour a day) for preventing and treating childhood obesity. Parents and children assigned to INT received all elements of SC, plus face-to-face counselling, telephone counselling, and newsletters addressing recommendations for/barriers to physical activity (PA) and dietary practices. | The primary outcomes—weight, waist circumference, and BMI—were measured at baseline, 2, 6, and 18 weeks. |
| Ely, 2008, United States | RCT | To conduct a pilot randomized trial of a chronic care model (CCM) program for obesity care in rural Kansas primary care. | Code: Patient Education/Navigation + Decision Support  The usual care arm received educational weight loss materials and outcome assessments at days 0, 90, and 180. The active arm received the same elements as the usual care arm plus a multicomponent obesity CCM implemented in the following manner: (a) Clinical Information System - an electronic registry of obese patients was created with regular updates to physicians on patient motivation for weight loss, and obesity care recommendations for the subsequent office visits; (b) Decision Support - physicians were provided NIH obesity guidelines and regular updates based on electronic registry information. These updates included guideline-based obesity care recommendations, feedback on patient progress with weight loss behaviour change, and (c) Self-Management Support - patients received regular telephone-based counselling, standardized weight loss materials, pedometers, and diet/physical activity diaries. | The primary outcome was weight change at 90 days. |
| Tyler, 2008, United States | Retrospective Cohort | To conduct a process evaluation of the collaborative negotiation process, an interventional approach was tested at a primary-care school-based clinic to help low-income families improve lifestyle and weight-related health indicators in their overweight children. | Code: Patient Education/Navigation  The intervention consisted of four collaborative negotiation visits with a child, parent and provider during the initial 12 weeks of the program and a booster visit on the 25th week. The intervention was delivered by two advanced practice nurses trained in motivational interviewing and experienced in working with children and families in primary care provided. All participants received information on the Surgeon General’s Call to Action to Prevent and Decrease Overweight and Obesity in Children and Adolescents, materials from the U.S. Department of Agriculture, and a reference page listing six strategies for life-long health based on national recommendations. | The qualitative data collected via structured field notes taken during the parent-child-provider encounters and audio recordings of some visits were analyzed to explore: (a) the participants’ goals and strategies for improving weight-related behaviours; (b) exploring actual and perceived challenges and facilitators to achieving goals; and (c) methods employed for gaining support and overcoming barriers to achieve goals. No quantitative measures were reported. |
