# Supplementary material for: Interventions to improve primary healthcare in rural settings: A scoping review
Source: PLoS One. 2024 Jul 11;19(7):e0305516. doi: 10.1371/journal.pone.0305516 (PMC11239038; doi:10.1371/journal.pone.0305516)
Supplement: S5 Appendix — (DOCX) [file pone.0305516.s006.docx]

**Quality: Patient experience**

| **Author, Year, Country** | **Design** | **Aim** | **Brief Intervention description** | **Outcome measurement** |
| --- | --- | --- | --- | --- |
| Antibiotic Prescribing | | | | |
| Yip, 2014, China | Cluster RCT | To evaluate the effects of capitation with pay-for-performance on primary care providers' antibiotic prescribing practices, health spending, outpatient visit volume, and patient satisfaction. | Code: Reorganization of services  Involved a policy intervention that changed from a fee-for-service to a capitated budget with pay-for-performance to cover outpatient services. | The primary outcome measures were the proportion of patient visits that included one or more prescriptions for antibiotics, total healthcare expenditure per visit, drug expenditure per visit, the number of patient consultations per day in a facility, and patient satisfaction. |
| Burn Rehabilitation | | | | |
| Wiechman, 2014, United States | RCT | To overcome the barriers to effective burn rehabilitation by utilizing an expanded care coordinator (ECC) to supplement the existing outpatient services. | Code: Increasing Staff Resources + Coordination/Referral Pathways  In this between-group, single-blind, randomized, controlled trial, the control group received standard outpatient care, and the experimental group received additional services provided by the ECC, including telephone calls at set intervals (24 hours postdischarge, 2, 4, 8, 12 weeks postdischarge and 5, 7, 9 months postdischarge). The ECC was trained in motivational interviewing, crisis intervention, and solution-focused counselling. They assisted patients before and after each clinic visit, coordinated outpatient services in their geographic area (physical and occupational therapy, counselling, primary care provider referrals, etc.), and helped develop problem-solving approaches to accomplish individualized goals. | Outcome measures included patient-identified goals utilizing the goal attainment scale, the burn-specific health scale-brief, the Short Form 12, a patient satisfaction survey, and a return to work survey. |
| Cancer | | | | |
| Belkora, 2006, United States | Cross-sectional | To determine whether patients receiving consultation planning were satisfied with the service and to explore the variation in patient satisfaction according to co-variates describing the location, provider, and recipient of the CP service. | Code: Increasing Staff Resources  The resource centres sent eight employees and two volunteers for training at UCSF in Consultation Planning (CP). CP is a service to help patients list questions before seeing their doctors. The UCSF CP training sessions consisted of 1-day workshops with two h of intensive instruction on administering the CP prompt sheet and six h of supervised role-playing with feedback. | Measured patient satisfaction of CP service with the Satisfaction with Visit Preparation (SVP) scale. |
| Chronic Disease | | | | |
| Baldwin, 2017, United States | Cross-sectional | To examine the impact of state/territory policy support on the uptake of evidence-based continuous quality improvement activities and quality of care for Indigenous Australians. | Code: Audit and Feedback  The intervention involved implementing the Audit and Best Practice in Chronic Disease (ABCD) programme, which is a continuous quality improvement (CQI) action programme that focuses on enhancing care delivered through Indigenous primary healthcare (PHC) services across Australia. The programme is designed to enable services to assess their practice against evidence-based guidelines, and an audit protocol accompanies each tool in the programme to assess performance. | They used the Quality of Care Index to measure adherence to evidence-based clinical best practice guidelines. |
| Diabetes | | | | |
| McLendon, 2019, United States | Uncontrolled before/after | To evaluate a grant‐funded pilot diabetes care program for rural adults | Code: Patient Education/Navigation + Implementing a New Service  Interventions included nurse care management, telemedicine endocrinology consults, as well as diabetes self‐management education (DSME) to enhance disease management and prevention of complications. | A1C, hospital utilization via hospital claims, and patient and provider satisfaction were measured pre and post-intervention. |
| Pape, 2011, United States | Cluster RCT | To evaluate the impact of remote physician-pharmacist team-based care on cholesterol levels in patients with diabetes mellitus (DM). | Code: Telehealth or Virtual Care  The intervention included remote physician-pharmacist team-based care focused on cholesterol management in DM using a health information technology tool CareManager. It provided automated DM-related point-of-care prompts, a Web-based registry, and performance feedback with benchmarking. According to protocol, the pharmacy practitioner reviewed the patients with elevated LDL-C levels in medical charts. Based on patients' medical conditions and medication history, the pharmacist developed individualized, evidence-based treatment recommendations, including medication therapy and follow-up laboratory monitoring. The proposed treatment plan was electronically sent to the physician for review. The physician had the option to ignore the recommendation, act on the recommendation, or approve the intervention by the pharmacist. | Study outcomes included the difference in low-density lipoprotein cholesterol (LDL-C) goal attainment, mean LDL-C, prescribed lipid-lowering therapy, and patient satisfaction between the intervention and control arms. |
| Elder Care | | | | |
| Tuntland, 2015, Norway | RCT | To investigate the effectiveness of reablement in home-dwelling older adults compared with usual care in relation to daily activities, physical functioning, and health-related quality of life. | Code: Reorganization of Services  Sixty-one home-dwelling older adults with functional decline were randomized to an intervention or control group. The intervention group received ten weeks of multicomponent home-based rehabilitation. | The Canadian Occupational Performance Measure (COPM) was used to measure self-perceived activity performance and satisfaction with performance. In addition, physical capacity and health-related quality of life were measured. The participants were assessed at baseline and 3- and 9-month follow-ups. |
| Family Planning | | | | |
| Tiruneh, 2018, Ethiopia | Uncontrolled before/after | To assess the effectiveness of the BEmONC initiative, this study measures its implementation strength. It examines the effect of its variability across intervention health centres on the rate of facility deliveries and the met need for BEmONC. | Code: Healthcare Provider Training + Increasing Staff Resources  Basic emergency obstetric and newborn care (BEmONC) is a primary health care level initiative promoted in low- and middle-income countries to reduce maternal and newborn mortality. Tailored support, including BEmONC training to providers, mentoring and monitoring through supportive supervision, provision of equipment and supplies, strengthening referral linkages, and improving infection-prevention practice, was provided in a package of interventions to 134 health centres covering 91 rural districts of Ethiopia to ensure timely BEmONC. | Before and after data from 134 intervention health centres were collected in April 2013 and July 2015, a BEmONC implementation strength index was constructed from seven input and five process indicators measured through observation, record review, and provider interview; while facility delivery rate and the met need for expected obstetric complications were measured from service statistics and patient records. |
| Josif, 2014, Australia | Uncontrolled before/after | To describe the experiences of women, midwives, and others during the establishment of a new model of maternity care for remote-dwelling Aboriginal women transferred to a regional centre in northern Australia for maternity care and birth. | Code: Reorganization of Services  The MGP comprises six full-time equivalent (FTE) midwives, two FTE AHWs who are also enrolled as Bachelor of Midwifery students, a Senior Aboriginal woman (SW) from one of the remote communities, and a Coordinator and Administration Ofﬁcer. The MGP team is based in a suburban shopping complex three kilometres from the regional hospital. Care is provided to approximately 190 remote-dwelling Aboriginal women who travel to the regional centre from seven remote 'top-end' communities for maternity care each year. | Data for this paper were collected from semi-structured interviews, ﬁeld notes and observations and analyzed thematically. Data were collected from Aboriginal women using informal conversational interviews and prompts. Semi-structured interviews were conducted with other participants [participants here refer to the Department of Health staff, e.g., the midwives, etc. and stakeholders]. Interview questions with others included the advantages and disadvantages of the MGP and the impact of the MGP on their role or experience. Questions aimed to determine the new model's effectiveness, sustainability and quality. They were subsequently interviewed every three months over one year. |
| Orrantia, 2010, Canada | Cross-sectional | To investigate patient and provider satisfaction with a new model for obstetric care. | Code: Reorganization of Services  The providers created a new model of obstetric care. This involved the local obstetric care providers, each taking one month of the year in rotation and following up with any woman due that month for prenatal and intrapartum services. | Patients were surveyed on patient demographics and satisfaction with their obstetric experience using a Likert scale, yes/no and short-answer questions. Physicians were surveyed on demographics, history of involvement with obstetric service, comparing old and new models concerning patient care and professional and personal issues. |
| Health System Performance | | | | |
| Gur, 2022, Israel | RCT | To assess the impact of the implementation of predefined interventions on the reduction of foci of waste in the clinic workflow. | Code: Well-being  The foci of waste and acceptable interventions were determined and implemented by clinic staff. The interventions focused on reducing wait times (e.g. having patients see the next available physician), clear labelling of exam rooms to avoid confusion, addition of extra work station to increase the efficacy of uploading patient files to EMR, using a word processor to document to prevent loss of data, and moving the printer to increase the efficiency of documentation. | The primary outcome was defined as the average total value stream duration. Secondary outcomes included physician's productivity (measured by the average  number of appointments per working hour) and burnout  (Assessed by the Maslach Burnout Inventory), patient satisfaction (assessed using a scale of 1–6), the proportion of patients returning to the clinic within 30 days, and quality of care (assessed using the Israel Defense Forces Medical Corps "tracers of quality medical care "score). |
| Farmer, 2011, United Kingdom | Cohort | To evaluate the impact and contribution made by PAs to delivering effective health care in National Health Service (NHS) Scotland. | Code: Extending Scope of Practice - Non-FP  The medical home team consisted of a primary care physician and office staff, the child and the family, a nurse practitioner (NP), and a parent consultant (a paid family member of a child with special health care needs). The intervention focused on providing care coordination, information about resources and services, emotional support and encouragement, and empowerment for families to advocate for their children. To accomplish these goals, every participant received from the NP a set of basic services that included a home visit to conduct a comprehensive assessment of medical and nonmedical needs of the child and family members, a personalized letter that described health, educational, and community resources for meeting these needs; an individualized written health plan for the child; assistance in developing short-term family goals; and at least one follow-up to discuss progress toward goals and to problem solve about any barriers to needed care. In addition, the parent consultant assisted with family-to-family support as needed. The intervention lasted six months from the first home visit, with periodic follow-ups in the second 6 months. | Outcomes were assessed at the first visit and after completion of the program. Outcomes included family demographics, child health services (including current health service needs, health service utilization and parental satisfaction with services), family functioning, child functioning, and program acceptability. |
| Anderko, 2000, United States | Uncontrolled before/after | To describe an innovative nurse-managed health centre to improve access to primary health care for residents of a Midwestern three-county rural area. | Code: Extending Scope of Practice - Non-FP  PAs were first piloted in England in 2003 in response to difficulties recruiting medical staff to work as general practitioners (GPs) in disadvantaged areas and inner-city emergency medicine departments. | Patients were satisfied with PAs. The scope of practice did not replicate US work. The inability to prescribe was a hindrance. PAs tended to have longer consultations but provided continuity and an educational resource. They were assessed as mid-level practitioners approximating nurse practitioners or generalist doctors. Valued features were generalism, medical background, confidence differential diagnosis and communication. Interviewees suggested PAs could fulfil medical staff's current roles, potentially saving resources. |
| HIV & Hypertension | | | | |
| Ameh, 2020, South Africa | Cross-sectional | To determine the quality of care provided in the integrated chronic disease model, describe patients' and operational managers' perceptions of the model, and assess the effectiveness of the integrated model in controlling CD4 counts and blood pressure of patients from 2011 to 2013. | Code: Reorganization of Services  The government implemented an integrated chronic disease management (ICDM) model in health facilities as a pilot programme for adult populations. | The primary outcomes included patients' and operational managers' satisfaction using Avedis Donabedian's quality of medical care framework. Additionally, CD4 counts & blood pressure was also measured. |
| Hypertension | | | | |
| Carter, 1997, United States | Controlled before/after | To build on previous research evaluating pharmacy care for hypertension patients, evaluate the program in a rural clinic pharmacy. The impact on blood pressure control, quality of life, patient satisfaction, quality of care, and cost of care was evaluated. | Code: Extending Scope of Practice - Non-FP + Healthcare Provider Training  Researchers designed a program to train community pharmacists to provide hypertension monitoring and direct consultation to physicians and nurses. The pharmacists were provided extensive skill development, including problem-solving workshops and practice experience with the principal investigator in a Veterans Affairs pharmacist-managed hypertension clinic in Chicago. They were given extensive reading materials concerning hypertension, including national guidelines. | Outcome measures include surveys and blood pressure measurements using the American Heart Association Standards and Guidelines. Both study groups completed the Short Form 36 at baseline and six months. Patients also received a questionnaire at the end of the study to assess their overall satisfaction with care delivery and pharmacy services. |
| Integrated Care | | | | |
| Farmer, 2005, United States | Uncontrolled before/after | To evaluate the feasibility and impact of a medical home demonstration project in a rural population. | Code: Coordination/Referral Pathways  The medical home team consisted of a primary care physician and office staff, the child and the family, a nurse practitioner (NP), and a parent consultant (a paid family member of a child with special health care needs). The intervention focused on providing care coordination, information about resources and services, emotional support and encouragement, and empowerment for families to advocate for their children. To accomplish these goals, every participant received from the NP a set of basic services that included a home visit to conduct a comprehensive assessment of medical and nonmedical needs of the child and family members, a personalized letter that described health, educational, and community resources for meeting these needs; an individualized written health plan for the child; assistance in developing short-term family goals; and at least one follow-up to discuss progress toward goals and to problem solve about any barriers to needed care. In addition, the parent consultant assisted with family-to-family support as needed. The intervention lasted six months from the first home visit, with periodic follow-ups in the second 6 months. | Outcomes were assessed at the first visit and after completion of the program. Outcomes included family demographics, child health services (including current health service needs, health service utilization and parental satisfaction with services), family functioning, child functioning, and program acceptability. |
| Medication (prescribing or medication safety) | | | | |
| Vandenberg, 2018, United States | Controlled before/after | To describe the methods used to implement the expanded IMPROVE model and report its effectiveness results, including dissemination feasibility to rural settings. | Code: Healthcare Provider Training + Audit and Feedback  Adapted a successful medication management model, Integrated Management and Polypharmacy Review of Vulnerable Elders (IMPROVE), from an urban geriatric speciality clinic to rural community-based clinics that deliver primary care. The goals were to promote prescribing quality and safety for older adults, including reducing prescribing of potentially inappropriate medications (PIMs). They augmented the original model, which involved a pharmacist-led, one-on-one medication review with high-risk older veterans, to provide rural primary care providers (PCPs) and pharmacists with educational outreach through academic detailing and tools to support safe geriatric prescribing practices, as well as individual audit and feedback on prescribing practice and confidential peer benchmarking. | Analyzed prescribing data in aggregate over three periods: baseline, intervention, and after implementation. The following quality improvement measures were calculated: PIM incidence—number of new PIM prescriptions divided by all encounters (opportunities) that a provider had with veterans aged 65 and older PIM prevalence—number of encounters with veterans currently taking at least 1 PIM divided by all encounters; Multiple PIM prevalence—number of encounters with veterans taking two or more PIMs divided by all encounters They also tracked use of the IMPROVE templated note in the electronic medical record to quantify individual medication management visits with a clinical pharmacist in each CBOC. They collected demographic information on veterans seen and medications stopped or started at the visit with the clinical pharmacist. They contacted each IMPROVE participant (PCP, pharmacist, individuals seen by pharmacist) for a telephone interview to assess their satisfaction with the IMPROVE intervention. |
| Inch, 2017, United Kingdom | Case series | To assess the feasibility and acceptability of delivering five community pharmacy services (CPS) (including advice, sale of six over-the-counter products and dispensing of prescriptions) by tele‐technology (the Telepharmacy 7 Robotic Supply Service (TPRSS)) to a rural population in Scotland. | Code: Decision Support  The TPRSS comprised a medicines supply robot which stocked and supplied over-the-counter medicines and dispensed prescriptions. The robot had a videoconferencing facility with a high‐definition camera, a touch‐screen facility, an integrated telephone, a prescription bar‐code reader, payment capability, and associated hardware and software at the base and remote sites. | Outcomes were assessed using interviews and a follow-up survey. People using the service were invited to be interviewed and offered a follow-up survey. All professionals involved in delivering the TPRSS were invited to a second interview. Interview and focus group schedules were designed to explore stakeholders' perceptions of the need for a TPRSS and to identify their expectations and concerns. The survey Sections included questions on the current use of prescription medicine services; accessing OTC medicines; preferred TPRSS hours of service; expectations of the TPRSS; and demography. |
| Frail, 2016, United States | Retrospective Cohort | To describe an innovative community pharmacy-based pilot program using technology to support care transitions for patients living in rural areas. | Code: Extending Scope of Practice - Non-FP  Community pharmacists worked with patients immediately following discharge to reconcile their medications and make recommendations to optimize therapy. The pharmacy packaged their new medication regimen in precise, individual dose adherence packaging. Medications were delivered by a staff driver to the patient's home within 72 hours of discharge. Patients consulted with the pharmacist by videoconference using a computer tablet device. Patients received telephone follow-ups shortly before their medication supply was to run out and additionally as needed individually. | Self-reported hospital readmissions were collected at 30 and 180 days after enrollment. Patient satisfaction data were also collected at 30 and 180 days using a tool modified from the 5-item Transition Measure (15-item Care Transitions Measure). |
| Mental Health | | | | |
| Rojas, 2018, Chile | Uncontrolled before/after | This study reports the feasibility, acceptability, and effectiveness of a remote collaborative care program for patients with depression living in rural areas of Chile. | Code: Coordination/Referral Pathways  The intervention involves following specific algorithms for treating depression. This involved any primary care clinician referring suspected cases of depression to an on-site physician who can diagnose and initiate treatment. Severe cases were referred to specialized mental health services, and mild to moderate cases may receive a combination of antidepressants, psychosocial interventions, and monitoring visits in primary care, according to severity. | Baseline and follow-up assessments 3 and 6 months after baseline evaluation were carried out via telephone. Treatment adherence to antidepressants during the previous three months was assessed using a questionnaire, and user satisfaction was measured through a depression treatment satisfaction scale. Depressive symptom scores were assessed using the Beck Depression Inventory (BDI-I), and health-related quality of life was recorded by the 36-item Short Form Survey (SF-36). |
| Whealin, 2017, United States | Prospective Cohort | To assess the feasibility of a cultural adaptation of a cognitive-behavioural clinical intervention for use by rural Pacific Island veterans. | Code: Patient Education/Navigation  The " Koa " intervention is a multisession family psychoeducational program that integrates selected Pacific Islander values, beliefs, and healing traditions with an empirically based mainstream US intervention. | Outcomes included relationship quality, relationship satisfaction, caregiver burnout, and patient satisfaction/acceptability. All outcomes were measured via standardized scales or interviews. The fidelity of the intervention's content and process was also monitored to ensure the outcomes could be replicated. |
| Fisher, 2017, United States | Cohort | To assesses whether Project ECHO (Extension for Community Healthcare Outcomes) GEMH (geriatric mental health)—a remote learning and mentoring program—is an effective strategy to address geriatric mental health challenges in rural and underserved communities. | Code: Healthcare Provider Training  They implemented a Project ECHO geriatric mental health (GEMH) hub connecting a team of specialists (geriatric psychiatry and medicine, nursing, social work, psychology, and pharmacy) to spokes of primary care and social service sites. The curriculum consisted of case presentations and didactic lessons that provided participants with information on screening, treatment, and diagnosis of geriatric mental health conditions, focusing on issues faced by older adults related to depression, anxiety, and dementia. Didactic portions of the clinics focused on education and best treatment practices for medication therapies, behavioural interventions, social services, caregiver support, and sleep hygiene. | To understand the program's short-term impact, this study explored changes in participants' geriatric mental health care knowledge, confidence, and treatment practices. It also examined satisfaction with the program and obtained health insurance claims data from a private payer to assess changes in health care utilization and costs before and after the implementation of Project ECHO GEMH. |
| Kozlowski, 2015, United States | Uncontrolled before/after | To assess the feasibility and effects of a brief seven-session cognitive behavioural skills-building intervention, Creating Opportunities for Personal Empowerment (COPE), in children with anxiety. | Code: Patient Education/Navigation  COPE was administered by a trained pediatric nurse practitioner (PNP) using two manuals – a user manual for the PNP and a manual for the child/teen. The individual COPE sessions lasted 30 minutes and followed a specific topic built from session to session. The core concept, a subset of CBT techniques, was presented to the child and then reinforced through games, interactive activities, and real-life application of these concepts. Homework/skills-building activities were assigned to reinforce the topic further. | Postintervention outcomes included assessment of anxiety symptom reduction, cognitive skills knowledge, level of functioning, and post-program satisfaction. Cognitive-behavioural skills learned through the COPE program were assessed through a 15-question content quiz given before and after all seven sessions were complete. Both the children and parents responded to open-ended questions about the helpfulness of the program, specifically what they learned, the length of the program, the therapy location, and if they would recommend this program to another child. |
| Pomerantz, 2008, United States | Uncontrolled before/after | To provide an example of implementing a new program that enhances access to mental health care in primary care. | Code: Coordination/Referral Pathways + Reorganization of Services  Implemented a primary mental health care clinic that provides immediate access to assessment and treatment for all individuals needing mental health services, whether self-referred, identified by their primary care provider (PCP), emergency room staff, or other triage or community referral sources. The clinic relies on self-report psychometrics (patient completes on entry to the clinic) to guide assessment and treatment and to measure outcomes. Clinicians work collaboratively with psychiatrists or psychiatric nurses for diagnostic assessment and treatment plans. | Outcomes included the number of referrals, individuals seen, no-shows/cancellations, wait times, referrals to speciality clinics and speciality no-show rates compared from the 2nd quarter of 2004 to the 4th quarter of 2004. Additionally, they measured patient satisfaction with care via a questionnaire. |
| Cullum, 2007, United Kingdom | RCT | To compare liaison psychiatric nursing with usual medical care in managing older medical inpatients who screened positive for depression. | Code: Reorganization of Services  The intervention was implemented by a liaison psychiatric nurse (LPN) who assessed participants, formulated a care plan for their depression, ensured its implementation through liaison with appropriate agencies, and monitored participants' mood and response to treatment for up to 12 weeks. Participants in the control group received the usual treatment from the hospital and primary care staff. | The primary outcomes at follow-up were the presence of ICD-10-defined depressive disorder and a change in Geriatric Depression Scale-15 score from baseline. Secondary outcomes were differences in quality-adjusted life weeks (QALWs) and patient satisfaction ratings. Outcomes were compared between the intervention group and the control (usual care) group. |
| Minor Illness | | | | |
| Chiu, 2012, Taiwan | Cross-sectional | To evaluate a community-run and GP-supervised self-care for minor illnesses (CGPSC) program in a mountainous area where medical resources for caring for minor illnesses were scarce. | Code: Extending Scope of Practice - Non-FP  The program was implemented through easy-access self-care medical spots (ESCMSs) that were set up in the community with the following services: non-prescription medications for minor illness (MMI) service, materials for caring for minor injuries, a pamphlet on self-care for minor illnesses and minor injuries and medical consultation line. Ten selected residents were trained to run the ESCMSs. The GP supervised all services in charge. The services provided by ESCMS included (i) the MMI service, (ii) supplementary materials (such as bandages, sterilized gauges, etc.) for the ﬁrst-aid kit each household received, (iii) thermometers, (iv) extra copies of the pamphlet on self-care and (v) a 24-hour consultation hotline. All services were free except for the supplementary materials for the ﬁrst-aid kit. Those materials were provided at a low and affordable cost based on the recommendation of the local leaders to prevent abuse of the resource. | A post-intervention questionnaire was conducted to analyze residents' attitudes towards and experience with the intervention and assess the intervention's effect on residents' behaviour on self-care for minor illnesses. |
| Shum, 2000, United Kingdom | RCT | To assess the acceptability and safety of a minor illness service led by practice nurses in general practice. | Code: Extending Scope of Practice - Non-FP  Patients were assigned to treatment by either a specially trained nurse or a general practitioner. Patients seen by a nurse were referred to a general practitioner when appropriate. | The consultation satisfaction questionnaire measured the general satisfaction of the patients. Other outcome measures included the length of the consultation, number of prescriptions written, rates of referral to general practitioners, patient's reported health status, patient's anticipated behaviour in seeking health care in future, and number of patients who returned to the surgery, visits to accident and emergency, and out of hours calls to doctors. |
| Pain Management | | | | |
| Reynolds, 2009, United States | Controlled before/after | To implement and evaluate in a rural setting a predischarge patient education intervention focused on self-pain management of uncomplicated postsurgical patients after being discharged home. | Code: Patient Education/Navigation  The intervention involved patients randomly assigned to the education intervention or control group. The intervention group involved a 10 min session with a research assistant to review a two-page patient education brochure on pain management. | A knowledge and experience questionnaire was completed before and after the study. Additionally, other outcomes included the Brief Pain Inventory, demographics, and a patient pain log used to evaluate the effectiveness of the intervention. |
| Klein, 2017, United States | Cross-sectional | To evaluate a pilot study on improving the care continuity for rural Veterans by health information sharing at a community appointment. | Code: Patient Education/Navigation + Telehealth or Virtual Care  Patients can access their electronic health record data through patient portals to facilitate information exchange. This intervention involved the following: (1) engagement of Veteran's Affairs (VA) facilities and rural community healthcare organizations to develop optimal processes for information exchange to non-VA providers, and (2) Veteran engagement and training in health information sharing using a Continuity of Care Document (CCD). | Patients and non-VA providers completed surveys on their experiences. The questionnaire assessed provider satisfaction with the CCD and whether the provider believed the CCD impacted the care provided. Veterans were also contacted to ask if they discussed their CCD at their appointment, the type of appointment (i.e., primary care or speciality care), and if VA paid for any of the care received. |
| Palliative Care | | | | |
| Bonsignore, 2018, United States | Cohort | To evaluate a telehealth system's feasibility, usability, and acceptability in palliative care. | Code: Telehealth or Virtual Care  Involved using the TapCloud remote patient monitoring application and videoconferencing system. The system allowed patients to access an interdisciplinary team. It addressed the needs of patients with life-limiting illnesses through symptom management, prognostication, psychosocial care, advance care planning (ACP), spiritual care, caregiver support, patient/ family education, and coordination with community-based resources. Physicians and community health workers were trained in using the program to monitor patients' health status and connected with interdisciplinary teams to assist them. | The primary outcomes included patient symptom burden and improvement, hospice transitions, and advanced directives. Qualitative data on satisfaction with the program was collected from a subpopulation of telehealth patients, caregivers, and providers using interviews. The self-efficacy of community healthcare workers and primary care providers was also measured using surveys. All survey measures were reported on a scale of 1 to 7, where "1" indicated "none or no skill" and "7" indicated "expert, teach others." |
| Stroke Rehabilitation | | | | |
| Sylaja, 2021, India | Cluster RCT | To evaluate whether a Community Health Worker (CHW) based educational intervention will improve risk factor control among stroke survivors and enhance behaviour change communication. | Code: Training of Lay Community Members  A formal training program on symptoms of acute stroke and its management, nursing care of stroke survivors, control of vascular risk factors, caregiver-based rehabilitation, benefits of making regular visits to the home of stroke survivors and education of the patient and the family regarding risk factor control, lifestyle modifications, medication adherence, and blood pressure monitoring was conducted for CHW. | Control of selected risk factors for secondary prevention of stroke (blood pressure, blood sugar levels, cholesterol levels, tobacco and alcohol consumption) among stroke survivors. The quality of behaviour change communication provided by CHWs is assessed through feedback from patients/ caregivers. All the study participants were asked to provide feedback on a structured interview schedule regarding the number of health visits being undertaken by CHWs, and health education provided by CHWs on medication adherence, lifestyle changes, rehabilitation, and dietary modifications. |
| Therapeutic Relationship | | | | |
| Naccarella, 2003, Australia | Cross-sectional | The Adolescent Health Project (AHP) was a rural pilot project to strengthen the relationship between general practitioners (GPs) and adolescents within three Divisions of general practice. The evaluation assessed the implementation of the AHP model and strategies and their impact. | Code: Healthcare Provider Training  The intervention included: Health professional education seminar program providing education to GPs, GP clinic staff, school counsellors and other service providers. Along with promoting youth-friendly general practice programs: students visit GP clinics and develop a GP Consultation Protocol and Youth Friendly General Practice Manual. It also included a school program where GPs visited schools and linked GPs and GP clinics with school counsellors. Furthermore, it included a parent education program that conducted a parent education session. The intervention also provided resource development: development of a GP Consultation Protocol, a Youth Health Management Resource/Referral Options, a Youth Friendly General Practice Manual, a Rights and Responsibility Poster, and a Final Project Manual. Finally, it developed new partnerships between Divisions, GPs, schools and the community: facilitating partnerships via structures such as Division-specific Steering Committees. The AHP was centrally managed and supported by an Inter-Divisional Coordinator based in a Division and a GP manager. A Division-specific Project Officer and a local Steering Committee developed and implemented strategies locally. | The evaluation assessed the implementation of the AHP model and strategies and their impacts within the rural general practice setting. The AHP used a qualitative evaluation approach (open-ended surveys, semi-structured interviews and focus group discussions). Feedback forms were developed to assess GPs', school counsellors' and students' beliefs, perceptions, and experiences of the AHP strategies. To assess the implementation of the AHP strategies, two semi-structured interviews were conducted with: the GP Project Manager, Inter-Divisional Coordinator and the three Divisional Project Officers; a sample of six GPs (i.e., six out of 16) and three GP clinic staff (i.e. three out of six) who had participated in the project; and all three School Counsellors who had participated in the project. Questions were posed about involvement in the AHP, perceptions about strategies and the AHP management and support, the transferability of the AHP model, outcomes experienced, future commitments and possible project developments. |
| Veteran's Healthcare | | | | |
| Howe, 2018, United States | Uncontrolled before/after | To strengthen team-based solutions in recognizing issues faced by older veterans. | Code: Healthcare Provider Training  The Rural Interdisciplinary Team Training Program (RITT) is a team-based educational component of the Veterans Health Administration (VHA) Office of Rural Health Geriatric Scholars Program. It is a workforce development program to enhance the geriatrics knowledge and skills of VA primary care clinicians and staff caring for older veterans in rural communities. The RITT workshop, accredited for 6.5 hours, is interactive and multi-modal with didactic mini-lectures, interactive case discussions and role-play demonstrations of assessments. | Evaluated team development with a web-based survey administered before, three, and six months after the workshop. Additionally, evaluated change in geriatric knowledge from immediately before to immediately after the workshop with a geriatrics knowledge test that included multiple choice and true/false questions. Content analysis was used to evaluate the quality of action plans. In addition, participant satisfaction with the program was assessed. |
| Weight Management | | | | |
| Brown, 2020, United States | Uncontrolled before/after | To describe and evaluate a telemedicine weight management programme, Wellness Connect, on weight outcomes from seven patient cohorts. | Code: Telehealth or Virtual Care + Patient Education/Navigation  Eight bi-weekly sessions were provided via telemedicine videoconferencing for groups of patients at these rural primary care clinics led by registered dietitians, exercise physiologists and clinical psychologists. The sessions covered various weight management topics related to the dietary, exercise and behavioural management of obesity. | The primary outcome was a change in weight from baseline to post as measured by Bluetooth-enabled peripherals, which synced data in real-time to a secure iPad app. Participant and provider satisfaction was also examined via a survey. |
