# Supplementary material for: Interventions to improve primary healthcare in rural settings: A scoping review
Source: PLoS One. 2024 Jul 11;19(7):e0305516. doi: 10.1371/journal.pone.0305516 (PMC11239038; doi:10.1371/journal.pone.0305516)
Supplement: S6 Appendix — (DOCX) [file pone.0305516.s007.docx]

**Quality: Patient Equity**

| **Author, Year, Country** | **Design** | **Aim** | **Brief Intervention description** | **Outcome measurement** |
| --- | --- | --- | --- | --- |
| Family Planning | | | | |
| Vellakkal, 2017, India | Controlled before/after | To evaluate the impacts of the National Rural Health Mission (NRHM) on socioeconomic inequities in the uptake of institutional delivery and antenatal care (ANC) across high-focus (deprived) Indian states. | Code: Reorganization of Services  In 2005 India launched the National Rural Health Mission (NRHM) to strengthen the primary healthcare system. NRHM also encourages pregnant women, particularly those of low socioeconomic backgrounds, to use institutional maternal healthcare. The NRHM had a set of core strategies, including increasing public health funding, decentralizing village and district level health planning and management, strengthening the public health service delivery infrastructure, particularly at the village, primary and secondary levels, and promoting the non-profit sectors to increase social participation and community empowerment. | Data from District Level Household and Facility Surveys (DLHS) Rounds 1 (1995–99) and 2 (2000–04) from the pre-NRHM period, and Round 3 (2007–08), Round 4 and Annual Health Survey (2011–12) from the post-NRHM period were used. Wealth-related and education-related relative indexes of inequality, and pre-post difference-in-differences models for wealth and education tertiles, adjusted for maternal age, rural-urban, caste, parity and state-level fixed effects, were estimated. |
| Mental Health | | | | |
| Bowen, 2020, United States | Cohort | To compare the effects of implementing a collaborative care program on depression outcomes in rural Native American and Alaska Native (AI/AN) patients with White patients and patients of other ethnic backgrounds at three clinics. | Code: Reorganization of Services  The intervention was designed to integrate treatment for common mental health disorders (e.g. depression, anxiety) into primary care settings using principles of chronic disease management using an interdisciplinary team (primary care provider, a psychiatric consultant, and a behavioural health care manager). The intervention provided clinic staff with training on universal screening for depression, evidence-based treatment to target, and the use of behavioural health care. | Primary outcomes were depression response (i.e., reduction in symptom severity as measured by PHQ-9) and depression remission (measured by HEDIS) measured over two years. |
| Hanlon, 2019, Ethiopia | Cohort | To evaluate the safety and impact of a district-level plan for task-shared mental health care on six and 12-month clinical and social outcomes of people with severe mental illness in rural southern Ethiopia. | Code: Healthcare Provider Training  An interdisciplinary intervention was implemented at the health system level, primary healthcare facility and community. The intervention focused on training primary healthcare (PHC) workers to assess community referrals, diagnose severe mental illness and initiate treatment with independent research diagnostic assessments by psychiatric nurses. | Primary outcomes included clinical symptom severity (measured by the Brief  Psychiatric Rating Scale), disability (measured by the World Health Organisation  Disability Assessment Schedule), the experience of discrimination (measured using the ‘unfair treatment’ subscale of the discrimination and stigma scale), restraint (measured by self-report of whether the person had been ‘restrained, chained or confined’ in the months preceding), alcohol use disorder (measured by Alcohol Use Disorder Identification Test, depression (measured by Patient Health Questionnaire), suicide attempts in the past three months (measured via Mini International Neuropsychiatric Interview). |
| Mutiso, 2019, Kenya | Uncontrolled before/after | To determine the feasibility of the WHO Mental Health Treatment Gap Interventions Guidelines (mhGAP-IG) to reduce stigma in face-to-face contacts during interventions for specific DSM-IV/ICD 10 diagnoses over six months. | Code: Healthcare Provider Training  Several 1-hour community education sessions were conducted to enhance mental health knowledge and awareness in the catchment communities of the 20 facilities. | They measured stigma using the Discrimination and Stigma Scale at baseline, followed by training the health professionals on intervention using the WHO Mental Health Treatment Gap Interventions Guidelines and then conducted a follow-up on the Discrimination and Stigma Scale assessment after six months. |
