# Supplementary material for: Interventions to improve primary healthcare in rural settings: A scoping review
Source: PLoS One. 2024 Jul 11;19(7):e0305516. doi: 10.1371/journal.pone.0305516 (PMC11239038; doi:10.1371/journal.pone.0305516)
Supplement: S7 Appendix — (DOCX) [file pone.0305516.s008.docx]

**Efficiency: Patient Safety**

| **Author, Year, Country** | **Design** | **Aim** | **Brief Intervention description** | **Outcome measurement** |
| --- | --- | --- | --- | --- |
| Any condition | | | | |
| Jarret, 2019, United States | Uncontrolled before/after | To pilot test a medication reconciliation process called MedManage informed by the Medications at Transitions and Clinical Handoffs (MATCH) toolkit with nursing staff in a rural primary care clinic. | Code: Coordination/Referral Pathways  The intervention consisted of a chart audit tool and a medication reconciliation tool called MedManage. | The primary outcome was medication lists for each patient before and after implementation. |
