# Supplementary material for: Interventions to improve primary healthcare in rural settings: A scoping review
Source: PLoS One. 2024 Jul 11;19(7):e0305516. doi: 10.1371/journal.pone.0305516 (PMC11239038; doi:10.1371/journal.pone.0305516)
Supplement: S8 Appendix — (DOCX) [file pone.0305516.s009.docx]

**Access: Recruitment**

| **Author, Year, Country** | **Design** | **Aim** | **Brief Intervention description** | **Outcome measurement** |
| --- | --- | --- | --- | --- |
| Physician Access | | | | |
| Chevillard, 2021, France | Cohort | To assess the effect of the diffusion of Primary Care Teams (PCTs) on attraction and retention of GPs. | Code: Reorganization of Services + Well-being  A healthcare services delivery reorientation policy was implemented to support the development of primary care teams (PCTs) in France. PCTs are multi-professional group practices with at least two GPs and one paramedic, delivering primary care and services based on cooperation and coordination. It's hypothesized that PCTs will improve healthcare professionals' working conditions, quality, and efficiency gains in delivering care and services (i.e., do more and better with fewer doctors). | The primary outcomes were GP density and PCT locations. A spatial analytical framework was developed to analyze the location and specific impact of PCTs on several types of places. |
| Campbell, 2019, Australia | Cohort | To compare the work locations of graduated medical practitioners who have participated in a new rural placement with those who had not completed any rural medical training and those who had completed rural medical training elsewhere. | Code: Medical Education - Exposure to Rural Practice  The intervention involved the implementation of the East Gippsland rural longitudinal integrated curriculum (EGLIC). The program is classified as a comprehensive LIC because the year four medical course (MBBS) disciplines are integrated, giving rise to year-long continuous professional learning relationships between students, clinicians and the community. | The primary outcome was work location post-training. |
| Chevillard, 2019, France | Cohort | To measure the impact of PCTs settlement on the evolution of GP density in rural areas. | Code: Financial Incentive  France has implemented several policies to provide financial and other incentives to support the development of multi-professional group practices, Primary Care Teams (PCTs) to attract and retain GPs in underserved areas. | The primary outcome was the change in GP density between rural areas with PCTs and similar rural areas without PCTs, before and after the development of PCTs facilities. |
| Flum, 2016, Germany | Cross-sectional | To assess whether implementing a "Rural Day" for GP trainees influences their intention to work in rural practice. | Code: Medical Education- Exposure to Rural Practice  The intervention included a "Rural Day" to ensure rural health exposure for GP trainees. The "Rural Day" consisted of information about the rural region and discussions between GP trainees and political stakeholders. | An internet-based questionnaire was distributed by e-mail to GP trainees who had participated in one of the rural days. The questionnaire contained items related to trainees' socio-demographic characteristics, such as age, sex and origin. Additionally, questions relating to participants' overall and specific attitudes towards rural healthcare, intention to work in rural areas, and the perception of healthcare delivered in rural areas before and after the rural day were included. |
| Taylor, 2016, United States | Retrospective Cohort | To assess the relationship between area health education centre-sponsored family medicine clerkships with a self-reported intent to practice in a rural setting upon graduation. | Code: Medical Education- Exposure to Rural Practice  The intervention allowed 3rd-year medical students to serve vulnerable populations in medically underserved communities in rural settings during their family medicine clerkship in Indiana. | Clerkship data was collected and coded to determine the student's self-reported intent to practice in a rural setting after completing the family medicine clerkship. |
| Wendling, 2016, United States | Retrospective Cohort | To examine the impact of a rural physician program on primary care physician and rural physician recruitment over 30 years. | Code: Medical Education- Selective Recruitment  Michigan State University implemented a rural physician training program to increase interest in rural and primary care practice. | They used data from the American Medical Association Masterfile to determine graduates' specialities and practice locations. |
| Jamieson, 2013, Canada | Cohort | To compare the eventual practice location of family physicians who undertook their postgraduate training through a single university but who were based in either metropolitan or distributed, non-metropolitan communities. Additionally, to identify personal and educational factors that predict future practice location. | Code: Medical Education- Exposure to Rural Practice  They provided a 2-year postgraduate training program for family medicine in two Metro Vancouver (St Paul's and Greater Vancouver) locations, one in three regional settings and a rural program that places residents with preceptors in over 30 small, rural and remote communities. | Residents were surveyed at 2, 5 and 10 years after completion of training. The two-year survey is the most comprehensive, with background information including gender, age at graduation, the status of student loans and prior experience in rural settings such as rural upbringing and rural undergraduate training. All surveys ask about current practice and recent professional activities, reasons for choosing the practice location, and professional and personal satisfaction. Respondents were asked to rate their level of preparedness for practice in several areas on a four-point Likert scale and their overall level of preparedness on a scale of 0–100. |
| MacDowell, 2013, United States | Uncontrolled before/after | To report on the retention and practice outcomes of the University of Illinois College of Medicine at Rockford Rural Medical Education (RMED) Program and to examine distance from influential locations concerning graduates' current practice location. | Code: Medical Education- Selective Recruitment  The RMED program recruits candidates from rural backgrounds, provides a supplemental curriculum addressing rural topics and experiences and tracks speciality and location outcomes for graduates. | Practice location and speciality were compared for 160 RMED graduates and 2,663 non-RMED graduates from 1997 to 2007. |
| Petrany, 2013, United States | Controlled before/after | This study examined the impact of the rural track (RT) on the program's training outcomes and assessed the academic equivalence of the RT and traditional track (TT) curricula. | Code: Medical Education- Exposure to Rural Practice  The Marshall University Family Medicine (MUFMR) implemented its rural residency program to increase the number of its graduates practising in West Virginia. Specifically, each week RT residents provide longitudinal continuity care at the Lincoln Primary Care Centre for one half-day during their first year, three half-days during their second year and 4.5 half-days during their third year. | Practice outcomes (location and type following graduation) were analyzed for program graduates from 1984-2006. Rural-track (RT) and traditional-track (TT) graduates were compared before and after implementing the rural-track program. |
| Pathman, 2012, United States | Uncontrolled before/after | To assess changes to workforce distribution following additional funding for physicians willing to work in underserved areas. | Code: Financial Incentive  The intervention involved loan repayment awards to clinicians who agreed to work in underserved areas. | The primary outcomes were growth and changes in the disciplinary composition of the NHSC's workforce and its rural/urban and state-to-state distribution. |
| Nilsen, 2011, Norway | Controlled before/after | To determine if the programme contributed to reduced vacancies, whether the learning outcome of the off-campus courses was the same as the on-campus programme, and how the education influenced the nurses' professional practice in local health services. | Code: Medical Education- Exposure to Rural Practice  An off-campus bachelor programme (BA) for nurses with flexible learning methods and team-based learning groups in rural contexts was implemented. | Data about course completion, average age, average grades, and retention effect were collected from 3-off campus classes and their contemporary on-campus classes. Additionally, 7 of the off-campus nurses were interviewed. |
| Quinn, 2011, United States | Cohort | To report on the speciality choices and first practice locations of students who participated in the Rural Track Pipeline Program (RTPP). | Code: Medical Education- Selective Recruitment  The RTPP program was created to increase Missouri's supply and retention of rural physicians. The program contains a preadmissions program for rural students (Rural Scholars), a summer community program for second-year students, a six-month rural track clerkship (RTC) program for third-year students, and a rural track elective program for fourth-year students. | They used collected data to compare the speciality choices of Rural Scholars to non-participants and the residency speciality choices of RTC participants and RTC plus participants (students who participated in the RTC program plus an additional RTPP component) to non-participants. The relative likelihood of participants matching into primary care compared to non-participants was calculated, and first practice locations were compared. |
| Rabinowitz, 2011, United States | Retrospective Cohort | To examine the impact of the Physician Shortage Area Program on the proportion of women practising rural family medicine. | Code: Medical Education- Selective Recruitment  The Jefferson Medical College's Physician Shortage Area Program (PSAP) is a special admissions and educational program designed to increase the supply of rural family physicians by recruiting and selectively admitting academically qualified students who grew up or lived in a rural area or small town and who also have a firm commitment to practice the speciality of family medicine. | Data on the sex, speciality, and practice location of all PSAP graduates and their non-PSAP peers from the graduating classes of 1992 to 2002 were used to measure the proportion of women practising rural family medicine. |
| Rabinowitz, 2011, United States | Retrospective Cohort | To compare the geographic location and speciality of Physician Shortage Area Program (PSAP) graduates to non-PSAP graduates and the distribution of PSAP graduates in rural Pennsylvania. | Code: Medical Education- Selective Recruitment  The Jefferson Medical College's Physician Shortage Area Program (PSAP) is a special admissions and educational program designed to increase the supply of rural family physicians by recruiting and selectively admitting academically qualified students who grew up or lived in a rural area or small town and who also have a firm commitment to practice the speciality of family medicine in a similar area. | The data on the 2007 practice location and speciality of PSAP graduates and non-PSAP graduates from 1992-2002 were obtained to determine the likelihood both of PSAP graduates and non-PSAP graduates practising rural family medicine and of all PSAP graduates versus non-PSAP graduates practising in Pennsylvania's rural counties. |
| Straume, 2010, Norway | Retrospective Cohort | To determine the impact of a postgraduate training model for family and public health/community medicine physicians on physician retention in a rural county in Norway. | Code: Medical Education- Exposure to Rural Practice  A postgraduate training model for physicians and public health/community medicine physicians based on tutorial and in-service training in rural areas in Norway. The curriculum is a five-year program of 4 years in family medicine/public health and one year in hospital. | Data was collected over time on postgraduate students and where they are currently practising. The retention rate was defined as 'still working in Finnmark 5 years after completion of the program'. |
| Glasser, 2008, United States | Uncontrolled before/after | To present presents the characteristics and results of the Rural Medical Education (RMED) Program, which addresses medical workforce needs focused on reducing rural health disparities. | Code: Medical Education- Selective Recruitment  The program is comprehensive in implementing a system of recruiting candidates from rural backgrounds, offering a rural-focused curriculum, and instituting evaluative components to track outcomes. Distinctive program features include a Recruitment and Retention Committee of rural community members; special rural-focused topics and events during the first three years of undergraduate medical education; and a required fourth-year, 16-week rural preceptorship through which students work with primary care physicians and conduct community-oriented primary care projects. | Outcomes included recruitment and admissions (to the program), curriculum (self-assessed skills before and after the program), residency and rural practice (program graduates' choice of residency and location of practice), and rural retention (3 or more years). |
| Florence, 2007, United States | Controlled before/after | To compare career choices, attitudes, and practice locations of Community Partnerships Program graduates with traditional graduates. | Code: Medical Education- Selective Recruitment  A health curriculum was developed for nursing, public health and medical students in a rural setting with reinforcement of an emphasis on career choices in medically underserved rural communities. | Used a 21-item questionnaire designed explicitly for the 10-year program assessment to assess career choices, practice locations and attitudes of graduates |
| Dunbabin, 2006, Australia | Cross-sectional | To track the career choice and practice location of medical students entering the Cadetship Program before 1999 and to comment on the program's impact on the rural medical workforce. | Code: Financial Incentive  A Rural Resident Medical Officer Cadetship Program offered bonded scholarships providing residents financial support. In return, cadets are contracted to complete 2 of their three postgraduate years in rural hospitals. | The career choice and practice locations of medical students who received cadetships were tracked. Questionnaires were also used to fill knowledge gaps about where cadets grew up, their vocational training, and where they worked in 2004. |
| Pacheco, 2005, United States | Cross-sectional | To determine the impact of four New Mexico Family Residency Programs on rural New Mexico and what factors may have contributed to its impact. | Code: Medical Education- Exposure to Rural Practice  The University of New Mexico created four strategies to address the health needs of rural New Mexicans. These included: implementing recruiting preferences for rural and ethnic minority applicants, obtaining funding for rural resident positions for one to two months in each of their three years, creating three different rural sites, and creating a state-subsidized locum tenens program staffed primarily by family medicine residents to offer relief to rural practitioners. | They measured several variables correlated with current practice location, including gender, ethnicity, and whether the resident was from the University of New Mexico or another medical school. The program's impact on rural communities was also assessed by examining several factors, such as if the graduates from this program were more likely to remain in New Mexico and practice in rural areas. |
| Jackson, 2003, United States | Controlled before/after | To Assess four service-contingent financial incentive programs for rural physicians | Code: Financial Incentive  Financial incentive programs that offer scholarships and loan repayment to attract students to practice in rural and underserved areas of West Virginia | A 9-page, self-administered questionnaire was mailed to all physician-recipients of 1 or more of the four state financial incentive programs who were currently in their service practice or had completed at least one year of their service since the inception of these programs. Obligated physicians were asked questions about satisfaction with the financial incentive program and factors influencing program commitment. They were compared to a group of rural primary physicians who did not receive financial incentives. |
| Rabinowitz, 2001, United States | Retrospective Cohort | To identify factors independently predictive of rural primary care supply and retention and to determine which components of the PSAP lead to its outcomes. | Code: Medical Education- Selective Recruitment  The Jefferson Medical College's Physician Shortage Area Program (PSAP) is a special admissions and educational program designed to increase the supply of rural family physicians by recruiting and selectively admitting academically qualified students who grew up or lived in a rural area or small town and who also have a firm commitment to practice the speciality of family medicine in a similar area. | Data on physician speciality and 19 predictor variables (demographic variables, premedical background, GPA, admission tests, self-reported career plans, clerkship location, scholarship programs, economic issues) were collected and used to identify factors predictive of rural primary care supply and retention, as well as which components of the PSAP lead the program's success. |
