# Supplementary material for: Interventions to improve primary healthcare in rural settings: A scoping review
Source: PLoS One. 2024 Jul 11;19(7):e0305516. doi: 10.1371/journal.pone.0305516 (PMC11239038; doi:10.1371/journal.pone.0305516)
Supplement: S9 Appendix — (DOCX) [file pone.0305516.s010.docx]

**Access: Retention**

| **Author, Year, Country** | **Design** | **Aim** | **Brief Intervention description** | **Outcome measurement** |
| --- | --- | --- | --- | --- |
| Physician Access | | | | |
| Kitchener, 2020, Australia | Cohort | To evaluate a program for regionalized training for general practice to promote more rural practising physicians. | Code: Medical Education – Exposure to Rural Practice  The Australian General Practice Training (AGPT) program was updated following a review of national training for general practice, intending to produce more rural general practitioners (GPs), addressing the increasing geographical maldistribution of doctors. | The study had two primary outcomes: (1) workforce contribution in the region during training (measured by recording the number of full-time equivalent weeks of training); and (2) retention in the region's rural practices (measured by recording alums' location of practice one year since completion of training). |
| Chevillard, 2019, France | Cohort | To measure the impact of PCTs settlement on the evolution of GP density in rural areas. | Code: Financial Incentive  France has implemented several policies to provide financial and other incentives to support the development of multi-professional group practices, Primary Care Teams (PCTs) to attract and retain GPs in underserved areas. | The primary outcome was the change in GP density between rural areas with PCTs and similar rural areas without PCTs, before and after the development of PCTs facilities. |
| Gardiner, 2013, Australia | Controlled before/after | To determine the relationship between cognitive behavioural coaching, the well-being of rural general practitioners (GPs), their intentions to leave and actual leaving of rural general practice. | Code: Well-Being  A 9-hour cognitive behavioural coaching program known as the Country Practice Retreats. GPs received group and individual coaching, along with 6-weeks of e-mail coaching. | All participants completed a questionnaire before and 3 to 42 months after the program. Rural doctor distress, doctor's intention to leave rural general practice and retention rate was analyzed. |
| Jamieson, 2013, Canada | Cohort | To compare the eventual practice location of family physicians who undertook their postgraduate training through a single university but who were based in either metropolitan or distributed, non-metropolitan communities. Additionally, to identify personal and educational factors that predict future practice location. | Code: Medical Education- Exposure to Rural Practice  It provided a 2-year postgraduate training program for family medicine in two Metro Vancouver (St Paul's and Greater Vancouver) locations, one in three regional settings and a rural program that places residents with preceptors in over 30 small, rural and remote communities. | Residents were surveyed at 2, 5 and 10 years after completion of training. The two-year survey is the most comprehensive, with background information including gender, age at graduation, the status of student loans and prior experience in rural settings such as rural upbringing and rural undergraduate training. All surveys ask about current practice and recent professional activities, reasons for choosing the practice location, and professional and personal satisfaction. Respondents were asked to rate their level of preparedness for practice in several areas on a four-point Likert scale and their overall level of preparedness on a scale of 0–100. |
| MacDowell, 2013, United States | Uncontrolled before/after | To report on the retention and practice outcomes of the University of Illinois College of Medicine at Rockford Rural Medical Education (RMED) Program and to examine distance from influential locations concerning graduates' current practice location. | Code: Medical Education- Selective Recruitment  The RMED program recruits candidates from rural backgrounds, provides a supplemental curriculum addressing rural topics and experiences, and tracks graduates' speciality and location outcomes. | Practice location and speciality were compared for 160 RMED graduates and 2,663 non-RMED graduates from 1997 to 2007. |
| Rabinowitz, 2013, United States | Retrospective Cohort | To evaluate the long-term retention of family physicians from the Physician Shortage Area Program (PSAP) of Jefferson Medical College (JMC) practising in rural areas. | Code: Medical Education- Selective Recruitment  The Jefferson Medical College's Physician Shortage Area Program (PSAP) is a special admissions and educational program designed to increase the supply of rural family physicians by recruiting and selectively admitting academically qualified students who grew up or lived in a rural area or small town and who also have a firm commitment to practice the speciality of family medicine in a similar area. | All JMC graduates were identified from the classes of 1978 to 1986 (including PSAP graduates) who initially practised family medicine in a rural county when first located in practice. The numbers of PSAP and non-PSAP graduates practising family medicine in the same area in 2011 were compared. |
| Nilsen, 2011, Norway | Controlled before/after | To determine if the programme contributed to reduced vacancies, whether the learning outcome of the off-campus courses was the same as the on-campus programme, and how the education influenced the nurses' professional practice in local health services. | Code: Medical Education- Exposure to Rural Practice  An off-campus bachelor programme (BA) for nurses with flexible learning methods and team-based learning groups in rural contexts was implemented. | Data about course completion, average age, average grades, and retention effect were collected from 3-off campus classes and their contemporary on-campus classes. Additionally, 7 of the off-campus nurses were interviewed. |
| Matsumoto, 2010, Japan | Retrospective Cohort | The aim was to determine the impact of a medical education fee waiver program in exchange for 6 to 7 years of rural practice on physicians' practice choices. | Code: Financial Incentive  The Jichi Medical University (JMU) offers a complete medical education scholarship program in exchange for an obligatory 6 to 7-year service in underserved areas. However, whether the experience of contractual rural service affects the physician's choice after the service is completed remains unknown. | JMU graduates under rural service in 2000 and completed service by 2006 were analyzed based on their registered work addresses. Population density quintiles determined the rurality of the communities. |
| Straume, 2010, Norway | Retrospective Cohort | To determine the impact of a postgraduate training model for family and public health/community medicine physicians on physician retention in a rural county in Norway. | Code: Medical Education- Exposure to Rural Practice  A postgraduate training model for physicians and public health/community medicine physicians based on tutorial and in-service training in rural areas in Norway. The curriculum is a five-year program of 4 years in family medicine/public health and one year in hospital. | Data was collected over time on postgraduate students and where they are currently practising. The retention rate was defined as 'still working in Finnmark 5 years after completion of the program'. |
| Matusmoto, 2008, Japan | Retrospective Cohort | To examine the effectiveness of the Jichi Medical University (JMU) 'home prefecture recruiting scheme' and its impact on the retention of students in their home prefecture. | Code: Financial Incentive  The JMU has a contract-based "home prefecture recruiting scheme" in which students recruited from all 'prefectures' are required to work in their home 'prefecture' for nine years after graduation in exchange for having their tuition fees waived. | Baseline data was collected from 1,477 students who graduated from JMU. Follow-up data was collected on whether the graduates still worked in their home prefectures. |
| Gardiner, 2006, Australia | Controlled before/after | This study aimed to evaluate the impact of the Dr DOC program. | Code: Well-Being  The Dr DOC program is a rural workforce support program offering social and emotional support strategies and practice interventions to improve GPs' health and well-being. | GPs completed a questionnaire assessing their levels of support, intention to leave rural practice, use of the Dr DOC program, and psychological health. |
| Pacheco, 2005, United States | Cross-sectional | To determine the impact of four New Mexico Family Residency Programs on rural New Mexico and what factors may have contributed to its impact. | Code: Medical Education- Exposure to Rural Practice  The University of New Mexico created four strategies to address the health needs of rural New Mexicans. These included: implementing recruiting preferences for rural and ethnic minority applicants, obtaining funding for rural resident positions for one to two months in each of their three years, creating three different rural sites, and creating a state-subsidized locum tenens program staffed primarily by family medicine residents to offer relief to rural practitioners. | They measured several variables correlated with current practice location, including gender, ethnicity, and whether the resident was from the University of New Mexico or another medical school. The program's impact on rural communities was also assessed by examining several factors, such as if the graduates from this program were more likely to remain in New Mexico and practice in rural areas. |
| Rabinowitz, 2005, United States | Retrospective Cohort | To determine the long-term retention of rural family physicians graduating from the Physician Shortage Area Program (PSAP) of Jefferson Medical College. | Code: Medical Education- Selective Recruitment  A Physician Shortage Area Program was implemented to increase the retention of rural physicians, consisting of admission, curricular, mentorship, and financial aid components. Only students with firm commitments to practice rural family medicine are admitted, and each student is provided with a faculty advisor, attends regular meetings on family medicine topics and is given a small amount of additional financial aid. | The number of Physician Shortage Area Program graduates and non-PSAP graduates practising family medicine in the same rural area in 2002 were identified compared to the number of graduates practising rural family medicine when they were first located in practice 11-16 years earlier. |
| Jackson, 2003, United States | Controlled before/after | To assess four service-contingent financial incentive programs for rural physicians | Code: Financial Incentive  Financial incentive programs that offer scholarships and loan repayment to attract students to practice in rural and underserved areas of West Virginia | A 9-page, self-administered questionnaire was mailed to all physician-recipients of 1 or more of the four state financial incentive programs who were currently in their service practice or had completed at least one year of their service since the inception of these programs. Obligated physicians were asked about satisfaction with the financial incentive program and factors influencing program commitment. This was compared to a group of rural primary physicians who did not receive financial incentives. |
| Rabinowitz, 2001, United States | Retrospective Cohort | To identify factors independently predictive of rural primary care supply and retention and to determine which components of the PSAP lead to its outcomes. | Code: Medical Education- Selective Recruitment  The Jefferson Medical College's Physician Shortage Area Program (PSAP) is a special admissions and educational program designed to increase the supply of rural family physicians by recruiting and selectively admitting academically qualified students who grew up or lived in a rural area or small town and who also have a firm commitment to practice the speciality of family medicine in a similar area. | Data on physician speciality and 19 predictor variables (demographic variables, premedical background, GPA, admission tests, self-reported career plans, clerkship location, scholarship programs, economic issues) were collected and used to identify factors predictive of rural primary care supply and retention, as well as which components of the PSAP lead the program's success. |
