# Supplementary material for: Interventions to improve primary healthcare in rural settings: A scoping review
Source: PLoS One. 2024 Jul 11;19(7):e0305516. doi: 10.1371/journal.pone.0305516 (PMC11239038; doi:10.1371/journal.pone.0305516)
Supplement: S10 Appendix — (DOCX) [file pone.0305516.s011.docx]

**Access: Access to alternative primary care provider**

| **Author, Year, Country** | **Design** | **Aim** | **Brief Intervention description** | **Outcome measurement** |
| --- | --- | --- | --- | --- |
| Diabetes | | | | |
| Bray, 2005, United States | Controlled before/after | To explore the efficacy of combining care management and interdisciplinary group visits for rural African American patients with diabetes mellitus. | Code: Extending Scope of Practice - Non-FP  In the intervention practice, an advanced practice nurse visited the practice weekly for 12 months and facilitated diabetes education, patient flow and management. Patients participated in a 4-session group visit education/support program led by a nurse, a physician, a pharmacist, and a nutritionist. The control practice patients in a separate practice received the usual care. | Outcomes included weight, blood pressure, and HbA1c values from before enrollment in the system redesigns and from approximately 12 months after enrollment were abstracted from patient records in the CVDEMS system at the intervention practice. Data on demographics and glycemic control only were collected in the control practice. |
| Family Planning | | | | |
| Bonnell, 2018, Dominican Republic | Uncontrolled before/after | To evaluate the feasibility and acceptability of using mobile health technology by community health workers (CHWs) to improve the identification of pregnancy complications and access to care for pregnant women. | Code: Training of Lay Community Members  CHWs in three communities were taught to provide third-trimester antenatal assessment, upload the data on a mobile phone application, send the data to the local physician who monitored data for "red flags," and call directly if a mother had an urgent problem. | Data were measured at baseline and after baby delivery (or follow-up visit). Variables measured included gravida; parity; abortions (loss of pregnancy before 20 weeks gestation); natimuertos (loss of pregnancy after 20 weeks gestation); the number of cesarean deliveries; birth date of most recent child; method of delivery; child's general health at birth; ages of children; last menstrual cycle; due date; the number of prenatal visits with current pregnancy; a medical history of thalassemia; whether mother has received tetanus vaccination, and if so how many; medications being taken, including folic acid, iron, and calcium; maternal height and weight (body mass index); and hemoglobin. Women were also assessed for alcohol, tobacco, substance use, and safety in the home. A senior supervising CHW and/or the physician obtained a haemoglobin level. Additionally, the number of participants lost to follow-up was also measured. |
| Health System Performance | | | | |
| Farmer, 2011, United Kingdom | Cohort | To evaluate the impact and contribution made by PAs to delivering effective health care in National Health Service (NHS) Scotland. | Code: Extending Scope of Practice - Non-FP  The medical home team consisted of a primary care physician and office staff, the child and the family, a nurse practitioner (NP), and a parent consultant (a paid family member of a child with special health care needs). The intervention focused on providing care coordination, information about resources and services, emotional support and encouragement, and empowerment for families to advocate for their children. To accomplish these goals, every participant received from the NP a set of essential services that included a home visit to conduct a comprehensive assessment of medical and nonmedical needs of the child and family members, a personalized letter that described health, educational, and community resources for meeting these needs; an individualized written health plan for the child; assistance in developing short-term family goals; and at least one follow-up to discuss progress toward goals and to problem solve about any barriers to needed care. In addition, the parent consultant assisted with family-to-family support as needed. The intervention lasted six months from the first home visit, with periodic follow-ups in the second 6 months. | Outcomes were assessed at the first visit and after completion of the program. Outcomes included family demographics, child health services (including current health service needs, health service utilization and parental satisfaction with services), family functioning, child functioning, and program acceptability. |
| Probst, 2009, United States | Cross-sectional | To clarify the contribution that community health centres (CHCs) and rural health clinics (RHCs) may make to the accessibility of primary health care. | Code: Financial Incentive  Conducted an ecologic analysis of the relationship between facility presence and county-level hospitalization rates, using 2002 discharge data from eight states within the US (579 counties). | They measured county-level hospitalization rates for ambulatory care-sensitive (ACS) conditions. Discharge rates were based on the individual's county of residence and were obtained by dividing ACS hospitalizations by the relevant county population. |
| Sears, 2008, United States | Uncontrolled before/after | To (1) describe the contribution of nurse practitioners to Washington's workers' compensation provider workforce, (2) evaluate change in provider availability attributable to new policy changes. | Code: Extending Scope of Practice - Non-FP  A 3-year pilot program to expand the role of nurse practitioners (NPs) in the Washington State workers' compensation system was implemented in 2004. NPs were allowed to treat injured workers without needing a physician to sign off on key workers' compensation forms. | Data was collected one year before the policy and one-year post changes. Data measured included NP contribution to the workforce (via computation of provider-to-population ratios, medical morbidity, and utilization), the incidence of provider applications, and change in timely accident report filing for NPs. |
| Anderko, 2000, United States | Uncontrolled before/after | To describe an innovative nurse-managed health centre to improve access to primary health care for residents of a Midwestern three-county rural area. | Code: Extending Scope of Practice - Non-FP  PAs were first piloted in England in 2003 in response to difficulties recruiting medical staff to work as general practitioners (GPs) in disadvantaged areas and inner-city emergency medicine departments. | Surveys were used to measure client satisfaction and registration and billing information was used to measure healthcare utilization. |
| Shum, 2000, United Kingdom | RCT | To assess the acceptability and safety of a minor illness service led by practice nurses in general practice. | Code: Extending Scope of Practice - Non FP  Patients were assigned to treatment by either a specially trained nurse or a general practitioner. Patients seen by a nurse were referred to a general practitioner when appropriate. | The general satisfaction of the patients as measured by the consultation satisfaction questionnaire. Other outcome measures included the length of the consultation, number of prescriptions written, rates of referral to general practitioners, patient's reported health status, patient's anticipated behaviour in seeking health care in future, and number of patients who returned to the surgery, visits to accident and emergency, and out of hours calls to doctors. |
| Medication (prescribing or medication safety) | | | | |
| Falamic, 2018, Croatia | RCT | To determine the effect of a pharmacist-delivered education and medication review on time in therapeutic range (TTR) in elderly patients on warfarin. | Code: Extending Scope of Practice - Non-FP  45-minute education session on warfarin treatment. Participants were then given a dosing scheme for their warfarin therapy and followed-up monthly for six months. | TTR was measured using the Rosendaal method. |
| Frail, 2016, United States | Retrospective Cohort | To describe an innovative community pharmacy-based pilot program using technology to support transitions of care for patients living in rural areas. | Code: Extending Scope of Practice - Non-FP  Community pharmacists worked with patients immediately following discharge to reconcile their medications and make recommendations to optimize therapy. The pharmacy packaged their new medication regimen in clear, individual dose adherence packaging. Medications were delivered by a staff driver to the patient's home within 72 hours of discharge. Patients consulted with the pharmacist by videoconference using a computer tablet device. Patients received telephone follow-up shortly before their medication supply was to run out, and additionally as needed on an individual basis. | Self-reported hospital readmissions were collected at 30 and 180 days after enrollment. Patient satisfaction data were also collected at 30 and 180 days using a tool modified from the 5-item Transition Measure (15-item Care Transitions Measure). |
| Mental Health | | | | |
| Buist, 2019, Scotland | Uncontrolled before/after | To evaluate a pilot mental health intervention using pharmacists to provide psychopharmacological care for depression and anxiety from the patient and provider perspective. | Code: Extended Scope of Practice – Non -FP  A 12-month pilot was implemented in two general practices in remote and rural Scotland. General practitioners referred patients to specialist mental health pharmacists as independent prescribers for evidence-based psychopharmacological interventions. As part of the service, all patients completed Patient Health Questionnaire (PHQ-9) and/or Generalized Anxiety Disorder (GAD-7) rating scales at their frst and last appointments. | The primary outcome was reduction in anxiety and/or depression symptoms as measured by Patient Health Questionnaire (PHQ-9) and/or Generalized Anxiety Disorder (GAD-7) rating scales. |
| Maconick, 2018, South Africa | Prospective Cohort | To develop and evaluate a locally delivered, long-term, in-service training programme to facilitate mental health care in primary care. | Code: Extending Scope of Practice - Non-FP  The in-service training programme was delivered weekly 1-h sessions by local psychiatry staff to 20 primary care nurses at the clinic over five months. The training was based on the "Practical Approach to Care Kit" guidelines that teach primary care workers first-line treatments for depression, substance misuse, psychosis and dementia. | Data were collected before the training began and again at four months. A questionnaire was administered to all participants examining competence at diagnosing and treating common mental disorders and some core skills for mental health, such as mental state examination. Additionally, the number of referrals was collected for one month before training and at the 4-month evaluation mark after training was complete. Interviews were conducted to assess potential barriers to using the training at work every day. |
| Maulik, 2017, India | Uncontrolled before/after | To evaluate the feasibility and acceptability of an intervention for identifying and treating common mental health disorders. | Code: Extending Scope of Practice - Non-FP  A mental health services delivery model that leveraged technology and task sharing to facilitate identification and treatment (including following treatment guidelines) of common mental disorders (CMDs) such as stress, depression, anxiety and suicide risk in rural areas of Andhra Pradesh, India. The intervention was delivered by lay village health workers (Accredited Social Health Activists – ASHAs) and primary care doctors. An anti-stigma campaign using multi-media approaches was conducted across the villages at the project's outset. | The primary outcome was an evaluation of pre- and post-intervention mental health service utilization. Additionally, depression and anxiety scores of those who tested positive for a CMD at baseline were measured and compared post-intervention. Trained interviewers conducted a baseline survey. The survey enquired about sociodemographic details, stressors, social networks, CMD, history of mental disorders and their treatment, family history of mental disorders, and perceptions about stigma related to mental health. Process evaluation of the project was done using focus group discussions and in-depth interviews with key stakeholders. |
| Malcolm, 2002, Australia | Uncontrolled before/after | To evaluate the effects of employing a mental health worker to provide additional mental health services on access to treatment and mental health outcomes (i.e., symptoms and functioning). | Code: Extending Scope of Practice - Non-FP  Involved employing a mental health worker to provide counselling, educate patients and the public about mental illness, improve the skills of local health workers, liaise with other counselling agencies, and undertake research into mental health in the area. | Questionnaires were given to patients seen and returned anonymously. Questions covered demographics, referral source, reason for presentation, the effect of stigma on help-seeking behaviour and changes in symptoms. Other questionnaires were used to help assess the project's impact on patients presenting with non-mental health problems, relatives of those with mental illness, GPs and the local community. Figures were obtained from the State Coroner's Office for suicides in the project area. Number of known suicides in the area in the three years before the commencement of the project and the three years during which the project was noted. Additionally, numbers of new patients, age-range and geographical distribution, and diagnoses made by the mental health worker were recorded. |
| Minor Illness | | | | |
| Chiu, 2012, Taiwan | Cross-sectional | To evaluate a community-run and GP-supervised self-care for minor illnesses (CGPSC) program in a mountainous area where medical resources for caring for minor illnesses were scarce. | Code: Extending Scope of Practice - Non-FP  The program was implemented through easy-access self-care medical spots (ESCMSs) that were set up in the community with the following services: non-prescription medications for minor illness (MMI) service, materials for caring for minor injuries, a pamphlet on self-care for minor illnesses and minor injuries and medical consultation line. Ten selected residents were trained to run the ESCMSs. The GP supervised all services in charge. The services provided by ESCMS included (i) the MMI service, (ii) supplementary materials (such as bandages, sterilized gauges, etc.) for the ﬁrst-aid kit each household received, (iii) thermometers, (iv) extra copies of the pamphlet on self-care and (v) a 24-hour consultation hotline. All services were provided free of charge except for the supplementary materials for the ﬁrst-aid kit. Those materials were provided at a low and affordable cost based on the recommendation of the local leaders to prevent abuse of the resource. | A post-intervention questionnaire waws conducted to analyze residents' attitude towards and experience with the intervention and assessing the effect the intervention had on residents' behaviour on self-care for minor illnesses. |
| Palliative Care | | | | |
| Mitchell, 2016, Australia | Uncontrolled before/after | To evaluate a pilot on whether nurse practitioner (NP)-led care, including clinical care plans negotiated with involved health professionals, including the general practitioner (GP), ± patient and carer, through a single multidisciplinary case conference (SMCC), could influence patient and health system outcomes. | Code: Extending Scope of Practice - Non-FP  The intervention was NP-led and involved the NP preforming an assessment followed by an SMCC as soon as possible after referral. Then a clinical care plan was developed to create management plans for current and anticipated problems and to dictate who was responsible for each action. | Eligible patients had baseline, 1 and 3-month patient-reported assessment of function, quality of life, depression and carer stress, and a clinical record audit. Interviews with key service providers assessed the utility and feasibility of the service. |
