# Supplementary material for: Interventions to improve primary healthcare in rural settings: A scoping review
Source: PLoS One. 2024 Jul 11;19(7):e0305516. doi: 10.1371/journal.pone.0305516 (PMC11239038; doi:10.1371/journal.pone.0305516)
Supplement: S11 Appendix — (DOCX) [file pone.0305516.s012.docx]

**Access: Services not normally delivered by family physicians**

| **Author, Year, Country** | **Design** | **Aim** | **Brief Intervention description** | **Outcome measurement** |
| --- | --- | --- | --- | --- |
| Cancer | | | | |
| Johansson, 1999, Sweden | RCT | To evaluate the effects of intensified primary care on cancer patients' home care nurse contacts and to study if patients' use of home care services six months after diagnosis can be predicted. | Code: Extending Scope of Practice - Non-FP  Intensified primary care involves routines to improve general practitioners' and home care nurses' possibilities to support and monitor patients, i.e., increased information from specialist care, education and supervision in cancer care. | Demographic data and data on treatments were collected during the first six months after diagnosis. Anxiety and depression were also assessed using a standardized measure. An 18-item questionnaire was developed to assess patients' contacts with the home care nurses and the perceived benefits of these contacts six months after diagnosis. |
| Cardiovascular Disease | | | | |
| Shetty, 2017, India | Prospective Cohort | To demonstrate the feasibility of a telecardiology system to link rural clinics to a teaching hospital. | Code: Extending Scope of Practice - Non-FP  Five rural clinics were linked to a teaching hospital, using an inexpensive system of cardiographs and tablet PCs to transmit ECGs to the hospital and have them interpreted by cardiologists. Training was provided to clinical staff. | Outcomes included metrics of data quality, system performance, and operational variables related to the recording of quality, noise-free ECGs, the transmission of ECGs from the clinics to the hospital, ECG interpretation, and response to PCP. In addition, data on the prevalence of ECG abnormalities in enrolled patients and cardiologists' advice to patients on further follow‑up were also analyzed. |
| Care Seeking | | | | |
| Jordans, 2017, Nepal | Prospective Cohort | To determine whether the application of the tool increases help-seeking behaviour among people who would otherwise be unlikely to seek care and to assess how many of the referred people pursued primary healthcare services and started on treatment. | Code: Coordination/Referral Pathways  The tool facilitates the detection of people with depression, alcohol-use disorder, epilepsy and psychosis and helps identified people seek care. The tool is developed on the premise that people intimately connected within the community, such as community health workers (CHWs), can identify those needing care if provided with a tool for identification. The structured tool contains vignettes, which are sensitive to the context, rather than symptom checklists and uses pictures that are easy to understand for low literacy populations. Trained lay community informants (e.g., CHWs or civil society women's groups) use the tool during daily routine activities, where they check the extent to which people match paragraph-long vignettes using a four-point scale. The pictorial vignettes are designed to initiate help-seeking for mental health treatment in primary care settings. The community informants do the vignette matching based on their observation of people as part of their interactions during their regular responsibilities. If the person fits well with the description, they will ask additional questions on the need for support or impairment in functioning. | The people who the informants were able to reach after three weeks and who provided consent to participate in the study were asked whether they had visited a healthcare facility in the past three weeks. The participants who answered "yes" were also asked who or what determined whether they sought help (including referral through the tool as one of the options), what problem they sought help for, whether treatment was initiated, and if so, what treatment. Additionally, we asked participants for sociodemographic characteristics. For the participants that accessed health care, we cross-checked their answers with their clinical diagnosis and treatment records and checked the clinical records for completeness. |
| Chronic Disease | | | | |
| Wu, 2016, China | Controlled before/after | To evaluate the effectiveness of an intervention to increase access to Basic Public health Services (BPHS) in the Henan province of China. | Code: Patient Education/Navigation + Increasing Staff Resources  The Basic Public Health Services (BPHS) intervention included: the creation of health records for rural families, health education, and health care for children under three years old, maternal health care, health care for older persons, immunization, reporting of infectious disease, management of hypertension and diabetes and treatment of severe mental illness. The county developed detailed management guidelines for each item in the package according to the National BPHS Guideline Book. It established a team to monitor each township quarterly to assess their adherence to the management guidelines. Monitoring reports captured the performance at township and village levels and determined the performance-related element of their pay. | They evaluated the intervention from five aspects, i) distribution of funds; ii) workload of health services providers; iii) payment to health workers; iv) number of patient referrals between different levels of health institutes) outputs of chronic disease management (hypertension and diabetes). |
| Diabetes | | | | |
| Peckens, 2020, United States | Cohort | To describe the strategies a family medicine clinic in Appalachia utilized to increase nephropathy screening rates and explore the factors predictive of nephropathy screening in patients with diabetes. | Code: Screening + Patient Education/Navigation + Healthcare Provider Training  Involved bulk orders for albumin-to-creatinine (ACR) testing and urine collection during clinic visits, enhanced patient communication through bulk communication reminders and individual patient outreach, and education of clinic providers. | To compare patients screened within the previous 12 months to those overdue for screening, 2-sample t-tests were used to examine differences in patients' age, HbA1c, ACR, creatinine level and the distance (in miles) between the patient's home and the clinic. |
| Kirby, 2018, Australia | Retrospective Cohort | To test the feasibility of providing a nurse-led annual cycle of diabetes care in remote locations and to explore patient-reported factors important in diabetes self-management. | Code: Reorganization of Services  Supervised speech pathology students on rural clinical placement and provided speech, language and communication screening, assessment and therapy to children starting kindergarten in Broken Hill, New South Wales, Australia. The students collected service outcome data for children in the program. | Quarterly clinical outcomes and lifestyle changes were collected from the patient records of all patients involved in the pilot. Categorical variables were created for patient demographics (age, sex, living alone/with others, medications) and outcomes (HbA1C level, glomerular filtration rate, weight) for analysis. Interviews were also conducted. Interview questions covered perceptions of diabetes care before and during the nurse-led care and lifestyle adjustments necessitated by diabetes. |
| Feltner, 2017, United States | Controlled before/after | To evaluate the effectiveness of community health workers' (CHWs) health coaching and support in improving diabetes health outcomes. | Code: Extending Scope of Practice - Non-FP  Seventeen CHWs were trained to administer the study measures, provide coordination for the nurse educator, answer questions and provide supporting educational materials after clients received the intervention. All participants completed each nurse education screening visit. They all received the CHW health coaching home visits to repeat the nurse education materials and to preview the following nurse education materials on diabetes self-management education. | All participants completed all pre- and post-test surveys and clinical outcome measures (height, weight, to calculate their BMI, blood pressure, random glucose level, foot check, medication review, and HbA1c). The CHWs collected demographic and background data, including age, gender, marital status, level of education, income, federal poverty level, health insurance status, visits to diabetes educators, and New Vital Sign (NVS) test of health literacy levels. The CHWs also administered the Diabetes Knowledge Test (DKT), Diabetes Empowerment Scale-Short Form (DES-SF), and the Summary of Diabetes Self-Care Activities (SDSCA) measures pre- and post-Diabetes Self-Management Education (DSME) intervention. |
| RodriguezVilla, 2016, Spain | Retrospective Cohort | The objective of this study is to analyze the results of said teleophthalmology program, assess the epidemiological characteristics of the included population, the diagnostic capability of primary care physicians to identify diabetic retinopathy (DR) and adequate coordination with ophthalmologists, as well as assessing the savings said program produces. | Code: Extending Scope of Practice - Non-FP  Primary care physicians were trained to interpret retinographs (DR classification as per the international DR severity scale) with four-hour training workshops. In addition, two nurses were trained to carry out non-mydriatic retinographs (NMR), one posterior pole and four peripheral, and assess intraocular pressure (IOP) with applanation tonometry and detect alterations in the Amsler grid. | Outcomes included clinical measures of age, type of DM, years of evolution, treatment received (only diet, oral antidiabetics [OAD], insulin or combination of both), glycosylated hemoglobin percentage (HbA1c) taken three months before or after the retinograph date, presence of other cardiovascular risk factors (arterial hypertension, dyslipidemia, diabetic nephropathy defined as microalbuminuria >30mg/ml/24h or microalbumin/creatinine ratio of >20mg/g), cardiovascular pathologies of the ischemic cardiopathy type, ischemic encephalopathy and peripheral ischemia such as diabetic foot or amputations due to distal necrosis. Additionally, all retinographs of the sample were assessed by two ophthalmologists for accuracy. |
| Pastel, 2009, United States | Interrupted Time Series | To evaluate the change caused by process redesign to identify all patients with diabetes every time they visit the clinic and improve performance and documentation of recommended process measures. | Code: Extending Scope of Practice - Non-FP  The team of providers and licensed nursing assistants (LNAs) assessed the gaps in care by walking through the process of an office visit. Key leverage points were identified, such as notifying providers of care needed that day, engaging frontline workers in completing the care, and creating a system to ensure documentation. The LNAs generated a list of patients with diabetes who had scheduled appointments every clinic day using the hospital database. A flow sheet was designed that listed the last dates of all required routine care for a patient with diabetes (I.e., the last date of A1C testing and the last date of foot exam). Flow sheets were completed by an LNA each day for scheduled patients with diabetes. The flow sheets prompted LNAs and providers to address needed care. Documentation of the day's care was then entered into the electronic medical record by an LNA. Results and ongoing plans were displayed on a dashboard in the staff lounge and discussed during staff meetings. | To track improvement, the clinic focused on both process and outcome measures. Data were obtained monthly from the Hitchcock Data Reporting System (HDRS), a database of patient measures based on coding and documentation in the computerized information system flow sheets. The team followed rates of pneumococcal and influenza vaccinations, foot exams, eye exams, A1C testing, and LDL and blood pressure control. Data were obtained monthly from HDRS reports from February 2007 until April 2008. |
| Elder Care | | | | |
| Prasad, 2014, Canada | Uncontrolled before/after | To describe the implementation of the Care for Seniors model of care and to provide preliminary evidence of the effective use of specialist resources and acute care services. | Code: Coordination/Referral Pathways  The Care for Seniors model is innovative in improving care coordination and integration. This program aims to enable older adults with complex medical conditions to live to their fullest quality of life in a safe and acceptable environment. In this model, the NP-Geri is pivotal as she collaborates with PCPs and a geriatrician to provide coordinated, comprehensive care. | Number of new geriatrician referrals and follow-up visits before and after the launch of the Care for Seniors program, number of Nurse Practitioner visits in a primary care setting, in-home, retirement home and hospital, number of discharges from hospital and length of hospital stay. |
| Family Planning | | | | |
| Bonnell, 2018, Dominican Republic | Uncontrolled before/after | To evaluate the feasibility and acceptability of using mobile health technology by community health workers (CHWs) to improve the identification of pregnancy complications and access to care for pregnant women. | Code: Training of Lay Community Members  CHWs in three communities were taught to provide third-trimester antenatal assessment, upload the data on a mobile phone application, send the data to the local physician who monitored data for "red flags," and call directly if a mother had an urgent problem. | Data were measured at baseline and after baby delivery (or follow-up visit). Variables measured included gravida; parity; abortions (loss of pregnancy before 20 weeks gestation); natimuertos (loss of pregnancy after 20 weeks gestation); the number of cesarean deliveries; birth date of most recent child; method of delivery; child's general health at birth; ages of children; last menstrual cycle; due date; the number of prenatal visits with current pregnancy; a medical history of thalassemia; whether mother has received tetanus vaccination, and if so how many; medications being taken, including folic acid, iron, and calcium; maternal height and weight (body mass index); and hemoglobin. Women were also assessed for alcohol, tobacco, substance use, and safety in the home. A senior supervising CHW and the physician obtained a hemoglobin level. Additionally, the number of participants lost to follow-up was also measured. |
| Yugbaré Belemsaga, 2018, Burkina Faso | Uncontrolled before/after | To reduce maternal and newborn mortality and morbidity within the year after childbirth in four sub-Saharan African countries. | Code: Extending Scope of Practice - Non-FP  MOMI interventions, including integrating maternal and infant services in the postpartum (PP) period at day 6–10, weeks 6–8, and month nine, were implemented from September 2013 to December 2015. | Outcome indicators were: attendance of PPC on days 6–10 and weeks 6–8, provision of PP family planning counselling, and management of PP morbidity in mother and infant. |
| Iyer, 2017, Rwanda | Interrupted Time Series | To evaluate the impact of an HSS (health system strengthening) intervention to improve primary health care facility readiness on health service utilization for pregnancy-related health care in two rural districts of Rwanda. | Code: Extending Scope of Practice - Non-FP  Used controlled ITS analysis to compare changes in healthcare utilization at health centres (HC) that received the intervention to propensity score matched non-intervention health centres. HC support included infrastructure renovation, salary support, medical equipment, referral network strengthening, and clinical training. | Baseline quarterly mean outpatient visit rates and population density were used to model propensity scores. Used monthly healthcare utilization data from the national Rwandan HMIS to study changes in the number of facility deliveries per 10,000 women, the number of referrals for high-risk pregnancy per 100,000 women, and the number of outpatient visits performed per 1,000 catchment population. |
| Patel, 2016, Ghana | Uncontrolled before/after | To increase the capacity in the community to recognize signs and symptoms of emergencies, encourage prompt decision-making to seek care, and increase the use of Sustainable Emergency Referral Care (SERC). | Code: Transportation  SERC was designed as a low-cost emergency transportation and communication system with community education activities. The program aimed to facilitate rapid transport of patients from their community locations or subdistrict health centre to higher levels of care and used 3-wheeled motorcycles driven by trained community volunteers to transport patients. Delivery referrals were redirected from health centres to hospitals capable of advanced services, including cesarean deliveries. | Volunteers, health workers, and district supervisors completed monthly SERC monitoring to help assess both referral volume by location and the types of cases associated with referral operations. Monitoring included station-specific information on distances travelled, transit times, adherence to protocols, types of emergency, and patient outcomes. A survey was administered to health workers by trained professional interviewers in December 2013 to assess their perspectives on SERC components and challenges. |
| Goldstein, 2003, Canada | Cross-sectional | To determine the effect of an educational tool on the frequency of contraceptive counselling by resident physicians. | Code: Healthcare Provider Training  Resident physicians were instructed to complete a checklist of female patients presenting to the clinic to determine their eligibility for counselling. If they were eligible, contraceptive counselling was provided by the resident using an educational table outlining the available contraception forms. | The rate of contraceptive counselling was reported with an ICD-10 code. |
| Febrile Illness | | | | |
| Castellani, 2016, Burkina Faso, Nigeria and Uganda | Uncontrolled before/after | To quantify the time community health workers (CHWs) spent providing healthcare before and during implementing an integrated program of diagnosis and treatment of febrile illness in 3 African countries. | Code: Training of Lay Community Members  CHWs were trained to assess and manage febrile patients in keeping with Integrated Management of Childhood Illness recommendations to use rapid diagnostic tests, artemisinin-based combination therapy, and rectal artesunate for malaria treatment. All CHWs provided healthcare only to young children, usually <5 years of age. | Daily time allocation of their time to child healthcare was documented for one day (in the high malaria season) before the intervention and at several time points following the implementation of the intervention. Time spent providing child healthcare was valued in the earnings of persons with similar experiences. |
| Health System Performance | | | | |
| Farmer, 2011, United Kingdom | Cohort | To evaluate the impact and contribution made by PAs to delivering effective health care in National Health Service (NHS) Scotland. | Code: Extending Scope of Practice - Non-FP  The medical home team consisted of a primary care physician and office staff, the child and the family, a nurse practitioner (NP), and a parent consultant (a paid family member of a child with special health care needs). The intervention focused on providing care coordination, information about resources and services, emotional support and encouragement, and empowerment for families to advocate for their children. To accomplish these goals, every participant received from the NP a set of essential services that included a home visit to conduct a comprehensive assessment of medical and nonmedical needs of the child and family members, a personalized letter that described health, educational, and community resources for meeting these needs; an individualized written health plan for the child; assistance in developing short-term family goals; and at least one follow-up to discuss progress toward goals and to problem solve about any barriers to needed care. In addition, the parent consultant assisted with family-to-family support as needed. The intervention lasted six months from the first home visit, with periodic follow-ups in the second 6 months. | Outcomes were assessed at the first visit and after completion of the program. Outcomes included family demographics, child health services (including current health service needs, health service utilization and parental satisfaction with services), family functioning, child functioning, and program acceptability. |
| Human Immunodeficiency Virus | | | | |
| Naidoo, 2018, South Africa | Cross-sectional | To measure fidelity to HIV programme implementation as provided by community health workers from the community's perspective. | Code: Training of Lay Community Members  Community health workers worked under the supervision of a nurse to provide services of health education to prevent HIV infection, identifying individuals that need to test for HIV, referring HIV-infected individuals not yet in care to start antiretroviral therapy (ART), providing adherence support to those on ART, tracing and referral of HIV-infected individuals that have been lost to the ART programme, and identifying individuals who have clinically failed on ART and require further assessment. | Implementation fidelity was assessed by measuring content, coverage, frequency, and duration with the provincial guidelines that are currently in place. Coverage, 'reach', as measured by the proportion of households that reported CHW visits ever. Frequency was assessed by determining the proportion of households that received CHW visits according to the required schedule (at least once a month in the case of vulnerable households). Duration of implementation refers to the need for ongoing service delivery, i.e., no major programme interruptions; defined duration as high in case of CHW visit < 6 months ago or low for CHW visit > 6 months ago. The content was measured by the proportion of individuals reporting HIV services delivered by CHWs that the researchers deemed aligned with the guidelines, including HIV health education and referrals. |
| Lifson, 2017, Ethiopia | Uncontrolled before/after | To evaluate the implementation of a community support worker program to reduce loss of follow-up for HIV-infected patients in rural Ethiopia. | Code: Extending Scope of Practice - Non-FP  Patients were assigned 1 of 13 trained community health support workers (CHSWs) who were HIV positive and from the same neighbourhood/village. The CHSWs provided HIV and health education, counselling/social support, and facilitated communication with the HIV clinics. | Changes in client knowledge, perceived social support, self-reported quality of life (QOL), and feelings of internalized stigma over one year were measured and analyzed. Additionally, clinical and patient care outcomes were abstracted from clinic records at baseline, six months and 12 months. Variables measured included dates of clinic visits, transfers of care, cause of death (if applicable), and CD4 counts. |
| Integrated Care | | | | |
| Peterson, 2017, United States | Uncontrolled before/after | To demonstrate the value of an integrated behavioural health program within primary care practices and to evaluate the financial viability of an integrated care model in a rural setting. | Code: Extending Scope of Practice - Non-FP  Three Behavioral Health Providers (BHPs), the clinic physicians, and the administration received training in an integrated care model. The BHPs were expected to work in a brief solution-focused model, using warm hand-offs with their schedules built for 20-minute appointments. It was expected that patients would average three sessions per episode of care. All behavioural health providers completed a 40-hr "boot camp" training to ensure sufficient provider training and understanding of the model. The training included a comprehensive overview of the behavioural health model outlined by Robinson and Reiter (2007), program Startup, interdisciplinary communication and working effectively as part of a multidisciplinary team, and evidence-based interventions for common behavioural health and medical presentations within a primary care setting. Following the training, providers received in-clinic support during their initial two weeks of startup and continued to attend monthly consultation meetings emphasizing model adherence and best practices. | Changes in the number of medical claims measured effectiveness (i.e., reduction of medical claims). Healthcare utilization included primary care visits, inpatient speciality care, outpatient speciality care, emergency care, ambulance use, and lab and facility charges. This data was pulled before the intervention and for the six months following the patients' respective episodes of care with the BHP. This data was also used to assess the reach of the intervention. |
| Farmer, 2005, United States | Uncontrolled before/after | To evaluate the feasibility and impact of a medical home demonstration project in a rural population. | Code: Coordination/Referral Pathways  The medical home team consisted of a primary care physician and office staff, the child and the family, a nurse practitioner (NP), and a parent consultant (a paid family member of a child with special health care needs). The intervention focused on providing care coordination, information about resources and services, emotional support and encouragement, and empowerment for families to advocate for their children. To accomplish these goals, every participant received from the NP a set of essential services that included a home visit to conduct a comprehensive assessment of medical and nonmedical needs of the child and family members, a personalized letter that described health, educational, and community resources for meeting these needs; an individualized written health plan for the child; assistance in developing short-term family goals; and at least one follow-up to discuss progress toward goals and to problem solve about any barriers to needed care. In addition, the parent consultant assisted with family-to-family support as needed. The intervention lasted six months from the first home visit, with periodic follow-ups in the second 6 months. | Outcomes were assessed at the first visit and after completion of the program. Outcomes included family demographics, child health services (including current health service needs, health service utilization and parental satisfaction with services), family functioning, child functioning, and program acceptability. |
| Malaria | | | | |
| Siribie, 2016, Burkina Faso, Nigeria, and Uganda | Cross-sectional | To report on the training and performance of community health workers in applying HIV care recommendations. | Code: Training of Lay Community Members  The intervention involved choosing community members to act as community healthcare workers to aid in treating malaria. Community healthcare workers underwent extensive training that covered diagnosing malaria, treating malaria (including using indicators or tools like respiratory rates or thermometers), counselling caregivers posttreatment, documentation, follow-up visits, and referral advice for immediate transit. The training comprised practical sessions with theory, including demonstrations, role play and discussion sessions. | The training was evaluated via pre and post-tests using a standardized questionnaire and through observation of practice on patients by facilitators of training sessions. |
| Maternal & Child Health | | | | |
| Okonofua, 2022, Nigeria | Uncontrolled before/after | To determine the effectiveness of a set of multifaceted interventions designed to increase rural women's access to antenatal, intrapartum, postpartum and childhood immunization services offered in primary healthcare facilities. | Code: Healthcare Provider Training + Coordination/Referral Pathways + Transportation + Increasing Staff Resources  Seven community-led interventions were implemented over 27 months, consisting of a community health fund, engagement of transport owners on emergency transport of pregnant women to primary health centres with the use of rapid short message service (SMS), drug revolving fund, community education, advocacy, retraining of health workers and provision of essential equipment. | The outcome measures included the number of women who used the primary health centres for skilled pregnancy care and immunization of children aged 0–23 months. |
| Jabbari Beyrami, 2019, Iran | Interrupted Time Series | To assess the impact of the Family Physician programme on maternal and child health (MCH) indicators in 20 years in Iran. | Code: Financial Incentive + Increasing Staff Resources + Expanded Scope of Practice – Non-FP  This intervention comprised a medical, maternal, and childcare services package and aimed to make healthcare services affordable through a new insurance plan. The team structure of health centres was expanded to include additional staff, such as a general physician, midwives and a pharmaceutical technician. One family physician was settled in each village. A new insurance plan (Rural Health Insurance) was also scaled up to cover the whole Iranian rural population. GP services were increased to round-the-clock service, with increased GPs available in each area. GP salaries and services were increased to include special antenatal and postnatal care, laboratory tests (including measurement of haemoglobin (Hb) levels to monitor iron supplementation plan) and routine ultrasounds to detect fetus abnormality. The FP teams also started providing maternity care for all women in rural areas. | The indicators were grouped into three categories: structure (mother's age, education, occupation and gravidity), process (number of antenatal care visits (ACVs), laboratory tests, ultrasounds and natural vaginal deliveries (NVDs)) and outcomes (maternal mortality ratio (MMR), the neonatal mortality rate (NMR), birth weight (BW), history of abortion and stillbirth, and hemoglobin level (Hb)). |
| Medication (prescribing or medication safety) | | | | |
| Kelaher, 2006, Australia | Retrospective Cohort | To report on the effectiveness of a program to supply Pharmaceutical Benefits Scheme (PBS) medicines to remote Aboriginal and Torres Strait Islander Health Services (ATSIHSs) | Code: Financial Incentive  The special PBS arrangements (SPBSAs) allowed approved ATSIHSs to order PBS medicine in bulk through local pharmacies and supply them as needed to patients on-site. The usual co-payment associated with PBS medicine is not charged, and the pharmacist remuneration structure differs. | There were six main data collection components: public submissions; interviews with government and other key stakeholders; pharmacist survey; medicine utilization and expenditure data (total use and trends in the use of certain medications of use in this population were tracked); national ATSIHS minimum dataset; and case studies of ATSIHSs. |
| Taylor, 2003, United States | RCT | To determine the effect of pharmacist-provided pharmaceutical care on adverse drug-related events. | Code: Extending Scope of Practice - Non-FP  Pharmacists provided pharmaceutical care to patients 2-3 times per week in addition to usual care. Pharmaceutical care included individualized patient education and review of medical records to determine drug compliance and complications. | Number of ED visits and hospitalizations obtained from review of medical records and patient interviews. Medication compliance was self-reported by patients, and the 36-Item Short-Form Health Survey was used to assess quality of life and mental health. Prescribing appropriateness was determined with the Medication Appropriateness Index. Clinical endpoints such as hypertension, diabetes, anticoagulation, and dyslipidemia were also reviewed. |
| Mental Health | | | | |
| Maulik, 2017, India | Uncontrolled before/after | To evaluate the feasibility and acceptability of an intervention for identifying and treating common mental health disorders. | Code: Extending Scope of Practice - Non-FP  A mental health services delivery model that leveraged technology and task sharing to facilitate identification and treatment (including following treatment guidelines) of common mental disorders (CMDs) such as stress, depression, anxiety and suicide risk in rural areas of Andhra Pradesh, India. The intervention was delivered by lay village health workers (Accredited Social Health Activists – ASHAs) and primary care doctors. An anti-stigma campaign using multi-media approaches was conducted across the villages at the project's outset. | The primary outcome was an evaluation of pre- and post-intervention mental health service utilization. Additionally, depression and anxiety scores of those who tested positive for a CMD at baseline were measured and compared post-intervention. Trained interviewers conducted a baseline survey. The survey enquired about sociodemographic details, stressors, social networks, CMD, history of mental disorders and their treatment, family history of mental disorders, and perceptions about stigma related to mental health. Process evaluation of the project was done using focus group discussions and in–depth interviews with key stakeholders. |
| Kessler, 2012, United States | Cross-sectional | To examine rates of treatment startup in 2 collaborative care settings: a rural family medicine ofﬁce and a suburban internal medicine ofﬁce. | Code: Coordination/Referral Pathways  A collaborative care pilot tracked mental health referrals and treatment initiation as part of quality improvement efforts. After the initial pilot in a family medicine practice, a second intervention was started in an internal medicine practice, providing the opportunity to report rates of attendance at initial mental health visits at a second site. In both practices, referrals for mental health services are made within the practice. The collaborative care model focuses on the clinical, operational, and ﬁnancial elements of care and planning, and ongoing quality improvement is built into implementation and operation. Model elements are drawn from best practices identiﬁed in the ﬁeld. | Outcomes included the number of patients referred by the provider, the number who scheduled an appointment and the number who attended the ﬁrst appointment. |
| Perkins, 2010, Australia | Prospective Cohort | To evaluate an innovative rural service offering comprehensive primary health care for mental health service clients. | Code: Extending Scope of Practice - Non-FP  A monthly clinic was held in a general practice to provide primary health care for clients of the community mental health team (CMHT). The CMHT was responsible for organizing client appointments and assisting clients to attend (including providing transport if needed). They also accompanied clients during their consultations. No co-payments were charged, and details of the consultation were recorded in the general practice and CMHT client records. | The primary outcomes included client utilization and clinic activity data. Data items included: client characteristics (date of birth, gender, time as a client of the CMHT, primary mental health diagnosis), number of GP Clinic attendances (by month and location) and GP management of client (diagnosis of physical health problems, client referrals for health and welfare services, and arrangements for ongoing access to GP services). Provider views of service effectiveness, possible improvements and sustainability were assessed. |
| Pomerantz, 2008, United States | Uncontrolled before/after | To provide an example of implementing a new program that enhances access to mental health care in primary care. | Code: Coordination/Referral Pathways + Reorganization of Services  They implemented a primary mental health care clinic that provides immediate access to assessment and treatment for all individuals needing mental health services, whether self-referred, identified by their primary care provider (PCP), emergency room staff, or other triage or community referral sources. The clinic relies on self-report psychometrics (patient completes on entry to the clinic) to guide assessment and treatment and to measure outcomes. Clinicians work collaboratively with psychiatrists or psychiatric nurses for diagnostic assessment and treatment plans. | Outcomes included the number of referrals, individuals seen, no-shows/cancellations, wait times, referrals to speciality clinics and speciality no-show rates compared from the 2nd quarter of 2004 to the 4th quarter of 2004. Additionally, they measured patient satisfaction with care via a questionnaire. |
| Non-Communicable Disease | | | | |
| Saleh, 2018, Lebanon | RCT | To assess the effect of low-cost mHealth tools on the accessibility to health services and improvement of health indicators of individuals with NCDs in rural areas and refugee camps in Lebanon. | Code: Coordination/Referral Pathways  The intervention involved community healthcare workers conducting outreach screenings for diabetes and hypertension. During the visits, referrals were made remotely via an online appointment system linked to the corresponding primary healthcare centre. | The primary outcome variable was compliance with appointments. Compliance was determined by a short phone survey with referred individuals (i.e., show-ups/no-shows and reasons for no-shows). |
| Pregnancy-Related | | | | |
| Gatakaa, 2019, Kenya | Cross-sectional | To investigate the effect of autonomous community medical centres on maternal and newborn care access indicators over two years of initial implementation. | Code: Implementing a New Service  A network of 16 autonomous community medical centres/kiosks were implemented to help facilitate access to maternal and newborn care services. | The primary outcomes on the setting of the last delivery, the number of visits to a health facility, and examination of the newborn post-delivery in the previous pregnancy were assessed via a questionnaire. |
| Preventative Care | | | | |
| Nagykaldi, 2017, United States | Uncontrolled before/after | To implement and evaluate a sustainable, rural community–based patient outreach model for preventive care provided through primary care practices (PCPs) in a rural county in Oklahoma. | Code: Coordination/Referral Pathways  A Wellness Coordinator (WC) working with PCPs, the county health department, the county hospital, and a health information exchange (HIE) organization helped county residents receive evidence-based preventive services. The WC used a community wellness registry connected to electronic medical records via HIE and called patients at the county level based on PCP-prioritized and tailored protocols. The registry flagged patient-level preventive care gaps, tracked outreach efforts, and documented the delivery of preventive services throughout the community. | Return on investment (ROI) for prioritized preventive services was estimated in participating organizations. Rates of preventive service delivery for selected services were calculated for a pre-implementation baseline year and the intervention year for each participating practice and the hospital. |
| Surgery | | | | |
| Zarrabian, 2017, Canada | Prospective Cohort | To determine the effect of Inter-professional Spine Assessment and Education Clinics (ISAEC) on access to surgical assessment, referral appropriateness and efficiency for patients meeting a priori referral criteria in rural, urban and metropolitan settings. | Code: Coordination/Referral Pathways  The ISAEC are a shared-care management system for LBP among primary care providers, allied health providers and specialists to deliver evidence-based LBP assessment, education and care recommendations, timely access, and support to enable patients to self-manage LBP. | The primary outcomes were the number of patients meeting surgical referral criteria, wait times for surgical assessment, surgical referral–related magnetic resonance imaging (MRI) scans and appropriateness of referral. |
