# Supplementary material for: Interventions to improve primary healthcare in rural settings: A scoping review
Source: PLoS One. 2024 Jul 11;19(7):e0305516. doi: 10.1371/journal.pone.0305516 (PMC11239038; doi:10.1371/journal.pone.0305516)
Supplement: S12 Appendix — (DOCX) [file pone.0305516.s013.docx]

**Efficiency: Wait times**

| **Author, Year, Country** | **Design** | **Aim** | **Brief Intervention description** | **Outcome measurement** |
| --- | --- | --- | --- | --- |
| Mental Health | | | | |
| Hine, 2020, United States | Uncontrolled before/after | To determine the effect of Inter-professional Spine Assessment and Education Clinics (ISAEC) on access to surgical assessment, referral appropriateness and efficiency for patients meeting a priori referral criteria in rural, urban and metropolitan settings. | Code: Coordination/Referral Pathways  The ISAEC are a shared-care management system for LBP among primary care providers, allied health providers and specialists to deliver evidence-based LBP assessment, education and care recommendations, timely access, and support to enable patients to self-manage LBP. | The primary outcomes were the number of patients meeting surgical referral criteria, wait times for surgical assessment, surgical referral–related magnetic resonance imaging (MRI) scans and appropriateness of referral. |
| Pomerantz, 2008, United States | Uncontrolled before/after | To provide an example of implementing a new program that enhances access to mental health care in primary care. | Code: Coordination/Referral Pathways + Reorganization of Services  They implemented a primary mental health care clinic that provides immediate access to assessment and treatment for all individuals needing mental health services, whether self-referred, identified by their primary care provider (PCP), emergency room staff, or other triage or community referral sources. The clinic relies on self-report psychometrics (patient completes on entry to the clinic) to guide assessment and treatment and to measure outcomes. Clinicians work collaboratively with psychiatrists or psychiatric nurses for diagnostic assessment and treatment plans. | Outcomes included the number of referrals, individuals seen, no-shows/cancellations, wait times, referrals to speciality clinics and speciality no-show rates compared from the 2nd quarter of 2004 to the 4th quarter of 2004. Additionally, they measured patient satisfaction with care via a questionnaire. |
| Surgery | | | | |
| Zarrabian, 2017, Canada | Cohort | To extend the previously implemented Autism Diagnostic Consultation program to a diverse range of clinics that provide healthcare to rural and underserved communities. | Code: Reorganization of Services + Healthcare Provider Training  Clinics were staffed with Behavioural Health Consultants (BHC), and training was provided to clinicians to confidently discuss and accurately diagnose Autism in young children within their community settings. The training involved an intensive 1.5 or 2-day workshop focusing on using clinical tools to help make diagnoses, clinical interviewing, explaining results and diagnoses to families, service recommendations, and coding processes for third-party reimbursement. Patients were given a screening questionnaire for Autism and other developmental disorders, and if they tested positive, they were scheduled for a follow-up appointment with a BHC. | The primary outcome was latency to diagnostic consultation pre and post-intervention implementation measured using the patient's medical records. |
