# Supplementary material for: Interventions to improve primary healthcare in rural settings: A scoping review
Source: PLoS One. 2024 Jul 11;19(7):e0305516. doi: 10.1371/journal.pone.0305516 (PMC11239038; doi:10.1371/journal.pone.0305516)
Supplement: S13 Appendix — (DOCX) [file pone.0305516.s014.docx]

**Efficiency: Healthcare utilization**

| **Author, Year, Country** | **Design** | **Aim** | **Brief Intervention description** | Outcome measurement |
| --- | --- | --- | --- | --- |
| Any Condition | | | | |
| Morris-Paxton, 2020, South Africa | Controlled before/after | To evaluate the use of a non-governmental organization (NGO)-supported mobile healthcare service in a remote area. | Code: Implementing a New Service  A mobile-tented clinical outreach service was implemented to assist with screening, onward referral, treatment of minor ailments and emergency medication. This service supports the most overloaded government primary healthcare clinics and serves some of the least accessible areas. | The distance travelled from the centre of the community to the nearest fixed service was assessed. Secondly, a detailed log sheet was used to record the number of people visiting the mobile NGO service and ascertain the services provided to them. |
| Myers, 2020, United States | Case Series | To develop and evaluate a community paramedicine program to better meet the needs of complex, high-utilizer patients in a rural setting. | Code: Extended Scope of Practice + Implementing a New Service  A community paramedicine program was created to train community paramedics to extend primary care outside hospital and clinic settings. Patients were referred to the program by physicians. Community paramedics conducted home visits to assess patients' health status, review medical history, medication reconciliation, physical exams, conduct environment assessments, and complete specific physician requests (e.g., testing glucose, wound care, etc.) | The primary end-point was to analyze whether changes in utilization type (ED, primary care, and hospitalizations) occurred by implementing CP visits. |
| Moe, 2019, Canada | Cross-sectional | To examine patients' perceptions of care outcomes following the introduction of collaborative teams into community family practices. | Code: Reorganization of Services + Increasing Staff Resources  A collaborative team model was implemented, adding nonphysician healthcare professionals into member clinics, including nursing, pharmacy, social work, and mental health professionals. Additionally, the proactive office encounter technician (POET) role  was created who were trained medical office assistants or licensed practical nurses who prepare prompts displayed in the electronic medical record for the attending physician at the time of the office encounter. Westview Primary Care Network's decentralized model allows site-specific implementation and clinic-level governance of the contractual relationships between clinics and collaborative teams. The network provides overarching guidelines and vision for the program and operational support (e.g., contract templates, orientation, training). | The Primary Care Assessment Tool (PCAT) was used to evaluate standardized primary care delivery domains. Between-year changes were compared. |
| Kramer, 2018, United States | Cohort | To assess the change in access to Veterans Affairs long-term care benefits and utilization for rural populations after the expansion of the home-based primary care program to rural communities. | Code: Reorganization of Services  Home-based primary care is a VA benefit that provides patients with interdisciplinary, noninstitutional long-term care for veterans with complex chronic, disabling conditions. | The primary outcomes were activities of daily living (ADL), previous VA enrollment, hospital admissions and emergency department (ED) visits as a function of time, accounting for IHS beneficiary and functional statuses. |
| Any Condition (Elderly) | | | | |
| deBattle, 2021, Spain | Controlled before/after | To assess the effectiveness and cost-effectiveness of implementing a mobile health (mHealth)-enabled integrated care model for elderly, complex chronic patients. | Code: Reorganization of Services  The CONNECARE organizational integrated care model provided patients with coordinated interdisciplinary care with an eHealth platform supporting it, consisting of a patient self-management app, a set of integrated sensors, and a web-based platform connecting professionals from different settings. | The primary outcomes were changes in health status (Short-Form Survey), unplanned visits and admissions during a 6-month follow-up, and the incremental cost-effectiveness ratio (ICER). |
| Asthma | | | | |
| Cowan, 2004, Canada | Controlled before/after | To determine the impact of a regional asthma education centre on reducing asthma-related morbidity and improving the quality of prescribing. | Code: Patient Education/Navigation  An accredited asthma teaching centre was implemented in a community health centre to educate patients with severe, moderate and mild asthma. The centre was staffed by two nurses and supervised by a physician. Patients with asthma are referred to the asthma teaching program by their physicians. The first educational session usually lasted from 60 min to 90 min. Repeat sessions last 30 to 45 min and are scheduled based on perceived needs by the patient and nurse educator. Topics covered include mechanisms of asthma, triggers and inducers, goals of asthma treatment, medication use, side effects, and inhaler technique. | The number of emergency department (ED) visits for respiratory-related illness and prescribing anti-asthmatic medications were monitored during consecutive 18-month pre- and postintervention periods in two communities with similar health care resources. The quality of prescribing was assessed by calculating the ratio of inhaled corticosteroids to inhaled, short-acting beta2-agonists. |
| Chronic Disease | | | | |
| Mitton, 2007, Canada | Uncontrolled before/after | To implement and evaluate a collaborative partnership between homecare nurses and family physicians for the chronically ill. | Code: Coordination/Referral Pathways  Patients were enrolled in a shared care plan developed by the physician and nurse (along with other allied health professionals) and then implemented by the nurse. The plan included comprehensive biopsychosocial assessment, early intervention, health education and self-management. | Quantitative and qualitative findings related to patient, system and provider outcomes, and health system costs (i.e., direct payer) for the 12 months pre- and post-enrolment. Quantitative data examined the incremental resource impact between the two models of service delivery, including the nurses' salaries and differences in service use (physician visits, emergency department use, hospitalizations, referrals, and medications and equipment) due to changing practice patterns. Qualitative data was collected via semi-structured interviews with health providers working with the physicians, patients and primary caregivers at six months and 12 months to explore descriptions and understandings of the partnership's structures, processes and impacts. |
| Dental Care | | | | |
| Dahlberg, 2019, United States | Uncontrolled before/after | To determine if implementing fluoride varnish (FV) application to young children by providers in a rural primary care office was feasible. | Code: Expanding Scope of Practice – FP  Primary care providers at the clinical site participated in an educational session that included information on fluoride varnish application. Clinics were provided with a resource notebook containing national guidelines on FV application, recent journal publications regarding FV in primary care, and third-party reimbursement information. The nursing staff was provided with FV patient education handouts and stickers to give to pediatric patients who received FV. | The number of FV applications, time spent on procedure, perceived barriers, and overall cost. |
| Health System Performance | | | | |
| Burdick, 2022, United States | Uncontrolled before/after | To increase the use of Transitional Care Management (TCM) services in primary care to prevent hospital readmissions. Specifically, to increase TCM phone calls, office visits, and use of the TCM billing codes, as well as to spread the use of TCM tools across all clinics. | Code: Coordination/Referral Pathways +  Reorganization of Services  An intervention was implemented to improve the utilization of Transitional Care Management (TCM), a reimbursable service designed to minimize hospital readmissions. It involved a learning health system model, an improvement support team (IST), and a learning collaborative that included webinars and in-person support. The process emphasized user-centred system redesign, coaching, electronic health record (EHR) improvements, and real-time feedback. | The primary outcomes were related to TCM utilization: (1) TCM Episodes documented in the electronic health records, (2) TCM Calls, and (3) TCM Visits. Primary care providers' use of TCM billing codes and hospital readmissions was also measured. |
| Gur, 2020, Israel | RCT | To assess the impact of predefined interventions on reducing foci of waste in the clinic workflow. | Code: Well-being  The foci of waste and acceptable interventions were determined and implemented by clinic staff. The interventions focused on reducing wait times (e.g., having patients see the next available physician), clear labelling of exam rooms to avoid confusion, the addition of extra workstation to increase the efficacy of uploading patient files to EMR, using a word processor to document to prevent loss of data, and moving the printer to increase the efficiency of documentation. | The primary outcome was defined as the average total value stream duration. Secondary outcomes included physician's productivity (measured by the average number of appointments per working hour) and burnout (assessed by the Maslach Burnout Inventory), patient satisfaction (assessed using a scale of 1–6), the proportion of patients returning to the clinic within 30 days, quality of care (assessed using the Israel Defense Forces Medical Corps "tracers of quality medical care "score). |
| Nagykaldi, 2020, United States | Uncontrolled before/after | To implement and evaluate a sustainable, rural community-based patient outreach model for preventive care provided through primary care practices located in 3 rural counties in Oklahoma. | Code: Coordination/Referral Pathways + Implementing a New Service  The wellness coordinators used a wellness registry connected to electronic medical records and health information exchange networks and called patients at the county level based on primary care practitioner–preferred protocols. The registry flagged patient-level preventive care gaps, tracked outreach efforts, and documented the delivery of services throughout the community. | The study team estimated participating organizations' return on investment (ROI). The Preventive Services Reminder System tracked preventive service delivery, practice electronic medical records, health system databases, and health information exchanges and was measured pre and post-intervention. |
| Pu, 2020, China | Uncontrolled before/after | To evaluate a pre/post-reform pilot study from a rural county of Zhejiang Province, China, to realign the provider payment system for primary health care (PHC). | Code: Financial Incentive  The reimbursement framework consisted of two levels. At the facility level, a mixed system of input-based (line-item budget) and categorized output-based payments was launched to meet balanced equity and efficiency objectives. At the individual level, a basic salary plus a bonus based on performance was given to incentivize PCPs. | The differences in total salaries among independent groups (central towns and streets, sparsely populated rural towns, and others) and differences in annual income between permanent and temporary employees were analyzed. The total volume of inpatient visits, outpatient visits, and the National Essential Public Health Services Package (NEPHSP) was measured. |
| Kranker, 2018, United States | Cohort | To evaluate the impacts of a telephonic transitional care program on service use and spending for Medicare fee-for-service beneficiaries at a rural hospital in the US. | Code: Coordination/Referral Pathways  Involved assigning patients who have been discharged from the Atlantic General Hospital (AGH) and have an AGH primary care provider were a nurse care coordinator for 30 days. The nurse provided care coordination by reviewing the patient's condition, assessing the need for transition support, weekly telephone calls to monitor adherence to treatment plans, and scheduling follow-up appointments. | Claims data were used to evaluate impacts on service use and spending using a difference-in-differences design with a matched comparison group. |
| Liddy, 2017, Canada | Cross-sectional | To describe the use of the Champlain BASETM (Building Access to Specialists through eConsultation) service in Nunavut and conduct a cost evaluation. | Code: Coordination/Referral Pathways  The Champlain BASETM (Building Access to Specialists through eConsultation) service facilitates asynchronous communication between primary care providers (PCP) and specialists. PCPs can log onto the web-based platform and enter their questions for specialists and pertinent clinical information into the appropriate fields. Specialists are asked to respond within seven days and may request more information through the platform. | The service automatically collected primary outcomes on utilization data, including the speciality group referred to; the dates of case submission, length of time to receive an initial response from specialists, and case closure; and specialists' self-reported time spent answering the case. Data on case outcomes were obtained from a mandatory closeout survey completed by PCPs, which consists of multi-choice questions and free-text field questions to capture information regarding eConsult's impact on PCPs' course of action, whether or not a face-to-face referral was avoided, and PCPs' opinions regarding the value of the eConsult for the patient and themselves. |
| Baldwin, 2014, United States | Uncontrolled before/after | To describe a cost-avoidance transitional care mode using a clinical nurse specialist to prevent readmissions of uninsured and underinsured patients. | Code: Reorganization of Services  The intervention involved d a community nursing case management program to decrease preventable readmissions to the hospital and emergency department by providing telephonic case management and, if needed, onsite assessment and treatment by a clinical nurse specialist (CNS) with prescriptive authority. | Outcomes measured included hospital and emergency department admissions. |
| Rashidian, 2013, Iran | Interrupted Time Series | To assess the effects on hospital utilization rates of a significant health system reform – a family physician programme and a social protection scheme | Code: Reorganization of Services  The intervention was to improve rural households' access to outpatient and hospital care through a social protection scheme whereby paying a nominal fee, participants gained access to services at a reduced cost. | Hospitalization rates were calculated pre and post-intervention. |
| Hussain, 2012, Australia | Cross-sectional | To investigate whether increased numbers of primary healthcare clinical consultations in Indigenous communities in some remote areas of Australia are associated with the reduced need for urgent medical evacuations and remote telephone consultations. | Code: Reorganization of Services  Conducted a retrospective comparison study of service data. | Main outcome measures included the number of acute medical evacuations and remote telephone consultations relative to the number of face-to-face consultations with Aboriginal health workers, remote area nurses and general practitioners. |
| Human Immunodeficiency Virus | | | | |
| Hontelez, 2016, South Africa | Retrospective Cohort | To establish the population-level impact of antiretroviral treatment (ART) programs on health care utilization in the public-sector health system. | Code: Reorganization of Services  Compared trends in health care utilization among HIV-infected people receiving and not receiving ART with HIV-uninfected people during a rapid ART scale-up. | The primary outcomes were indicators of healthcare utilization. These included: the number of hospital visits in the last 12 months, whether a person visited a public-sector PHC clinic in the past six months, and whether an individual visited a private-sector PHC clinic in the last six months. Additionally, they tracked the ART status of all individuals in the study. |
| Integrated Care | | | | |
| Peterson, 2017, United States | Uncontrolled before/after | To demonstrate the value of an integrated behavioural health program within primary care practices and to evaluate the financial viability of an integrated care model in a rural setting. | Code: Extending Scope of Practice - Non-FP  Three Behavioral Health Providers (BHPs), the clinic physicians, and the administration received training in an integrated care model. The BHPs were expected to work in a brief solution-focused model, using warm hand-offs with their schedules built for 20-minute appointments. It was expected that patients would average three sessions per episode of care. All behavioural health providers completed a 40-hr "boot camp" training to ensure sufficient provider training and understanding of the model. The training included a comprehensive overview of the behavioural health model outlined by Robinson and Reiter (2007), program Start-up, interdisciplinary communication and working effectively as part of a multidisciplinary team, and evidence-based interventions for common behavioural health and medical presentations within a primary care setting. Following the training, providers received in-clinic support during their initial two weeks of start-up and continued to attend monthly consultation meetings emphasizing model adherence and best practices. | A reduction in medical claims measured effectiveness. Healthcare utilization included primary care visits, inpatient speciality care, outpatient speciality care, emergency care, ambulance use, and lab and facility charges. This data was pulled before the intervention and for the six months following the patients' respective episodes of care with the BHP. This data was also used to assess the reach of the intervention. |
| Rebello, 2017 United States | Controlled before/after | To evaluate the efficacy of the Rural Pharmacological Intervention in Late Life (PILL) program. | Code: Reorganization of Services + Decision Support  A quality improvement initiative in which a Boston-based pharmacist provided post-discharge telepharmacy care to veterans. Using an automated screening tool, they identified 100 veterans aged 65 and older who had an acute care admission to VA medical centres and were at risk of problems with medication management. The PILL pharmacist called patients the week after hospital discharge to reconcile medications, assess adherence, and identify potentially inappropriate drugs. The pharmacist worked with each veteran's family and providers to resolve problems and increase support. | To determine whether the intervention decreased acute care admissions, rehospitalizations, or deaths, we matched one unique control to each PILL patient by age, hospital location, length of stay, admitting service, and reason for admission. |
| Brock-Martin, 2007, United States | Controlled before/after | To evaluate the effectiveness of a pilot project providing a medical home to children with special health care needs (CSHCN) on preventative and emergency care utilization. | Code: Increasing Staff Resources  The medical home intervention started with hiring a care coordinator (a licensed social worker experienced in working with CSHCN) and a mother of a child with special needs for administrative help. The practice employs two physicians, who also serve as faculty in a rural residency program, with up to 27 resident physicians rotating each year. Numerous changes were implemented to support better care for CSHCN, such as colour-coordinated charts for children with special needs, the development of a resource manual, and care coordination meetings. | Outcomes for the cohort of CSHCN were measured one year before and two years after participation in a medical home intervention. Outcomes included the number of hospitalizations, length of hospital stay, emergency room visits, and annual "early and periodic screening, diagnosis, and treatment" visits. |
| Mental Health | | | | |
| Fisher, 2017, United States | Cohort | This study assesses whether Project ECHO (Extension for Community Healthcare Outcomes) GEMH (geriatric mental health)—a remote learning and mentoring program—is an effective strategy to address geriatric mental health challenges in rural and underserved communities. | Code: Healthcare Provider Training  They implemented a Project ECHO geriatric mental health (GEMH) hub connecting a team of specialists (geriatric psychiatry and medicine, nursing, social work, psychology, and pharmacy) to spokes of primary care and social service sites. The curriculum consisted of case presentations and didactic lessons that provided participants with information on screening, treatment, and diagnosis of geriatric mental health conditions, focusing on issues faced by older adults related to depression, anxiety, and dementia. Didactic portions of the clinics focused on education and best treatment practices for medication therapies, behavioural interventions, social services, caregiver support, and sleep hygiene. | This study examined changes in participants' geriatric mental health care knowledge, confidence, and treatment practices to understand the program's short-term impact. It also examined satisfaction with the program and obtained health insurance claims data from a private payer to assess changes in health care utilization and costs before and after the implementation of Project ECHO GEMH. |
| Sutor, 2007, United States | Controlled before/after | To examine health care utilization patterns of patients with depression and explore the effect psychiatric consultation and collaborative care have on these patients' overall usage. | Code: Coordination/Referral Pathways  They implemented a program that provides psychiatric consultation, follow-up, and collaboration for physicians and trainees in a rural family medicine clinic. Patients referred for psychiatric evaluation are seen in the clinic and are often introduced to the psychiatrist by the referring clinician. Ongoing care is then shared by the psychiatrist and family physician, depending on the patient's needs. Patients with more severe psychiatric illness, medical comorbidity, or treatment-refractory illness might be seen more frequently by the psychiatrist; those with less severe illness or who respond quickly might be seen by the psychiatrist for consultation only, with all additional follow-up provided by a family physician. | Ambulatory visits and inpatient hospital days were compared for 49 patients with depression and 49 matched patients (age/gender/ severity of co-morbid conditions) who were not depressed. Use of health care services was measured one year before and one year after intervention and compared with that of the control patients. |
| Noncommunicable Diseases | | | | |
| Pati A, 2021, India | Quasi-Randomized Cluster Trial | To examine whether interventions aimed at health service optimization alone or combined with community platform strengthening improve access to medicines at the primary health care level within a local health system. | Code: Healthcare Provider Training + Coordination/Referral Pathways  Arm A: A package of interventions included training of PHC staff (doctors, pharmacists, laboratory technicians, nurses) on standard treatment protocols for the diagnosis and management of diabetes and hypertension, introduction of patient-retained medical records and primary health care ({PHC)-based records for registration, follow-up of diabetes and hypertension patients, advocacy and coordination at the state, district and taluka levels to ensure a continuous supply of medicines to the PHCs. | Primary outcomes of the study are the mean number of days of availability of antidiabetic and antihypertensive medicines at primary healthcare centres, the mean number of patients obtaining medicines from PHC and out-of-pocket expenses. |
| Pati B, 2021, India | Quasi-Randomized Cluster Trial | To examine whether interventions aimed at health service optimization alone or combined with community platform strengthening improve access to medicines at the primary health care level within a local health system. | Code: Healthcare Provider Training + Coordination/Referral Pathways + Patient Education/Navigation  Arm B: Included development and dissemination of awareness materials, formation of patient groups, and meeting with Arogya Raksha Samiti (ARS) members on matters related to diabetes and hypertension care in addition to all interventions of Arm A. | Primary outcomes of the study are the mean number of days of availability of antidiabetic and antihypertensive medicines at primary healthcare centres, the mean number of patients obtaining medicines from PHC and out-of-pocket expenses. |
| Palliative Care | | | | |
| vandeMortel, 2017, Australia | Controlled before/after | To examine the feasibility of using a GP registrar (GPR) to facilitate communication among palliative care specialists, consumers and GPs, and provide risk assessment, care planning and continuity of care. | Code: Implementing a New Service  Intervention group participants received the GPR service, which involved liaison among the patient, family, General Practitioner, specialist palliative care team and community nurses. Specified risk assessment, care planning and continuity of care were provided. The GPRs conducted the initial patient assessment and case conference with the medical and nursing teams and the family to develop the care plan. They also conducted a 3-month follow-up (for stable patients) or re-assessed and updated the plan if the patient deteriorated. | Hospital admissions per 100  patient-days, bed-days per 100 patient-days and proportion of deaths at home. |
| Carey, 2017, Australia | Controlled before/after | To determine the impact of establishing a palliative and chronic disease respite facility on the extent to which patients accessed hospital resources. | Code: Reorganization of Services  The respite facility was staffed by nursing staff, which enabled patients attending the facility to have some of their medical needs attended to. While at the facility, participants could engage in unstructured leisure and social activities such as cooking, watching television, and socializing with staff and other patients. | Respite service use and hospital use data were collected over two periods: the 12 months before the establishment of the service and the first 10 months of the operation. The National Weighted Activity Unit (NWAU, a standardized measure of hospital expenditure) was used to determine the mean cost savings. The impact of the respite service on admissions to the Emergency Department (ED), to the Wards, and the Intensive Care Unit (ICU) was measured via hospital records. The number of ventilator hours consumed was also recorded. |
| Surgery | | | | |
| Zarrabian, 2020, China | Prospective Cohort | To determine the effect of Inter-professional Spine Assessment and Education Clinics (ISAEC) on access to surgical assessment, referral appropriateness and efficiency for patients meeting a priori referral criteria in rural, urban and metropolitan settings. | Code: Coordination/Referral Pathways  The ISAEC are a shared-care management system for LBP among primary care providers, allied health providers and specialists to deliver evidence-based LBP assessment, education and care recommendations, timely access, and support to enable patients to self-manage LBP. | The primary outcomes were the number of patients meeting surgical referral criteria, wait times for surgical assessment, surgical referral–related magnetic resonance imaging (MRI) scans and appropriateness of referral. |
