# Supplementary material for: Interventions to improve primary healthcare in rural settings: A scoping review
Source: PLoS One. 2024 Jul 11;19(7):e0305516. doi: 10.1371/journal.pone.0305516 (PMC11239038; doi:10.1371/journal.pone.0305516)
Supplement: S14 Appendix — (DOCX) [file pone.0305516.s015.docx]

**Efficiency: Cost and cost effectiveness**

| **Author, Year, Country** | **Design** | **Aim** | **Brief Intervention description** | **Outcome measurement** |
| --- | --- | --- | --- | --- |
| Any Condition (Elderly) | | | | |
| deBattle, 2021, Spain | Controlled before/after | To assess the effectiveness and cost-effectiveness of implementing a mobile health (mHealth)-enabled integrated care model for elderly, complex chronic patients. | Code: Reorganization of Services  The CONNECARE organizational integrated care model provided patients with coordinated interdisciplinary care with an eHealth platform supporting it, consisting of a patient self-management app, a set of integrated sensors, and a web-based platform connecting professionals from different settings. | Primary outcomes were changes in health status (Short-Form Survey), unplanned visits and admissions during a 6-month follow-up, and the incremental cost-effectiveness ratio (ICER). |
| Atrial Fibrillation | | | | |
| Orchard, 2020, Australia | Cross-sectional | To improve the proportion of patients screened and treated for atrial fibrillation (AF) using the refined eHealth tools and to inform strategies on AF screening implementation in the rural setting. In addition, this study provides the first cost-effectiveness analysis in Australian general practice. | Code: Decision Support + Telehealth or Virtual Care  General practitioners/nurses at practices in rural Australia screened eligible patients (≥65 years of age without AF) using a smartphone ECG during practice visits. eHealth tools included electronic prompts, guideline-based electronic decision support, and regular data reports. | Primary outcomes were the proportion of screened patients with confirmed new AF, the proportion of AF and screened patients where the electronic decision support was accessed, the proportion of patients with AF who were prescribed an OAC according to guidelines, baseline AF prevalence in patients ≥65 years of age compared with metropolitan and nonrandomized control groups, new screen-detected AF incidence at the end of the study period in patients ≥65 years of age compared with the metropolitan and nonrandomized control groups, rates of OAC and antiplatelet treatment at baseline and completion for patients in the OAC recommended category compared with the metropolitan and nonrandomized control groups. The economic model developed in the SEARCH-AF (Screening Education and Recognition  in Community Pharmacies of Atrial Fibrillation)20  pharmacy screening study was adapted to evaluate the cost-effectiveness of the iECG screening in general practice. |
| Cancer | | | | |
| Denewer, 2010, Egypt | Cross-sectional | To evaluate the disease pattern and the screen-detected breast cancer rate and determine the effectiveness of clinical breast assessment-based screening (clinical examination and history taking with selective diagnostic breast imaging). | Code: Screening  Clinical breast assessment-based screening. Stage 1: a detailed questionnaire to identify at-risk women. Stage 2: Depending on the woman's age, CBE and mammography with/without ultrasonography. | Cost-effectiveness of screening program and detection rate using history taking and selective imaging. |
| Cardiovascular Disease | | | | |
| Blattner, 2014, New Zealand | Uncontrolled before/after | To evaluate a cardiac exercise tolerance test (ETT) service project designed to improve access to ETT cost-effectively and not compromise standards of care. | Code: Reorganization of Services  For 12 months, from Sept 2011, a generalist-led ETT service was provided in two rural hospitals in New Zealand: Dunstan in Central Otago and Rawene in Northland. According to established guidelines, the rural hospitals drew up clinical protocols with input from the participating specialists.17 General Practitioners were advised of the referral process by letter. A standardized clinical record form was completed by the generalist for each ETT. | Patient outcomes included: ongoing GP management, referral to a cardiologist, percutaneous intervention (PCI), and Coronary Artery Bypass Grafting (CABG). Additionally, they audited the reporting of ETT by generalist doctors, determined the tangible costs and attempted to determine whether or not there was any rural-urban difference in the utilization of ETT. |
| Hippisley-Cox, 2000, United Kingdom | Controlled before/after | To evaluate the feasibility and cost-effectiveness of screening and treating hyperlipidaemia in patients with ischaemic heart disease in primary care. | Code: Coordination/Referral Pathways  The intervention involved developing management plans for hyperlipidaemia with a local consultant chemical pathologist and cardiologist. Additionally, a nurse gave patients with hyperlipidaemia dietary advice and a diet sheet. After a three-month diet trial, all patients with persistent hyperlipidaemia were asked to see their usual GP to discuss lipid-lowering drugs. Patients who did not attend for follow-up were sent a reminder. | The primary outcomes measured were costs, benefits, mood and quality of life. Costs were measured by administration costs of identifying and inviting patients for screening; data entry time; number of GP consultations; number of nurse consultations; costs of all the blood tests as specified in protocol; and costs of lipid-lowering drugs and the cost of an average 10 min consultation with a GP. Benefits were estimated using the number of patients who needed treatment to prevent cardiac events and deaths. Mood and quality of life were measured via questionnaires before and six months after treatment. |
| Chronic Disease | | | | |
| Mitton, 2007, Canada | Uncontrolled before/after | To implement and evaluate a collaborative partnership between homecare nurses and family physicians for the chronically ill. | Code: Coordination/Referral Pathways  Patients were enrolled in a shared care plan developed by the physician and nurse (along with other allied health professionals) and then implemented by the nurse. The plan included comprehensive biopsychosocial assessment, early intervention, health education and self-management. | Quantitative and qualitative findings related to patient, system and provider outcomes, and health system costs (i.e., direct payer) for the 12 months pre- and post-enrolment. Quantitative data examined the incremental resource impact between the two models of service delivery, including the nurses' salaries and differences in service use (physician visits, emergency department use, hospitalizations, referrals, and medications and equipment) due to changing practice patterns. Qualitative data was collected via semi-structured interviews with health providers working with the physicians, patients and primary caregivers at six months and 12 months to explore descriptions and understandings of the partnership's structures, processes and impacts. |
| Diabetes | | | | |
| Bray, 2005, United States | Uncontrolled before/after | This study assessed the feasibility and potential for the cost-effectiveness of restructuring care in rural fee-for-service practices for predominantly minority patients with diabetes mellitus. | Code: Patient Education/Navigation + Reorganization of Services  An advanced practice nurse visited each practice weekly for 12 months, provided intensive diabetes case management, and facilitated a 4-session group visit educational program. The nurse case manager reviewed the patient's care plan and facilitated or provided, under protocol and supervision, the contemporary diabetes care that each patient needed. This included facilitating laboratory testing as well as referrals to other providers. The case manager also facilitated a return visit schedule and worked with office staff to create a reminder and callback system for patients who missed appointments. To track patient progress, the leadership team implemented a registry system, allowing the case manager to remind providers of care standards and diabetes disease management goals. | The program's sustainability was measured regarding improved clinic productivity and billable encounters, including the proportion of patients achieving diabetes management goals, with a documented self-management goal, a documented lipid panel, current aspirin use and current foot examination. |
| Dental Care | | | | |
| Dahlberg, 2019, United States | Uncontrolled before/after | To determine if implementing fluoride varnish (FV) application to young children by providers in a rural primary care office was feasible. | Code: Expanding Scope of Practice – FP  Primary care providers at the clinical site participated in an educational session that included information on fluoride varnish application. Clinics were provided with a resource notebook containing national guidelines on FV application, recent journal publications regarding FV in primary care, and third-party reimbursement information. The nursing staff was provided with FV patient education handouts and stickers to give to pediatric patients who received FV. | The number of FV applications, time spent on procedure, perceived barriers, and overall cost. |
| Epilepsy | | | | |
| Wagner, 2021, South Africa | Markov Model | To determine whether deploying community health workers to improve epilepsy treatment adherence in rural South Africa would be cost-effective. | Code: Healthcare Provider Training + Training of Lay Community Members  Community Health Workers (CHWs) will undergo intensive and rigorous training led by provincial neurologists and district clinicians and supported by professional nurses. CHWs will visit people with epilepsy once every three months to assess adherence. During these visits, the CHW will review patient-held seizure diaries, inquire about seizure frequency, provide psychological support, and refer the patient to the primary healthcare facilities if seizures are uncontrolled or comorbidities are present. Furthermore, CHWs will regularly meet with community leaders, including school principals, to provide education on epilepsy. | Data were derived using published studies from rural South Africa. Official statistics and international disability weights provided cost and health state values, respectively, and health gains were measured using quality-adjusted life years (QALYs). |
| Ding, 2008, China | Uncontrolled before/after | To determine the cost-effectiveness and efficacy of using phenobarbital (PB) for epilepsy treatment in a rural primary care setting. | Code: Reorganization of Services  Patients received PB treatment and were followed up by rural clinics or town hospitals every two weeks for the first month and monthly after. | Efficacy was defined as the patients who became seizure-free during the last six months of follow-up. Costs were calculated for one year of the intervention. These included: drug costs, personnel, training, and operating were calculated and compared between the two study sites. An economic burden questionnaire was used to establish the costs to patients associated with contact with (and fees paid for) other healthcare services (hospital outpatient and inpatient care) at different levels of the health system (village health worker, town hospital doctor, neurologist, and hospital admission) as well as time costs associated with travel and waiting. |
| Family Planning | | | | |
| Schnippel, 2015, South Africa | Cross-sectional | To conduct a descriptive cost evaluation of two mobile reproductive and primary care clinics. | Code: Reorganization of Services  Services included cervical cancer screening, HIV counselling and testing, syndromic management of sexually transmitted infections (STIs), breast exams, provision of condoms, contraceptives, and general health education. | Fixed costs, including vehicle purchase and conversion, equipment, operating costs and mobile clinic staffing, were collected from program records and public sector pricing information. The number of women accessing different services was multiplied by ingredients-based variable costs, reflecting the consumables required. |
| Febrile Illness | | | | |
| Castellani, 2016, Burkina Faso, Nigeria and Uganda | Uncontrolled before/after | To quantify the time community health workers (CHWs) spent providing healthcare before and during implementing an integrated program of diagnosis and treatment of febrile illness in 3 African countries. | Code: Training of Lay Community Members  CHWs were trained to assess and manage febrile patients in keeping with Integrated Management of Childhood Illness recommendations to use rapid diagnostic tests, artemisinin-based combination therapy, and rectal artesunate for malaria treatment. All CHWs provided healthcare only to young children, usually <5 years of age. | Daily time allocation of their time to child healthcare was documented for one day (in the high malaria season) before the intervention and at several time points following the implementation of the intervention. Time spent providing child healthcare was valued in the earnings of persons with similar experience. |
| Health System Performance | | | | |
| Nagykaldi, 2020, United States | Uncontrolled before/after | To implement and evaluate a sustainable, rural community-based patient outreach model for preventive care provided through primary care practices located in 3 rural counties in Oklahoma. | Code: Coordination/Referral Pathways + Implementing a Service  The wellness coordinators used a wellness registry connected to electronic medical records and health information exchange networks. They called patients at the county level based on primary care practitioner–preferred protocols. The registry flagged patient-level preventive care gaps, tracked outreach efforts, and documented the delivery of services throughout the community. | The study team estimated participating organizations' return on investment (ROI). The Preventive Services Reminder System tracked preventive service delivery, practice electronic medical records, health system databases, and health information exchanges and was measured pre and post-intervention. |
| Kranker, 2018, United States | Cohort | To evaluate the impacts of a telephonic transitional care program on service use and spending for Medicare fee-for-service beneficiaries at a rural hospital in the US. | Code: Coordination/Referral Pathways  Involved assigning patients who have been discharged from the Atlantic General Hospital (AGH) and have an AGH primary care provider were a nurse care coordinator for 30 days. The nurse provided care coordination by reviewing the patient's condition, assessing the need for transition support, weekly telephone calls to monitor adherence to treatment plans, and scheduling follow-up appointments. | Using a difference-in-differences design with a matched comparison group claims data was used to evaluate impacts on service use and spending. |
| Liddy, 2017, Canada | Cross-sectional | To describe the use of the Champlain BASETM (Building Access to Specialists through eConsultation) service in Nunavut and conduct a cost evaluation. | Code: Coordination/Referral Pathways  The Champlain BASETM (Building Access to Specialists through eConsultation) service facilitates asynchronous communication between primary care providers (PCP) and specialists. PCPs can log onto the web-based platform and enter their questions for specialists and pertinent clinical information into the appropriate fields. Specialists are asked to respond within seven days and may request more information through the platform. | The service automatically collected primary outcomes on utilization data, including the speciality group referred to; the dates of case submission, length of time to receive an initial response from specialists, and case closure; and specialists' self-reported time spent answering the case. Data on case outcomes were obtained from a mandatory closeout survey completed by PCPs, which consists of multi-choice questions and free-text field questions to capture information regarding eConsult's impact on PCPs' course of action, whether or not a face-to-face referral was avoided, and PCPs' opinions  regarding the value of the eConsult for the patient and themselves. |
| Baldwin, 2014, United States | Uncontrolled before/after | To describe a cost-avoidance transitional care mode using a clinical nurse specialist to prevent readmissions of uninsured and underinsured patients. | Code: Reorganization of Services  The intervention involved a community nursing case management program to decrease preventable readmissions to the hospital and emergency department by providing telephonic case management and, if needed, onsite assessment and treatment by a clinical nurse specialist (CNS) with prescriptive authority. | Outcomes measured included hospital and emergency department admissions. |
| Lee, 2004, Korea | Cross-sectional | To analyze community health practitioner (CHP) services in primary health care and evaluate some of the economic impacts of these services through a cost-minimization analysis | Code: Reorganization of Services  A cost-minimization analysis of CHP services was investigated using a retrospective, descriptive, correlational design. A self-administered questionnaire was sent to CHPs. It consisted of demographic and locality data items, roles and activities of CHPs in primary health care settings, and costs relating to providing services. | Data was collected on CHP activities, including outpatient services, health promotion, maternal–child health, visiting nurse service, and information on the number of client visits, telephone counselling calls, and home visits. Additionally, the average cost per CHP per month and the cost of a lack of CHP services were calculated. |
| Integrated Care | | | | |
| Peterson, 2017, United States | Uncontrolled before/after | To demonstrate the value of an integrated behavioural health program within primary care practices and to evaluate the financial viability of an integrated care model in a rural setting. | Code: Extending Scope of Practice - Non-FP  Three Behavioral Health Providers (BHPs), the clinic physicians, and the administration received training in an integrated care model. The BHPs were expected to work in a brief solution-focused model, using warm hand-offs with their schedules built for 20-minute appointments. It was expected that patients would average three sessions per episode of care. All behavioural health providers completed a 40-hr "boot camp" training to ensure sufficient provider training and understanding of the model. The training included a comprehensive overview of the behavioural health model outlined by Robinson and Reiter (2007), program Startup, interdisciplinary communication and working effectively as part of a multidisciplinary team, and evidence-based interventions for common behavioural health and medical presentations within a primary care setting. Following the training, providers received in-clinic support during their initial two weeks of startup and continued to attend monthly consultation meetings emphasizing model adherence and best practices. | Effectiveness was measured by a reduction in medical claims. Healthcare utilization was measured, including primary care visits, inpatient speciality care, outpatient speciality care, emergency care, ambulance use, and lab and facility charges. This data was pulled before the intervention and for the six months following the patients' respective episodes of care with the BHP. This data was also used to assess the reach of the intervention. |
| Medication (prescribing or medication safety) | | | | |
| Riou, 2007, France | Controlled before/after | To assess the impact of a quality-circle GP prescribing improvement programme. | Code: Healthcare Provider Training  Quality circles were used in a GP prescribing improvement programme. Two public health consultants met all GPs in each area every 2.5 months to introduce independent information about various drug types and polymedications. They also provided feedback information about prescribing patterns and cost. Quality circles met every six weeks. | Outcome variables included all GPs' annual prescription cost per patient and markers of prescribing efficiency related to documentary supports. Costs were calculated per quarter, per half-year and year. Differences pre and post-intervention were calculated. |
| Mental Health | | | | |
| Basu, 2017, United States | Microsimulation Model | To assess the financial impact of integrating behavioural health services into primary care. | Code: Reorganization of Services  The intervention was a collaborative care model involving telephone-based follow-up from a behaviourist care manager or a primary care behaviourist model involving an in-clinic behaviourist. Patients referred with mental health issues are provided brief, time-limited behavioural treatments. | The primary outcomes involved measuring the net revenue change per full-time physician as a result of the addition of a behavioural therapy specialist. |
| Fisher, 2017, United States | Cohort | This study assesses whether Project ECHO (Extension for Community Healthcare Outcomes) GEMH (geriatric mental health)—a remote learning and mentoring program—is an effective strategy to address geriatric mental health challenges in rural and underserved communities. | Code: Healthcare Provider Training  They implemented a Project ECHO geriatric mental health (GEMH) hub connecting a team of specialists (geriatric psychiatry and medicine, nursing, social work, psychology, and pharmacy) to spokes of primary care and social service sites. The curriculum consisted of case presentations and didactic lessons that provided participants with information on screening, treatment, and diagnosis of geriatric mental health conditions, focusing on issues faced by older adults related to depression, anxiety, and dementia. Didactic portions of the clinics focused on education and best treatment practices for medication therapies, behavioural interventions, social services, caregiver support, and sleep hygiene. | This study explored changes in participants' geriatric mental health care knowledge, confidence, and treatment practices to understand the program's short-term impact. It also examined satisfaction with the program and obtained health insurance claims data from a private payer to assess changes in health care utilization and costs before and after the implementation of Project ECHO GEMH. |
| Pyne A, 2015, United States | RCT | To examine the cost-effectiveness of onsite practice-based collaborative care (PBCC) versus off-site telemedicine-based collaborative care (TBCC) for depression in federally qualified health centres (FQHCs). | Code: Decision Support + Healthcare Provider Training + Increasing Staff Resources  Practice-based collaborative care involves two types of providers: onsite PC providers and onsite nurse depression care managers (DCMs). Each clinic location employed a half-time DCM funded by the study. All DCMs received one day of training in depression care management, a care manager training manual, and access to a Web-based decision support system. Depending on patient preference encounters with a DCM were conducted face-to-face or by telephone. The initial encounter with the DCM included PHQ-9 symptom monitoring, education and self-management behavioural activation, barrier assessment and resolution, and establishing self-management goals, such as planning physical, rewarding, and social activities. Follow-up encounters included monitoring symptoms with the PHQ-9, medication adherence, side effects, and engagement in planned self-management activities. | Primary effectiveness outcomes for cost-effectiveness analysis were depression-free days and quality-adjusted life years (QALYs). Depression outcomes of Depression treatment response, remission, and severity were compared between groups using the Symptom Checklist. |
| Pyne B, 2015, United States | RCT | To examine the cost-effectiveness of onsite practice-based collaborative care (PBCC) versus off-site telemedicine-based collaborative care (TBCC) for depression in federally qualified health centres (FQHCs). | Code: Decision Support + Healthcare Provider Training + Increasing Staff Resources + Telehealth or Virtual Care + Coordination/Referral Pathways  TBCC involved five types of providers: onsite PC providers and off-site DCM (a registered nurse), clinical pharmacist (Pharm.D.), psychologist (PhD), and psychiatrist (M.D.). All encounters between DCMs and patients were conducted by telephone and followed the protocol described above. The DCM met weekly with the  psychiatrist to discuss clinical cases and treatment recommendations. These notes were faxed to the FQHC for implementation by the PC providers. If the patient did not respond to the initial antidepressant, the off-site pharmacist conducted a medication history and provided medication management recommendations as needed. A psychiatry consultation via interactive video was scheduled if the patient did not respond to two trials. Patients had access to cognitive-behavioural therapy delivered via interactive video at any time. | Primary effectiveness outcomes for cost-effectiveness analysis were depression-free days and quality-adjusted life years (QALYs). Depression outcomes of Depression treatment response, remission, and severity were compared between groups using the Symptom Checklist. |
| Osteoarthritis | | | | |
| Nelson, 2014, United States | Markov Modeling | To evaluate the cost-effectiveness of training rural primary care providers to perform knee injections in community-based outpatient clinics (CBOCs). | Code: Healthcare Provider Training  Developed a decision-analysis model to compare costs and outcomes of hypothetical patients seen by rural providers who are trained to perform knee injections versus those patients seen by providers who are not trained. | The primary outcomes were costs and effectiveness. The costs from the perspective of the VA included the direct medical costs to perform the knee injection, the mini-residency training program, and travel reimbursement given to qualified patients. All costs were converted to 2011 US dollars. The effectiveness outcome was quality-adjusted life years. |
| Osteoporosis | | | | |
| Nelson, 2014, United States | Markov Modeling | To determine the cost-effectiveness analysis of training rural providers to identify and treat osteoporosis. | Code: Healthcare Provider Training  A Markov microsimulation model was used to compare the costs and outcomes of a hypothetical cohort of veterans seen by rural providers. Parameter estimates were derived from previously published studies, and we conducted one-way and probabilistic sensitivity analyses on the parameter inputs. | Outcomes included the impact of training on patient life years, quality-adjusted life years (QALYs), treatment rates, fracture incidence, and costs from the Department of Veterans Affairs perspective. |
| Palliative Care | | | | |
| Carey, 2017, Australia | Controlled before/after | To determine the impact of establishing a palliative and chronic disease respite facility on the extent to which patients accessed hospital resources. | Code: Reorganization of Services  The respite facility was staffed by nursing staff, which enabled patients attending the facility to have some of their medical needs attended to. While at the facility, participants could engage in unstructured leisure and social activities such as cooking, watching television, and socializing with staff and other patients. | Respite service use and hospital use data were collected over two periods: the 12 months before the establishment of the service and the first 10 months of the operation. The National Weighted Activity Unit (NWAU, a standardized measure of hospital expenditure) was used to determine the mean cost savings. The impact of the respite service on admissions to the Emergency Department (ED), to the Wards, and the Intensive Care Unit (ICU) was measured via hospital records. The number of ventilator hours consumed was also recorded. |
| Preventative Care | | | | |
| Nagykaldi, 2017, United States | Uncontrolled before/after | To implement and evaluate a sustainable, rural community–based patient outreach model for preventive care provided through primary care practices (PCPs) in a rural county in Oklahoma. | Code: Coordination/Referral Pathways  A Wellness Coordinator (WC) working with PCPs, the county health department, the county hospital, and a health information exchange (HIE) organization helped county residents receive evidence-based preventive services. The WC used a community wellness registry connected to electronic medical records via HIE and called patients at the county level based on PCP-prioritized and tailored protocols. The registry flagged patient-level preventive care gaps, tracked outreach efforts, and documented the delivery of preventive services throughout the community. | Return on investment (ROI) for prioritized preventive services was estimated in participating organizations. Rates of preventive service delivery for selected services were calculated for a pre-implementation baseline year and the intervention year for each participating practice and the hospital. |
| Gray, 2010, Canada | RCT | To evaluate the cost-effectiveness of Anticipatory and Preventive Team Care (APTCare). | Code: Coordination/Referral Pathways  At-risk patients were randomly assigned to receive usual care from their family physicians or APTCare from a collaborative team. APTCare consisted of being assigned to the care of 1 of 3 NPs, the pharmacist, and their usual family physicians. Care provided by the NPs and pharmacists was delivered almost exclusively in the patients' homes, while patients continued to see their family physicians in the office. The main objective of the intervention was to ensure evidence-based disease management and strong social support for patients. Additionally, some patients received a telehealth system in the home for remote monitoring of clinical parameters (e.g., blood pressure, weight, glucose levels, and blood oxygen levels) by the NPs. | Cost-effectiveness and the net benefit to society of the APTCare intervention. Costs measured included clinical-related costs (such as medication, lab tests, physician salary etc.), APTCare-specific costs (such as staff training, medical supplies etc.), overhead, and human resource costs. Each participant's quality of care score was also calculated, and patient characteristics were recorded. |
| Upper Respiratory Tract Infections | | | | |
| Zhang, 2018, China | RCT | To assess the cost-effectiveness of our intervention in reducing antibiotic prescribing in rural primary care facilities as measured by the intervention's effect on the antibiotic prescription rates for childhood URTIs. | Code: Healthcare Provider Training + Patient Education/Navigation + Audit and Feedback  The intervention included concise, evidence-based clinical guidelines on URTI management. It facilitated training on using/applying the guidelines during consultations and monthly peer review meetings assessing providers' antibiotic prescription rates (APR). Patients and caregivers received information on appropriate antibiotic use verbally and via an educational leaflet. Additionally, a video with key  messages on the appropriate use of antibiotics were played daily in the township hospitals' waiting rooms and public areas. | The primary outcome was the cost per percentage point decrease in the antibiotic prescribing for childhood URTIs in the intervention arm compared to the control arm. |
