# Supplementary material for: Interventions to improve primary healthcare in rural settings: A scoping review
Source: PLoS One. 2024 Jul 11;19(7):e0305516. doi: 10.1371/journal.pone.0305516 (PMC11239038; doi:10.1371/journal.pone.0305516)
Supplement: Attachment — Submitted filename: Response to reviews 2.docx [file pone.0305516.s017.docx]

Dear Dr. Yourkavitch

We would like to thank you and the reviewers for the thoughtful comments and suggestions on our review. Our point-by-point response is below:

Please review your reference list to ensure that it is complete and correct. If you have cited papers that have been retracted, please include the rationale for doing so in the manuscript text, or remove these references and replace them with relevant current references. Any changes to the reference list should be mentioned in the rebuttal letter that accompanies your revised manuscript. If you need to cite a retracted article, indicate the article’s retracted status in the References list and also include a citation and full reference for the retraction notice.

Thank you. We have reviewed our reference list and updated some citations that were not properly ordered. No papers were retracted from our reference list.

Please include a separate legend for each figure within your manuscript file.

As requested, we have added a legend for each figure within the manuscript.

**Reviewer #1:** Thank you for your thoughtful responses to my comments and to those of the other review and the editor. I find the manuscript clearer now. I especially like the clear focus on pointed out issues for further in-depth review. I find the discussion more in-depth and the findings in each sub-section of the results better summarized.
I only add that I think that one of my previous comments was not completely addressed. I am speaking of my first comment:
• The authors don’t clearly and explicitly state the definition or framework they are using for Primary Care or Primary Health Care. They seem to be screening articles mainly by level of care and excluding specialist or hospital-delivered care. But are they using any kind of characteristics of primary care, like the classic 4 C’s (first contact, coordination, comprehensiveness, and continuity). If the common feature of included articles is only that they are at the most basic level of care (i.e., “first contact”), but not necessarily that the care is coordinated or comprehensive, for instance, then are all included articles truly about “PHC” or “PC” or would they more accurately be described as about “basic health care” or “non-specialist care.”

I appreciate that you added a sentence in the limitations. But I actually don't find a clear delineation/definition of Primary Care/Primary Health Care to be a limitation. I think that having (and stating) a definition makes clear the scope of the Scoping Review. I think that a statement of the definition you are using belongs in the Introduction. I think that some of what you wrote in your response to me should go there - Namely, that you are using the Canadian Foundation for Healthcare Improvement's definition and that this adheres to the common PC principles of First Contact, Comprehensiveness, Continuity, an Coordinated. But that you also "loosened" your criteria (I would be explicit about HOW exactly you loosened the criteria......I think that this concern is most relevant when I see in the manuscript various entries for articles about mental health. Were these done in a setting that in which the services were delivered by mental health specialists or were they integrated into a comprehensive primary care service delivery setting or system? I bring up mental health as an example, but I also have a question in my mind when I see the entries for cancer screening. I have seen such programs carried out as part of "community outreach" by tertiary care hospitals. I don't know if you encountered any examples in the articles you reviewed. If so, did you exclude them or include them? I think you should make state such considerations and your decisions about them clearly in the introduction.
Other than this one remaining comment, I am quite satisfied with your responses and the improvements in the manuscript.

Thank you for helping to clarify this point for us. Just to note, in our first response to this comment we did include what we wrote in our response into the methods section of the manuscript – I am not sure if that was clear from our first response. However, we also agree that we can bring this up the in the introduction as well. We didn’t want to repeat the information in both places, so we have introduced the Canadian Foundation for Healthcare Improvement's definition in the introduction and then left the information about how we used it to guide the actual inclusion/exclusion criteria and how we “loosened” the criteria in the methods section. We hope the additional information provides a clearer understanding of our selection process.

In terms of your specific comment about what was included and excluded with respect to some of the topics that might have services/interventions/programs commonly delivered in both primary care and other areas of the health system, we included the study if:

- any part of the intervention was delivered by a primary care provider individually or as part of a comprehensive or collaborative or multidisciplinary team. Note: This was the case for many of the studies that included interventions related to mental health. We excluded studies in which the intervention was solely testing an intervention delivered by a mental health specialist that had no connection to a primary care provider or setting

OR

- it was testing a multilevel intervention and one of the interventions was delivered in primary care. For example, if the study aimed to improve referral rates/time to referral from primary care to another care service (secondary or tertiary), this was treated as an intervention to improve coordination/referral pathways of primary care. Even if the study also went on to measure patient outcomes from the secondary or tertiary care intervention.

OR

- If the intervention was initiated outside a first-contact primary care site but the aim was to improve access to primary care or improve follow-up management of conditions, etc. (this was rare but did come up once or twice in relation to screening)

OR

- If the study stated it was delivered or taking place in a primary care setting and no further details were provided

We believe the additional inclusion/exclusion criteria we added to Table 1 should cover this at a high level, however, we could include these more very specific screening rules as an appendix if that is deemed helpful.

**Reviewer #2:** Detailed additions that address earlier concerns.
Typo on line 599: the increase in the yearly number of rural primary care must be...

Thanks for catching this. We have fixed this typo (see tracked changes).
